# Direct electrochemical hydrodefluorination of trifluoromethylketones enabled by non-protic conditions †

**DOI:** 10.1039/d1sc01574e

**Published:** 2021-07-06

**Authors:** John R. Box, Alexander P. Atkins, Alastair J. J. Lennox

**Affiliations:** School of Chemistry, University of Bristol Cantock's Close Bristol BS8 1TS UK a.lennox@bristol.ac.uk

## Abstract

CF_2_H groups are unique due to the combination of their lipophilic and hydrogen bonding properties. The strength of H-bonding is determined by the group to which it is appended. Several functional groups have been explored in this context including O, S, SO and SO_2_ to tune the intermolecular interaction. Difluoromethyl ketones are under-studied in this context, without a broadly accessible method for their preparation. Herein, we describe the development of an electrochemical hydrodefluorination of readily accessible trifluoromethylketones. The single-step reaction at deeply reductive potentials is uniquely amenable to challenging electron-rich substrates and reductively sensitive functionality. Key to this success is the use of non-protic conditions enabled by an ammonium salt that serves as a reductively stable, masked proton source. Analysis of their H-bonding has revealed difluoromethyl ketones to be potentially highly useful dual H-bond donor/acceptor moieties.

## Abstract 2

The electrochemical hydrodefluorination of trifluoromethylketones under non-protic conditions make this single-step reaction at deeply reductive potentials uniquely amenable to challenging electron-rich substrates and reductively sensitive functionalities.

## Graphical abstract

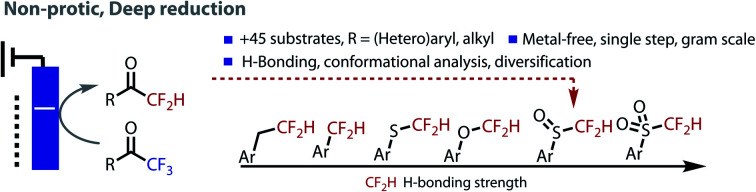

The difluoromethyl group (CF_2_H) has attracted significant recent attention in medicinal chemistry,^1,2^ which complements the well-documented importance and growing use of fluorine in small molecule pharmaceuticals.^3–6^ The CF_2_H group is an H-bond donor^7,8^ that is also lipophilic,^9,10^ a unique combination that positions it as an increasingly valuable tool within drug-discovery.^11^ CF_2_H has been used as a bioisostere of OH and SH in serine and cystine moieties, respectively, as well as NH_2_ groups, where greater lipophilicity and rigidity provide advantages to pharmacokinetics and potency.^12–14^

The hydrogen-bond acidity of CF_2_H groups is exceptionally dependent on the atom or group to which it is appended (Fig. 1A).^1,2^ The H-bond acidity of alkyl-CF_2_H groups is half that of O–CF_2_H and even a quarter of SO_2_–CF_2_H groups.^1^ This mode of control allows the H-bonding strength and, therefore its function, to be finely tuned. While much research has focused on the synthesis, behaviour and use of XCF_2_H groups, where X = O, S, SO, SO_2_, Ar, it is surprising that the corresponding carbonyl containing moiety (X = CO) has remained relatively elusive in these contexts. Not only would difluoromethyl ketones (DFMK) be expected to provide a relatively strong H-bond, but the carbonyl unit provides a complementary, yet proximal mode of intermolecular interaction (Fig. 1B). Indeed, the dual action of neighbouring H-bond donor and acceptor functionalities provides the fundamental basis for many biological systems, including in the secondary structure assembly mechanisms for proteins and DNA/RNA nucleobase pairing, as well as in enzyme/substrate complexes. Indeed, the DFMK functionality has demonstrated important utility in biological applications, including anti-malarial and -coronaviral properties.^15^ Finally, the carbonyl provides a useful synthetic handle for further derivatization.

**Fig. 1:**
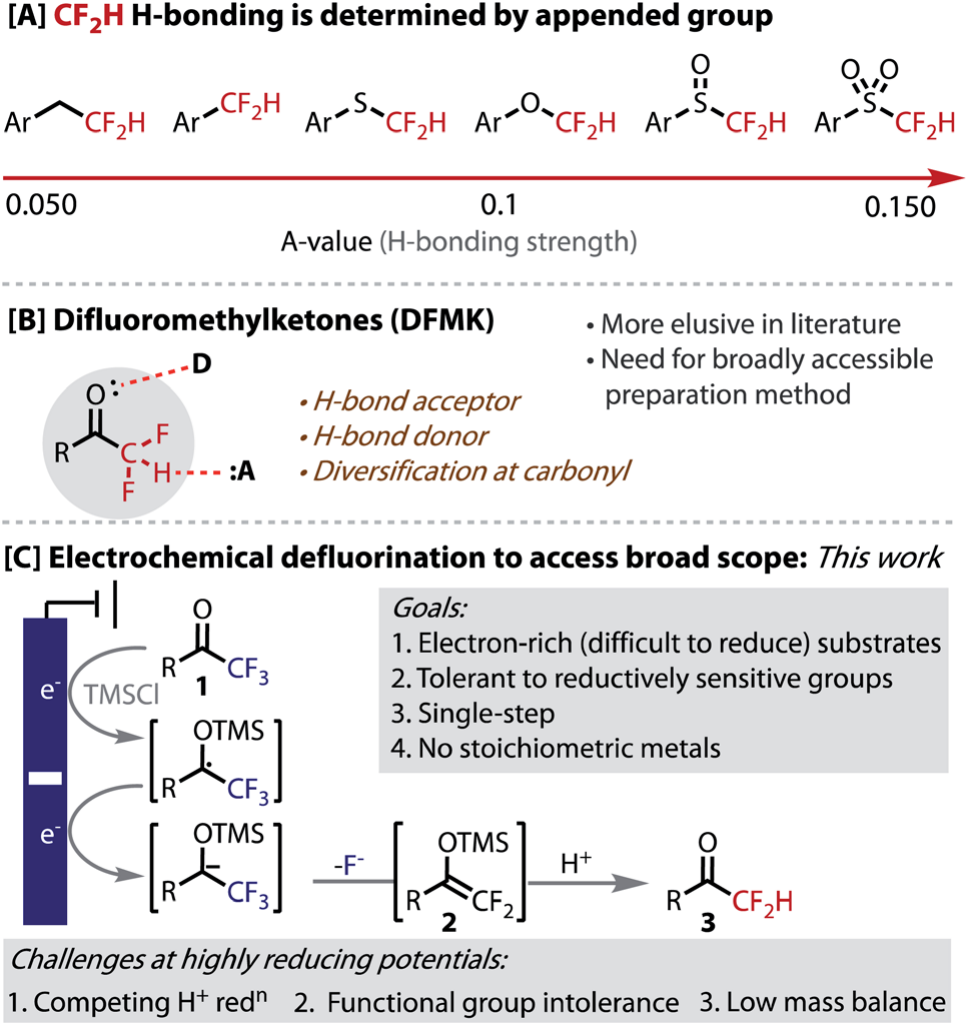
H-Bonding in DFMKs and their synthesis *via* hydrodefluorination.

While some progress has been made on the synthesis of DFMKs,^16^ there still remains a need for a general and more broadly accessible route to their preparation. Current strategies for DFMK preparation require multi-step processes, expensive reagents, installation of activating groups, or are inherently low yielding.^15*a*,16–25^ The hydrodefluorination of trifluoromethyl ketones (**1**) potentially represents the most accessible strategy, as the starting materials are most readily prepared through a high-yielding trifluoroacetylation of C–H or C–X bonds.^26–29^ In 2001, Prakash demonstrated the viability of this approach using 2 equivalents of magnesium metal as stoichiometric reductant to drive the defluorination, with a second hydrolysis step (HCl (3–5 M) or fluoride, overnight stirring) to reveal the product.^30^ The scope in this 2-step process (6 substrates) reflects the limitations of using a reductant, such as Mg, that has a fixed reduction potential, as well as incompatibilities arising from Mg/halide exchange with aryl halides. Similar limitations with the use of electron-rich substrates were revealed in related contributions from Uneyama.^31^

In order to access more electron-rich and reductively challenging substrates, such as those containing medicinally relevant heterocycles, we postulated that electrochemical reduction could be employed (Fig. 1C). Electrosynthesis is becoming an increasingly valuable enabling technology and has seen a recent resurgence due to the precise control, unique selectivity, and the potential scalability and sustainability benefits that it offers.^32–36^ This strategy would avoid the undesirable use of stoichiometric metals and the ‘deep-reduction’ potentials required are readily accessed by simply selecting the applied potential. Pioneering early work from Uneyama on the cathodic formation of silylenol ether intermediate **2**, suggested this approach could be viable.^37,38^ The fundamental challenge in designing a practical, single-step process under highly reducing potentials (<−2.0 V *vs.* Fc/Fc^+^), is to avoid the reduction of the proton source, which would otherwise compete to generate H_2_ gas and leave the starting material untouched. Uneyama does not demonstrate hydrodefluorination, presumably due to this problem. Additional challenges posed by ‘deep-reduction’ include a lack of tolerance for reduction-sensitive functionality (alkene, C–X bonds *etc.*), low mass balance due to substrate decomposition and the undesirable use of sacrificial metal anodes.^39^ Solving these problems should provide generally applicable, safe and scalable conditions for the hydrodefluorination of readily accessible trifluoromethyl ketones (**1**).

Given the electron-rich nature of indoles, their ubiquity in bioactive compounds, and their ease of functionalisation, we chose indole **1a** as the model substrate for optimisation. The highly reductive potentials required will render it a challenging substrate, which should lead to more general conditions suitable for other important substrate classes. Indeed, when we applied the Mg conditions of Prakash to this substrate, no silyl enol ether intermediate (**2a**) was observed, nor product **3a**, and the starting material remained completely untouched (Table 1, entry 1). Moving to an electrochemical set-up, the use of a sacrificial Mg anode in an undivided cell again returned no defluorinated product (entry 2). The applied potential was sufficiently negative to reduce the evolving Mg^2+^ ions, and so the substrate was again left untouched.

**Table 1:** Optimisation reactions

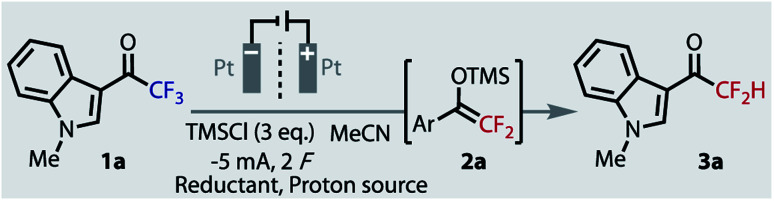					
Entry	Conditions different from above	Reductant	Proton source	**1a** a/%	(**2a**) **3a**^a^/%
1	*Mg* ^*0*^, THF, no electricity *(Prakash conditions for***3***)*	Mg^0^	—	100	(0) n/a
2^b^	Undivided cell, TBAPF_6_	Sacrificial Mg anode	—	100	(0) n/a
3^b^	Pb:C (cath:an), 0 ^o^C, 30 mA *(Uneyama conditions for***2***)*	TBABr (4 eq.)	—	33	(32) 0
4^b^	—	TBABr (2 eq.)	(a) Acetic acid; (b) oxalic acid.	51; 100	0; 0
5^b^	—	TBABr (2 eq.)	Dimethylurea	82	0
6^b^	—	TBABr (2 eq.)	TEAPF_6_ (4 eq.)	49	45
7	TMSCl (0 eq.)	TBABr (2 eq.)	TEAPF_6_ (4 eq.)	83	0
8^b^	TMSCl (6 eq.)	TBABr (2 eq.)	TEAPF_6_ (4 eq.)	49	49
9^c^	**TMSCl (3 + 3 eq.)**	**TBABr (2 eq.)**	**TEAPF**_**6**_ **(4 eq.)**	**0**	**97**
10^c^	Entry 9, but Pt:Gr (cath:An)	TBABr (2 eq.)	TEAPF_6_ (4 eq.)	0	94
11^c^	Entry 9, but Ni:Pt (cath:An)	TBABr (2 eq.)	TEAPF_6_ (4 eq.)	0	83
12^c^	Entry 9, but Stainless Steel:Pt (cath:An)	TBABr (2 eq.)	TEAPF_6_ (4 eq.)	0	85
13^c^	Entry 9, but Gr:Pt (cath:An)	TBABr (2 eq.)	TEAPF_6_ (4 eq.)	0	18

a ^19^F NMR yields.

b TMSCl only added to cathodic chamber.

c TMSCl added to both cathodic and anodic chambers.

The electrochemical conditions of Uneyama for preparing silylenol ethers (**2**) were applied to our indole **1a** (entry 3). Unsurprisingly, no hydrodefluorinated product was observed, however intermediate **2a** was formed in a 32% yield. In an effort to improve this yield we explored several solvents, reductants, additives and electrode materials, all of which were conducted in a divided cell at constant current and ambient temperature.^40^ In addition, as we were keen to develop a single-step protocol, by avoiding the second hydrolysis step that can readily form homo-coupled aldol side products,^38^ we surveyed a range of added proton sources for *in situ* delivery of **3a**. The addition of carboxylic acids, such as acetic or oxalic acid (entry 4), gave no desired product, as the competing reduction of protons to H_2_ gas dominated. Dimethylurea was recently used as a proton source in an electrochemical ‘deep-reduction’,^41^ but it returned no trace of intermediate **2a** or product **3a** (entry 5). We hypothesized that increasing the conductivity of the system, with additional tetraalkylammonium salts (from 2 to 4 eq.), the formation of intermediate **2a** may be facilitated by avoiding large cell potentials. While this change did facilitate a lower cell potential, we discovered these salts behaved as reductively stable yet competent masked proton donors: 4 eq. NEt_4_PF_6_ gave 45% yield of product **3a**, with no sign of intermediate **2a** (entry 6). The detection of triethylamine in solution suggests donation through a Hoffmann elimination.^42^ With the exception of NMe_4_^+^, other tetraalkylammonium salts were also competent proton donors (NEt_4_^+^ > NBu_4_^+^ > NPr_4_^+^).

A critical improvement to the yield was observed when the use of the radical anion trapping agent, TMSCl, was optimised. With no TMSCl, **3a** was not observed (entry 7), and a loading of 6 equivalents saw little improvement over 3 equivalents (entry 8 *vs.* 6). Experiments hitherto described were conducted with TMSCl added only to the cathodic chamber (entries 2–8). Only when the 6 equivalents was split between both chambers was a drastic improvement observed (entry 9), giving an optimised yield of 97%. Notably, the increase in conversion still occurred with only 2 F, implying that a lower steady-state concentration may be important in the cathode chamber. To test this hypothesis, TMSCl was slowly added to the catholyte by syringe-pump addition over the course of the reaction, which gave a similar yield of 94%.^40^ Although intermediate **2a** is transient and was never observed, the importance of TMSCl to trap and stabilise reduced **1a** was revealed by DFT (B3LYP/6-311+g(d)) calculations,^40^ which suggested a thermodynamically highly challenging reaction in its absence.

The oxidation of bromide to tribromide occurs on the anode, which is an ideal counter-electrode process: not only is bromide an inexpensive and metal-free sacrificial reductant, but as the produced Br_3_^−^ is anionic, it does not rapidly migrate to the cathodic chamber, preventing unwanted side reactions.^43^ The generated Br_3_^−^ can even be used in follow-up bromination reactions.^44^ An increase in the applied cell potential during the reaction signifies the consumption of Br^−^, and the oxidation of Br_3_^−^ to Br_2_ (Fig. 2).^45^ Despite needing 3 equivalents of Br^−^ to form 2 equivalents of Br_3_^−^ after 2 F, the loading of Br^−^ could be reduced to 2 equivalents without affecting yield. No over-reduction of **3a** to the monofluoromethyl ketone was observed, which is significant considering the small difference in reduction potentials.^40^ This emphasises the importance of a flat chronopotentiometry trace that is achieved with Br^−^ oxidation. Other reductants were found to be sub-optimal, including diisopropylamine and oxalic acid.^40^

**Fig. 2:**
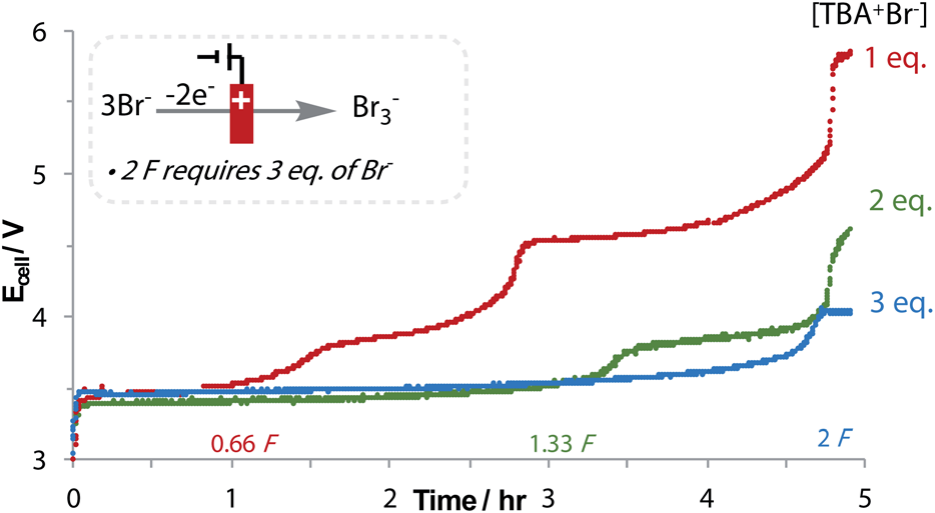
Reaction of **1a** to **3a** with 3 different Br^−^ concentrations.

A graphite anode performed equally well as platinum for the counter electrode reaction (entry 10). Only marginally reduced yields were observed with nickel and stainless-steel cathodes (entries 11 and 12), however, a drastic decrease in the yield was observed with a graphite cathode (entry 13), possibly due to substrate grafting.^39^

We proceeded to explore the substrate scope with our optimized conditions, Fig. 3. As expected, our electrochemical conditions were suitable for the hydrodefluorination of electron-poor acetophenone derivatives (**1b**, **1c**). However, unlike with the use of Mg,^30^ substrates containing electron donating substituents are now well tolerated (**1d–k**). In addition, no hydrodebromination was observed for **1b**, highlighting the selectivity and orthogonality granted by the use of our Mg-free, non-protic conditions. A selection of extended π-systems was tolerated, producing pyridyl **3l**, biphenyl **3m**, benzothiophene **3n**, primary amine **3o**, and pyrimidines **3p** and **3q** and in moderate to excellent yields. Chromoionophore dye **1r** and stilbene **1s** and were transformed in excellent yield, demonstrating tolerance to reductively sensitive alkenes, which would otherwise hydrogenate under protic electrochemical conditions.^46^ Anthracenyl **1t** and naphthyl substrates **1u** and **1v** all transformed efficiently in good to excellent yields, the latter of which underwent direct double hydrodefluorination. 4.5% over-reduction was observed in the double defluorination product, **3v**, which was the only instance where this side-product was observed in greater than 1% quantities.^40^ The good mass-balance and faradaic efficiency is notable considering the delocalization of charge around extended π-systems increases the likelihood of grafting.^47^

**Fig. 3:**
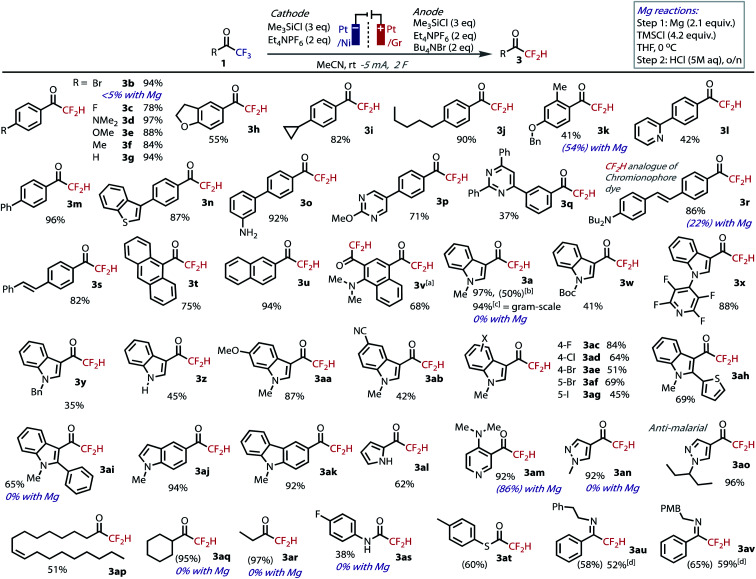
Isolated yields of DFMKs tested under the reaction conditions at 0.5 mmol scale. NMR yields in parentheses. ^a^Reaction run at 10 mA; ^b^reaction run in IKA Divided ProSyn: quantitative yield based on RSM; ^c^5 mmol scale, Ni foil:Gr (cath:an); ^d^isolated as the corresponding ketone following purification on silica.^49^

The model indole substrate **1a** gave an excellent yield of DFMK at 0.5 mmol scale, which gave equally high yields when scaled up 10-fold (5 mmol), thereby demonstrating the robustness and practicality of the technique. We were also able to successfully prepare **3a** in a commercially available divided cell set-up.^40^ Alternative groups on nitrogen, including Boc, perfluoropyridyl and benzyl (**3w–y**), as well as the free indole **3z**, were well tolerated and gave moderate to good yields of **3**. Tosyl and acetyl groups on nitrogen were less well tolerated.^40^ As with the acetophenones, indoles with electron donating (**1aa**) and withdrawing (**1ab**) groups proceeded to product. Methoxy demethylation of **3aa** should lead to the corresponding phenol,^48^ which is difficult to prepare using other methodologies due to competing side-reactions. Halide substitution also successfully yielded DFMKs (**3ac–ag**). The inclusion of the aryl-iodide functionality is especially notable due to its facile reduction; when a silver cathode was used to convert **1ag**, hydrodeiodination was observed, but which was absent under our non-protic conditions with a Pt cathode. Increased steric bulk around the reacting center in thiophenyl and phenyl-substituted substrates **1ah** and **1ai** had no negative influence and gave good yields of product.

Heterocyclic trifluoromethylketones were successfully hydrodefluorinated under the standard conditions, including indole **3aj**, carbazole **3ak**, pyrrole **3al**, pyridine **3am**, and pyrazoles **3an** and **3ao**, the latter of which leads to a compound with anti-malarial activity.^15*a*^ Alkyl trifluoromethylketones are more difficult to reduce compared to aromatic trifluoromethylketones, and are therefore challenging substrates to hydrodefluorinate, and impossible to convert using other methods. Nevertheless, oleyl **1ap**, cyclohexyl **1aq** and ethyl **1ar** substrates were all amenable to the conditions, although the smaller alkyl products were cumbersome to isolate due to their volatility. The non-protic optimized conditions ensured no loss of mass-balance at these enhanced reduction potentials (|*E*_cell_| = *ca.* 3.4–3.7 V for alkyl substrates *vs. ca.* 2.3–2.7 V for acetophenones and indoles). Finally, we tested the conditions on trifluoroacetamide **1as**, thioester **1at** and imines **1au** and **1av**. For each of these, the corresponding product was returned in moderate to good yields. Despite some complications in their isolation, these results are notable considering their difference in structure and lack of precedent. Unsuccessful substrates included a nitro-substituted indole, which was insoluble in the reaction medium, and hydrated TFMKs.^40^

We tested a variety of substrates with the Mg-mediated conditions reported by Prakash to gauge the level of complementary between the methods.^30^ While acetophenone derivatives **1k** and **1am** were amenable to reduction with Mg, bromide substitution in **1b** was unsurprisingly not tolerated with Grignard formation dominating. Indoles – **1a**, **1ai**, pyrazole – **1an**, alkyl – **1aq**, **1ar** and anilide – **1as** based trifluoromethylketones were untouched by Mg in all cases, with starting materials recovered only.

To explore the value of the DFMK moiety in synthesis, we derivatized it in a variety of ways, Fig. 4. Resubjecting the product **3a** to our non-protic hydrodefluorination conditions led to monofluorinated product **4**, providing an alternative to the use of electrophilic fluorine sources.^50^ Reduction of the ketone in **3ae** to the methyl ether and alcohol successfully gave products, **5** and **6**, respectively. The dithiane of **3a**, which is a useful synthetic intermediate, was formed in excellent yield (**7**). A Corey–Chaykovsky methenylation gave epoxide **8** in good yield. A Horner–Wadsworth–Emmons reaction transformed the carbonyl to give alkene **9**. Nucleophilic attack of the ketone was demonstrated with a trifluoromethylation reaction to give highly fluorinated alcohol **10**. Orthogonal reactivity was also demonstrated with a Suzuki–Miyaura cross-coupling that gave biaryl **11**. Interestingly, deuterium was not exchanged into **3a** when stirred in a mixture of D_2_O and MeCN, providing evidence for a less favourable enolization.

**Fig. 4:**
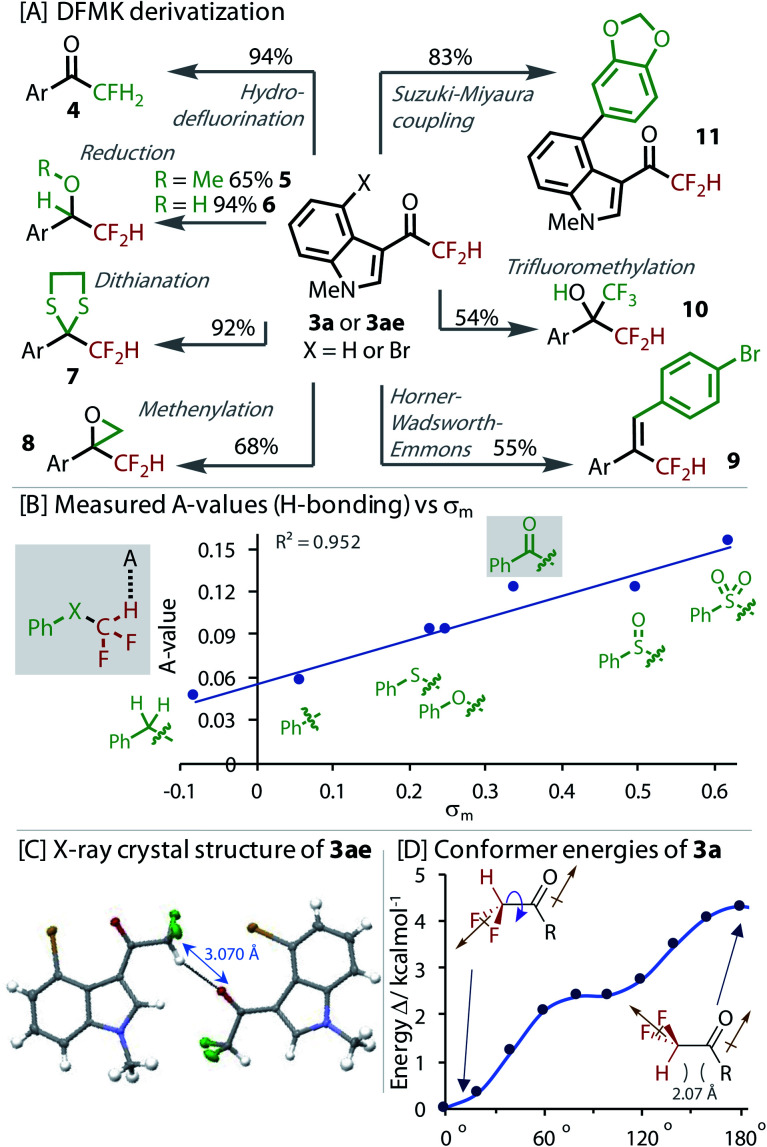
[A] Derivatization of DFMKs. X = H (**3a**) for **4**, **7**, and **8**, X = Br (**3ae**) for others; [B] H-bond strength (*A*-value) correlated to *σ*_m_ Hammett parameter; [C] intermolecular H-bond revealed in X-ray crystal structure of **3ae**; [D] DFT calculated (B3LYP/6-311+g(d)) relative energies of conformers with rotation around HC–CO bond. Brown arrows indicate direction of dipole.

The H-bond strength (*A*-value) was measured for a series of phenyl substituted X–CF_2_H derivatives using the NMR method from Abraham, Fig. 4B.^51–53^ These experiments confirmed the sensitivity of the H-bonding ability to the identity of X. DFMK **3g** and sulfoxide–CF_2_H were found to be comparable H-bond donors, which were only marginally less than the sulfone–CF_2_H. The H-bond strength correlated best with the *σ*_m_ parameter, reflecting the strong influence of inductive effects. Multiple regression analysis showed that any contribution of *σ*_p_ was statistically insignificant (*P* value = 0.33).

Analysis of the X-ray crystal structure of **3ae**, showed an inter-molecular H-bond between the CF_2_H and a carbonyl from a neighbouring molecule (Fig. 4C). DFT was used to calculate the relative conformer energy with rotation about the (O)C–CF_2_H dihedral bond (Fig. 4D). The lowest energy conformer eclipsed the H with the carbonyl, implying the possibility of an energy lowering intra-molecular H-bond. However, analysis of the other derivatives in the set (C(O)CH_3_, C(O)CFH_2_ and C(O)CF_3_) revealed that the alignment of dipoles was the dominant effect (brown arrows, Fig. 4D).^40^ The absence of an unusually low or even negative *A*-value also provides evidence against an intramolecular H-bond.^51^ Interestingly, in the solid-state structure (Fig. 4C), the highest energy conformer (with dipoles aligned) is adopted, highlighting the stronger propensity of this moiety to engage in H-bonding interactions.

In conclusion, we have developed a mono-selective hydrodefluorination to access a broad scope of DFMKs, enabled by non-protic electrochemical conditions at deeply reducing potentials. These moieties have been studied and diversified and reveal themselves to be potentially useful dual H-bond donor/acceptor moieties. This is especially interesting considering the structurally related trifluoromethylketones are known reversible protease inhibitors;^54,55^ thus, the additional H-bonding moiety could enhance interaction within enzymatic active sites.^15^

## Data availability

All underlying data are provided as ESI accompanying this paper.

## Author contributions

J. R. B. and A. P. A. performed the experimental work, J. R. B. conducted the computational calculations, J. R. B. and A. J. J. L. wrote the manuscript, A. J. J. L. conceived and directed the project.

## Conflicts of interest

There are no conflicts to declare.

## Supplementary Material

SC-012-D1SC01574E-s001

SC-012-D1SC01574E-s002
