# Supplementary material for: Direct electrochemical hydrodefluorination of trifluoromethylketones enabled by non-protic conditions
Source: Chem Sci. 2021 Jul 6;12(30):10252–8. doi: 10.1039/d1sc01574e (PMC8336478; doi:10.1039/d1sc01574e)
Supplement: SC-012-D1SC01574E-s001 [file SC-012-D1SC01574E-s001.pdf]

Electronic Supplementary Material (ESI) for Chemical Science.
This journal is © The Royal Society of Chemistry 2021

Supporting Information

Direct Electrochemical Hydrodefluorination of Trifluoromethylketones Enabled by Non-protic Conditions

John R. Box, Alexander P. Atkins, Alastair J. J. Lennox*

University of Bristol, School of Chemistry, Cantock's Close, Bristol, BS8 1TS, UK

Contents

General Experimental Details ... 3

Full Optimisation Studies ... 5

Cyclic voltammograms of model substrates and defluorinated derivatives ... 6

Synthesis and Characterisation of Substrates ... 7

General Defluorination Procedures ... 43

Characterisation of Products ... 45

Less successful and unsuccessful substrates ... 88

Electrochemical Set Up ... 89

Product Diversification Experiments ... 92

Determination of H-bonding strength (A value) ... 113

Computational Studies ... 117

CIF of 3ae ... 140

NMR Spectra of Novel Substrates ... 142

NMR Spectra of Novel Products ... 174

References ... 228

General Experimental Details

Techniques

Manipulations involving air and moisture sensitive materials were conducted employing standard Schlenk-line and glovebox techniques, using vacuum lines attached to a double manifold with greaseless J. Youngs valves equipped with an oil pump (0.1 mmHg) under an atmosphere of dry nitrogen. All glassware was dried overnight before use, in a 180º oven and then allowed to cool under vacuum at 0.1 mmHg. The removal of solvents in vacuo was achieved using a Büchi rotary evaporator (bath temperatures up to 40 °C) at a pressure of 15 mmHg (diaphragm pump), or at 0.1 mmHg (oil pump) on a vacuum line at room temperature. The addition of < 200uL of liquids was achieved using a Gilson PIPETMAN p20, otherwise standard syringe practices were employed.

Solvents

THF (tetrahydrofuran), CH_2Cl_2, CH_3CN and Et_2O was dried using an Anhydrous Engineering alumina column drying system situated in the University of Bristol's chemistry department. All solvents were collected using Strauss flasks using a gastight J. Youngs valve. CH_3CN was degassed by four freeze-pump-thaw cycles under N_2. Deuterated solvents for NMR analysis were purchased from Sigma Aldrich.

Chromatography

TLC analysis was performed on Merck Silica gel $60F_{254}$ glass backed plates. Visualisation was achieved by UV fluorescence (254 nm). Flash column chromatography was conducted using Sigma 60 silica: 230-400 mesh (40-63 μm) or using a Biotage Selekt automated flash purification system using Biotage Sfar Duo pre-packed columns of size 5 g or 25 g.

Reagents

All reagents were purchased from TCI UK, Apollo Scientific, Sigma Aldrich, Alfa Aeser or Fluorochem and used as received unless otherwise stated. Anhydrous TMSCl was purchased from Sigma Aldrich and stored under N_2 over 3A molecular sieves. Electrolyte salts ($TEAPF_6$, $TBAPF_6$, TBAB) were purchased from Sigma Aldrich and stored in a vacuum desiccator between uses.

Analysis

NMR spectra were recorded on Bruker Nano 400 or Bruker Advance III HD 500 cryo spectrometers. Chemical shifts (δ) are quoted in parts per million (ppm), referenced to the residual solvent peak (1H and 13C NMR) and coupling constants (J) are given in Hz. Multiplicities are abbreviated as: s (singlet), d (doublet), t (triplet), q (quartet), m (multiplet) or combinations thereof. NMR shifts for novel compounds have been assigned with the use of the appropriate 2D NMR experiments, such as COSY, HSQC and HMBC. Infrared spectra were recorded using a Perkin Elmer Spectrum Two FTIR spectrometer.

Electrochemical techniques

All cyclic voltametric (CV) and chronopotentiometric measurements were performed at room temperature using an Autolab M101 or MultiPalmsens 4 instrument and the ElectraSyn 2.0 (purchased from IKA). CV experiments were carried out with a working electrode (GC = glassy carbon, Pt, Au, 1-3 mm diameter), a counter electrode (platinum wire) and a 0.1 M Ag/$AgNO_3$ reference electrode. All working electrodes were polished before each experiment. Before each CV, the solution was stirred for approximately 10 seconds, whilst being degassed by a stream of N_2.

Full Optimisation Studies

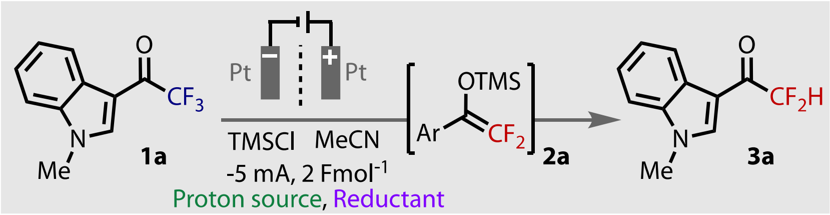

Entry	Conditions different from above	Yield/% *1a*	*(2a) 3a*
1	Mg^0, THF, no electricity (*Prakash conditions for* ***2a***)	100	(0) n/a
2[a]	Undivided cell, sacrificial Mg anode, TBAPF$_6$ (4 eq.)	100	(0) n/a
3[a]	Pb:C (Cath:An), TBABr (4 eq.), 0 °C, 30 mA (*Uneyama conditions for* ***2a***)	33	(32) 0
4[a]	a) TMSCl (3 eq.), Acetic acid (1 eq.), TBABr (2 eq.)<br>b) TMSCl (3 eq.), Oxalic acid (1 eq.), TBABr (2 eq.)	51;<br>100	(0) 0;<br>(0) 0
5[a]	TMSCl (3 eq.), Dimethylurea (2 eq.), TBABr (2 eq.)	82	(0) 0
6[a]	TMSCl (3 eq.), TEAPF$_6$ (4 eq.), TBABr (2 eq.)	49	(0) 45
7	TMSCl (0 eq.), TEAPF$_6$ (4 eq.), TBABr (2 eq.)	87	(0) 0
8[a]	TMSCl (6 eq.), TEAPF$_6$ (4 eq.), TBABr (2 eq.)	49	49
**9[b]**	**TMSCl (3 + 3 eq.), TEAPF$_6$ (4 eq.), TBABr (2 eq.)**	**0**	**(0) 97**
10[b]	Entry 9 but Pt:Gr (Cath:An)	0	(0) 94
11[b]	Entry 9 but Ni:Pt (Cath:An)	0	(0) 83
12[b]	Entry 9 but Stainless steel:Pt (Cath:An)	0	(0) 85
13[b]	Entry 9 but Gr:Pt (Cath:An)	0	(0) 18
14[b]	TMSCl (3 + 3 eq.), Dimethylmalonate (1 eq.), TBABr (2 eq.)	0	(0) 90
15[b]	TMSCl (3 + 3 eq.), TEAPF$_6$ (4 eq.), NH*i*Pr$_2$ (2 eq.)	28	(0) 65
16[a]	Entry 6 but extra 3 eq. TMSCl added by syringe pump to cathode	0	(0) 93
17[b]	TMSCl (3 + 3 eq.), TEAPF$_6$ (4 eq.), TBABr (2 eq.), -10 mA	0	(0) 92[c]
18[b]	Entry 9 but TEABF$_4$ (4 eq.) not TEAPF$_6$ (4 eq.)	0	(0) 90[c]

Table S1. 1a (0.5 mmol), TEAPF$_6$ (1 (cathodic chamber) + 1 mmol (anodic chamber)), TBABr (1 mmol), TMSCl (1.5 + 1.5 mmol), MeCN (2.5 + 2.5 mL), N_2, RT. Pt:Pt (coil), -5 mA CCE, 2 F (19300 s). ^{19}F NMR yields relative to internal C_6F_6 standard. [a] TMSCl only added to cathodic chamber; [b]TMSCl added to both cathodic and anodic chambers. [c] Remaining material was over reduced product R-CFH_2.

Cyclic voltammograms of model substrates and defluorinated derivatives

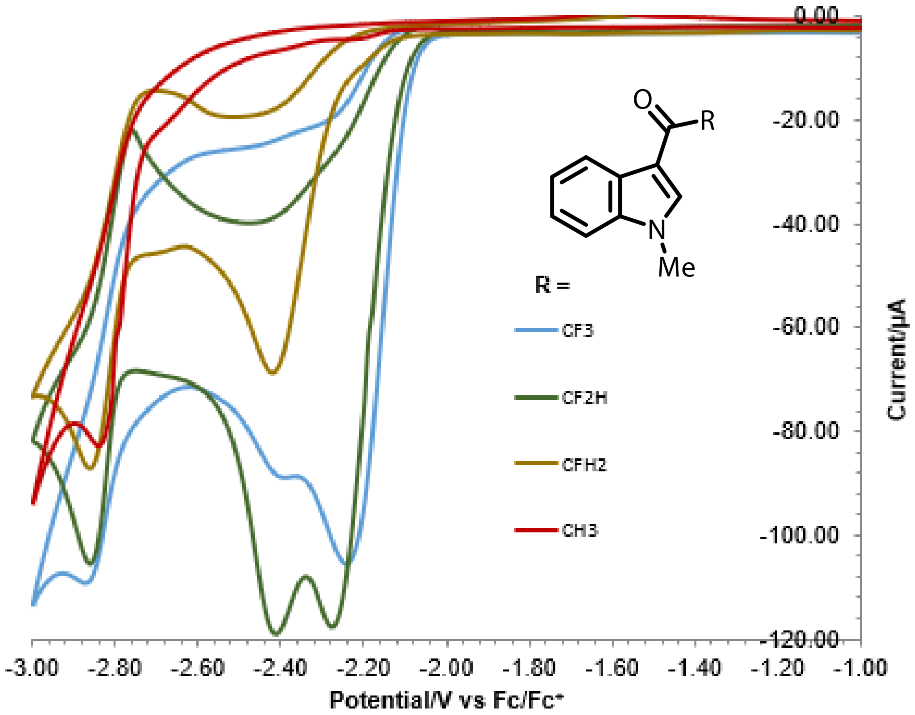

Figure S1. CVs of model substrate **1a, 3a, 4** and 1-(1-methyl-1H-indol-3-yl)ethan-1-one. Conditions: Pt (disk): Pt(disk), 0.1 M $TBAPF_6$, 5 mM substrate, 2.5 mL degassed MeCN under N_2, scan rate = 100 mv/s, $Ag/AgNO_3$ (0.1 M) pseudo reference electrode.

Synthesis and Characterisation of Substrates

General procedure A – for the synthesis of indole-based substrates

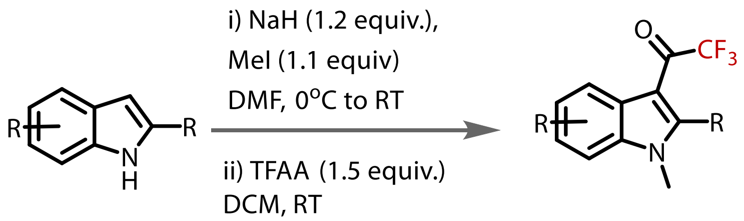

In a 20 ml vial, under air, a suspension of NaH (1.2 equiv., 60% dispersion in mineral oil) in DMF (0.5 M with respect to substrate) was cooled to 0°C using an ice bath. To this, the indole substrate (1.0 equiv.) was added portion wise (with care to avoid violent H_2 evolution) and then stirred for 15 minutes at 0°C. The resulting suspension was warmed to RT over 45 minutes and MeI (1.1 equiv.) added dropwise. The reaction was stirred at RT until complete conversion observed by TLC (generally 20% Et_2O/Hexane). Upon completion (*c.a* 4 h) sat. aq. NH_4Cl was added. The resulting mixture was partitioned, and the organic layer diluted with EtOAc and washed three times with water and then once with brine. The organic layer was dried over $MgSO_4$, filtered and concentrated *in vacuo* (40°C bath, 100 mBar). To the resulting crude material, under air, was added DCM (0.5 M) then trifluoroacetic anhydride (TFAA) (1.5 equiv.) dropwise with stirring (potential exotherm). When the reaction was complete as observed by TLC (generally 20% Et_2O /Hexane), sat. aq. Na_2CO_3 was added and the layers partitioned. The organic layer was diluted with DCM and washed once with water and once with brine. The organic layer was dried with $MgSO_4$, filtered and concentrated *in vacuo* (40°C bath, 100 mBar) to yield analytically pure product.

General procedure B – Pd-catalysed cross-coupling

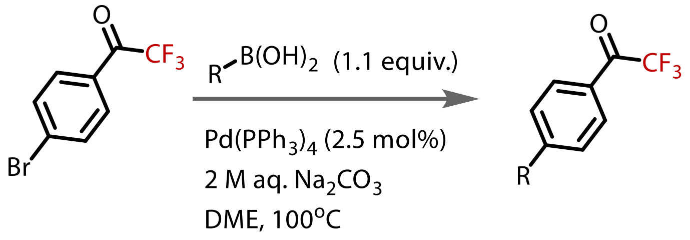

In a 20 ml vial, under air, was added $Pd(PPh_3)_4$ (28.9 mg, 0.075 mmol, 2.5 mol%), boronic acid (3.3 mmol, 1.1 equiv.), and 4'-bromo-2,2,2-trifluoroacetophenone (759 mg, 3 mmol, 1.0 equiv.) sequentially. DME (7 mL) and 2 M aq. Na_2CO_3 (3 mL) were then added and the vial capped and placed in a pre-heated oil bath at 100ºC and the biphasic mixture was stirred overnight. The vial was removed from the heating bath, allowed to cool and filtered through a pad of Celite, rinsing the vial and eluting with $CHCl_3$ (15 mL). The resulting solution was concentrated *in vacuo* (40°C bath, 10 mBar) to yield sufficiently pure product.

General procedure C – lithiation-trifluoroacetylation of aryl bromides

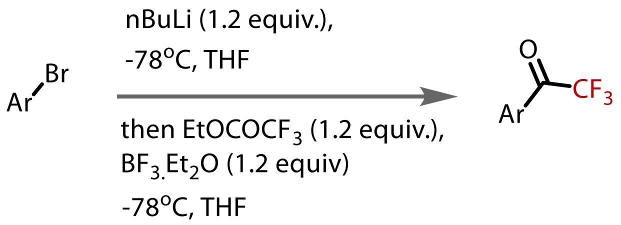

To a flame-dried Schlenk tube under a N_2 atmosphere was added substrate (1.0 equiv.) and THF (0.25 M with respect to substrate). The reaction mixture was cooled to -78 °C and nBuLi (1.2 equiv., 2.5 M solution in hexanes) was added dropwise and the resulting solution stirred at -78 °C for 1 hour. Ethyl trifluoroacetate (1.2 equiv.) and boron trifluoride diethyl etherate (1.2 equiv.) were premixed at RT and added dropwise to the reaction mixture which was then allowed to stir for 2 hours or until complete conversion was observed by TLC control (generally 20% EtOAc in Hexane). The reaction mixture was warmed to RT and quenched with sat. aq. NH_4Cl and partitioned. The organic layer was diluted with EtOAc and washed three times with water and then once with brine. The organic layer was dried over $MgSO_4$, filtered and concentrated *in vacuo* (40 °C bath, 100 mBar) to yield crude product which was purified by flash column chromatography (EtOAc in Hexane).

2,2,2-trifluoro-1-(1-methyl-1H-indol-3-yl)ethan-1-one, **1a**

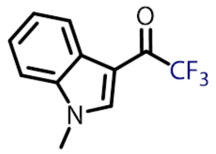

1a was synthesised from indole following **General Procedure A** on a 30 mmol scale to yield a yellow-orange solid (4.65 g, 98%).

R$_f$ = 0.3 (20% Et_2O in Hexane)

^{1}H NMR (400 MHz, $CDCl_3$) δ: 8.61 – 8.63 (m, 1H), 7.94 – 7.85 (m, 1H), 7.42 – 7.36 (m, 3H), 3.92 (s, 3H).

^{13}C NMR (131 MHz, $CDCl_3$) δ: 174.7 (q, *J* = 34.9 Hz), 138.3 (q, *J* = 4.9 Hz), 137.3, 126.9, 124.6, 124.0, 122.6, 117.1 (q, *J* = 291.2 Hz), 110.1, 109.4, 34.0.

^{19}F NMR (376 MHz, $CDCl_3$) δ: -72.3 (s, 3F).

Spectral data in accordance with literature.[1]

1-(4-cyclopropylphenyl)-2,2,2-trifluoroethan-1-one, **1i**

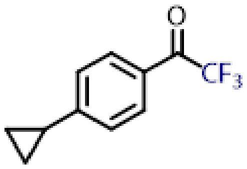

1i was synthesised from cyclopropylboronic acid following **General Procedure B** on a 3 mmol scale, purified using silica gel chromatography eluting with 0% Et_2O in Hexane to 30% Et_2O in Hexane to give a colourless solid (347 mg, 54%).

$\mathbf{R_f}$ = 0.3 (20% Et_2O in Hexane)

^{1}H NMR (400 MHz, $CDCl_3$) δ: 8.02 – 7.93 (m, 2H), 7.24-7.14 (m, 2H), 2.04 (tt, J = 8.4, 5.0 Hz, 1H), 1.23 – 1.12 (m, 2H), 0.84 (dt, J = 6.8, 4.8 Hz, 2H).

^{13}C NMR (131 MHz, $CDCl_3$) δ: 179.9 (q, J = 34.7 Hz), 153.9, 130.4 (q, J = 2.3 Hz), 127.2, 126.0, 116.9 (q, J = 291.5 Hz), 16.2, 11.2.

^{19}F NMR (376 MHz, $CDCl_3$) δ: -71.2 (s, 3F).

Spectral data in accordance with literature.[12]

2,2,2-trifluoro-1-(4-pentylphenyl)ethan-1-one, ***1j***

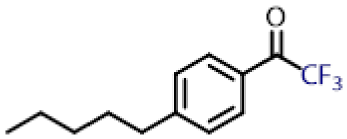

1j was prepared on a 5 mmol scale from 1-bromo-4-pentylbenzene using **General Procedure C** to yield a colourless oil (639 mg, 52%)

R_f = 0.4 (Hexane)

^{1}H NMR (400 MHz, $CDCl_3$) δ 8.00 (d, *J* = 7.6 Hz, 1H), 7.36 (d, *J* = 8.6 Hz, 1H), 2.70 (t, *J* = 7.9 Hz, 1H), 1.66 (m, 1H), 1.34 (m, 2H), 0.90 (t, *J* = 7.0 Hz, 1H).

^{19}F NMR (376 MHz, $CDCl_3$) δ -71.2 (s, 3F).

^{13}C NMR (101 MHz, $CDCl_3$) δ 180.2 (q, *J* = 34.7 Hz), 152.1, 130.4, 129.3, 127.7, 117.0 (q, *J* = 291.5 Hz), 36.3, 31.5, 30.7, 22.6, 14.0.

HRMS (EI$^+$) calc: $[M]^+$ ($C_{13}H_{15}OF_3$) 244.1070; measured: 244.1068 = 0.8 ppm difference

IR (neat) νmax/ cm^{-1}: 2932, 1714, 1607, 1140, 1172, 939, 853, 740.

1-(4-(benzyloxy)-2-methylphenyl)-2,2,2-trifluoroethan-1-one, ***1k***

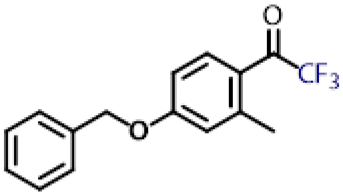

1k was prepared on a 5 mmol scale from 4-(benzyloxy)-1-bromo-2-methylbenzene using **General Procedure C** to yield a white solid (384 mg, 26%)

R_f = 0.3 (1% EtOAc in Hexane)

^{1}H NMR (400 MHz, $CDCl_3$) δ 7.95-7.89 (m, 1H), 7.52 – 7.36 (m, 5H), 7.00 – 6.90 (m, 2H), 5.17 (s, 2H), 2.65 (s, 3H).

^{19}F NMR (376 MHz, $CDCl_3$) δ -70.1 (s, 3F).

^{13}C NMR (101 MHz, $CDCl_3$) δ 180.1 (q, *J* = 33.1 Hz), 163.2, 146.6, 135.9, 134.0 (q, *J* = 4.2 Hz), 128.8, 128.5, 127.6, 121.9, 119.3, 117.0 (q, *J* = 293.0 Hz), 111.9, 70.3, 22.9.

HRMS (ESI$^+$) calc: $[M+Na]^+$ ($C_{16}H_{13}F_3NaO_2$) 317.0760; measured: 317.0773 = 4.3 ppm difference

IR (neat) vmax/ cm^{-1}: 1690, 1309, 1251, 1179, 959, 762.

2,2,2-trifluoro-1-(4-(pyridin-2-yl)phenyl)ethan-1-one, **1l**

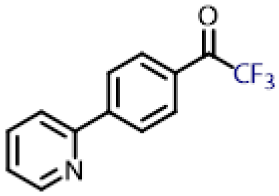

1l was prepared on a 5 mmol scale from 2-(4-bromophenyl)pyridine **General Procedure C** to yield a yellow solid (559 mg, 48%).

R_f = 0.3 (30% EtOAc in Hexane)

^{1}H NMR (400 MHz, $CDCl_3$) δ 8.79 – 8.73 (m, 1H), 8.25 - 8.18 (m, 4H), 7.86 – 7.81 (m, 2H), 7.34 (q, *J* = 4.0 Hz, 1H).

^{19}F NMR (377 MHz, $CDCl_3$) δ -71.3 (s, 3F).

^{13}C NMR (101 MHz, $CDCl_3$) δ 180.4 (q, *J* = 35.1 Hz), 155.3, 150.2, 145.9, 137.2, 130.7, 129.9, 127.5, 123.6, 121.4, 116.8 (q, *J* = 292.1 Hz).

HRMS (EI$^+$) calc: $[M]^+$ ($C_{13}H_8NOF_3$) 251.0553; measured: 251.0550 = 1.2 ppm difference

IR (neat) νmax/ cm^{-1}: 1716, 1605, 1469, 1338, 942, 858, 753.

1-([1,1'-biphenyl]-4-yl)-2,2,2-trifluoroethan-1-one, **1m**

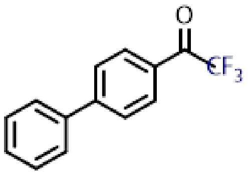

1m was synthesised from phenylboronic acid following **General Procedure B** on a 3 mmol scale to yield a colourless solid (630 mg, 84%).

R_f = 0.3 (30% Et_2O in Hexane)

^{1}H NMR (400 MHz, $CDCl_3$) δ: 8.24 – 8.12 (m, 2H), 7.81 – 7.74 (m, 2H), 7.65-7.58 (m, 2H), 7.59 – 7.43 (m, 3H).

^{13}C NMR (131 MHz, $CDCl_3$) δ: 180.1 (q, *J* = 35.0 Hz), 148.2, 139.1, 130.8 (q, *J* = 2.1 Hz), 129.2, 128.9, 128.6, 127.7, 127.4, 116.8 (q, *J* = 291.4 Hz).

^{19}F NMR (376 MHz, $CDCl_3$) δ: -71.2 (s, 3F).

Spectral data in accordance with literature.[2]

1-(4-(benzo[b]thiophen-3-yl)phenyl)-2,2,2-trifluoroethan-1-one, **1n**

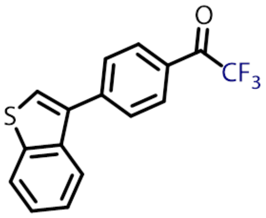

1n was synthesised from 3-benzo[b]thiophenylboronic acid following **General Procedure B** on a 3 mmol scale to yield a brown oil (593 mg, 65%). Note: isolated with 3% of remaining boronic acid, yield corrected to reflect this.

R_f = 0.3 (20% Et_2O in Hexane)

^{1}H NMR (400 MHz, $CDCl_3$) δ: 8.21-8.11 (m, 2H), 7.98 – 7.91 (m, 2H), 7.82 – 7.77 (m, 2H), 7.57 (s, 1H), 7.48 – 7.41 (m, 2H).

^{13}C NMR (131 MHz, $CDCl_3$) δ: 180.0 (q, *J* = 35.0 Hz), 143.3, 140.9, 137.1, 136.3, 130.7 (d, *J* = 2.1 Hz), 129.1, 128.8, 125.8, 125.0, 124.9, 123.2, 122.5, 116.8 (q, *J* = 291.4 Hz).

^{19}F NMR (376 MHz, $CDCl_3$) δ: -71.2 (s, 3F).

HRMS (APCI+) calc: $[M]^+$ ($C_{16}H_9OSF_3$) 306.0321, measured = 306.0318, 1.0 ppm difference.

IR (neat) vmax/ cm^{-1}: 3043, 1715, 1415, 1153, 942.

1-(3'-amino-[1,1'-biphenyl]-4-yl)-2,2,2-trifluoroethan-1-one **1o**

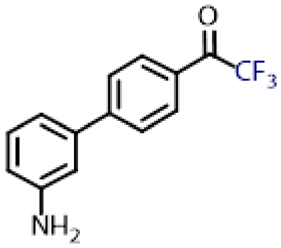

1o was synthesised from 3-aminophenylboronic acid according to **General Procedure B** on a 5 mmol scale and purified using silica gel chromatography (30% Et_2O in pentane to 80% Et_2O in pentane) to yield a yellow oil that was assessed to be 94% pure, the yield has been adjusted to reflect this (895 mg, 67%).

R_f = 0.1 (30% Et_2O in Pentane)

^{1}H NMR (400 MHz, $CDCl_3$) δ: 8.18 – 8.11 (m, 2H), 7.77 – 7.71 (m, 2H), 7.34 – 7.27 (m, 1H), 7.10 – 7.03 (m, 1H), 6.98 – 6.94 (m, 1H), 6.82 – 6.76 (m, 1H), 3.78 (brs, 2H).

^{13}C NMR (131 MHz, $CDCl_3$) δ: 180.2 (q, *J* = 35.0 Hz), 148.5, 147.1, 140.3, 130.7 (q, *J* = 2.2 Hz), 130.1, 128.5, 127.6, 119.3 (d, *J* = 323.3 Hz), 116.6 (q, *J* = 291.3 Hz), 115.6, 113.8

^{19}F NMR (376 MHz, $CDCl_3$) δ: -71.2 (s, 3F)

HRMS (ESI+) calc: [$M+H^+$] ($C_{14}H_{10}ONF_3$) 266.0715, measured = 266.0715, 0 ppm difference.

IR (neat) vmax/ cm^{-1}: 3388, 1712, 1602, 1177, 941, 766.

2,2,2-trifluoro-1-(4-(2-methoxypyrimidin-5-yl)phenyl)ethan-1-one, **1p**

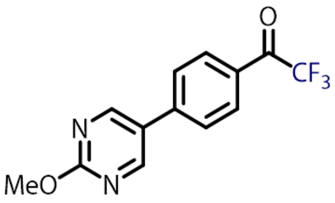

1p was synthesised from (2-methoxypyrimidin-5-yl)boronic acid following **General Procedure B** on a 3 mmol scale and purified using column chromatography on silica gel (0% MeOH in $CHCl_3$ to 10% MeOH in $CHCl_3$) to yield a white solid (431 mg, 51%).

R_f = 0.1 (40% Et_2O in Hexane)

^{1}H NMR (400 MHz, $CDCl_3$) δ: 8.80 (s, 2H), 8.23 (d, *J* = 7.6 Hz, 2H), 7.72 (d, *J* = 8.6 Hz, 2H), 4.09 (s, 3H).

^{13}C NMR (131 MHz, $CDCl_3$) δ: 179.9 (q, *J* = 35.3 Hz), 165.8, 157.6, 157.0, 141.5, 131.2 (q, *J* = 2.1 Hz), 129.3, 126.7 (d, *J* = 56.1 Hz), 116.6 (q, *J* = 291.2 Hz), 55.4.

^{19}F NMR (376 MHz, $CDCl_3$) δ: -71.3 (s, 3F).

HRMS (APCI+) calc: $[M]^+$ ($C_{13}H_9$O2N2F_3) 282.0611, measured = 282.0609, 0.7 ppm difference.

IR (neat) vmax/ cm^{-1}: 3013, 1701, 1402, 1021, 765.

1-(3-(2,6-diphenylpyrimidin-4-yl)phenyl)-2,2,2-trifluoroethan-1-one, **1q**

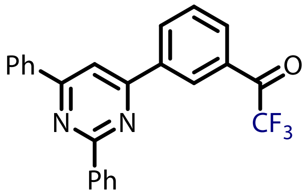

1q was prepared on a 5 mmol scale from 4-(3-bromophenyl)-2,6-diphenylpyrimidine using **General Procedure C** to yield a yellow solid (325 mg, 16%)

R_f = 0.3 (20% EtOAc in Hexane)

^{1}H NMR (400 MHz, $CDCl_3$) δ 8.97 (s, 1H), 8.75 – 8.71 (m, 2H), 8.68 – 8.65 (m, 1H), 8.34 – 8.29 (m, 2H), 8.25 – 8.21 (m, 1H), 8.06 (s, 1H), 7.80 – 7.73 (m, 1H), 7.62 – 7.54 (m, 6H).

^{13}C NMR (101 MHz, $CDCl_3$) δ 180.5 (q, *J* = 34.8 Hz), 165.4, 164.8, 162.9, 138.9, 137.8, 137.2, 134.2, 132.0, 131.3, 131.1, 130.7, 129.9, 129.5 – 128.3 (m), 127.5, 116.8 (q, *J* = 290.9 Hz), 110.3.

^{19}F NMR (377 MHz, $CDCl_3$) δ -71.2 (s, 3F).

HRMS (ACPI$^+$) Calc: $[M+H]^+$ ($C_{24}H_{15}F_3N_2O$) 405.1209; measured: 405.1195 = 3.5 ppm difference

IR (neat) νmax/ cm^{-1}: 2927, 1717, 1590, 1568, 1531, 860, 740.

1-(4-(benzo[b]thiophen-3-yl)phenyl)-2,2,2-trifluoroethan-1-one, **1r**

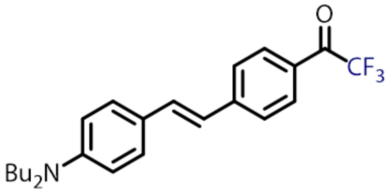

1r was synthesised following literature precedent[3] from 4-(dibutylamino)-benzaldehyde to yield an orange oil that solidified on standing (1.40 g, 51% over 2 steps).

R_f = 0.7 (20% Et_2O in Hexane)

^{1}H NMR (400 MHz, $CDCl_3$) δ: 8.31 – 7.92 (m, 2H), 7.71 – 7.52 (m, 2H), 7.44 (d, *J* = 8.8 Hz, 2H), 7.23 (d, *J* = 16.2 Hz, 1H), 6.93 (d, *J* = 16.1 Hz, 1H), 6.61 (d, *J* = 8.9 Hz, 2H), 4.19 – 3.1 (m, 4H), 2.11 – 1.52 (m, 4H), 1.42 (hept, *J* = 8.4, 7.9 Hz, 4H), 1.04 (t, *J* = 7.3 Hz, 6H).

^{13}C NMR (131 MHz, $CDCl_3$) δ: 179.5 (q, *J* = 34.6 Hz), 148.7, 145.9, 133.9, 130.7 (d, *J* = 2.1 Hz), 128.7, 127.3, 126.0, 123.3, 121.3, 116.9 (q, *J* = 291.5 Hz), 111.5, 50.8, 29.5, 20.3, 14.0.

^{19}F NMR (376 MHz, $CDCl_3$) δ: -70.9 (s, 3F).

Spectral data in accordance with literature.[3]

(E)-2,2,2-trifluoro-1-(4-styrylphenyl)ethan-1-one, **1s**

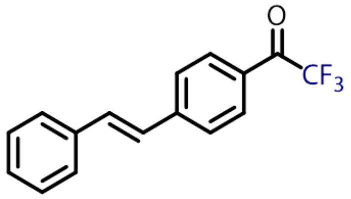

1s was synthesised from styrylboronic acid following **General Procedure B** on a 3 mmol scale to yield a brown solid (572 mg, 69%).

R_f = 0.3 (20% Et_2O in Hexane)

^{1}H NMR (400 MHz, $CDCl_3$) δ: 8.10 – 8.04 (m, 2H), 7.70 – 7.63 (m, 2H), 7.60 – 7.54 (m, 2H), 7.44 – 7.38 (m, 2H), 7.37 – 7.27 (m, 2H), 7.15 (d, *J* = 16.3 Hz, 1H).

^{13}C NMR (131 MHz, $CDCl_3$) δ: 179.8 (q, *J* = 34.9 Hz), 144.5, 136.3, 133.3, 130.7 (q, *J* = 1.9 Hz), 128.9, 128.8, 127.1, 126.9, 126.8, 126.4, 116.8 (q, *J* = 291.4 Hz).

^{19}F NMR (376 MHz, $CDCl_3$) δ: -71.1 (s, 3F).

HRMS (APCI+) calc: $[M]^+$ ($C_{16}H_{11}OF_3$) 276.0757, measured = 276.0756, 0.4 ppm difference.

IR (neat) vmax/ cm^{-1}: 2938, 1768, 1208, 1048, 691.

2,2,2-trifluoro-1-(naphthalen-2-yl)ethan-1-one, **1u**

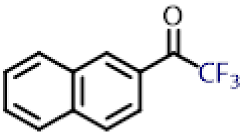

1u was prepared on a 3.0 mmol scale from 2-bromonaphthalene using **General Procedure C** to yield a white solid (499 mg, 74%)

R_f = 0.4 (10% EtOAc in Hexane)

^{1}H NMR (400 MHz, $CDCl_3$) δ 8.63 (s, 1H), 8.08 (d, *J* = 8.5 Hz, 1H), 8.02 (d, *J* = 8.2 Hz, 1H), 7.97 (d, *J* = 8.8 Hz, 1H), 7.92 (d, *J* = 8.2 Hz, 1H), 7.74 – 7.59 (m, 2H).

^{13}C NMR (101 MHz, $CDCl_3$) δ 180.6 (q, *J* = 35.0 Hz), 136.6, 134.0 – 133.0 (m), 132.3, 130.3, 130.2, 129.2, 128.0, 127.6, 127.3, 124.3, 117.0 (q, *J* = 291.3 Hz).

^{19}F NMR (376 MHz, $CDCl_3$) δ -70.6 (s, 3F).

Spectral data in accordance with literature.[4]

tert-butyl 3-(2,2,2-trifluoroacetyl)-1H-indole-1-carboxylate, **1w**

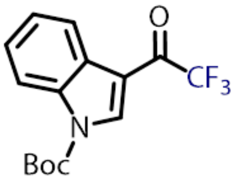

In a 20 ml vial, under air, to a solution of 3-trifluoroacetyl-1H-indole (1.07 g, 5 mmol, 1 equiv.) in DCM (10 ml, 0.5 M) was added Et_3N (759 mg, 7.5 mmol, 1.5 equiv.) and then Boc_2O (1.09 g, 5 mmol, 1 equiv.) with stirring. A white precipitate formed and the suspension was stirred overnight. Sat. aq. NH_4Cl and DCM was added and the layers partitioned. The organic layer was washed twice with water and once with brine. The organic layer was dried with $MgSO_4$, filtered and concentrated *in vacuo* (40°C bath, 100 mBar) to yield the title material as a white solid (1.31 g, 84%).

R_f = 0.3 (30% Et_2O in Hexane)

^{1}H NMR (400 MHz, $CDCl_3$) δ: 8.44 (d, *J* = 1.8 Hz, 1H), 8.36 (dd, *J* = 7.1, 2.0 Hz, 1H), 8.25 – 8.12 (m, 1H), 7.43 (m, 2H), 1.73 (s, 9H).

^{13}C NMR (131 MHz, $CDCl_3$) δ: 176.2 (q, *J* = 36.0 Hz), 148.5, 135.4 (d, *J* = 5.2 Hz), 135.3, 127.2, 126.5, 125.2, 122.4, 116.5 (q, *J* = 290.8 Hz), 115.3, 113.0, 86.6, 28.0

^{19}F NMR (376 MHz, $CDCl_3$) δ: -78.2 (s, 3F).

Spectral data in accordance with literature.[5]

2,2,2-trifluoro-1-(1-(perfluoropyridin-4-yl)-1H-indol-3-yl)ethan-1-one, **1x**

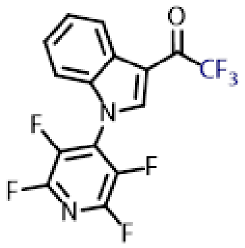

In a 20 ml vial, under air, to a solution of 3-trifluoroacetyl-1H-indole (1.07 g, 5 mmol, 1 equiv.) in DCM (10 ml, 0.5 M) was added Et_3N (759 mg, 7.5 mmol, 1.5 equiv.) and then perfluoropyridine (845 mg, 5 mmol, 1 equiv.) with stirring. A red precipitate formed and the suspension was stirred overnight. Sat. aq. NH_4Cl and DCM was added and the layers partitioned. The organic layer was washed twice with water and once with brine. The organic layer was dried with $MgSO_4$, filtered and concentrated *in vacuo* (40°C bath, 100 mBar) to yield the title material as a red solid (1.45 g, 80%).

R_f = 0.2 (30% Et_2O in Hexane)

^{1}H NMR (400 MHz, $CDCl_3$) δ: 8.56 – 8.47 (m, 1H), 8.11 - 8.04 (m, 1H), 7.60 – 7.48 (m, 2H), 7.28 - 7.19 (m, 1H).

^{13}C NMR (131 MHz, $CDCl_3$) δ: 175.6 (q, *J* = 36.2 Hz), 145.5 – 143.1 (m), 138.8 – 136.3 (m), 136.1 (d, *J* = 5.3 Hz), 135.9, 128.1 (d, *J* = 12.1 Hz), 126.4, 126.2, 125.4, 123.1, 116.5 (d, *J* = 290.7 Hz), 114.0, 111.0.

^{19}F NMR (376 MHz, $CDCl_3$) δ: -72.8 (s, 3F), -85.0 – -85.5 (m, 2F), -144.7 – -145.0 (m, 2F).

HRMS (ESI$^+$) calc: [M+Na$^+$] ($C_{25}H_5F_7N_2NaO$) 385.0182, measured = 385.0178, -1.0 ppm difference.

IR (neat) vmax/ cm^{-1}: 2967, 1658, 1537, 1183, 748.

1-(1-benzyl-1H-indol-3-yl)-2,2,2-trifluoroethan-1-one, **1y**

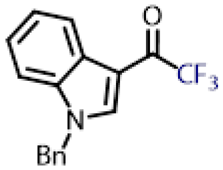

In a 20 ml vial, under air, to a solution of 3-trifluoroacetyl-1H-indole (1.07 g, 5 mmol, 1 equiv.) in DCM (10 ml, 0.5 M) was added Et_3N (759 mg, 7.5 mmol, 1.5 equiv.) and then BnBr (855 mg, 5 mmol, 1 equiv.) with stirring. A white precipitate formed and the suspension was stirred overnight. Sat. aq. NH_4Cl and DCM was added and the layers partitioned. The organic layer was washed twice with water and once with brine. The organic layer was dried with $MgSO_4$, filtered and concentrated in vacuo (40°C bath, 100 mBar) to yield the title material as a white solid (1.24 g, 4.1 mmol, 82%).

R_f = 0.3 (30% Et_2O in Hexane)

^{1}H NMR (400 MHz, $CDCl_3$) δ: 8.45 (d, *J* = 7.7 Hz, 1H), 8.00 (s, 1H), 7.36 (m, 6H), 7.19 (d, *J* = 6.9 Hz, 2H), 5.40 (s, 2H).

^{13}C NMR (131 MHz, $CDCl_3$) δ: 174.9 (q, *J* = 34.9 Hz), 137.7 (q, *J* = 5.0 Hz), 136.9, 134.8, 129.2, 128.6, 127.2, 127.0, 124.8, 124.1, 122.7, 117.1 (q, *J* = 291.1 Hz), 110.8, 109.9, 51.4

^{19}F NMR (376 MHz, $CDCl_3$) δ: -72.1 (s, 3F).

Spectral data in accordance with literature.[6]

2,2,2-trifluoro-1-(6-methoxy-1-methyl-1H-indol-3-yl)ethan-1-one, **1aa**

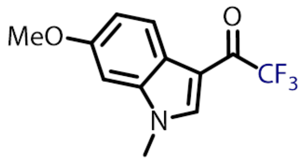

1aa was synthesised from 6-methoxyindole following **General Procedure A** on a 5 mmol scale to yield a colourless solid (1.17 g, 91%).

R_f = 0.2 (20% Et_2O in Hexane)

^{1}H NMR (400 MHz, $CDCl_3$) δ: 7.98 (d, *J* = 8.0 Hz, 1H), 7.75 - 7.69 (m, 1H), 7.36 – 7.14 (m, 1H), 6.78 (d, *J* = 7.9 Hz, 1H), 4.14 (s, 3H), 3.95 (s, 3H).

^{13}C NMR (131 MHz, $CDCl_3$) δ: 174.7 (q, *J* = 34.4 Hz), 147.9, 138.9 (d, *J* = 5.0 Hz), 129.5, 126.9, 124.9, 117.2 (d, *J* = 291.1 Hz), 114.9, 109.3, 105.5, 55.6, 38.3.

^{19}F NMR (376 MHz, $CDCl_3$) δ: -72.1 (s, 3F).

Spectral data in accordance with literature.[6]

1-methyl-3-(2,2,2-trifluoroacetyl)-1H-indole-5-carbonitrile, **1ab**

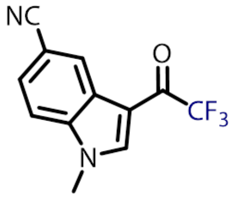

1ab was synthesised from 5-cyanoindole following **General Procedure A** on a 5 mmol scale to yield a brown solid (958 mg, 76%).

R_f = 0.3 (20% Et_2O in Hexane)

^{1}H NMR (400 MHz, $CDCl_3$) δ: 8.75 (dd, *J* = 1.6, 0.7 Hz, 1H), 8.03 (d, *J* = 1.7 Hz, 1H), 7.64 (dd, *J* = 8.6, 1.6 Hz, 1H), 7.50 (dd, *J* = 8.5, 0.7 Hz, 1H), 3.98 (s, 3H).

^{13}C NMR (131 MHz, $CDCl_3$) δ: 174.8 (q, *J* = 36.1 Hz), 139.8 (q, *J* = 4.8 Hz), 138.8, 127.9, 127.7, 126.7, 119.4, 116.7 (q, *J* = 290.7 Hz), 111.2, 109.8, 107.5, 34.3.

^{19}F NMR (376 MHz, $CDCl_3$) δ: -72.7 (s, 3F).

HRMS (APCI+) calc: $[M+H]^+$ ($C_{12}H_7N_2OF_3$) 253.0583, measured = 253.0577, 2.4 ppm difference.

IR (neat) νmax/ cm^{-1}: 3111, 1686, 1253, 754, 575.

1-(4-fluoro-1-methyl-1H-indol-3-yl)-2,2,2-trifluoroethan-1-one, **1ac**

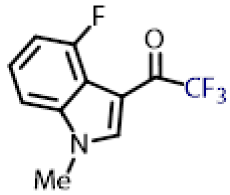

1ac was synthesised from 4-fluoroindole following **General Procedure A** on a 5 mmol scale to yield a colourless solid (1.11 g, 91%).

R_f = 0.3 (20% Et_2O in Hexane)

^{1}H NMR (400 MHz, $CDCl_3$) δ: 7.86 (s, 1H), 7.27 (td, J = 8.0, 4.4 Hz, 1H), 7.13 (d, J = 8.2 Hz, 1H), 6.96 (dd, J = 10.7, 7.9 Hz, 1H), 3.87 (s, 3H).

^{13}C NMR (131 MHz, $CDCl_3$) δ: 172.9 (q, J = 34.7 Hz), 156.6 (d, J = 255.0 Hz), 140.2 (d, J = 10.5 Hz), 139.2 (d, J = 5.2 Hz), 125.6 (d, J = 7.8 Hz), 117.4 (q, J = 291.9 Hz), 114.5 (d, J = 21.3 Hz), 109.8 (d, J = 21.4 Hz), 108.8 (d, J = 5.8 Hz), 106.5 (d, J = 4.2 Hz), 34.5.

^{19}F NMR (376 MHz, $CDCl_3$) δ: -71.2 (s, 3F), -107.7 (dd, J = 10.8, 4.5 Hz, 2F).

Spectral data in accordance with literature.[7]

1-(4-chloro-1-methyl-1H-indol-3-yl)-2,2,2-trifluoroethan-1-one, **1ad**

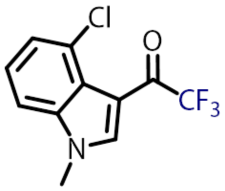

1ac was synthesised from 4-chloroindole following **General Procedure A** on a 5 mmol scale to yield a brown solid (1.16 g, 89%).

R_f = 0.3 (20% Et_2O in Hexane)

^{1}H NMR (400 MHz, $CDCl_3$) δ: 7.91 (q, *J* = 1.8 Hz, 1H), 7.45 – 7.26 (m, 3H), 3.89 (s, 3H).

^{13}C NMR (131 MHz, $CDCl_3$) δ: 172.9 (q, *J* = 34.4 Hz), 139.4 (q, *J* = 5.3 Hz), 139.2, 127.9, 125.5, 125.1, 124.3, 117.3 (q, *J* = 292.5 Hz), 109.5, 108.8, 34.3.

^{19}F NMR (376 MHz, $CDCl_3$) δ: -71.0 (s, 3F).

HRMS (ESI^+) calc: [$M+Na^+$] ($C_{11}H_7{}^{35}ClF_3NNaO$) 284.0060 measured 284.0064, 0.8 ppm difference.

IR (neat) vmax/ cm^{-1}: 2924, 1682, 1527, 1079, 721.

1-(4-bromo-1-methyl-1H-indol-3-yl)-2,2,2-trifluoroethan-1-one, **1ae**

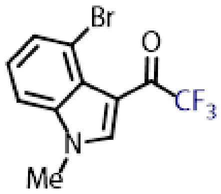

1ae was prepared on a 4 mmol scale from 4-bromoindole using **General Procedure A** to yield a brown solid (989 mg, 81%)

^{1}H NMR (400 MHz, $CDCl_3$) δ 7.89 – 7.85 (m, 1H), 7.51 (d, *J* = 7.7 Hz, 1H), 7.30 (d, *J* = 8.2 Hz, 1H), 7.16 (app. t, *J* = 8.0 Hz, 1H), 3.87 (s, 3H).

^{13}C NMR (101 MHz, $CDCl_3$) δ 173.0 (q, *J* = 34.3 Hz), 139.5 (q, *J* = 5.3 Hz), 139.2, 129.2, 126.0, 125.3, 117.4 (q, *J* = 291.2 Hz), 114.9, 109.5, 109.4, 34.4.

^{19}F NMR (376 MHz, $CDCl_3$) δ -71.1 (s, 3F).

HRMS (ESI$^+$) calc: $[M+Na]^+$ ($C_{11}H_7{}^{79}BrF_3NNaO$) 327.9555; measured: 327.9563 = 2.4 ppm difference

IR (neat) νmax/ cm^{-1}: 2955, 1676, 1526, 1079, 720.

1-(5-bromo-1-methyl-1H-indol-3-yl)-2,2,2-trifluoroethan-1-one, **1af**

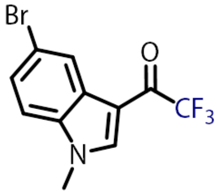

1af was synthesised from 5-bromoindole following **General Procedure A** on a 5 mmol scale to yield an orange solid (1.20 g, 80%).

R$_f$ = 0.3 (20% Et_2O in Hexane)

^{1}H NMR (400 MHz, $CDCl_3$) δ: 8.62 – 8.50 (m, 1H), 8.06 – 7.82 (m, 1H), 7.56 – 7.43 (m, 1H), 7.34 – 7.21 (m, 1H), 3.90 (s, 3H).

^{13}C NMR (131 MHz, $CDCl_3$) δ: 174.6 (q, *J* = 35.2 Hz), 138.4 (q, *J* = 4.9 Hz), 136.0, 128.4, 127.7, 125.2, 119.2 (q, *J* = 290.9 Hz), 117.8, 111.6, 108.9, 34.2.

^{19}F NMR (376 MHz, $CDCl_3$) δ: -72.4 (s, 3F).

Spectral data in accordance with literature.[6]

2,2,2-trifluoro-1-(5-iodo-1-methyl-1H-indol-3-yl)ethan-1-one, **1ag**

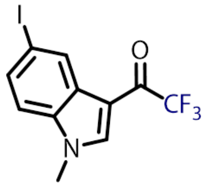

1ag was synthesised from 5-iodoindole following **General Procedure A** on a 5 mmol scale to yield a brown solid (1.52 g, 86%).

R_f = 0.3 (20% Et_2O in Hexane)

^{1}H NMR (400 MHz, $CDCl_3$) δ: 8.74 (s, 1H), 7.83 (s, 1H), 7.64 (d, *J* = 8.5 Hz, 1H), 7.15 (d, *J* = 8.6 Hz, 1H), 3.89 (s, 3H).

^{13}C NMR (131 MHz, $CDCl_3$) δ: 174.6 (q, *J* = 35.2 Hz), 138.4 (q, *J* = 4.9 Hz), 136.5, 133.3, 131.4, 128.9, 116.9 (q, *J* = 290.9 Hz), 111.9, 108.7, 88.5, 34.2

^{19}F NMR (376 MHz, $CDCl_3$) δ: -72.4 (s, 3F).

HRMS (ESI+) calc: $[M+Na]^+$ ($C_{11}H_7F_3INNaO$) 375.9417, measured 375.9414, 0.6 ppm difference.

IR (neat) vmax/ cm^{-1}: 3119, 2924, 1656, 1527, 883, 722.

2,2,2-trifluoro-1-(1-methyl-2-(thiophen-2-yl)-1H-indol-3-yl)ethan-1-one, **1ah**

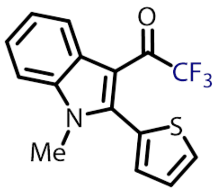

1ah was synthesised from 2-thiophenylindole following **General Procedure A** on a 5 mmol scale to yield a yellow solid (1.39 g, 90%).

R_f = 0.3 (10% Et_2O in Hexane)

^{1}H NMR (400 MHz, $CDCl_3$) δ: 8.31 – 8.24 (m, 1H), 7.63-7.55 (m, 1H), 7.47 – 7.38 (m, 3H), 7.24 – 7.19 (m, 2H), 3.64 (s, 3H).

^{13}C NMR (131 MHz, $CDCl_3$) δ: 176.1 (q, J = 36.5 Hz), 141.4, 137.2, 131.7, 129.4, 129.3, 127.2, 126.2, 124.5, 123.9, 121.9 (q, J = 2.2 Hz), 116.5 (q, J = 289.9 Hz), 110.9, 110.3, 31.3.

^{19}F NMR (376 MHz, $CDCl_3$) δ: -72.9 (s, 3F).

HRMS (ESI+) calc: $[M+H]^+$ ($C_{15}H_{10}NOSF_3$) 310.0508, measured 310.0505, 1.0 ppm difference.

IR (neat) vmax/ cm^{-1}: 3098, 1591, 1482, 1311, 742.

2,2,2-trifluoro-1-(1-methyl-2-phenyl-1H-indol-3-yl)ethan-1-one, **1ai**

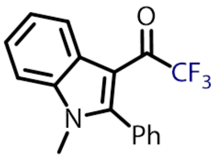

1ai was synthesised from 2-phenylindole following **General Procedure A** on a 5 mmol scale to yield a colourless solid (1.24 g, 82%).

R_f = 0.4 (30% Et_2O in Hexane)

^{1}H NMR (400 MHz, $CDCl_3$) δ: 8.38 – 8.29 (m, 1H), 7.59 – 7.50 (m, 3H), 7.45 – 7.37 (m, 5H), 3.54 (s, 3H).

^{13}C NMR (131 MHz, $CDCl_3$) δ: 175.97 (q, *J* = 36.3 Hz), 149.2, 136.5, 130.6, 130.2, 129.8, 128.2, 126.5, 124.2, 123.9, 122.5, 116.5 (q, *J* = 289.9 Hz), 110.3, 108.9, 31.3.

^{19}F NMR (376 MHz, $CDCl_3$) δ: -72.6 (s, 3F).

Spectral data in accordance with literature.[8]

2,2,2-trifluoro-1-(1-methyl-1H-indol-5-yl)ethan-1-one, **1aj**

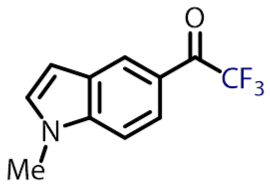

1aj was synthesised from 5-bromo-1-methyl indole (as prepared from 5-bromoindole using **General Procedure A**) following **General Procedure C** on a 8.6 mmol scale and purified using column chromatography on silica gel (23% Et_2O in Hexane to 43% Et_2O in Hexane) to yield an orange solid (804 mg, 41%).

R_f = 0.3 (30% Et_2O in Hexane)

^{1}H NMR (400 MHz, $CDCl_3$) δ: 8.43 (s, 1H), 8.04 (dd, *J* = 8.9, 1.7 Hz, 1H), 7.53 – 7.24 (m, 1H), 7.21 (d, *J* = 3.2 Hz, 1H), 6.84 – 6.53 (m, 1H), 3.81 (s, 3H).

^{13}C NMR (131 MHz, $CDCl_3$) δ: 180.5 (q, *J* = 34.2 Hz), 140.3, 131.4 (d, *J* = 19.3 Hz), 128.2, 126.0, 123.4, 121.8, 117.4 (d, *J* = 291.9 Hz), 109.9 (q, *J* = 15.2 Hz), 104.0, 33.2.

^{19}F NMR (376 MHz, $CDCl_3$) δ: -69.9 (s, 3F).

Spectral data in accordance with literature.[9]

2,2,2-trifluoro-1-(9-methyl-9H-carbazol-3-yl)ethan-1-one, **1ak**

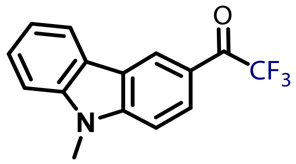

1ak was synthesised from carbazole according to literature precedent to yield a yellow oil that solidified overnight to a green-yellow solid (452 mg, 83%).

R$_f$ = 0.4 (30% Et_2O in Pentane)

^{1}H NMR (400 MHz, $CDCl_3$) δ: 8.77 (s, 1H), 8.22 – 8.14 (m, 1H), 8.11 (d, *J* = 7.8 Hz, 1H), 7.55 (ddd, *J* = 8.3, 7.1, 1.2 Hz, 1H), 7.43 – 7.29 (m, 3H), 3.82 (s, 3H).

^{13}C NMR (131 MHz, $CDCl_3$) δ: 179.8 (q, *J* = 34.0 Hz), 144.8, 141.8, 128.1 (q, *J* = 2.2 Hz), 127.2, 124.0 (q, *J* = 2.8 Hz), 123.0, 122.9, 121.1, 120.9, 120.8, 117.5 (q, *J* = 291.6 Hz), 109.4, 108.8, 29.4.

^{19}F NMR (376 MHz, $CDCl_3$) δ: -69.8 (s, 3F)

Spectral data in accordance with literature.[9]

2,2,2-trifluoro-1-(1-methyl-1H-pyrazol-4-yl)ethan-1-one, **1an**

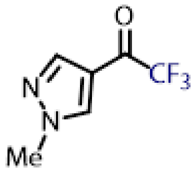

To a 50 mL round-bottomed flask equipped was added 1-methyl-1*H*-pyrazole (821 mg, 10 mmol, 1.0 equiv.) and pyridine (5 mL). The reaction mixture was cooled to 0 °C in an ice bath and TFAA (2.8 mL, 20 mmol, 2.0 equiv.) was added dropwise. The reaction was stirred at reflux for 24 hours before being quenched with water. The reaction mixture was partitioned and extracted into DCM (3x 20 mL). The organic layers were combined, dried over $MgSO_4$, filtered and concentrated in *vacuo* to afford **1an** as a brown oil (942 mg, 53%).

R_f = 0.3 (20% EtOAc in Hexane)

^{1}H NMR (400 MHz, $CDCl_3$) δ 8.06 (d, *J* = 6.9 Hz, 2H), 4.00 (s, 3H).

^{13}C NMR (101 MHz, $CDCl_3$) δ 174.4 (q, *J* = 36.7 Hz), 141.9, 135.1, 116.4 (p, *J* = 290.7 Hz), 39.5.

^{19}F NMR (376 MHz, $CDCl_3$) δ -74.9 (s, 3F).

Spectral data in accordance with literature.[10]

2,2,2-trifluoro-1-(1-(pentan-3-yl)-1H-pyrazol-4-yl)ethan-1-one, **1ao**

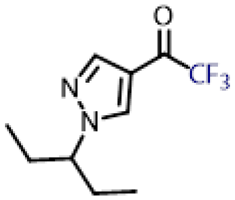

1ao was prepared according to literature precedent[11] and purified by flash column chromatography (20% EtOAc in Hexane) to yield a yellow oil (398 mg, 34%)

R_f = 0.4 (20% EtOAc in Hexane)

^{1}H NMR (400 MHz, $CDCl_3$) δ 8.10 (s, 1H), 8.04 (s, 1H), 4.02 – 3.92 (m, 1H), 2.02 – 1.79 (m, 4H), 0.79 (t, J = 7.4 Hz, 6H).

^{19}F NMR (376 MHz, $CDCl_3$) δ -74.7 (s, 3F).

^{13}C NMR (101 MHz, $CDCl_3$) δ 174.8 (q, J = 36.6 Hz), 141.9, 133.9, 116.6 (q, J = 290.2 Hz), 115.7, 68.0, 28.0, 10.6.

Spectral data in accordance with literature.[12]

(Z)-1,1,1-trifluorononadec-10-en-2-one, **1ap**

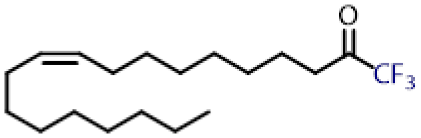

To a 7 mL vial, under air, was added methyl oleate (1.01 mL, 3 mmol, 1 equiv.) and $TESCF_3$ (2.07 mL, 9 mmol, 3 equiv.). With stirring, TBAT (162 mg, 0.3 mmol, 10 mol%) was added and the vial capped quickly (caution: exotherm). After stirring for 1 h (or until complete consumption of the starting material, determined by TLC) was added TBAF (3.2 mL of a 1 M THF solution, 3.2 mmol, 1.06 equiv). The resulting solution was stirred for 5 minutes before being concentrated directly onto silica. The crude material was purified in 3 portions through silica gel chromatography (7% Et_2O in Hexane to 17% Et_2O in Hexane) to yield the title material (440 mg, 41%) as a colourless oil. *Note: This product was isolated alongside c.a 5% TBAT which proved difficult to remove – the yield has been adjusted accordingly.*

R_f = 0.4 (20% Et_2O in Hexane)

^{1}H NMR (400 MHz, $CDCl_3$) δ: 5.53 – 5.31 (m, 2H), 2.72 (t, *J* = 7.2 Hz, 2H), 2.01 (tt, *J* = 6.5, 3.4 Hz, 4H), 1.74 (q, *J* = 7.1 Hz, 2H), 1.54 – 1.25 (m, 20H), 0.91 – 0.95 (m, 3H).

^{13}C NMR (131 MHz, $CDCl_3$) δ: 191.7 (q, *J* = 34.6 Hz), 130.2, 129.7, 115.7 (q, *J* = 292.3 Hz), 36.4, 32.0, 31.7, 29.9, 29.7, 29.6, 29.4, 29.2, 29.1, 28.8, 27.3, 27.2, 22.8, 22.4, 14.2.

^{19}F NMR (376 MHz, $CDCl_3$) δ: -79.2 (s, 3F).

Spectral data in accordance with literature.[13]

N-4-Fluorophenyl trifluoroacetamide, **1as**

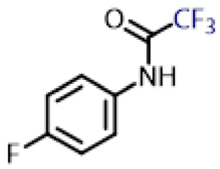

To a solution of 4-fluoroaniline (556 mg, 5 mmol, 1 equiv.) in DCM (0.7 M) at 0ºC was added TFAA (1.0 ml, 10 mmol, 2 equiv.) with stirring. After addition was complete, the reaction was warmed to RT and monitored by TLC (20% EA/hexane) until complete consumption of the SM was observed. One complete, saturated aq. Na_2CO_3 was added and the layers partitioned. The organic layer was washed once with saturated aq. Na_2CO_3, once with water and once with brine. The organic layer was dried over $MgSO_4$ and concentrated *in vacuo* to yield analytically pure title material (856 mg, 4.6 mmol, 83%) as an off white solid.

R_f = 0.3 (20% EtOAc in Hexane)

^{1}H NMR (400 MHz, $CDCl_3$) δ: 7.92 (br. s, 1H), 7.54 (dd, *J* = 4.6, 4.3 Hz, 2H), 7.09 (dd, *J* = 4.6, 1.1 Hz, 2H).

^{19}F NMR (376 MHz, $CDCl_3$) δ: -75.5 (s, 3F), -114.6 - -114.8 (m, 1F).

^{13}C NMR (131 MHz, $CDCl_3$) δ: 161.8 (q, *J* = 28.2 Hz), 159.4 (d, *J* = 245.4 Hz), 131.0 (d, *J* = 4.1 Hz), 122.6 (d, *J* = 8.1 Hz), 116.3 (d, *J* = 22.5 Hz), 115.8 (q, *J* = 286.3 Hz).

Spectral data in accordance with literature.[14]

(E)-2,2,2-trifluoro-N-phenethyl-1-phenylethan-1-imine, **1au**

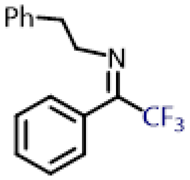

To a stirred solution of phenylethylamine (0.62 mL, 5 mmol, 1 equiv.) in $CHCl_3$ (5 mL) was added glacial acetic acid (0.29 mL, 5 mmol, 1 equiv.). 2,2,2-Trifluoroacetophenone (0.70 mL, 5 mmol, 1 equiv.) was added as a solution in $CHCl_3$ (1 mL) and the solution stirred at reflux overnight. Once cooled, sat. aq. Na_2CO_3 was added and the layers separated. The aqueous layer was extracted with once with $CHCl_3$ and the combined organic extracts dried with $MgSO_4$ and concentrated *in vacuo* (100 mBar, 40°C bath). The crude material was purified by chromatography on silica gel (7% Et_2O in Pentane to 30% Et_2O in pentane) to yield **1au** as a colourless oil (795 mg, 57%).

R_f = 0.3 (20% Et_2O in Pentane)

^{1}H NMR (400 MHz, $CDCl_3$) δ 7.53 – 7.34 (m, 3H), 7.32 – 7.17 (m, 3H), 7.15 – 7.04 (m, 2H), 6.94 – 6.80 (m, 2H), 3.66 (t, *J* = 7.1, 2H), 3.02 (t, *J* = 7.1 Hz, 2H).

^{19}F NMR (376 MHz, $CDCl_3$) δ -71.1 (s, 3F).

^{13}C NMR (101 MHz, $CDCl_3$) δ 158.9 (q, *J* = 33.6 Hz), 139.1, 130.2, 129.9, 129.1, 128.6, 128.4, 127.5, 126.4, 119.7 (q, *J* = 278.6 Hz), 54.8, 36.5.

HRMS (ESI$^+$) calc: $[M+Na]^+$ ($C_{16}H_{14}F_3NNa$) 300.0971, measured 300.0966, 2 ppm difference.

IR (neat) νmax/ cm^{-1}: 2838, 1667, 1512, 1329, 1035, 700.

(E)-2,2,2-trifluoro-N-(4-methoxybenzyl)-1-phenylethan-1-imine, ***1av***

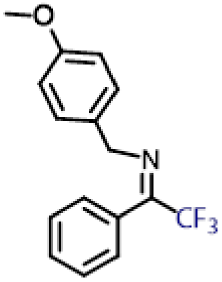

To a stirred solution of 4-methoxybenzylamine (0.65 mL, 5 mmol, 1 equiv.) in $CHCl_3$ (5 mL) was added glacial acetic acid (0.29 mL, 5 mmol, 1 equiv.). 2,2,2-Trifluoroacetophenone (0.70 mL, 5 mmol, 1 equiv.) was added as a solution in $CHCl_3$ (1 mL) and the solution stirred at reflux overnight. Once cooled, sat. aq. Na_2CO_3 was added and the layers separated. The aqueous layer was extracted with once with $CHCl_3$ and the combined organic extracts dried with $MgSO_4$ and concentrated *in vacuo* (100 mBar, 40°C bath). The crude material was purified by chromatography on silica gel (10% Et_2O in Pentane to 30% Et_2O in pentane) to yield **1av** as a colourless oil (965 mg, 66%).

R_f = 0.3 (20% Et_2O in Pentane)

^{1}H NMR (400 MHz, $CDCl_3$) δ 7.59 – 7.46 (m, 3H), 7.32 – 7.27 (m, 2H), 7.21 – 7.10 (m, 2H), 6.95 – 6.72 (m, 2H), 4.56 (d, *J* = 1.9 Hz, 2H), 3.80 (s, 3H).

^{19}F NMR (376 MHz, $CDCl_3$) δ -70.8 (s, 3F).

^{13}C NMR (101 MHz, $CDCl_3$) δ 158.6 (q, *J* = 35.9 Hz), 130.4, 130.3, 130.2, 129.0, 129.0, 127.8, 119.8 (d, *J* = 278.7 Hz), 114.4, 114.1, 56.5, 55.4.

HRMS (ESI$^+$) calc: $[M+Na]^+$ ($C_{16}H_{14}F_3NNaO$) 316.0920, measured 316.0926, 2 ppm difference.

IR (neat) vmax/ cm^{-1}: 2931, 1669, 1495, 1333, 1190, 697.

General Defluorination Procedures

General Procedure D - Standard 0.5 mmol scale procedure

An oven-dried (180°C) H-type divided cell equipped with a porous glass frit was evacuated and backfilled with N_2 three times. $TEAPF_6$ (275 mg, 1 mmol, 2 equiv.) was added to both the catholyte and anolyte. Trifluoromethylketone substrate (0.5 mmol, 1 equiv.) was added to the catholyte and TBAB (320 mg, 1 mmol, 2 equiv.) was added to the anolyte. To each compartment was added (with stirring) degassed, anhydrous MeCN (2.5 mL, 0.2 M) and then TMSCl (0.19 mL, 1.5 mmol, 3 equiv.). Under a strong flow of N_2 the septa were replaced with ones containing platinum coil electrodes (entire coil area made of ~ 10 cm Pt wire submerged). A constant current of -5 mA was applied for 19300 s (2 *F*). When the electrolysis was finished, the catholyte was concentrated directly onto silica and purified by flash column chromatography or concentrated and triturated with Et_2O to yield the product.

Scale-up 5 mmol scale experiment

An oven-dried (180°C) H-type divided cell equipped with a porous glass frit was evacuated and backfilled with N_2 three times. $TEAPF_6$ (2.75 g, 10 mmol, 2 equiv.) was added to both the catholyte and anolyte. **1a** (1.14 g, 5.0 mmol, 1 equiv.) was added to the catholyte compartment and TBAB (3.2 g, 10 mmol, 2 equiv.) was added to the anolyte compartment. To each compartment was added degassed, anhydrous MeCN (25 mL, 0.2 M) and then TMSCl (1.9 mL, 15 mmol, 3 equiv.). Under a strong flow of N_2 the septa were replaced with ones containing a Nickel foil cathode (6.0 cm x 2.0 cm plate, 5.0 cm x 2.0 cm submerged area) and a graphite rod anode (60 mm diameter, 5.0 cm length submerged). A constant current of -30 mA was applied for 32167 s (2 *F*) with stirring at 1000 RPM (E_{cell} ~ -3.0 V rising to -3.7 V after ~1.3 F). When the electrolysis was finished, the catholyte was concentrated and triturated with cold Et_2O (30 mL). The suspension was filtered, and the filtrate concentrated to yield analytically pure product as an off white solid (989 mg, 94%).

Electrasyn 2.0 experiment

$TEAPF_6$ (275 mg, 1.0 mmol, 2 equiv.) was added to both the catholyte and anolyte of the IKA ProDivide cell. **1a** (114 mg, 0.5 mmol, 1 equiv.) was added to the catholyte compartment and TBAB (320 mg, 1 mmol, 2 equiv.) was added to the anolyte compartment. To each compartment was added degassed, anhydrous MeCN (2.5 mL, 0.2 M) and then TMSCl (0.19 mL, 15 mmol, 3 equiv.). The cell was sealed with a Ni cathode and Graphite anode (used as received with the IKA Electrasyn 2.0) in place and a N_2 inlet added through a syringe. A canula was placed between the catholyte and anolyte compartments to ensure pressure equalisation. A constant current of -5 mA was applied for 19300 s (2 *F*). When the electrolysis was finished, the catholyte was concentrated and triturated with cold Et_2O (30 mL). The suspension was filtered, and the filtrate concentrated and analysed by ^{19}F NMR relative to an internal C_6F_6 standard which showed 50% conversion of **1a** to yield 50% of **3a**. Note: given poor seals, leaving the reaction running for longer than 2 F in the ProDivide cell saw an increase in cell potential corresponding to the reduction of O_2.

Characterisation of Products

2,2-difluoro-1-(1-methyl-1H-indol-3-yl)ethan-1-one, **3a**

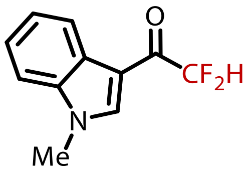

3a was synthesised from **1a** following **General Procedure D** and purified by Et_2O trituration of the concentrated crude material to yield an off white solid that turned pink on standing (101.9 mg, 97%).

R_f = 0.2 (20% EtOAc in Hexane)

^{1}H NMR (400 MHz, $CDCl_3$) δ: 8.42 – 8.31 (m, 1H), 8.02 (t, *J* = 1.8 Hz, 1H), 7.43 – 7.34 (m, 3H), 6.11 (t, *J* = 54.3 Hz, 1H), 3.91 (s, 3H).

^{13}C NMR (151 MHz, $CDCl_3$) δ: 182.7 (t, *J* = 25.2 Hz), 137.9 (t, *J* = 7.1 Hz), 137.2, 126.9, 124.2, 123.5, 122.5, 112.1 (t, *J* = 252.8 Hz), 110.1, 109.9, 33.8.

^{19}F NMR (376 MHz, $CDCl_3$) δ: -120.1 (d, *J* = 54.4 Hz).

Spectral data in accordance with literature.[14]

1-(4-bromophenyl)-2,2-difluoroethan-1-one, **3b**

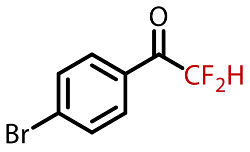

3b was synthesised from 4'-bromo-2,2,2-trifluoroacetophenone following **General Procedure D** and purified by Et_2O trituration of the concentrated crude material to yield a colourless solid (110.4 mg, 94%).

R_f = 0.3 (20% Et_2O in Hexane)

^{1}H NMR (400 MHz, $CDCl_3$) δ: 7.92 (d, *J* = 7.9, 2H), 7.75 (d, *J* = 8.8 Hz, 2H), 6.22 (t, *J* = 53.4 Hz, 1H).

^{13}C NMR (151 MHz, $CDCl_3$) δ: 186.9 (t, *J* = 25.9 Hz), 132.5, 131.1 (t, *J* = 2.5 Hz), 130.7, 130.2, 111.4 (t, *J* = 254.1 Hz).

^{19}F NMR (376 MHz, $CDCl_3$) δ: -121.5 (d, *J* = 53.9 Hz).

Spectral data in accordance with literature.[15]

2,2-difluoro-1-(4-fluorophenyl)ethan-1-one, **3c**

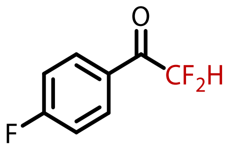

3c was synthesised from 4',2,2,2-tetrafluoroacetophenone following **General Procedure D** and purified using silica gel chromatography (2% Et_2O in Hexane to 19% Et_2O in Hexane) to yield a colourless oil (67.8 mg, 78%).

R_f = 0.3 (20% Et_2O in Hexane)

^{1}H NMR (400 MHz, $CDCl_3$) δ: 8.01 (dd, *J* = 8.7, 5.4 Hz, 2H), 7.13 (dd, *J* = 8.6 Hz, 1.0 Hz, 2H), 6.34 (t, *J* = 53.3 Hz, 1H).

^{13}C NMR (151 MHz, $CDCl_3$) δ: 187.9 (t, *J* = 25.4 Hz), 131.5, 131.1, 130.2, 129.1, 111.1 (t, *J* = 254.4 Hz).

^{19}F NMR (376 MHz, $CDCl_3$) δ: -122.8 (d, *J* = 53.5 Hz).

Spectral data in accordance with literature.[16]

1-(4-(dimethylamino)phenyl)-2,2-difluoroethan-1-one, **3d**

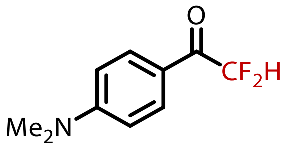

3d was synthesised from 1-(4-(dimethylamino)phenyl)-2,2,2-trifluoroethan-1-one following **General Procedure D** and purified using silica gel chromatography (2% Et_2O in Hexane to 19% Et_2O in Hexane) to yield a yellow solid (96.5 mg, 97%).

R_f = 0.3 (20% Et_2O in Hexane)

^{1}H NMR (400 MHz, $CDCl_3$) δ: 8.02 (d, *J* = 9.3, 2H), 6.73 (d, *J* = 9.2 Hz, 2H), 6.31 (t, *J* = 54.0 Hz, 1H), 3.11 (s, 6H).

^{13}C NMR (151 MHz, $CDCl_3$) δ: 185.1 (t, *J* = 24.2 Hz), 154.4, 132.0 (t, *J* = 2.3 Hz), 119.3, 111.3 (d, *J* = 110.2 Hz), 40.0.

^{19}F NMR (376 MHz, $CDCl_3$) δ: -120.9 (d, *J* = 54.1 Hz).

Spectral data in accordance with literature.[17]

2,2-difluoro-1-(4-methoxyphenyl)ethan-1-one, **3e**

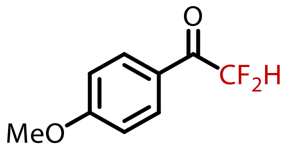

3e was synthesised from 2,2,2-trifluoro-1-(4-methoxyphenyl)ethan-1-one following **General Procedure D** and purified using silica gel chromatography (12% Et_2O in Hexane to 42% Et_2O in Hexane) to yield a colourless solid (81.8 mg, 88%).

$\mathbf{R_f}$ = 0.2 (20% Et_2O in Hexane)

^{1}H NMR (400 MHz, $CDCl_3$) δ: 8.44 – 7.91 (m, 2H), 7.12 – 6.61 (m, 2H), 6.32 (t, *J* = 53.7 Hz, 1H), 3.91 (s, 3H).

^{13}C NMR (151 MHz, $CDCl_3$) δ: 186.2 (t, *J* = 25.0 Hz), 165.0, 132.3 (t, *J* = 2.4 Hz), 124.6, 114.4, 111.6 (t, *J* = 253.7 Hz), 55.8.

^{19}F NMR (376 MHz, $CDCl_3$) δ: -121.3 (d, *J* = 53.9 Hz).

Spectral data in accordance with literature.[16]

2,2-difluoro-1-(4-methylphenyl)ethan-1-one, **3f**

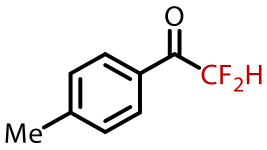

3f was synthesised from 2,2,2-trifluoro-1-(4-methylphenyl)ethan-1-one following **General Procedure D** and purified using silica gel chromatography (10% Et_2O in Hexane to 40% Et_2O in Hexane) to yield a colourless solid (71.4 mg, 84%).

R_f = 0.3 (20% Et_2O in Hexane)

^{1}H NMR (400 MHz, $CDCl_3$) δ: δ 7.96 (d, *J* = 8.1 Hz, 2H), 7.35 (d, *J* = 8.2 Hz, 2H), 6.29 (t, J = 53.5 Hz, 2H), 2.45 (s, 3H).

^{13}C NMR (151 MHz, $CDCl_3$) δ: 187.1 (t, *J* = 25.0 Hz), 146.2, 129.7 (t, *J* = 2.2 Hz), 129.7, 129.0, 111.3 (t, *J* = 254.1 Hz), 21.9.

^{19}F NMR (376 MHz, $CDCl_3$) δ: -121.8 (d, *J* = 53.7 Hz, 2F).

Spectral data in accordance with literature. [15]

2,2-difluoro-1-phenylethan-1-one, **3g**

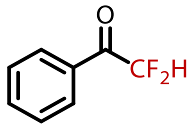

3g was synthesised from 2,2,2-trifluoroacetophenone following **General Procedure D** and purified using silica gel chromatography (10% Et_2O in Hexane to 19% Et_2O in Hexane) to yield a colourless oil (73.3 mg, 94%).

R_f = 0.2 (10% Et_2O in Hexane)

^{1}H NMR (400 MHz, $CDCl_3$) δ: 8.13 – 8.04 (m, 2H), 7.73 – 7.64 (m, 1H), 7.65 – 7.43 (m, 2H), 6.32 (t, *J* = 53.5 Hz, 1H).

^{13}C NMR (151 MHz, $CDCl_3$) δ: 187.5 (t, *J* = 25.1 Hz), 134.8, 131.3, 129.6, 129.1, 111.1 (t, *J* = 252.1 Hz)

^{19}F NMR (376 MHz, $CDCl_3$) δ: -121.8 (d, *J* = 53.6 Hz).

Spectral data in accordance with literature.[18]

1-(2,3-dihydrobenzofuran-5-yl)-2,2-difluoroethan-1-one, **3h**

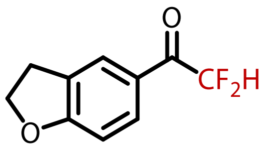

3h was synthesised from 1-(2,3-dihydrobenzofuran-5-yl)-2,2,2-trifluoroethan-1-one following **General Procedure D** and purified by using silica gel chromatography (18% Et_2O in Hexane to 27% Et_2O in Hexane) to yield a white solid (55.1 mg, 55%).

R_f = 0.3 (20% Et_2O in Hexane)

^{1}H NMR (400 MHz, $CDCl_3$) δ: 7.93 (d, *J* = 8.9 Hz, 2H), 6.86 (d, *J* = 8.4 Hz, 1H), 6.24 (t, *J* = 53.7 Hz, 1H), 4.70 (t, *J* = 8.8 Hz, 2H), 3.28 (t, *J* = 8.8 Hz, 2H).

^{13}C NMR (151 MHz, $CDCl_3$) δ: 185.9 (t, *J* = 24.9 Hz), 166.1, 132.2, 128.5, 127.1, 124.8, 111.7 (t, *J* = 253.8 Hz), 109.8, 72.6, 28.9.

^{19}F NMR (376 MHz, $CDCl_3$) δ: -121.0 (d, *J* = 53.9 Hz, 2F).

Spectral data in accordance with literature.[19]

1-(4-cyclopropylphenyl)-2,2-difluoroethan-1-one, **3i**

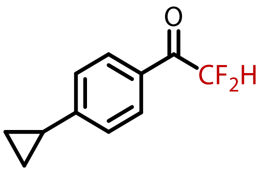

3i was synthesised from **1i** following **General Procedure D** and purified using silica gel chromatography (5% Et_2O in Hexane to 20% Et_2O in Hexane) to yield a colourless solid (80.3 mg, 82%).

R_f = 0.2 (20% Et_2O in Hexane)

^{1}H NMR (400 MHz, $CDCl_3$) δ: 8.01 (d, *J* = 8.1 Hz, 2H), 7.22 (d, *J* = 8.4 Hz, 2H), 6.32 (t, *J* = 53.6 Hz, 1H), 2.04 (tt, *J* = 8.4, 5.0 Hz, 1H), 1.25 – 0.94 (m, 2H), 0.81 (dt, *J* = 7.0, 4.8 Hz, 2H).

^{13}C NMR (151 MHz, $CDCl_3$) δ: 187.0 (t, *J* = 25.1 Hz), 152.9, 129.8, 128.8, 125.8, 111.3 (t, *J* = 253.6 Hz), 16.0, 10.9.

^{19}F NMR (376 MHz, $CDCl_3$) δ: -121.7 (d, *J* = 53.6 Hz).

Spectral data in accordance with literature.[16]

2,2-difluoro-1-(4-pentylphenyl)ethan-1-one, **3j**

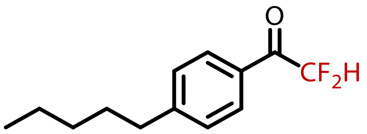

3j was synthesised from **1j** following **General Procedure D** and purified using silica gel chromatography (0% Et_2O in Hexane to 3% Et_2O in Hexane) to yield a yellow oil (102 mg, 90%).

R_f = 0.4 (10% Et_2O in Hexane)

^{1}H NMR (400 MHz, $CDCl_3$) δ: 7.99 (d, *J* = 8.0 Hz, 2H), 7.43 (d, *J* = 8.1 Hz, 2H), 6.28 (t, *J* = 53.6 Hz, 1H), 2.69 (t, *J* = 7.8 Hz, 2H), 1.65 (pent, *J* = 7.5 Hz, 2H), 1.33 (dhept, *J* = 7.0, 4.0, 3.3 Hz, 4H), 0.90 (t, *J* = 6.8 Hz, 3H).

^{13}C NMR (131 MHz, $CDCl_3$) δ: 187.2 (t, *J* = 25.0 Hz), 151.1, 129.8 (t, *J* = 2.3 Hz), 129.3 – 129.2 (m), 129.1, 111.3 (t, *J* = 253.6 Hz), 36.2, 31.4, 30.6, 22.5, 14.0.

^{19}F NMR (376 MHz, $CDCl_3$) δ: -121.8 (d, *J* = 53.6 Hz, 2F).

HRMS (ESI+) calc: [M+Na^+] ($C_{13}H_{16}NaOF_2$) 249.1061; measured: 249.1066 = 2 ppm difference.

IR (neat) vmax/ cm^{-1}: 2903, 1708, 1607, 1057, 572.

2,2-difluoro-1-(2-methyl-4-phenoxyphenyl)ethan-1-one, **3k**

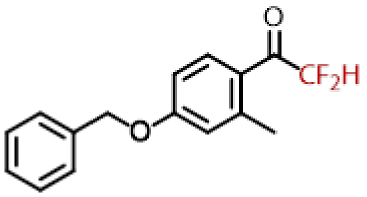

3k was synthesised from **1k** following **General Procedure D** and purified by using silica gel chromatography (7% Et_2O in Pentane to 60% Et_2O in Pentane) to yield a colourless solid (42.4 mg, 41%).

R_f = 0.6 (30% Et_2O in Hexane)

^{1}H NMR (400 MHz, $CDCl_3$) δ: δ 7.93 (dt, *J* = 8.7, 1.9 Hz, 1H), 7.43 – 7.32 (m, 5H), 6.97 – 6.84 (m, 2H), 6.24 (t, *J* = 53.9 Hz, 1H), 5.14 (s, 2H), 2.60 (s, 3H).

^{13}C NMR (151 MHz, $CDCl_3$) δ: 187.5 (t, *J* = 23.8 Hz), 162.6, 145.5, 135.9, 133.4 (t, *J* = 4.6 Hz), 128.8, 128.4, 127.5, 123.7, 119.1, 111.7, 111.3 (t, *J* = 254.2 Hz), 70.1, 22.6.

^{19}F NMR (376 MHz, $CDCl_3$) δ -120.5 (d, *J* = 54.0 Hz, 2F).

HRMS (ESI+) calc: [M+Na$^+$] ($C_{16}H_{14}F_2NaO_2$) 299.0854, measured 299.0857, 0.8 ppm difference.

IR (neat) vmax/ cm^{-1}:. 2954, 1719, 1635, 1407, 788, 564.

2,2-Difluoro-1-(4-(pyridin-2-yl)phenyl)ethan-1-one, **3l**

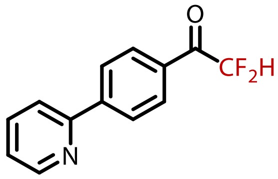

3l was synthesised from **1l** following **General Procedure D** and purified using silica gel chromatography (0% Et_2O in Hexane to 60% Et_2O in Hexane) to yield a white solid (45.5 mg, 39%).

R_f = 0.2 (20% Et_2O in Hexane)

^{1}H NMR (400 MHz, $CDCl_3$) δ: 8.75 (dt, *J* = 4.8, 1.4 Hz, 1H), 8.26 – 8.11 (m, 4H), 7.88 – 7.76 (m, 2H), 7.44 – 7.29 (m, 1H), 6.33 (t, *J* = 53.5 Hz, 1H).

^{13}C NMR (131 MHz, $CDCl_3$) δ: 187.3 (t, *J* = 25.4 Hz), 155.6, 150.1, 145.3, 137.0, 131.5 (t, *J* = 1.9 Hz), 130.2 (t, *J* = 2.3 Hz), 127.3, 123.4, 121.2, 111.3 (t, *J* = 253.7 Hz).

^{19}F NMR (376 MHz, $CDCl_3$) δ: -121.8 (d, *J* = 53.6 Hz, 2F).

HRMS (EI+) calc: [M^+] ($C_{13}H_9NOF_2$) 233.0647; measured: 233.0647 = 0 ppm difference.

IR (neat) vmax/ cm^{-1}: 2987, 1702, 1603, 1151, 1051.

1-([1,1'-biphenyl]-4-yl)-2,2-difluoroethan-1-one, **3m**

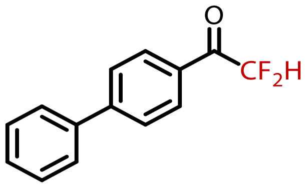

3m was synthesised from **1m** following **General Procedure D** and purified using silica gel chromatography (5% Et_2O in Hexane to 40% Et_2O in Hexane) to yield a yellow solid (113.4 mg, 96%).

R_f = 0.3 (20% Et_2O in Hexane)

^{1}H NMR (400 MHz, $CDCl_3$) δ: 8.24 – 8.15 (m, 2H), 7.93 – 7.72 (m, 2H), 7.65 – 7.69 (m, 2H), 7.54 – 7.44 (m, 3H), 6.32 (t, *J* = 53.5 Hz, 1H).

^{13}C NMR (151 MHz, $CDCl_3$) δ: 187.3 (t, *J* = 25.4 Hz), 147.7, 139.5, 130.4 (t, *J* = 2.4 Hz), 130.2, 129.2, 128.8, 127.7, 127.4, 111.4 (t, *J* = 253.8 Hz).

^{19}F NMR (376 MHz, $CDCl_3$) δ: -121.6 (d, *J* = 53.7 Hz).

Spectral data in accordance with literature.[20]

1-(4-(benzo[b]thiophen-3-yl)phenyl)-2,2-difluoroethan-1-one, **3n**

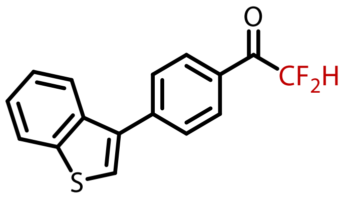

3n was synthesised from **1n** following **General Procedure D** and purified by using silica gel chromatography (12% Et_2O in Hexane to 52% Et_2O in Hexane) to yield a brown oil (125.3 mg, 87%).

R_f = 0.3 (20% Et_2O in Hexane)

^{1}H NMR (400 MHz, $CDCl_3$) δ: 8.25 – 8.18 (m, 2H), 7.99 – 7.99 (m, 2H), 7.80 – 7.75 (m, 2H), 7.55 (s, 1H), 7.48 – 7.48 (m, 2H), 6.34 (t, *J* = 53.5 Hz, 1H).

^{13}C NMR (151 MHz, $CDCl_3$) δ: 187.2 (t, *J* = 25.3 Hz), 142.7, 140.9, 137.2, 136.6, 130.4, 130.4, 129.1, 125.6, 125.0, 124.9, 123.2, 122.7, 111.5 (t, *J* = 253.8 Hz).

^{19}F NMR (376 MHz, $CDCl_3$) δ: -121.5 (d, *J* = 53.5 Hz).

HRMS (ESI+) calc: [M^+] ($C_{16}H_{10}OSF_2$) 288.0415, measured 288.0413, 0.7 ppm difference.

IR (neat) vmax/ cm^{-1}: 3011, 1703, 1603, 1137, 877.

1-(3'-amino-[1,1'-biphenyl]-4-yl)-2,2-difluoroethan-1-one **3o**

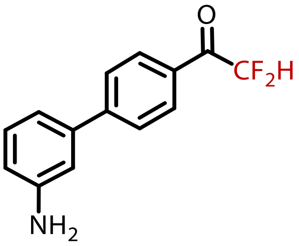

3o was synthesised from **1o** following **General Procedure D** and purified using silica gel chromatography (60% Et_2O in Pentane) to yield a pale yellow oil (112 mg, 91%).

R_f = 0.1 (40% Et_2O in Pentane)

^{1}H NMR (400 MHz, $CDCl_3$) δ: 8.20 – 8.10 (m, 2H), 7.78 – 7.68 (m, 2H), 7.32 – 7.23 (m, 1H), 7.10 – 7.03 (m, 1H), 6.98 – 6.93 (m, 1H), 6.83 – 6.73 (m, 1H), 6.34 (t, *J* = 53.5 Hz, 1H), 3.57 (brs, 2H).

^{13}C NMR (131 MHz, $CDCl_3$) δ: 187.2 (t, *J* = 25.3 Hz), 147.8, 146.9, 140.6, 130.2 (t, *J* = 2.3 Hz), 130.0, 127.5, 125.5, 117.8, 115.5, 113.8, 111.3 (t, *J* = 253.6 Hz).

^{19}F NMR (376 MHz, $CDCl_3$) δ: -121.7 (d, *J* = 53.4 Hz).

HRMS (ESI+) calc: [M+H$^+$] ($C_{14}H_{12}ONF_2$) 248.0881, measured 248.0878, 1.3 ppm difference.

IR (neat) vmax/ cm^{-1}: 3379, 2965, 1702, 1602, 1062, 769.

2,2-difluoro-1-(4-(2-methoxypyrimidin-5-yl)phenyl)ethan-1-one, **3p**

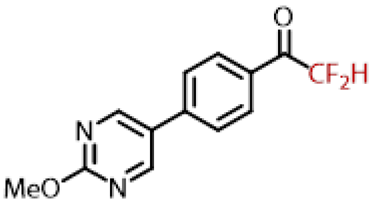

3p was synthesised from **1p** following **General Procedure D** and purified by using silica gel chromatography (24% Et_2O in Hexane to 50% Et_2O in Hexane) to yield a brown oil (92.4mg, 71%). Despite numerous attempts at purification, **3p** was isolated with *ca*. 10% of an unidentified inseparable impurity and the yield has been adjusted accordingly.

R_f = 0.3 (20% Et_2O in Hexane)

^{1}H NMR (400 MHz, $CDCl_3$) δ: 8.79 (s, 2H), 8.25-8.12 (m, 2H), 7.72 – 7.67 (m, 2H), 6.30 (t, *J* = 53.5 Hz, 1H), 4.09 (s, 3H).

^{13}C NMR (151 MHz, $CDCl_3$) δ: 187.0 (t, *J* = 25.8 Hz), 165.7, 157.6, 140.8, 132.1, 130.7 (t, *J* = 2.5 Hz), 128.6, 126.8, 111.4 (t, *J* = 254.0 Hz), 55.3.

^{19}F NMR (376 MHz, $CDCl_3$) δ: -121.5 (d, *J* = 53.4 Hz).

HRMS (EI+) calc: [M^+] ($C_{13}H_{10}N_2O_2F_2$) 264.0705, measured 264.0705, 0 ppm difference.

IR (neat) vmax/ cm^{-1}: 2986, 1706, 1596, 1411, 878.

1-(3-(2,6-diphenylpyrimidin-4-yl)phenyl)-2,2-difluoroethan-1-one, **3q**

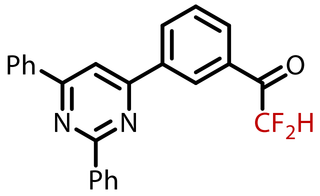

This product, **3q** was synthesised from **1q** following **General Procedure D** and purified by using silica gel chromatography (24% Et_2O in Hexane to 50% Et_2O in Hexane) to yield a brown oil (82.7mg, 37%).

R_f = 0.3 (20% Et_2O in Hexane)

^{1}H NMR (400 MHz, $CDCl_3$) δ: 8.94 (s, 1H), 8.76 – 8.70 (m, 2H), 8.66 – 8.60 (m, 1H), 8.36 – 8.28 (m, 2H), 8.26 – 8.21 (m, 1H), 8.05 (s, 1H), 7.78 – 7.71 (m, 1H), 7.63 – 7.51 (m, 6H), 6.39 (t, J = 53.4 Hz, 1H).

^{13}C NMR (151 MHz, $CDCl_3$) δ: 187.4 (t, J = 25.6 Hz), 165.3, 164.8, 163.1, 138.7, 137.8, 137.2, 133.5, 131.1, 130.9, 129.7, 129.0, 128.6, 128.5, 128.3 (t, J = 2.1 Hz), 127.4, 111.3 (t, J = 253.9 Hz), 110.3.

^{19}F NMR (376 MHz, $CDCl_3$) δ: -121.6 (d, J = 53.3 Hz).

HRMS (APCI+) calc: [M+H$^+$] ($C_{24}H_{16}N_2OF_2$) 387.1303, measured 387.1302, 0.3 ppm difference.

IR (neat) vmax/ cm^{-1}: 2891, 1702, 1505, 1152, 782.

(E)-1-(4-(4-(dibutylamino)styryl)phenyl)-2,2-difluoroethan-1-one, **3r**

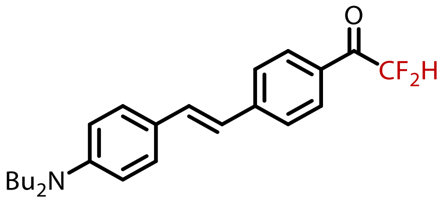

3r was synthesised from **1r** following **General Procedure D** and purified using silica gel chromatography (0% Et_2O in Hexane to 15% Et_2O in Hexane) to yield an orange solid (165.8 mg, 86%).

R_f = 0.4 (10% Et_2O in Hexane)

^{1}H NMR (400 MHz, $CDCl_3$) δ: 8.02 (d, *J* = 8.2 Hz, 2H), 7.57 (d, *J* = 8.4 Hz, 2H), 7.41 (d, *J* = 8.7 Hz, 2H), 7.21 (d, *J* = 16.2 Hz, 1H), 6.89 (d, *J* = 16.2 Hz, 1H), 6.64 (d, *J* = 8.8 Hz, 2H), 6.29 (t, *J* = 53.6 Hz, 1H), 3.35 – 3.27 (m, 4H), 1.65 – 1.53 (m, 4H), 1.38 (h, *J* = 7.4 Hz, 4H), 0.97 (t, *J* = 7.4 Hz, 6H).

^{13}C NMR (131 MHz, $CDCl_3$) δ: 186.6 (t, *J* = 25.0 Hz), 148.6, 145.3, 133.3, 130.2 (t, *J* = 2.3 Hz), 129.0 (t, *J* = 1.7 Hz), 128.6, 126.0, 123.4, 121.6, 111.6, 111.3 (t, *J* = 253.5 Hz), 50.8, 29.5, 20.4, 14.0.

^{19}F NMR (376 MHz, $CDCl_3$) δ: -121.6 (d, *J* = 53.7 Hz, 2F).

HRMS (ESI+) calc: [M+H$^+$] ($C_{24}H_{30}OF_2$) 386.2290; measured: 386.2301 = 3 ppm difference.

IR (neat) vmax/ cm^{-1}: 2969, 1697, 1583, 1181, 878.

2,2-Difluoro-1-(4-(pyridin-2-yl)phenyl)ethan-1-one, **3s**

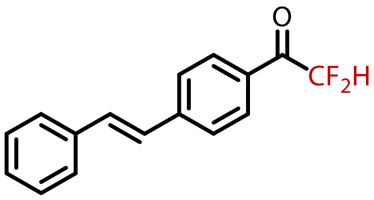

3s was synthesised from **1s** following **General Procedure D** and purified using silica gel chromatography (5% Et_2O in Hexane to 40% Et_2O in Hexane) to yield a white solid (105.9 mg, 82%).

R_f = 0.3 (20% Et_2O in Hexane)

^{1}H NMR (400 MHz, $CDCl_3$) δ: 8.06 (d, *J* = 8.2 Hz, 2H), 7.63 (d, *J* = 8.5 Hz, 2H), 7.59 – 7.50 (m, 2H), 7.44 – 7.23 (m, 4H), 7.13 (d, *J* = 16.3 Hz, 2H), 6.29 (t, *J* = 53.5 Hz, 1H).

^{13}C NMR (131 MHz, $CDCl_3$) δ: 186.9 (t, *J* = 25.3 Hz), 143.9, 136.5, 132.9, 130.3, 130.2, 129.0, 128.8, 127.1, 127.1, 126.9, 111.4 (t, *J* = 253.8 Hz).

^{19}F NMR (376 MHz, $CDCl_3$) δ: -121.6 (d, *J* = 53.6 Hz, 2F).

HRMS (ESI+) calc: [M+Na^+] ($C_{16}H_{12}NaOF_2$) 281.0748; measured: 281.0762 = 5 ppm difference.

IR (neat) vmax/ cm^{-1}: 3675, 2901, 1702, 1074, 691.

1-(Anthracen-9-yl)-2,2-difluoroethan-1-one, **3t**

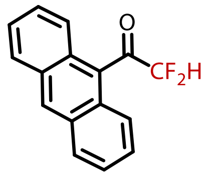

3t was synthesised from 1-(Anthracen-9-yl)-2,2,2-trifluoroethan-1-one following **General Procedure D** and purified using silica gel chromatography (7% Et_2O in Hexane to 20% Et_2O in Hexane) to yield an orange-yellow solid (96 mg, 75%).

R_f = 0.3 (20% Et_2O in Hexane)

^{1}H NMR (400 MHz, $CDCl_3$) δ: 8.61 (s, 1H), 8.11 – 8.04 (m, 2H), 7.85-7.74 (m, 2H), 7.62 – 7.56 (m, 4H), 6.35 (t, *J* = 53.9 Hz, 1H).

^{13}C NMR (131 MHz, $CDCl_3$) δ: 198.1 (t, *J* = 25.9 Hz), 130.8, 130.6, 129.0, 128.6, 127.7, 125.8, 124.0, 109.5 (t, *J* = 253.3 Hz).

^{19}F NMR (376 MHz, $CDCl_3$) δ: -124.8 (d, *J* = 54.1 Hz, 2F).

HRMS (ESI+) calc: [M+Na$^+$] ($C_{16}H_{10}ONaF_2$) 279.0592; measured: 279.0604 = 4 ppm difference.

IR (neat) vmax/ cm^{-1}: 2988, 1722, 1449, 1394, 1027.

2,2-difluoro-1-(naphthalen-2-yl)ethan-1-one, **3u**

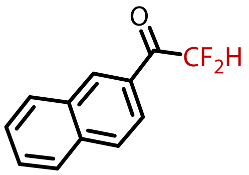

3u was synthesised from **1u** following **General Procedure D** and purified using silica gel chromatography (5% Et_2O in Hexane to 20% Et_2O in Hexane) to yield a colourless solid (96.8 mg, 94%).

R_f = 0.3 (20% Et_2O in Hexane)

^{1}H NMR (400 MHz, $CDCl_3$) δ: 8.63 (d, *J* = 1.4 Hz, 1H), 8.24 – 7.81 (m, 4H), 7.72 – 7.58 (m, 2H), 6.42 (t, *J* = 53.5 Hz, 1H).

^{13}C NMR (151 MHz, $CDCl_3$) δ: 187.7 (t, *J* = 25.2 Hz), 136.4, 132.6 (t, *J* = 3.3 Hz), 132.4, 130.2, 129.8, 129.1, 128.9, 128.0, 127.4, 124.2, 111.5 (t, *J* = 253.8 Hz).

^{19}F NMR (376 MHz, $CDCl_3$) δ: -121.1 (d, *J* = 53.6 Hz).

Spectral data in accordance with literature.[15]

1,1'-(4-(dimethylamino)naphthalene-1,3-diyl)bis(2,2-difluoroethan-1-one), **3v**

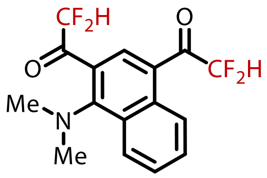

3v was synthesised from 1,1'-(4-(dimethylamino)naphthalene-1,3-diyl)bis(2,2,2-trifluoroethan-1-one) following **General Procedure D** on a 0.25 mmol scale at -10 mA and purified using silica gel chromatography (65% Et_2O in Hexane) to yield a yellow solid (111.3 mg, 68%).

R_f = 0.2 (50% Et_2O in Hexane)

^{1}H NMR (400 MHz, $CDCl_3$) δ: 9.03 (ddd, *J* = 8.6, 1.3, 0.6 Hz, 1H), 8.42 (s, 1H), 8.23 (ddd, *J* = 8.6, 1.3, 0.6 Hz, 1H), 7.69 (ddd, *J* = 8.5, 6.9, 1.4 Hz, 1H), 7.64 – 7.57 (m, 1H), 6.38 (d, *J* = 53.9 Hz, 1H), 6.32 (t, *J* = 53.9 Hz, 1H), 3.15 (s, 6H).

^{13}C NMR (131 MHz, $CDCl_3$) δ: 189.4 (t, *J* = 25.2 Hz), 187.6 (t, *J* = 24.1 Hz), 158.2, 134.9, 132.6 (tt, *J* = 5.0, 3.0 Hz), 131.1, 130.6, 130.5, 126.7, 126.6, 126.4, 121.9 (d, *J* = 20.6 Hz), 111.5 (t, *J* = 256.9 Hz), 110.2 (t, *J* = 256.9 Hz), 45.8.

^{19}F NMR (376 MHz, $CDCl_3$) δ: -119.1 (d, *J* = 53.9 Hz, 2F), -122.2 (d, *J* = 54.0 Hz, 2F).

HRMS (ESI+) calc: [$M+Na^+$] ($C_{16}H_{13}NaNO_2F_4$) 350.0775; measured: 350.0771 = -1 ppm difference.

IR (neat) vmax/ cm^{-1}: 2988, 2901, 1688, 1401, 1088.

tert-Butyl 3-(2,2-difluoroacetyl)-1H-indole-1-carboxylate, **3w**

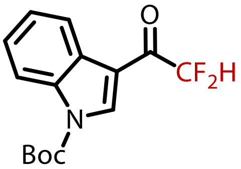

3w was synthesised from **1w** following **General Procedure D** and purified by using silica gel chromatography (19% Et_2O in Hexane to 32% Et_2O in Hexane) to yield a colourless solid (60.1 mg, 41%).

R_f = 0.2 (20% Et_2O in Hexane)

^{1}H NMR (400 MHz, $CDCl_3$) δ: 8.51 (t, *J* = 1.7 Hz, 1H), 8.42 – 8.33 (m, 1H), 8.16 (dd, *J* = 7.4, 1.8 Hz, 1H), 7.41 (dd, *J* = 7.2, 1.4 Hz, 2H), 6.15 (t, *J* = 53.9 Hz, 1H), 1.72 (s, 9H).

^{13}C NMR (151 MHz, $CDCl_3$) δ: 184.2 (t, *J* = 26.0 Hz), 148.7, 135.2, 134.9 (t, *J* = 7.2 Hz), 127.4, 126.5, 125.2, 122.4, 115.2, 114.1, 111.5 (t, *J* = 253.9 Hz), 86.2, 28.1.

^{19}F NMR (376 MHz, $CDCl_3$) δ: -121.2 (d, *J* = 53.8 Hz, 2F).

HRMS (ESI+) calc: [M+Na$^+$] ($C_{15}H_{15}F_2NNaO_3$) 318.0912, measured 318.0906, 1.8 ppm difference.

IR (neat) vmax/ cm^{-1}: 2925, 1655, 1526, 1396, 841, 749.

2,2-Difluoro-1-(1-(perfluoropyridin-4-yl)-1H-indol-3-yl)ethan-1-one, **3x**

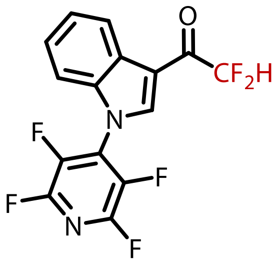

3x was synthesised from **1x** following **General Procedure D** and purified by trituration with Et_2O to yield a yellow solid (151 mg, 92%).

R_f = 0.2 (30% Et_2O in Hexane)

^{1}H NMR (400 MHz, $CDCl_3$) δ: 8.52 – 8.45 (m, 1H), 8.25-8.14 (m, 1H), 7.54 – 7.42 (m, 2H), 7.28 – 7.22 (m, 1H), 6.15 (t, *J* = 53.9 Hz, 1H).

^{13}C NMR (151 MHz, $CDCl_3$) δ: 183.7 (t, *J* = 26.3 Hz), 144.3 (dtd, *J* = 247.9, 14.6, 3.3 Hz), 140.0 – 136.1 (m), 136.0 – 135.6 (m), 128.7 – 128.3 (m), 126.3, 126.0, 125.4, 125.1, 123.1, 114.8 (t, *J* = 2.5 Hz), 111.8 (t, *J* = 253.0 Hz), 110.9 (t, *J* = 2.6 Hz).

^{19}F NMR (376 MHz, $CDCl_3$) δ: -85.5 – -85.9 (m, 2F), -120.7 (d, *J* = 53.9 Hz, 2F), -144.8 – -145.2 (m, 2F).

HRMS (ESI+) calc: [M+Na^+] ($C_{15}H_6ONaN_2F_6$) 367.0277; measured: 367.0262 = 4 ppm difference.

IR (neat) vmax/ cm^{-1}: 2897, 1663, 1536, 1209, 746.

1-(1-benzyl-1H-indol-3-yl)-2,2-difluoroethan-1-one, **3y**

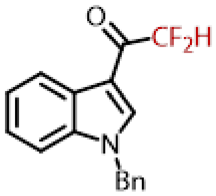

3y was synthesised from **1y** following **General Procedure D** and purified by using silica gel chromatography (24% Et_2O in Hexane to 50% Et_2O in Hexane) to yield a colourless solid (50.1 mg, 35%).

R_f = 0.2 (20% Et_2O in Hexane)

^{1}H NMR (400 MHz, $CDCl_3$) δ: 8.43 (d, *J* = 7.6 Hz, 1H), 8.08 (s, 1H), 7.38 – 7.30 (m, 6H), 7.18 (d, *J* = 7.0 Hz, 2H), 6.11 (t, *J* = 54.3 Hz, 1H), 5.40 (s, 2H).

^{13}C NMR (151 MHz, $CDCl_3$) δ: 182.9 (t, *J* = 25.3 Hz), 137.3 (t, *J* = 7.2 Hz), 136.7, 135.1, 129.2, 128.5, 126.9, 124.4, 123.7, 122.7, 112.1 (t, *J* = 253.9 Hz), 110.6, 51.2, 29.7.

^{19}F NMR (376 MHz, $CDCl_3$) δ: -120.2 (d, *J* = 54.2 Hz).

HRMS (ESI+) calc: [M+Na$^+$] ($C_{17}H_{13}F_2NONa$) 308.0857 measured 309.0858, 0.1 ppm difference.

IR (neat) vmax/ cm^{-1}: 2981, 1739, 1655, 1467, 746, 571.

2,2-difluoro-1-(1H-indol-3-yl)ethan-1-one, **3z**

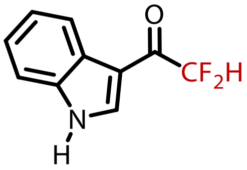

3z was synthesised from 2,2,2-trifluoro-1-(1H-indol-3-yl)ethan-1-one following **General Procedure D** and purified by purified using silica gel chromatography (9% EtOAc in Hexane to 18% EtOAc in Hexane) to yield a white solid (44.0 mg, 45%).

$\mathbf{R_f}$ = 0.1 (20% EtOAc in Hexane)

^{1}H NMR (400 MHz, $CDCl_3$) δ: 11.31 (s, 1H), 8.22 (m, 1H), 8.11 (s, 1H), 7.42 (m, 1H), 7.21 (d, *J* = 5.1 Hz, 2H), 6.12 (t, *J* = 54.4 Hz, 1H).

^{13}C NMR (151 MHz, $CDCl_3$) δ: 183.1 (t, *J* = 24.4 Hz), 136.6, 135.1 (t, *J* = 6.6 Hz), 126.1, 123.9, 123.1, 121.8, 112.2, 111.7 (t, *J* = 252.9 Hz), 111.1 (d, J= 1.7 Hz).

^{19}F NMR (376 MHz, $CDCl_3$) δ: -121.1 (d, *J* = 54.4 Hz, 2F).

Spectral data in accordance with literature.[19]

2,2-difluoro-1-(6-methoxy-1-methyl-1H-indol-3-yl)ethan-1-one , **3aa**

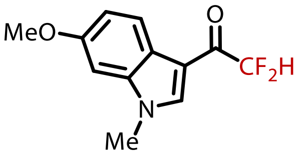

3aa was synthesised from **1aa** following **General Procedure D** and purified using silica gel chromatography (15% Et_2O in Hexane to 42% Et_2O in Hexane) to yield an off-white solid (98.4 mg, 87%). *Note: Product **3aa** appears to be sensitive to light and precautions to exclude light were taken during purification and characterisation.*

$\mathbf{R_f}$ = 0.3 (30% Et_2O in Hexane)

^{1}H NMR (400 MHz, $CDCl_3$) δ: 8.24 (dd, *J* = 8.7, 0.5 Hz, 1H), 7.87 (t, *J* = 1.7 Hz, 1H), 6.98 (dd, *J* = 8.8, 2.3 Hz, 1H), 6.79 – 6.76 (m, 1H), 6.09 (t, *J* = 54.3 Hz, 1H), 3.88 (s, 3H), 3.80 (s, 3H).

^{13}C NMR (131 MHz, $CDCl_3$) δ: 182.7 (t, *J* = 25.1 Hz), 157.9, 138.2, 137.3, 123.3, 120.8, 112.7, 112.1 (t, *J* = 253.8 Hz), 110.6, 93.8, 55.8, 33.9.

^{19}F NMR (376 MHz, $CDCl_3$) δ: -120.1 (d, *J* = 54.5 Hz, 2F).

HRMS (ESI+) calc: [$M+Na^+$] ($C_{12}H_{11}NaNO_2F_2$) 262.0650; measured: 262.0647 = -1 ppm difference.

IR (neat) vmax/ cm^{-1}: 3103, 2904, 1651, 1254, 1077.

.

3-(2,2-difluoroacetyl)-1-methyl-1H-indole-5-carbonitrile, **3ab**

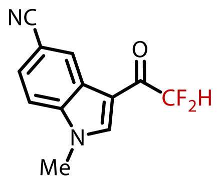

3ab was synthesised from **1ab** following **General Procedure D** and purified using silica gel chromatography (82% Et_2O in Hexane to 100% Et_2O in Hexane) to yield a white solid (49.2 mg, 42%).

R_f = 0.1 (30% Et_2O in Hexane)

^{1}H NMR (400 MHz, $CDCl_3$) δ: 8.77 (dd, *J* = 1.6, 0.7 Hz, 1H), 8.13 (d, *J* = 1.8 Hz, 1H), 7.62 (dd, *J* = 8.6, 1.6 Hz, 1H), 7.48 (d, *J* = 8.6 Hz, 1H), 6.09 (t, *J* = 54.1 Hz, 1H), 3.95 (s, 3H).

^{13}C NMR (131 MHz, $CDCl_3$) δ: 182.9 (t, *J* = 26.2 Hz), 139.4 (t, *J* = 7.3 Hz), 138.7, 127.9, 127.3, 126.8, 119.6, 112.1 (t, *J* = 253.9 Hz), 111.0, 110.6, 107.1, 34.1.

^{19}F NMR (376 MHz, $CDCl_3$) δ: -120.3 (d, *J* = 54.4 Hz, 2F).

HRMS (ESI+) calc: [M+Na$^+$] ($C_{12}H_8NaN_2OF_2$) 257.0497; measured: 257.0492 = 2 ppm difference.

IR (neat) vmax/ cm^{-1}: 2972, 2248, 1656, 1451, 1028.

2,2-difluoro-1-(4-fluoro-1-methyl-1H-indol-3-yl)ethan-1-one, **3ac**

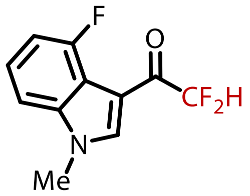

3ac was synthesised from **1ac** following **General Procedure D** and purified by using silica gel chromatography (0% Et_2O in Hexane to 20% Et_2O in Hexane) to yield a colourless solid (95.1 mg, 84%).

R_f = 0.3 (20% Et_2O in Hexane)

^{1}H NMR (400 MHz, $CDCl_3$) δ: 8.03 (d, *J* = 1.6 Hz, 1H), 7.32 (td, *J* = 8.1, 4.6 Hz, 1H), 7.18 (d, *J* = 8.2 Hz, 1H), 7.03 (dd, *J* = 10.9, 7.9 Hz, 1H), 6.22 (t, *J* = 54.3 Hz, 1H), 3.90 (s, 3H).

^{13}C NMR (151 MHz, $CDCl_3$) δ: 181.3 (t, *J* = 25.2 Hz), 156.6 (d, *J* = 253.8 Hz), 140.1 (d, *J* = 10.7 Hz), 138.5 (t, *J* = 6.7 Hz), 125.1 (d, *J* = 7.9 Hz), 114.5, 114.4, 112.3 (t, *J* = 251.3), 109.4 (d, *J* = 21.7 Hz), 106.2 (d, *J* = 4.1 Hz), 34.3.

^{19}F NMR (376 MHz, $CDCl_3$) δ: -108.3 – -108.8 (m, 1F), -119.7 (d, *J* = 54.3 Hz, 2F).

HRMS (ESI+) calc: [M+Na^+] ($C_{11}H_8F_3NNaO$) 250.0450, measured 250.0449, 0.5 ppm difference.

IR (neat) vmax/ cm^{-1}: 2937, 1663, 1532, 1379, 770.

1-(4-chloro-1-methyl-1H-indol-3-yl)-2,2-difluoroethan-1-one, **3ad**

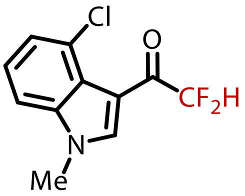

3ad was synthesised from **1ad** following **General Procedure D** and purified by using silica gel chromatography (18% Et_2O in Hexane to 40% Et_2O in Hexane) to yield a yellow solid (77.9 mg, 64%).

R_f = 0.2 (30% Et_2O in Hexane)

^{1}H NMR (400 MHz, $CDCl_3$) δ: 7.99 (s, 1H), 7.32 (dd, *J* = 5.3, 3.4 Hz, 2H), 7.25 (d, *J* = 2.1 Hz, 1H), 6.14 (t, *J* = 54.3 Hz, 1H), 3.87 (s, 3H)

^{13}C NMR (151 MHz, $CDCl_3$) δ: 181.3 (t, *J* = 24.9 Hz), 139.2 (t, *J* = 7.5 Hz), 139.0, 127.7, 125.1, 124.8, 124.3, 112.8 (t, *J* = 255.9), 110.1, 108.8, 34.2.

^{19}F NMR (376 MHz, $CDCl_3$) δ: -118.6 (d, *J* = 54.3, 2F).

HRMS (EI+) calc: [M^+] ($C_{11}H_8F_2NO^{35}Cl$) 243.0257, measured 243.0257, 0 ppm difference.

IR (neat) vmax/ cm^{-1}: 2912, 2134, 2032, 1601, 910.

1-(4-Bromo-1-methyl-1H-indol-3-yl)-2,2-difluoroethan-1-one, **3ae**

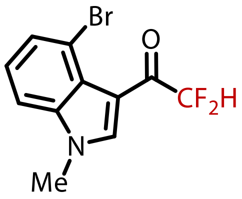

3ae was synthesised from **1ae** following **General Procedure D** and purified by using silica gel chromatography (0% Et_2O in Hexane to 20% Et_2O in Hexane) to yield a brown solid (72.3 mg, 51%).

R_f = 0.3 (20% Et_2O in Hexane)

^{1}H NMR (400 MHz, $CDCl_3$) δ: 7.96 (t, *J* = 1.7 Hz, 1H), 7.51 (d, *J* = 7.7 Hz, 1H), 7.29 (d, *J* = 8.1 Hz, 1H), 7.17 (t, *J* = 7.9 Hz, 1H), 6.14 (t, *J* = 54.5 Hz, 1H), 3.85 (s, 3H).

^{13}C NMR (151 MHz, $CDCl_3$) δ: 181.7 (d, *J* = 24.7 Hz), 139.4 (d, *J* = 6.1 Hz), 139.3, 129.1, 126.5, 125.3, 115.3, 113.0 (t, *J* = 254.3 Hz), 111.1, 109.7, 34.6

^{19}F NMR (376 MHz, $CDCl_3$) δ: -118.7 (d, *J* = 54.5 Hz).

HRMS (EI+) calc: [M^+] ($C_{11}H_8NO^{79}BrF_2$) 286.9752, measured 286.9752, 0 ppm difference.

IR (neat) vmax/ cm^{-1}: 3109, 2901, 1678, 1099, 880.

1-(5-Bromo-1-methyl-1H-indol-3-yl)-2,2-difluoroethan-1-one, **3af**

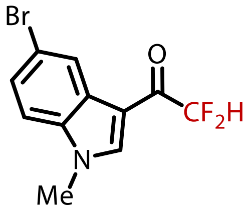

3af was synthesised from **1af** following **General Procedure D** and purified by using silica gel chromatography (0% Et_2O in Hexane to 20% Et_2O in Hexane) to yield a brown solid (99.0 mg, 69%).

R_f = 0.3 (20% Et_2O in Hexane)

^{1}H NMR (400 MHz, $CDCl_3$) δ: 8.53 (d, *J* = 2.0 Hz, 1H), 7.97 (t, *J* = 1.8 Hz, 1H), 7.44 (dd, *J* = 8.7, 2.0 Hz, 1H), 7.31 – 7.23 (m, 1H), 6.07 (t, *J* = 54.2 Hz, 1H), 3.87 (s, 3H).

^{13}C NMR (151 MHz, $CDCl_3$) δ: 181.2 (d, *J* = 24.7 Hz), 139.0 (d, *J* = 6.1 Hz), 138.9, 128.8, 126.1, 124.9, 115.0, 112.7 (t, *J* = 254.3 Hz), 110.6, 109.2, 34.1.

^{19}F NMR (376 MHz, $CDCl_3$) δ: -120.1 (d, *J* = 54.2 Hz, 2F).

HRMS (EI+) calc: [M^+] ($C_{11}H_8NO^{79}BrF_2$) 286.9752, measured 286.9750, 0.7 ppm difference.

IR (neat) vmax/ cm^{-1}: 2920, 1663, 1041, 874, 738.

2,2-difluoro-1-(5-iodo-1-methyl-1H-indol-3-yl)ethan-1-one, **3ag**

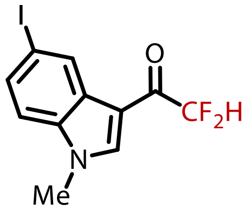

3ag was synthesised from **1ag** following **General Procedure D** and purified by using silica gel chromatography (0% Et_2O in Hexane to 30% Et_2O in Hexane) to yield a brown solid (75.0 mg, 45%).

R_f = 0.2 (20% Et_2O in Hexane)

^{1}H NMR (400 MHz, $CDCl_3$) δ: 8.76 (d, *J* = 1.8 Hz, 1H), 7.94 (d, *J* = 1.8 Hz, 1H), 7.64 (dd, *J* = 8.6, 1.7 Hz, 1H), 7.15 (d, *J* = 8.6 Hz, 1H), 6.07 (t, *J* = 54.2 Hz, 1H), 3.88 (s, 3H).

^{13}C NMR (151 MHz, $CDCl_3$) δ: 182.7 (t, *J* = 25.6 Hz), 138.1 (t, *J* = 7.2 Hz), 136.4, 132.9, 131.4, 129.0, 112.2 (t, *J* = 254.2 Hz), 111.8, 109.6, 88.0, 34.0.

^{19}F NMR (376 MHz, $CDCl_3$) δ: -120.0 (d, *J* = 54.2 Hz, 2F).

HRMS (ESI+) calc: [M+Na$^+$] ($C_{11}H_8F_2INNaO$) 357.9511 measured 357.9512, 0.4 ppm difference.

IR (neat) vmax/ cm^{-1}: 2966, 1649, 1467, 1076, 750.

2,2-difluoro-1-(1-methyl-2-(thiophen-2-yl)-1H-indol-3-yl)ethan-1-one, **3ah**

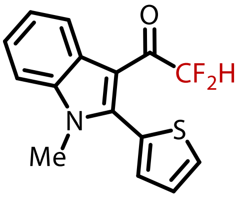

3ah was synthesised from **1ah** following **General Procedure D** and purified by using silica gel chromatography (0% Et_2O in Hexane to 29% Et_2O in Hexane) to yield a white solid (100.1 mg, 69%).

R_f = 0.2 (20% Et_2O in Hexane)

^{1}H NMR (400 MHz, $CDCl_3$) δ: 8.49 – 8.39 (m, 1H), 7.68 (dd, *J* = 5.1, 1.3 Hz, 1H), 7.45 – 7.35 (m, 3H), 7.30 (dd, *J* = 3.6, 1.2 Hz, 1H), 7.25 – 7.21 (m, 1H), 5.66 (t, *J* = 53.8 Hz, 1H), 3.61 (s, 3H).

^{13}C NMR (151 MHz, $CDCl_3$) δ: 182.8 (t, *J* = 23.8 Hz), 139.4, 137.3, 132.4, 130.2, 129.2, 127.9, 126.7, 124.8, 123.9, 122.7, 114.3, 110.0, 107.1 (t, *J* = 246.8 Hz), 31.1.

^{19}F NMR (376 MHz, $CDCl_3$) δ: -124.6 (d, *J* = 53.9 Hz, 2F).

HRMS (ESI+) calc: [M+Na^+] ($C_{15}H_{11}F_2NNaOS$) 314.0422, measured 314.0411, 3.4 ppm difference.

IR (neat) vmax/ cm^{-1}: 2923, 1651, 1466, 1052, 752.

2,2-difluoro-1-(1-methyl-2-phenyl-1H-indol-3-yl)ethan-1-one, **3ai**

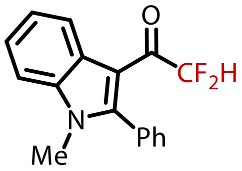

3ai was synthesised from **1ai** following **General Procedure D** and purified by using silica gel chromatography (16% Et_2O in Hexane to 24% Et_2O in Hexane) to yield a colourless solid (93.0 mg, 65%).

R_f = 0.2 (20% Et_2O in Hexane)

^{1}H NMR (400 MHz, $CDCl_3$) δ: 8.55 – 8.40 (m, 1H), 7.66 – 7.51 (m, 3H), 7.48 – 7.36 (m, 5H), 5.54 (t, *J* = 53.9 Hz, 1H), 3.54 (s, 3H).

^{13}C NMR (151 MHz, $CDCl_3$) δ: 182.6 (t, *J* = 23.9 Hz), 147.9, 137.0, 130.5, 130.4, 130.3, 128.9, 126.8, 124.4, 123.9, 122.7, 112.3, 109.9, 107.1 (t, *J* = 246.8 Hz), 31.1.

^{19}F NMR (376 MHz, $CDCl_3$) δ: -125.2 (d, *J* = 53.7 Hz, 2F).

HRMS (ESI+) calc: [M+Na$^+$] ($C_{17}H_{13}F_2NNaO$) 308.0857, measured 308.0853, 1.5 ppm difference.

IR (neat) vmax/ cm^{-1}: 2966, 1649, 1467, 1076, 750.

2,2-difluoro-1-(1-methyl-1H-indol-5-yl)ethan-1-one, **3aj**

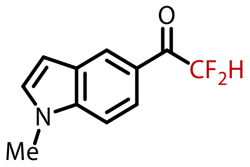

3aj was synthesised from **1aj** following **General Procedure D** and purified using silica gel chromatography (35% Et_2O in Hexane) to yield an off-white solid that turned orange on storage (98.7 mg, 94%).

R_f = 0.2 (20% Et_2O in Hexane)

^{1}H NMR (400 MHz, $CDCl_3$) δ: 8.43 (q, *J* = 1.2 Hz, 1H), 7.96 (ddd, *J* = 8.7, 1.7, 0.8 Hz, 1H), 7.39 (dt, *J* = 8.7, 0.8 Hz, 1H), 7.15 (d, *J* = 3.2 Hz, 1H), 6.65 (dd, *J* = 3.2, 0.9 Hz, 1H), 6.41 (t, *J* = 53.8 Hz, 1H), 3.83 (s, 3H).

^{13}C NMR (131 MHz, $CDCl_3$) δ: 187.4 (t, *J* = 24.2 Hz), 140.0, 131.1, 128.2, 125.1 (t, *J* = 3.0 Hz), 123.6, 122.9, 111.5 (t, *J* = 253.3 Hz), 109.9, 103.7, 33.2.

^{19}F NMR (376 MHz, $CDCl_3$) δ: -120.9 (d, *J* = 54.0 Hz, 2F).

HRMS (ESI+) calc: [M+Na$^+$] ($C_{11}H_{10}NaNOF_2$) 232.0559; measured: 262.0561 = -2.3 ppm difference.

IR (neat) vmax/ cm^{-1}: 2987, 2900, 1693, 1604, 991.

2,2-difluoro-1-(9-methyl-9H-carbazol-3-yl)ethan-1-one, **3ak**

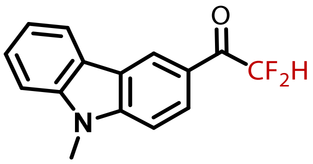

3ak was synthesised from **1ak** following **General Procedure D** and purified using silica gel chromatography (30% Et_2O in Pentane) to yield a pale yellow oil (119 mg, 92%).

R_f = 0.3 (30% Et_2O in Pentane)

^{1}H NMR (400 MHz, $CDCl_3$) δ: 8.76 (dt, *J* = 1.7, 0.9 Hz, 1H), 8.16 (ddt, *J* = 8.8, 1.8, 0.9 Hz, 1H), 8.10 (dt, *J* = 7.8, 1.0 Hz, 1H), 7.54 (ddd, *J* = 8.3, 7.2, 1.2 Hz, 1H), 7.40 (dt, *J* = 8.2, 0.9 Hz, 1H), 7.38 – 7.30 (m, 2H), 3.80 (s, 3H).

^{13}C NMR (101 MHz, $CDCl_3$) δ: 186.8 (t, *J* = 24.5 Hz), 144.5, 141.8, 127.5, 127.0, 123.4 (t, *J* = 2.6 Hz), 122.9, 122.9, 122.8, 120.8, 120.7, 111.7 (t, *J* = 253.4 Hz), 109.3, 108.7, 29.4.

^{19}F NMR (376 MHz, $CDCl_3$) δ: -120.6 (d, *J* = 53.7 Hz, 2F).

HRMS (ESI+) calc: [M+H$^+$] ($C_{15}H_{11}ONF_2$) 260.0881; measured: 260.0887 = 2 ppm difference.

IR (neat) vmax/ cm^{-1}: 3056, 2938, 1683, 1589, 1117, 747.

2,2-difluoro-1-(1H-pyrrol-2-yl)ethan-1-one, **3al**

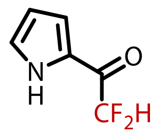

3al was synthesised from 2-trifluoroacetyl-1H-pyrrole following **General Procedure D** and purified using silica gel chromatography (19% Et_2O in Hexane to 29% Et_2O in Hexane) to yield a white solid (39.4 mg, 62%).

R_f = 0.2 (30% Et_2O in Hexane)

^{1}H NMR (400 MHz, $CDCl_3$) δ: 9.81 (brs, 1H), 7.23 (t, *J* = 2.1 Hz, 2H), 6.43 (t, *J* = 3.2 Hz, 1H), 6.22 (t, *J* = 53.9 Hz, 1H).

^{13}C NMR (151 MHz, $CDCl_3$) δ: 177.6 (t, *J* = 25.7 Hz), 128.0, 127.3, 120.6, 112.3, 110.5 (t, *J* = 251.6 Hz).

^{19}F NMR (376 MHz, $CDCl_3$) δ: -122.8 (d, *J* = 53.8 Hz).

Spectral data in accordance with literature.[17]

1-(4-(Dimethylamino)pyridin-3-yl)-2,2-difluoroethan-1-one, **3am**

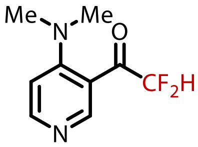

3am was synthesised from 1-(4-(Dimethylamino)pyridin-3-yl)-2,2,2-trifluoroethan-1-one following **General Procedure D** and purified using silica gel chromatography (20% Et_2O in Hexane to 80% Et_2O in Hexane) to yield a yellow solid (91.8 mg, 92%).

R_f = 0.15 (20% Et_2O in Hexane)

^{1}H NMR (400 MHz, $CDCl_3$) δ: 8.71 (s, 1H), 8.30 (d, *J* = 6.3 Hz, 1H), 6.76 (d, *J* = 6.2 Hz, 1H), 6.34 (t, *J* = 53.9 Hz, 1H), 2.93 (s, 6H).

^{13}C NMR (131 MHz, $CDCl_3$) δ: 187.3 (t, *J* = 25.0 Hz), 155.7, 152.1 (t, *J* = 4.1 Hz), 151.7, 116.2, 109.8 (t, *J* = 252.8 Hz), 109.7, 42.9.

^{19}F NMR (376 MHz, $CDCl_3$) δ: -122.0 (d, *J* = 54.1 Hz, 2F).

HRMS (ESI+) calc: [$M+H^+$] ($C_9H_{11}ON_2F_2$) 201.0834; measured: 201.0841 = -3 ppm difference.

IR (neat) vmax/ cm^{-1}: 2978, 1686, 1593, 1393, 976.

2,2-difluoro-1-(1-methyl-1H-pyrazol-4-yl)ethan-1-one, **3an**

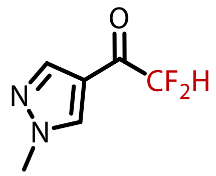

3an was synthesised from **1an** following **General Procedure D** and purified using silica gel chromatography (5% Et_2O in Hexane to 60% Et_2O in Hexane) to yield a colourless oil (73.6 mg, 92%).

R_f = 0.2 (30% Et_2O in Hexane)

^{1}H NMR (400 MHz, $CDCl_3$) δ: 8.12 (d, *J* = 4.7 Hz, 2H), 6.02 (t, *J* = 54.0 Hz, 1H), 4.05 (s, 3H).

^{13}C NMR (151 MHz, $CDCl_3$) δ: 182.4 (t, *J* = 26.8 Hz), 141.7 (t, *J* = 2.3 Hz), 134.4 (t, *J* = 3.8 Hz), 117.6 (t, *J* = 2.5 Hz), 111.2 (t, *J* = 253.2 Hz), 39.5.

^{19}F NMR (376 MHz, $CDCl_3$) δ: -123.3 (d, *J* = 54.0 Hz).

Spectral data in accordance with literature.[21]

2,2-difluoro-1-(1-methyl-1H-pyrazol-4-yl)ethan-1-one, **3ao**

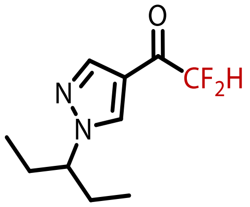

3ao was synthesised from **1ao** following **General Procedure D** and purified using silica gel chromatography (5% Et_2O in Hexane to 30% Et_2O in Hexane) to yield a colourless oil (103.6 mg, 96%).

R_f = 0.2 (20% Et_2O in Hexane)

^{1}H NMR (400 MHz, $CDCl_3$) δ: 8.24 – 7.73 (m, 2H), 6.04 (t, *J* = 53.9 Hz, 1H), 3.93 (tt, *J* = 9.4, 5.0 Hz, 1H), 2.42 – 1.78 (m, 4H), 0.82 (t, *J* = 7.4 Hz, 6H).

^{13}C NMR (151 MHz, $CDCl_3$) δ: 182.7 (t, *J* = 26.6 Hz), 141.6 (t, *J* = 2.5 Hz), 133.3 (t, *J* = 3.4 Hz), 116.8, 111.3 (t, *J* = 253.0 Hz), 67.8, 28.1, 10.6.

^{19}F NMR (376 MHz, $CDCl_3$) δ: -123.3 (d, *J* = 54.0 Hz).

Spectral data in accordance with literature.[21]

(Z)-1,1-difluorononadec-10-en-2-one, **3ap**

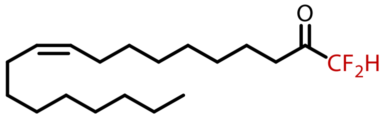

3ap was synthesised from **1ap** following **General Procedure D** and purified using silica gel chromatography (4% Et_2O in Hexane to 40% Et_2O in Hexane) to yield a colourless oil (80.1 mg, 51%). *Note:* ***3ap*** *was isolated alongside c.a 4% TBAT that remained from the synthesis of the precursor* ***1ap****, the yield has been adjusted to reflect this.*

R_f = 0.3 (10% Et_2O in Hexane)

^{1}H NMR (400 MHz, $CDCl_3$) δ: 5.67 (t, *J* = 54.0 Hz, 1H), 5.42 – 5.38 (m, 2H), 2.70 (t, *J* = 7.3 Hz, 2H), 2.07 – 1.95 (m, 4H), 1.69 – 1.59 (m, 2H), 1.39 – 1.21 (m, 20H), 0.91 – 0.85 (m, 3H).

^{13}C NMR (131 MHz, $CDCl_3$) δ: 200.0 (t, *J* = 26.1 Hz), 130.1, 129.7, 109.9 (t, *J* = 252.9 Hz), 36.0, 31.9, 29.8, 29.7, 29.5, 29.3, 29.2, 29.0, 28.9, 28.7, 27.23, 27.15, 22.7, 22.3, 14.1.

^{19}F NMR (376 MHz, $CDCl_3$) δ: -126.8 (d, *J* = 54.0 Hz, 2F).

HRMS (ESI+) calc: [M+Na$^+$] ($C_{19}H_{34}NaOF_2$) 339.2470; measured: 339.2465 = 1.5 ppm difference.

IR (neat) vmax/ cm^{-1}: 2923, 1746, 1452, 1027, 891.

2,2-difluoro-*N*-(4-fluorophenyl)acetamide, **3as**

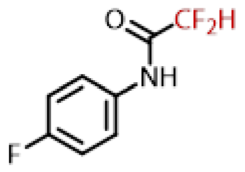

3as was synthesised from **1as** following **General Procedure D** and purified by using silica gel chromatography (20% EtOAc in Hexane) to yield a colourless solid (35.0 mg, 38%).

R_f = 0.2 (20% EtOAc in Hexane)

^{1}H NMR (400 MHz, $CDCl_3$) δ: 7.98 (s, 1H), 7.55 (dd, *J* = 8.9, 4.7 Hz, 2H), 7.07 (t, *J* = 8.6 Hz, 2H), 6.02 (t, *J* = 54.3 Hz, 1H).

^{19}F NMR (376 MHz, $CDCl_3$) δ: -115.6 - -115.9 (m, 1F), -125.5 (d, *J* = 54.2 Hz, 2F).

^{13}C NMR (131 MHz, $CDCl_3$) δ: 160.3 (t, *J* = 24.5 Hz), 160.2 (d, *J* = 245.8 Hz), 131.6 (d, *J* = 3.0 Hz), 122.2 (d, *J* = 8.1 Hz), 116.0 (d, *J* = 22.8 Hz), 108.5 (t, *J* = 254.2 Hz).

Spectral data in accordance with literature.[16]

Less successful and unsuccessful substrates

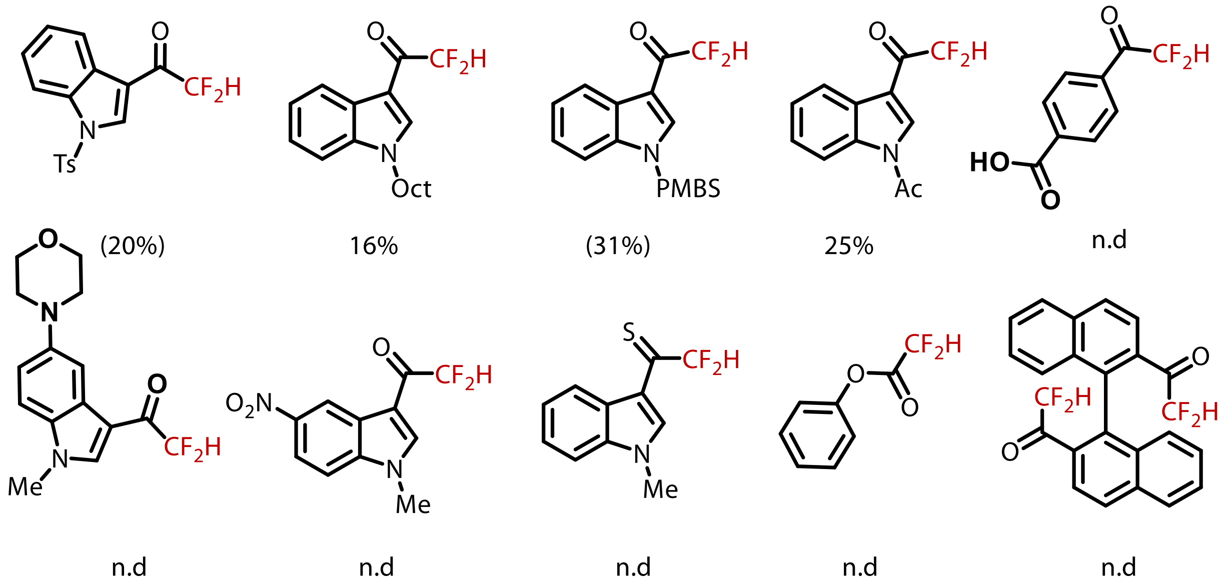

Reductive cleavage of Ts, PMBS, Ac and Bn groups was observed, owing to the low yield defluorination of indoles with those protecting groups. Carboxylic acid substitution saw competing proton reduction and no defluorination was observed (the corresponding anion likely also has a much lower reduction potential) Nitro-substitution and N-octyl protection led to decreased solubility in MeCN, though ultimately only nitro group reduction was observed when the reaction conducted in DMF (in which the substrate is soluble). Extensive decomposition was observed with thioketone containing substrates (likely polymerisation pathways) and ester cleavage observed when trifluoro acetyl esters used.

Electrochemical Set Up

Electrodes

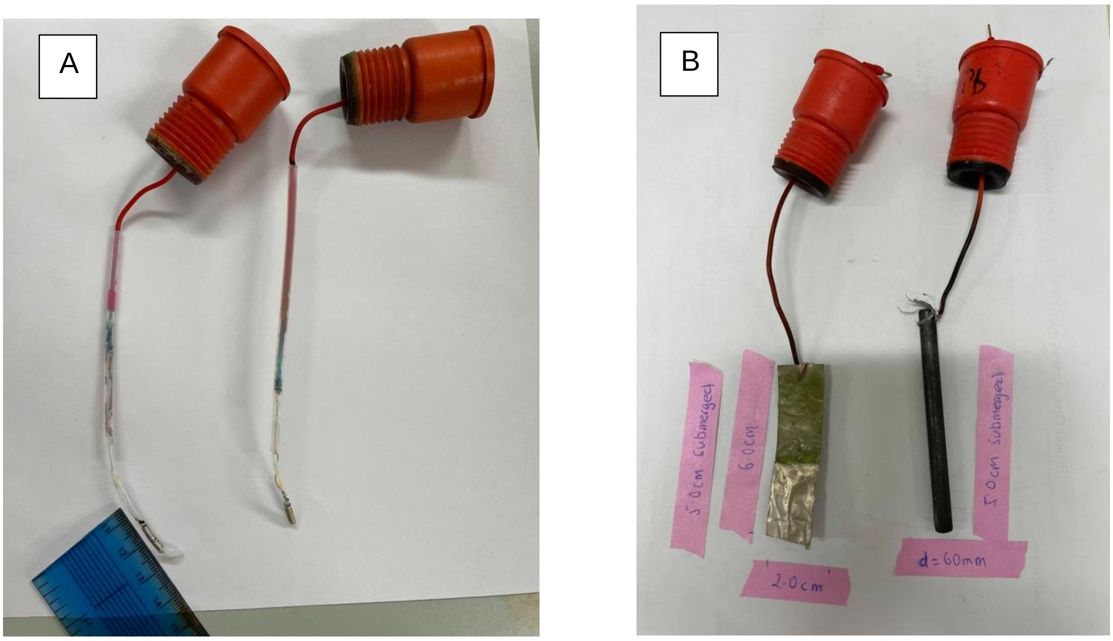

Figure S2. a) Platinum coil electrodes used in General Procedure D. b) Ni foil and Graphite rod electrodes used for scale-up experiment in General procedure C.

Platinum electrodes were made by wrapping platinum wire around PTFE tubing to create a surface area approximately ~1 cm^2. The platinum wire was then fed through PTFE tubing by creating a small hole on the side of the tubing. The wire was then spot- welded to a copper wire. Using a large gauge needle as a guide, the wire was fed through a new suba seal.

<u>Cell set-up</u>

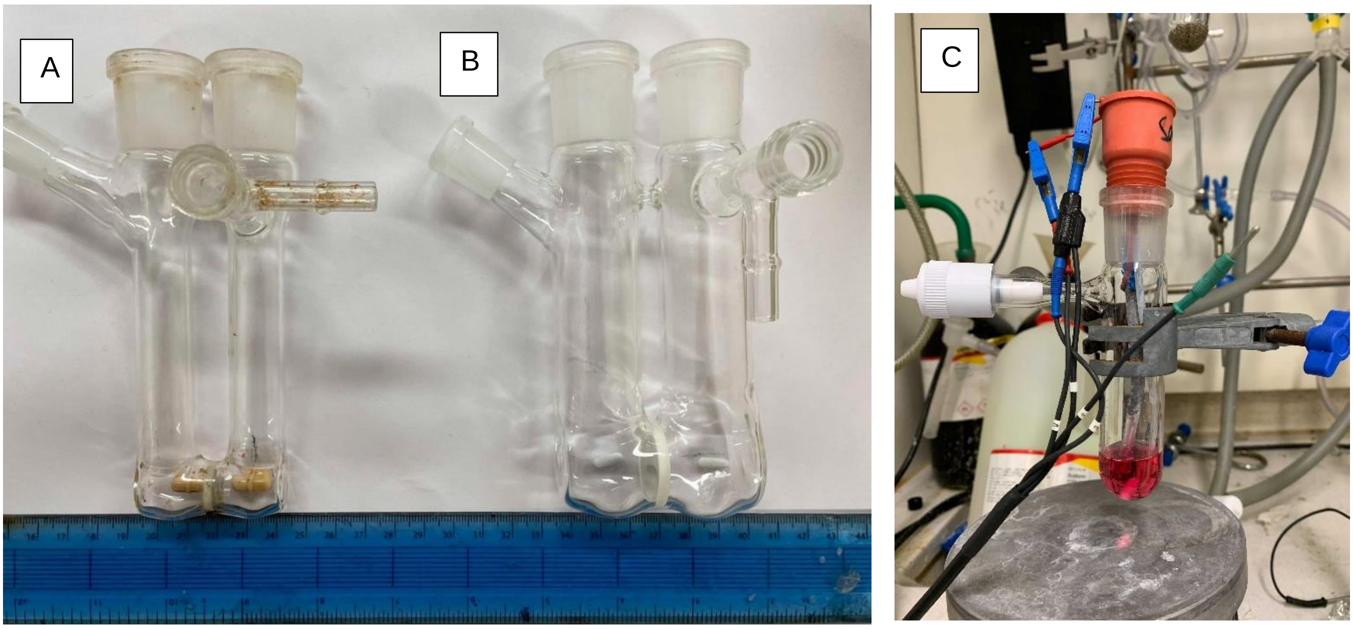

Figure S3. a) H-Cell used for General Procedure D, anolyte and catholyte capacity ~ 3 mL per side. b) H-Cell used for scale-up (General procedure C), anolyte and catholyte capacity ~ 30 mL per side. c) Undivided cell used in entry 2, table S1.

Electrasyn experiment

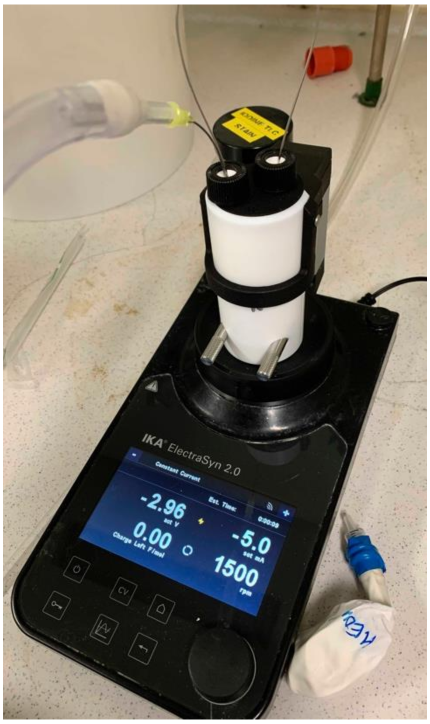

Figure S4. Electrasyn 2.0 with IKA ProDivide cell.

Using electrodes supplied with the Electrasyn 2.0 alongside the IKA ProDivide cell. A canula was inserted through the septa of both chambers with a nitrogen inlet inserted into the cathodic chamber.

Product Diversification Experiments

Hydride Reduction

1-(4-bromo-1-methyl-1H-indol-3-yl)-2,2-difluoroethan-1-ol, **6**

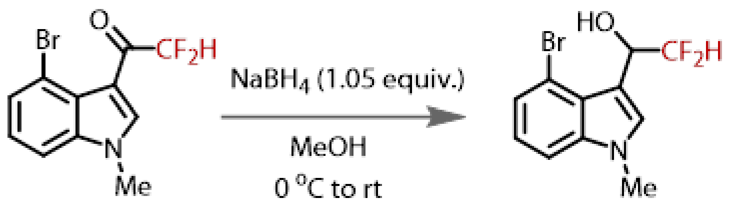

In a 7 mL vial, under air, $NaBH_4$ (37.8 mg, 1.05 mmol, 1.05 equiv.) was added in one portion to a MeOH (2.5 mL) solution of **3ae** (288 mg, 1.0 mmol, 1 equiv.) at 0°C. The vial was capped and allowed to warm to RT with stirring. When complete (*c.a* 1 hour) as determined by TLC (5% IPA in $CHCl_3$), the crude mixture was concentrated directly onto silica and purified by silica gel chromatography (1% IPA in $CHCl_3$ to 10% IPA in $CHCl_3$) to yield the alcohol product as a pink oil (281 mg, 94%).

R_f = 0.3 (5% IPA in $CHCl_3$)

^{1}H NMR (400 MHz, $CDCl_3$) δ: 7.31 – 7.20 (m, 3H), 7.04 (t, *J* = 7.9 Hz, 1H), 6.09 (td, *J* = 55.7, 3.1 Hz, 1H), 5.95 – 5.85 (m, 1H), 3.71 (s, 3H), 2.64 (s, 1H).

^{13}C NMR (151 MHz, $CDCl_3$) δ: 138.2, 125.1, 124.5, 122.9, 115.8 (t, *J* = 245.3 Hz), 113.4, 111.7 – 110.3 (m), 109.2, 66.6 – 65.9 (m), 33.3.

^{19}F NMR (376 MHz, $CDCl_3$) δ: -125.5 (ddd, *J* = 278.1, 55.3, 9.2 Hz, 1F), -131.1 (ddd, *J* = 278.1, 55.7, 14.6 Hz, 1F).

HRMS (ESI+) calc: [M+Na$^+$] ($C_{11}H_{10}NaNO^{79}BrF_2$) 311.9806; measured: 311.9809 = -0.8 ppm difference.

IR (neat) vmax/ cm^{-1}: 3969, 3058, 2966, 1065, 729.

[1]**H NMR** (400 MHz, $CDCl_3$):

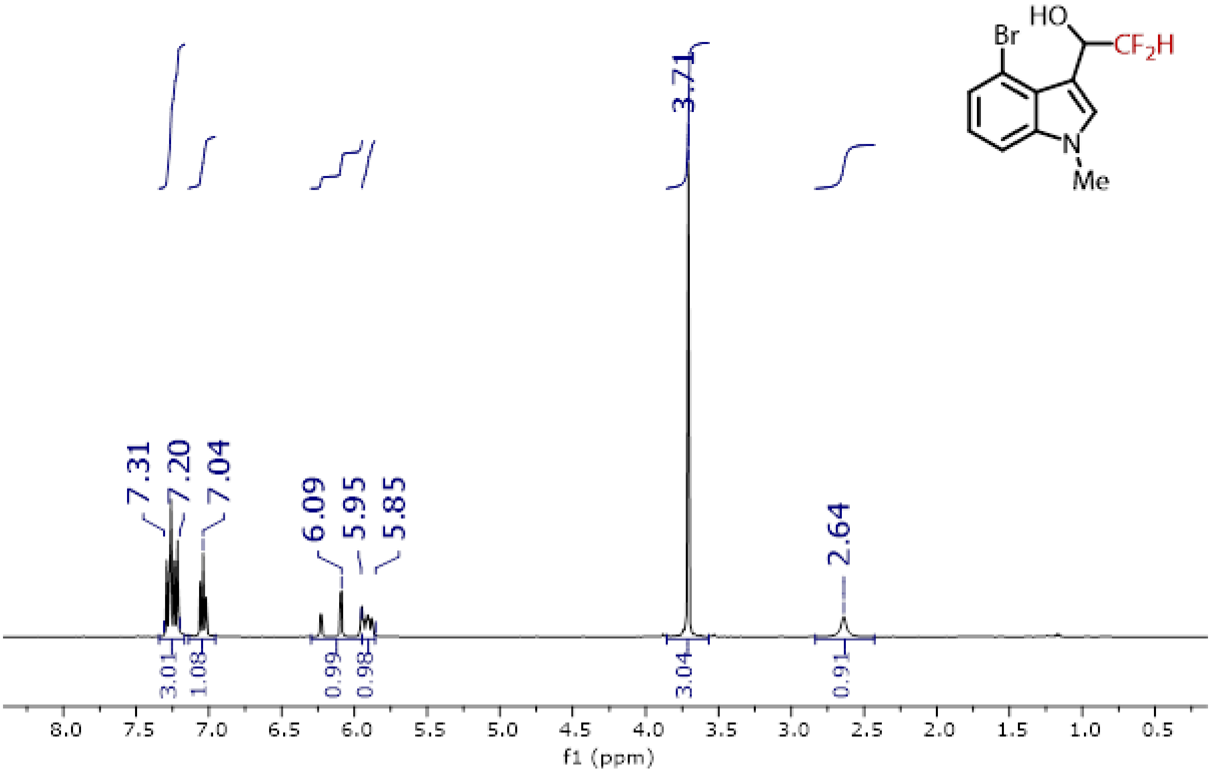

[13]**C NMR** (131 MHz, $CDCl_3$):

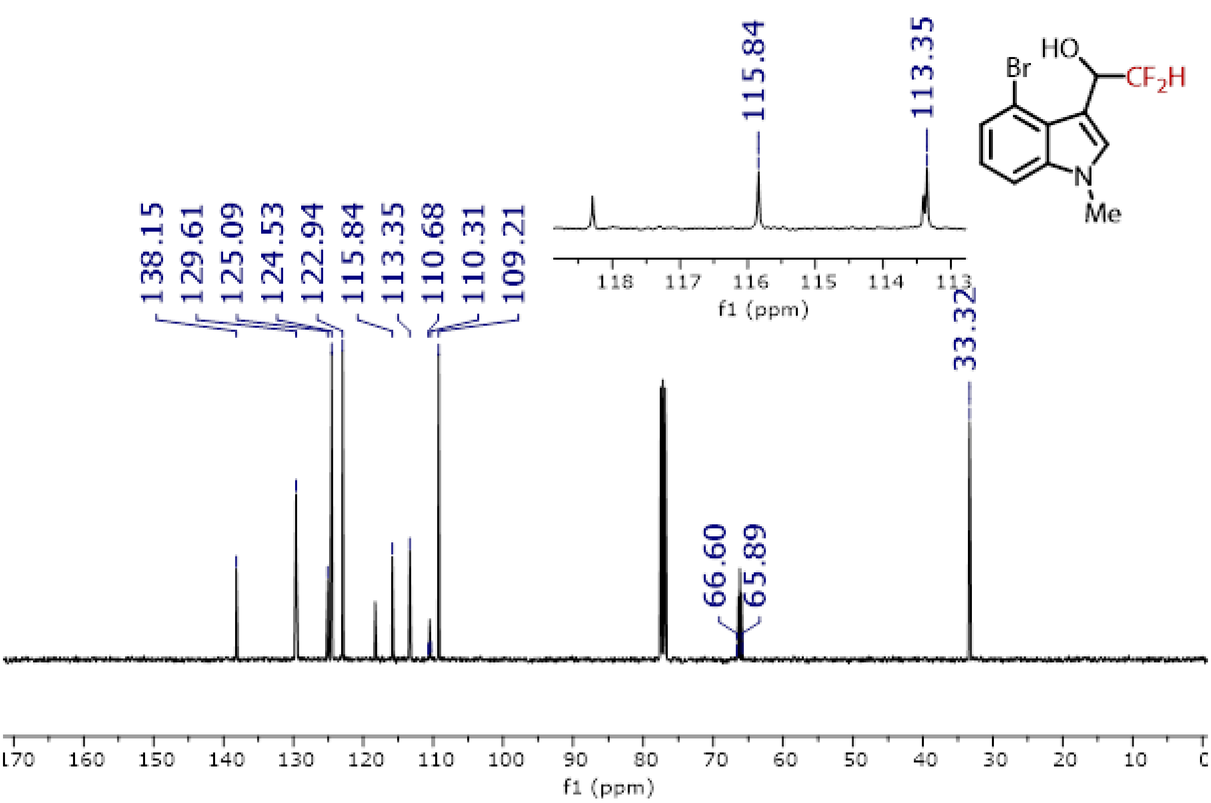

19F NMR (376 MHz, $CDCl_3$):

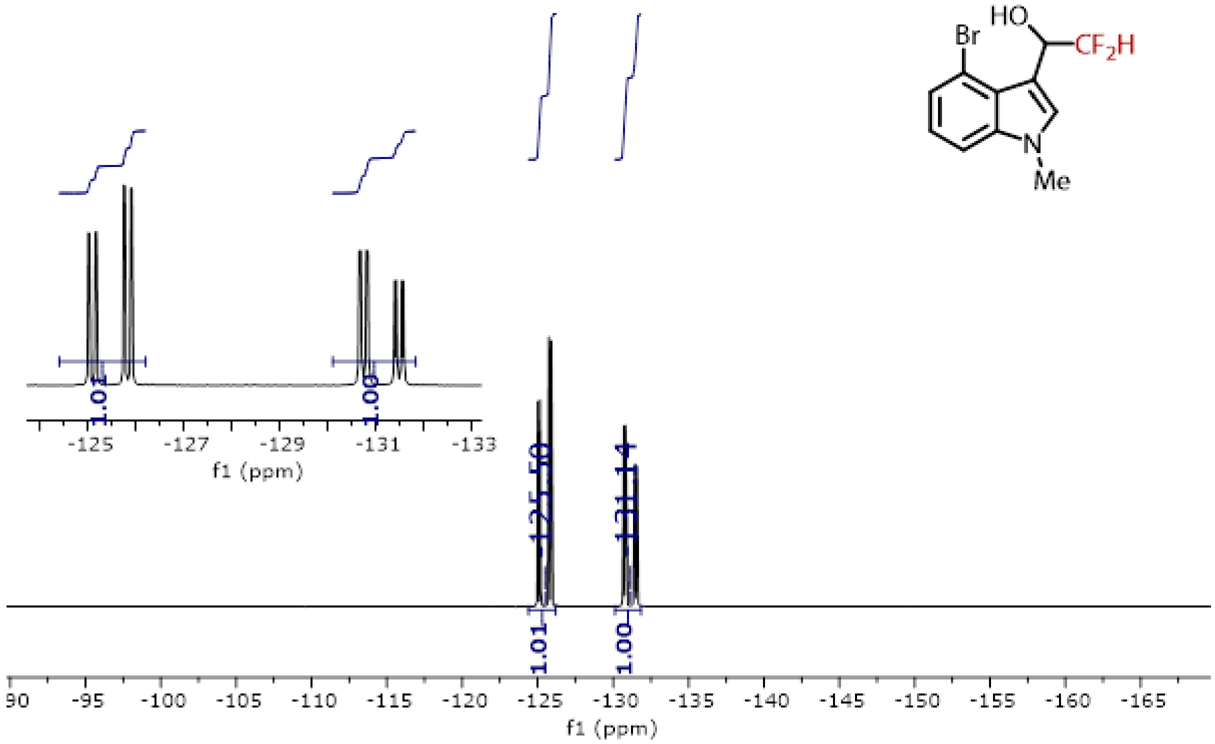

Suzuki-Miyaura Coupling

1-(4-(benzo[d][1,3]dioxol-5-yl)-1-methyl-1H-indol-3-yl)-2,2-difluoroethan-1-one, **11**

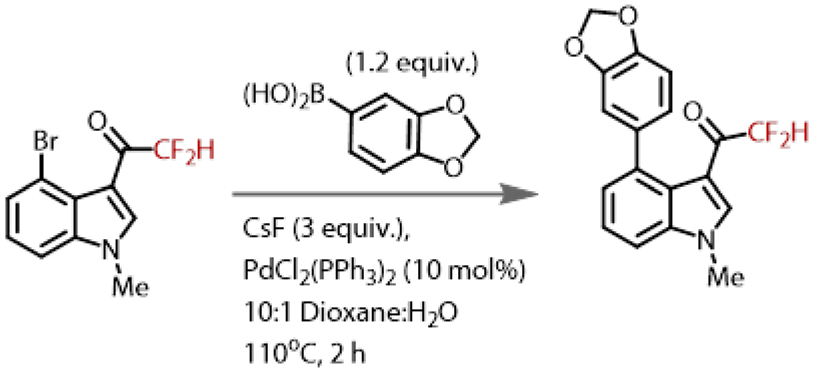

In a flame-dried Schlenk flask, under N_2, was added **3ae** (864 mg, 3 mmol, 1 equiv.), benzo[d][1,3]dioxol-5-ylboronic acid (597 mg, 3.6 mmol, 1.2 equiv.), CsF (1.37 g, 9 mmol, 3 equiv.) and $PdCl_2(PPh_3)_2$ (211 mg, 0.3 mmol, 10 mol%). A 10:1 dioxane:H_2O mixture (20 mL) was added and the flask placed into a pre-heated oil bath at 110°C with stirring. The solution was stirred for 2 h, allowed to cool and filtered through Celite, eluting with DCM. The resulting solution was concentrated and purified by chromatography on silica gel (30% Et_2O in Hexane to 70% Et_2O in Hexane) to yield the title material as a brown oil that solidified on standing (823 mg, 83%).

R_f = 0.3 (40% Et_2O in Hexane)

^{1}H NMR (400 MHz, $CDCl_3$) δ: 8.05 (d, *J* = 1.5 Hz, 1H), 7.46 – 7.36 (m, 2H), 7.29 (dd, *J* = 7.3, 1.1 Hz, 1H), 6.94 – 6.81 (m, 3H), 6.03 (s, 2H), 5.94 (t, *J* = 54.5 Hz, 1H), 3.89 (s, 3H).

^{13}C NMR (151 MHz, $CDCl_3$) δ: 181.0 (t, *J* = 24.6 Hz), 147.1, 146.6, 138.9 (t, *J* = 6.5 Hz), 138.4, 136.9, 136.7, 125.5, 124.1, 123.8, 122.1, 112.1 (d, *J* = 254.7 Hz), 111.4, 109.4, 108.9, 107.6, 101.0, 34.0.

^{19}F NMR (376 MHz, $CDCl_3$) δ: -118.6 (d, *J* = 54.3 Hz, 2F).

HRMS (ESI+) calc: [M+Na$^+$] ($C_{18}H_{13}NaNO_3F_2$) 352.0756; measured: 352.0761 = 1.4 ppm difference.

IR (neat) vmax/ cm^{-1}: 3675, 2957, 2901, 1279, 1075.

^{1}H NMR (400 MHz, $CDCl_3$):

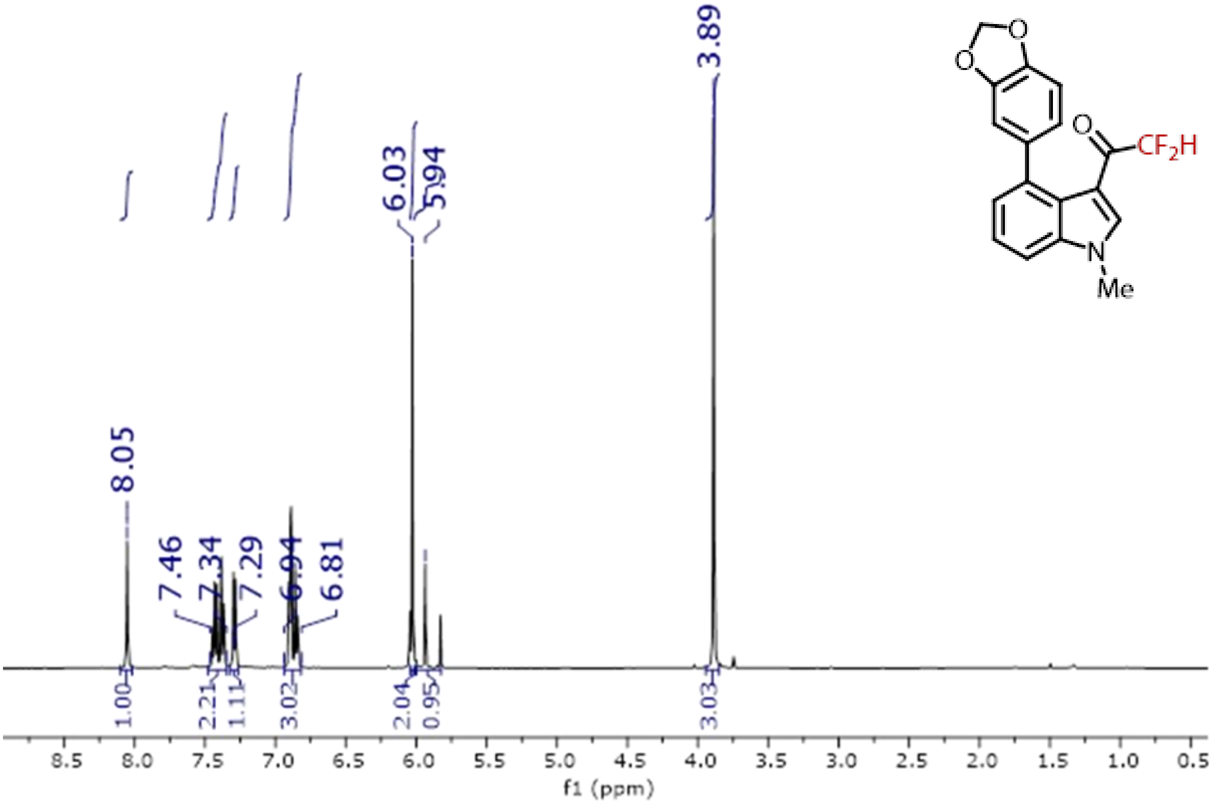

^{13}C NMR (131 MHz, $CDCl_3$):

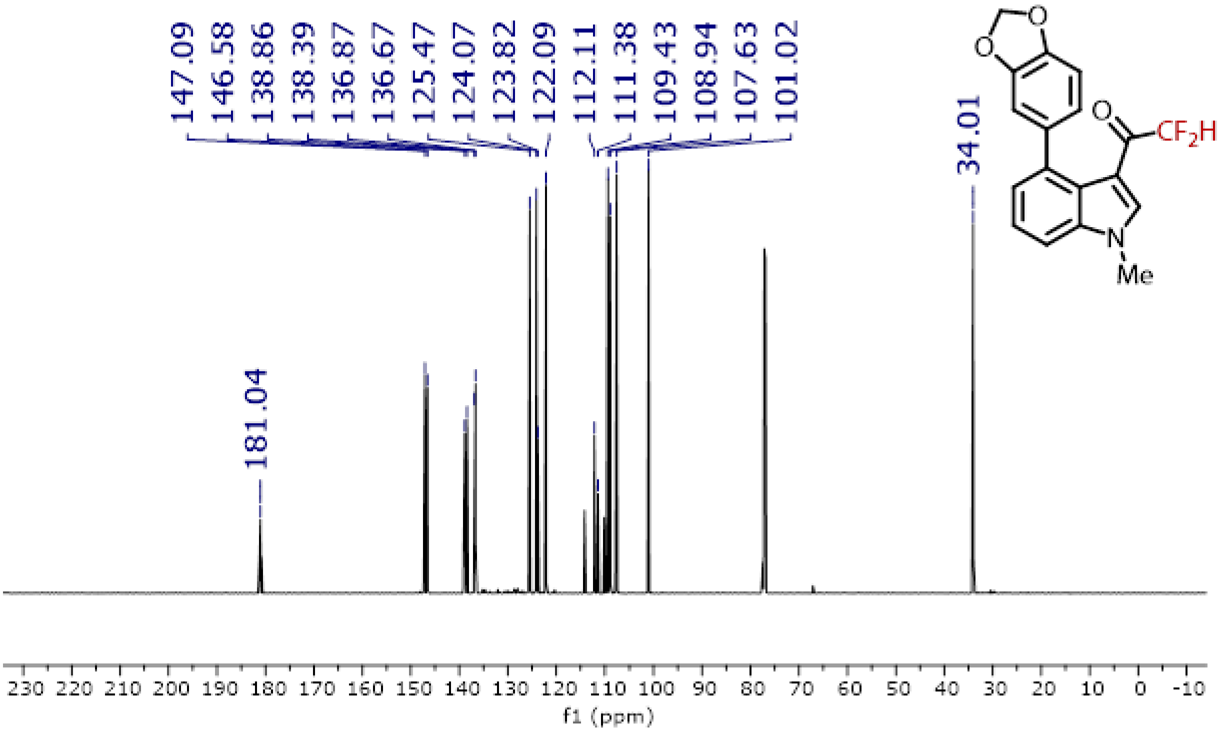

^{19}F NMR (376 MHz, $CDCl_3$):

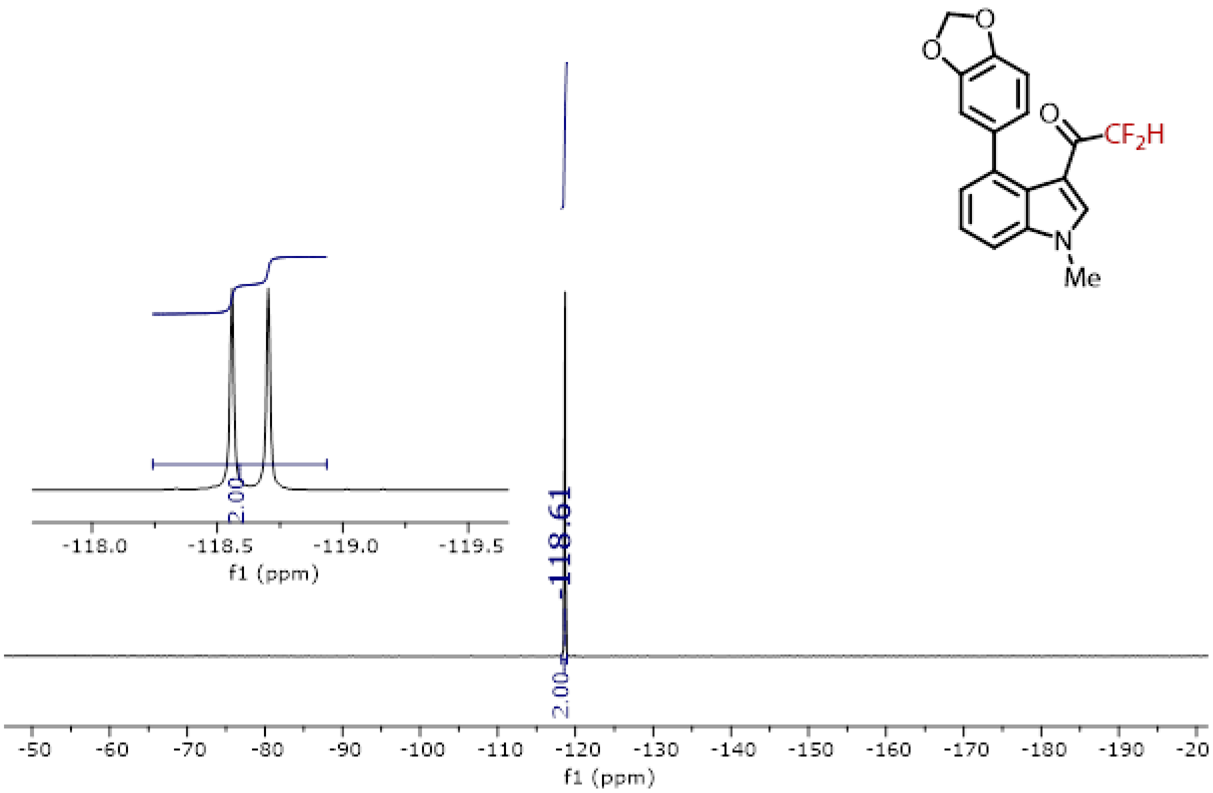

<u>Trifluoromethylation</u>

2-(4-bromo-1-methyl-1H-indol-3-yl)-1,1,1,3,3-pentafluoropropan-2-ol, **10**

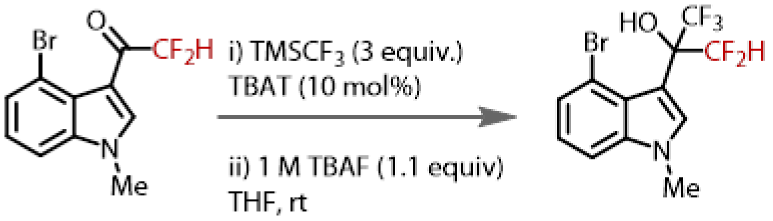

In a 7 mL vial, under air, was added **3ae** (288 mg, 1.0 mmol, 1.0 equiv.) and $TMSCF_3$ (0.44 mL, 3.0 mmol, 3.0 equiv.). TBAT (54 mg, 0.1 mmol, 10 mol%) was added and the vial capped quickly (caution: exotherm). The solution was stirred for 30 minutes at which time TLC analysis (50% Et_2O in Hexane) showed complete consumption of the starting material. To a portion (188 mg, 0.44 mmol) of the crude residue was added THF (3 mL) and TBAF (1 M in THF, 0.48 mL, 0.48 mmol, 1.1 equiv.). The solution was stirred for 1 hour and the crude residue concentrated onto silica and purified by chromatography on silica gel (12% Et_2O in Hexane to 100% Et_2O in Hexane) to yield the title material as a white solid (149 mg, 54% over two steps).

$\mathbf{R_f}$ = 0.2 (40% Et_2O in Hexane)

^{1}H NMR (400 MHz, $CDCl_3$) δ: 7.58 – 7.44 (m, 2H), 7.36 (dd, *J* = 8.3, 1.0 Hz, 1H), 7.14 (dd, *J* = 8.3, 7.6 Hz, 1H), 6.55 (t, *J* = 54.4 Hz, 1H), 4.58 (s, 1H), 3.81 (s, 3H).

^{13}C NMR (151 MHz, $CDCl_3$) δ: 139.0, 131.3, 126.6, 125.5, 124.1 (q, *J* = 288.5 Hz), 123.2, 114.3 (t, *J* = 252.5 Hz), 112.1, 109.8, 104.0, 76.4 – 75.1 (m), 33.7.

^{19}F NMR (376 MHz, $CDCl_3$) δ: -74.1 (t, *J* = 10.2 Hz, 3F), -127.1 (dd, *J* = 285.8, 54.8 Hz, 1F), -130.7 (ddq, *J* = 284.9, 54.1, 9.1 Hz, 1F).

HRMS (ESI+) calc: [$M+Na^+$] ($C_{12}H_9{}^{79}BrNNaOF_5$) 379.9680; measured: 379.9689 = 2.3 ppm difference.

IR (neat) vmax/ cm^{-1}: 3460, 2940, 1470, 1168, 922.

[1]**H NMR** (400 MHz, $CDCl_3$):

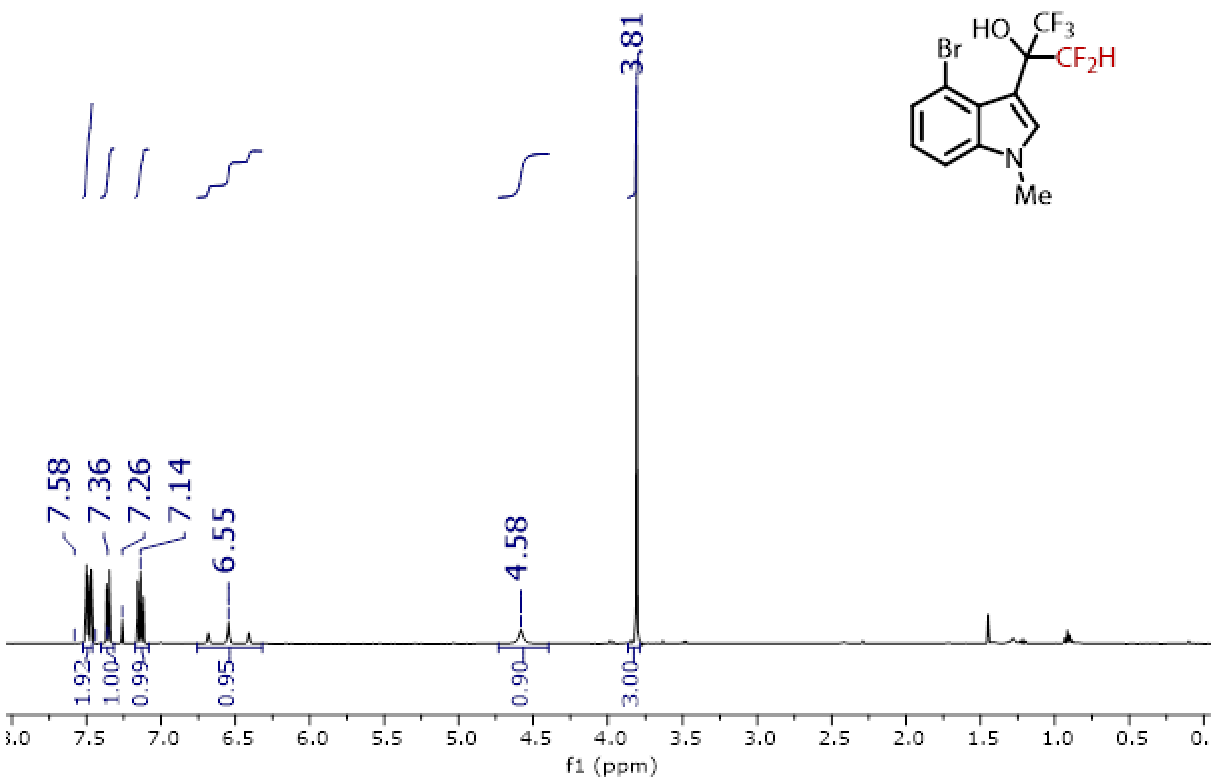

[13]**C NMR** (131 MHz, $CDCl_3$):

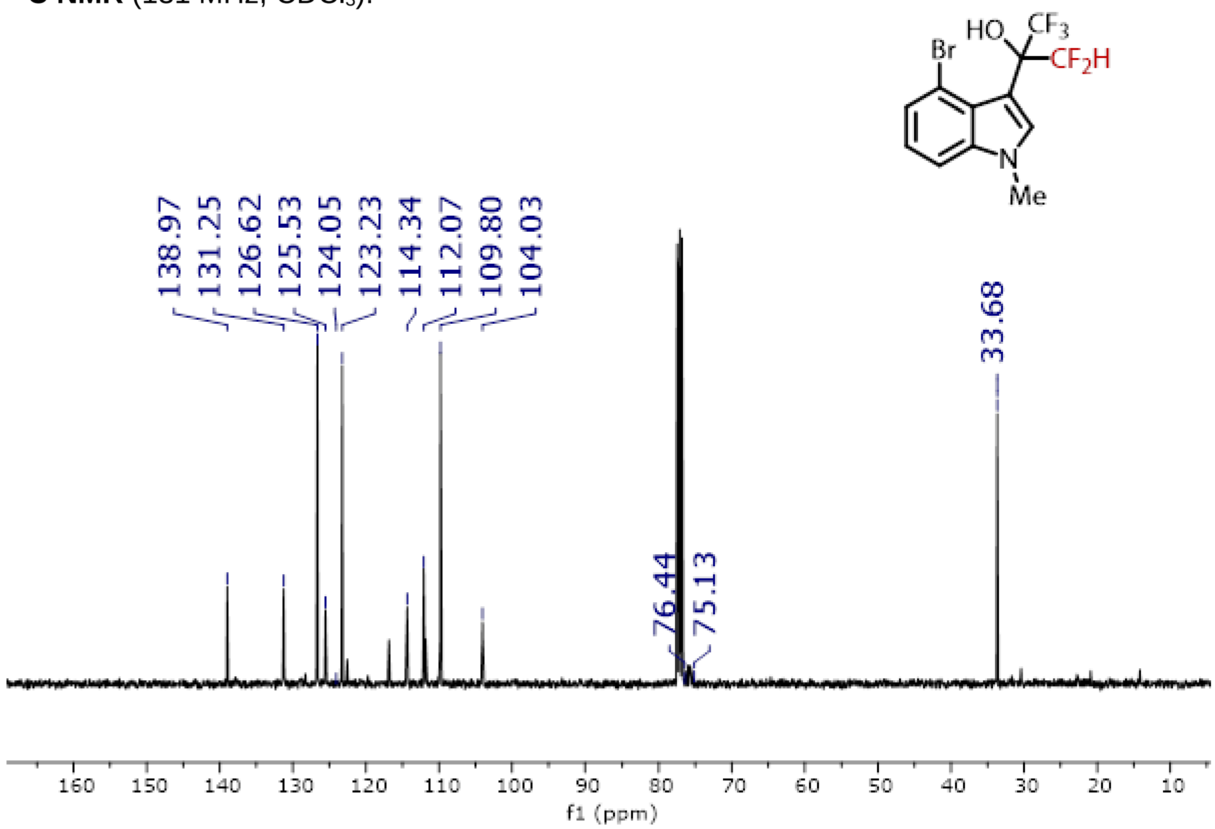

^{19}F NMR (376 MHz, $CDCl_3$):

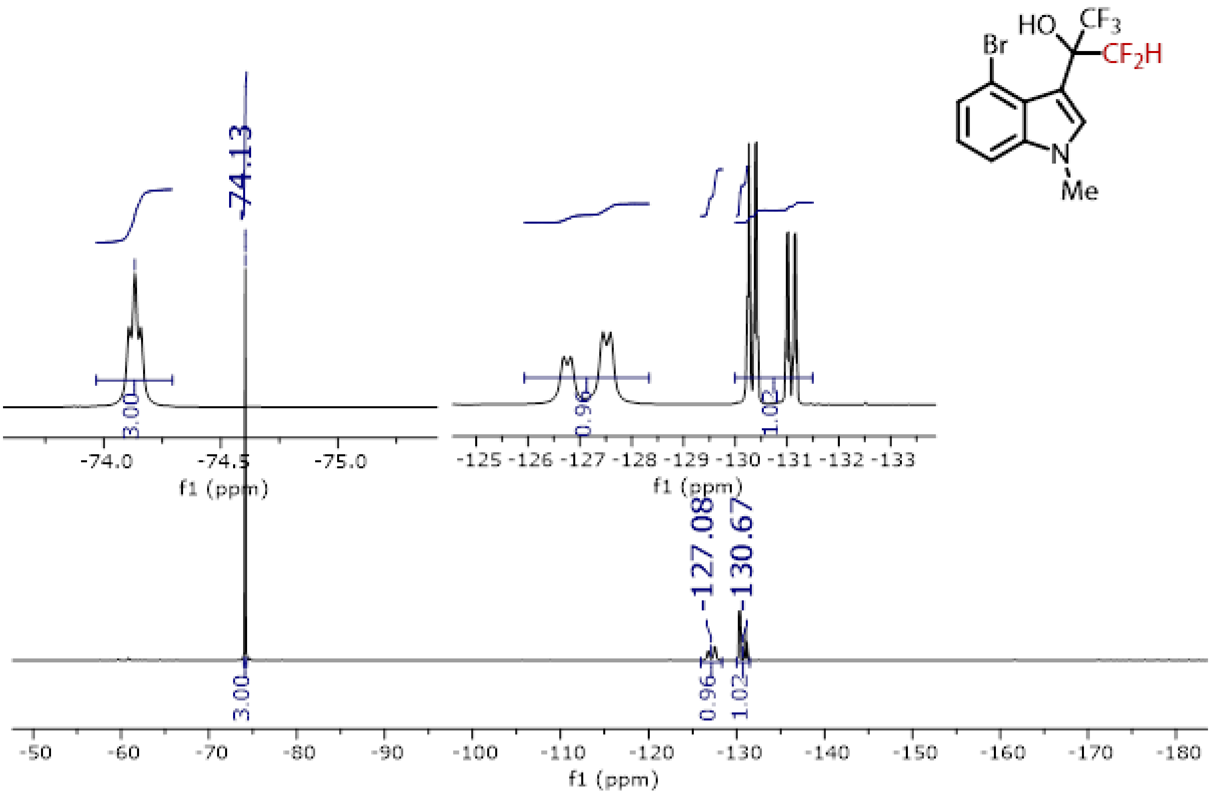

Reductive methoxylation

4-bromo-3-(2,2-difluoro-1-methoxyethyl)-1-methyl-1H-indole, **5**

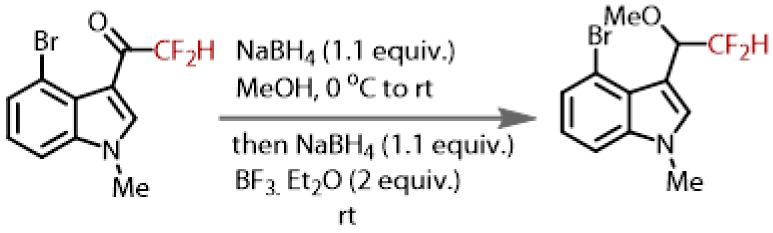

In a 7 mL vial, under air, $NaBH_4$ (41.6 mg, 1.1 mmol, 1.1 equiv.) was added in one portion to a MeOH (2.5 mL) solution of **3ae** (288 mg, 1.0 mmol, 1 equiv.) at 0°C. The vial was capped and allowed to warm to RT with stirring over 1 hour. $BF_3 \cdot Et_2O$ (0.25 mL, 2 mmol, 2 equiv.) was added and the solution turned purple, a second portion of $NaBH_4$ (41.6 mg, 1.1 mmol, 1.1 equiv.) was added and the solution stirred for 30 minutes. The crude material was concentrated directly onto silica and purified by chromatography on silica gel (2% Et_2O in Hexane to 18% Et_2O in Hexane) to yield the title material as a colourless solid (159 mg, 65%).

R_f = 0.3 (20% Et_2O in Hexane)

^{1}H NMR (400 MHz, $CDCl_3$) δ: 7.37 – 7.27 (m, 3H), 7.09 (dd, *J* = 8.2, 7.6 Hz, 1H), 6.05 (td, *J* = 55.5, 3.2 Hz, 1H), 5.77 – 5.69 (m, 1H), 3.79 (s, 3H), 3.52 (s, 3H).

^{13}C NMR (151 MHz, $CDCl_3$) δ: 138.2, 130.3, 125.9, 124.7, 122.7, 115.6 (t, *J* = 246.0 Hz), 113.5, 109.2, 108.7 (t, *J* = 3.8 Hz), 74.6 (t, *J* = 23.1 Hz), 57.8, 33.4.

^{19}F NMR (376 MHz, $CDCl_3$) δ: -125.7 (ddd, *J* = 279.5, 55.6, 9.7 Hz, 1F), -129.2 (ddd, *J* = 279.3, 55.4, 13.0 Hz, 1F).

HRMS (ESI+) calc: [M+Na$^+$] ($C_{12}H_{12}NaNO^{79}BrF_2$) 325.9963; measured: 325.9953 = 2.9 ppm difference.

IR (neat) νmax/ cm^{-1}: 2967, 2936, 1542, 1055, 737.

^{1}H NMR (400 MHz, $CDCl_3$):

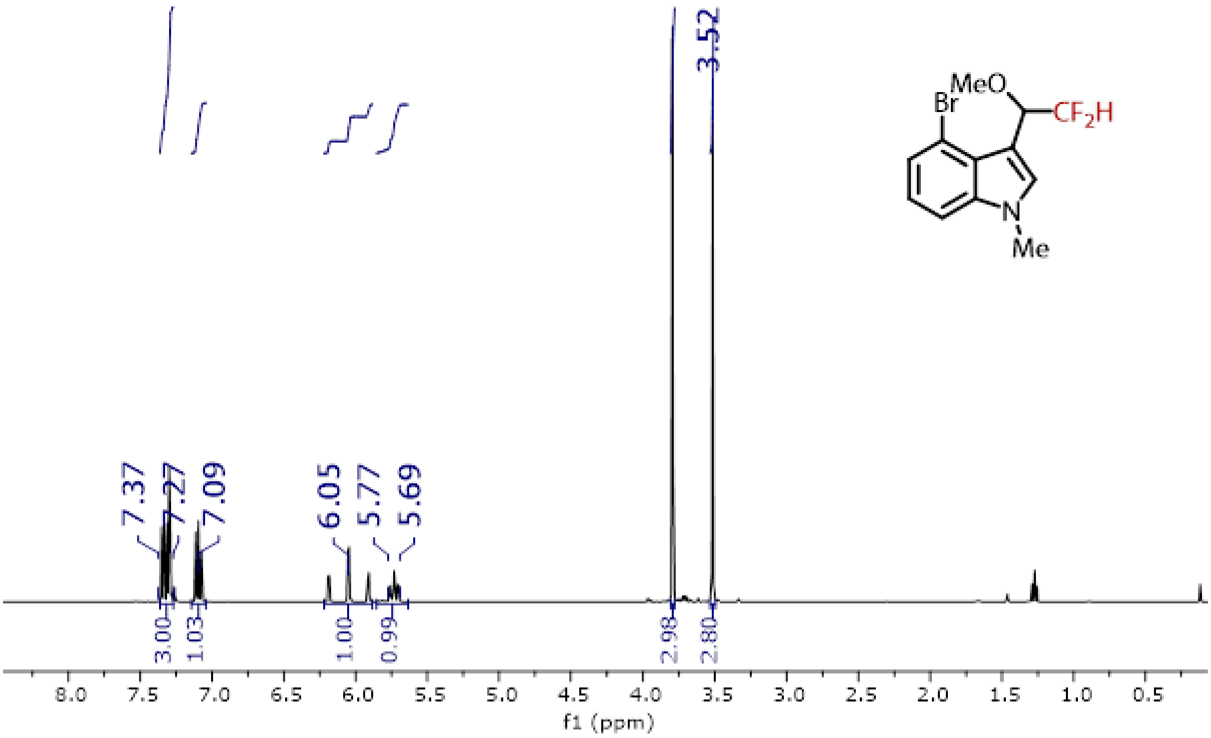

^{13}C NMR (131 MHz, $CDCl_3$):

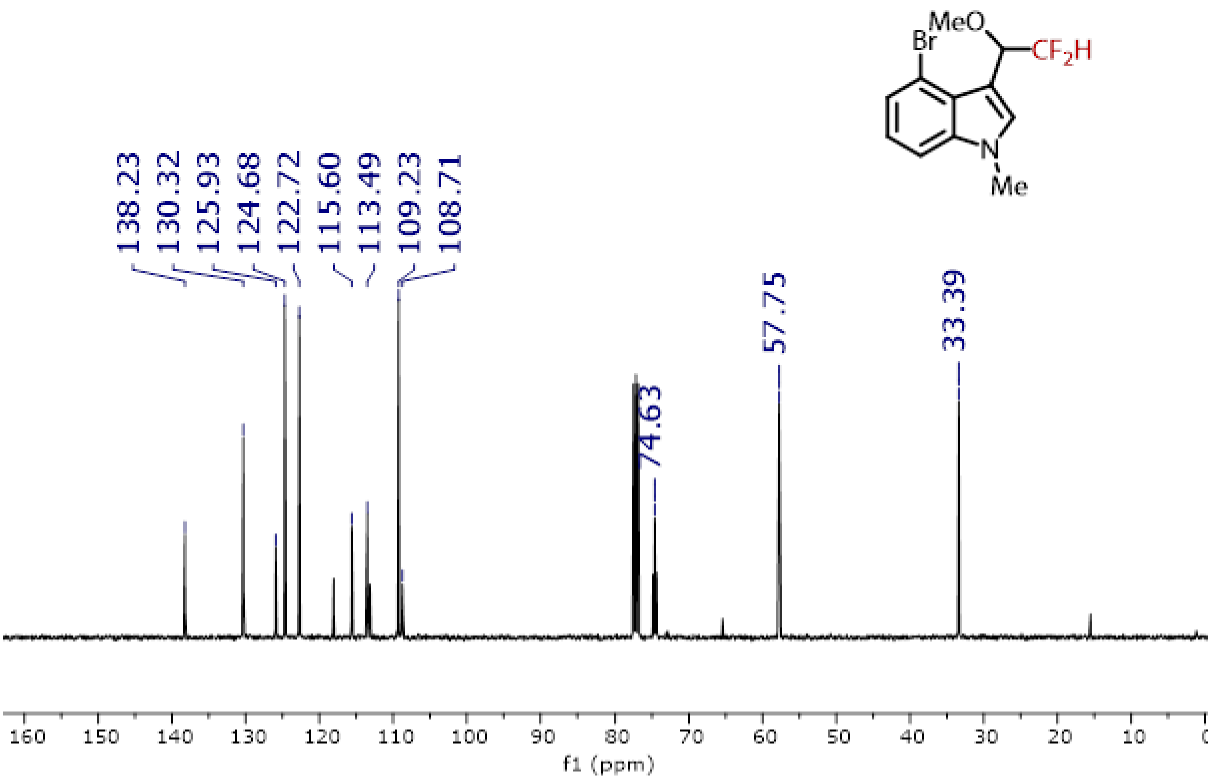

^{19}F NMR (376 MHz, $CDCl_3$):

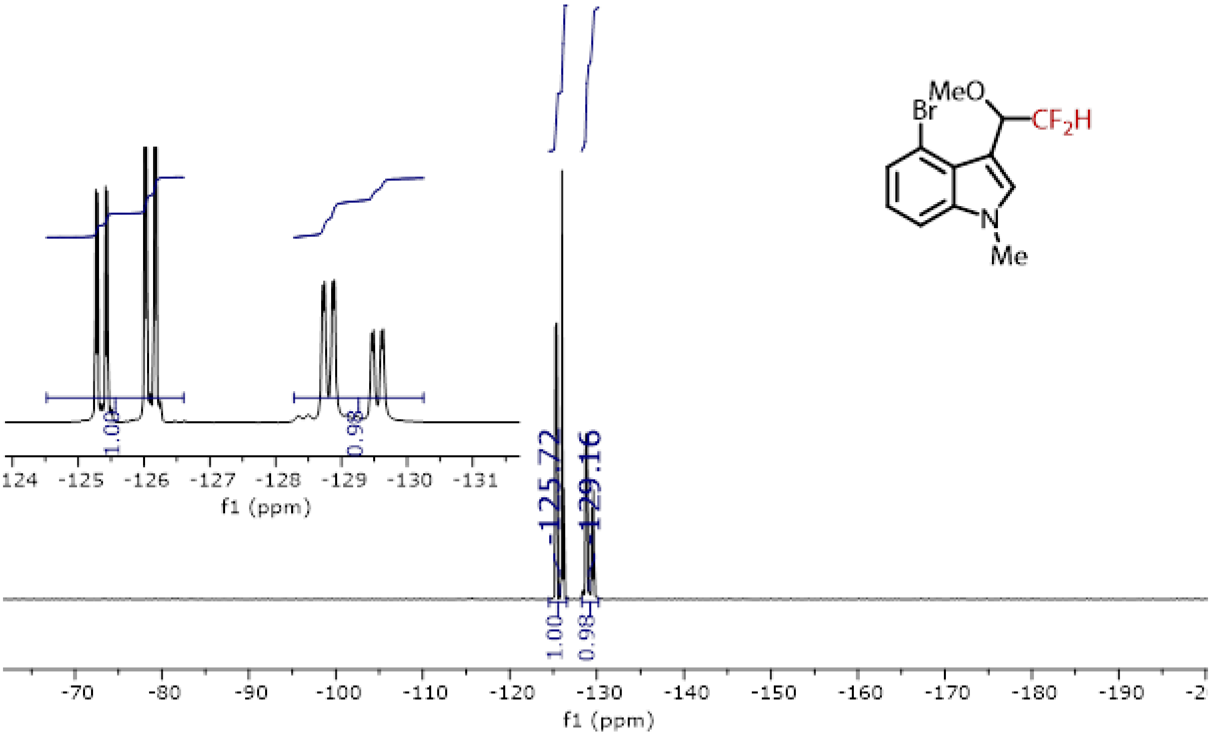

Hydrodefluorination

2-fluoro-1-(1-methyl-1H-indol-3-yl)ethan-1-one, **4**

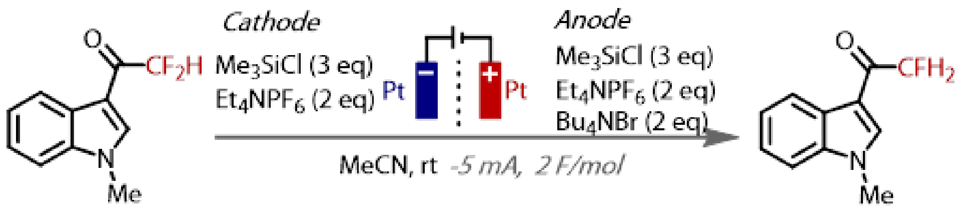

Subjecting **3a** to **General Procedure D** yielded **4** after purification through chromatography on silica gel (57% Et_2O in Hexane to 100% Et_2O in Hexane) as a yellow solid that turned red on standing (89.9 mg, 94%).

R$_f$ = 0.2 (40% Et_2O in Hexane)

^{1}H NMR (400 MHz, $CDCl_3$) δ: 8.41 (ddt, *J* = 6.0, 3.7, 1.9 Hz, 1H), 7.99 (d, *J* = 2.1 Hz, 1H), 7.39 – 7.31 (m, 3H), 5.22 (d, *J* = 47.7 Hz, 2H), 3.86 (s, 3H).

^{13}C NMR (151 MHz, $CDCl_3$) δ: 189.8 (d, *J* = 17.9 Hz), 137.1, 136.9, 126.8, 123.9, 123.2, 122.6, 113.0, 109.8, 85.4 (d, *J* = 184.9 Hz), 33.8.

^{19}F NMR (376 MHz, $CDCl_3$) δ: -222.4 (t, *J* = 47.7 Hz, 1F).

Spectral data in accordance with literature.[22]

Olefination

4-bromo-3-(1-(4-bromophenyl)-3,3-difluoroprop-1-en-2-yl)-1-methyl-1H-indole, **9**

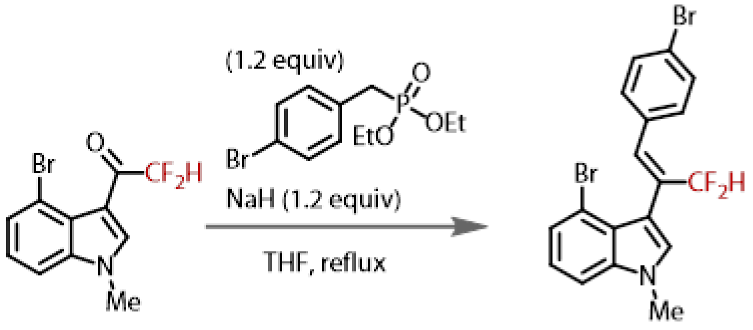

To a 25 mL round-bottomed flask was added **3ae** (72.0 mg, 0.25 mmol, 1.0 equiv.), diethyl (4-bromobenzyl)phosphonate (92.1 mg, 0.3 mmol, 1.2 equiv.) and THF (3 mL). The reaction mixture was allowed to stir and NaH (10.1 mg, 0.3 mmol, 1.2 equiv., 60% dispersion in mineral oil) was added in one portion. The reaction mixture was refluxed until complete by TLC (20% EtOAc in Hexane). After allowing the reaction mixture to cool to RT, the solvent was removed *in vacuo* (40 °C, 100 mBar) and the resulting crude mixture purified by flash column chromatography (25% EtOAC in Hexane) to afford the title compound as a colourless solid (62.4 mg, 57%)

R_f = 0.2 (25% EtOAc in Hexane)

^{1}H NMR (500 MHz, $CDCl_3$) δ: 7.37 – 7.21 (m, 4H), 7.12 (t, *J* = 7.9 Hz, 1H), 7.08 (s, 1H), 7.01 (d, *J* = 8.3 Hz, 2H), 6.97 (s, 1H), 6.45 (t, *J* = 56.7 Hz, 1H), 3.81 (s, 3H).

^{13}C NMR (126 MHz, $CDCl_3$) δ: 138.0, 134.3, 133.7 (d, *J* = 9.2 Hz), 131.4, 130.9, 129.5, 128.5 (d, *J* = 17.6 Hz), 125.3 (t, *J* = 223.6 Hz), 123.0, 117.8, 115.9 (d, *J* = 3.6 Hz), 114.3, 114.0, 109.0, 107.1 (t, *J* = 2.2 Hz), 33.3.

^{19}F NMR (376 MHz, $CDCl_3$) δ: -112.7 (dd, *J* = 284.8, 56.7 Hz, 1F), -116.5 (dd, *J* = 284.9, 56.8 Hz, 1F).

HRMS (APCI+) calc: [M+Na$^+$] ($C_{18}H_{13}N^{79}Br_2F_2$) 439.9456; measured: 439.9452, 0.9 ppm difference.

IR (neat) vmax/ cm^{-1}: 2922, 2852, 2033, 1982, 1963, 1462, 1066, 588

^{1}H NMR (500 MHz, $CDCl_3$):

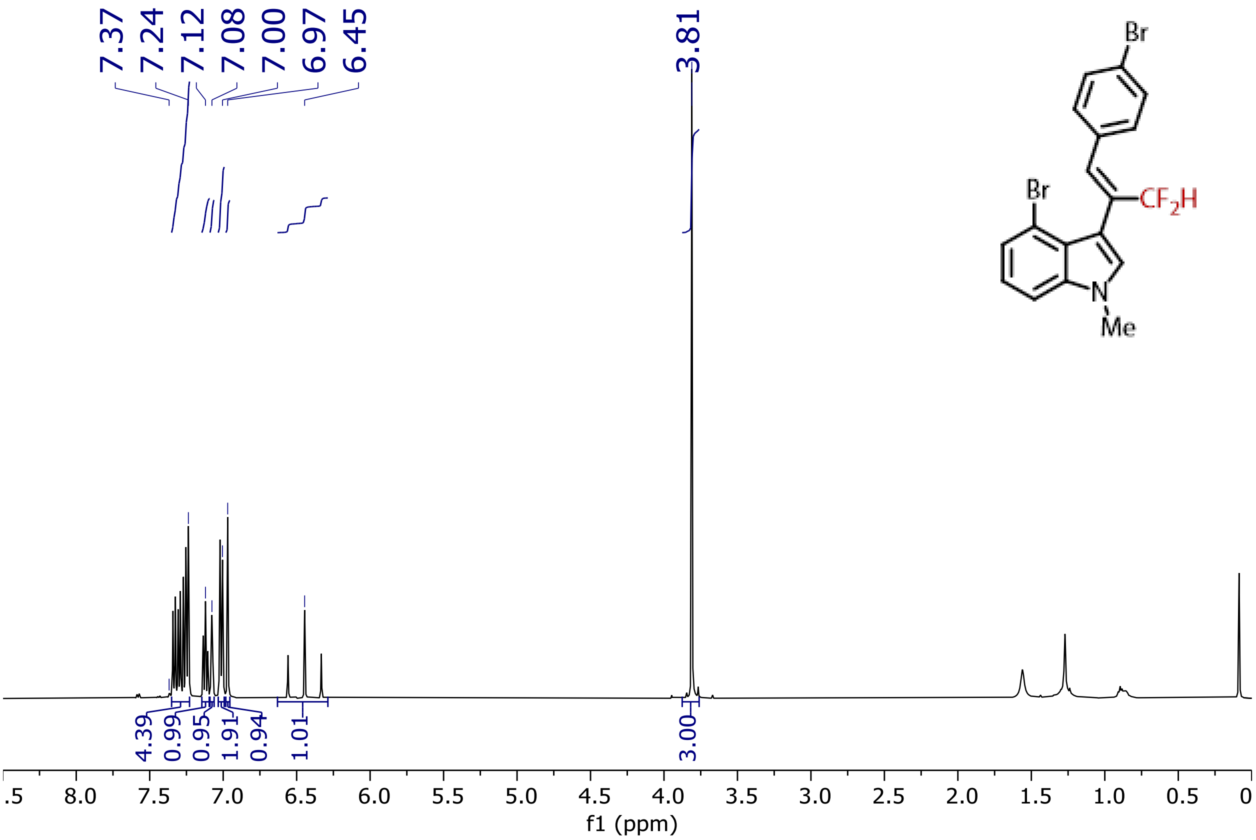

^{13}C NMR (126 MHz, $CDCl_3$):

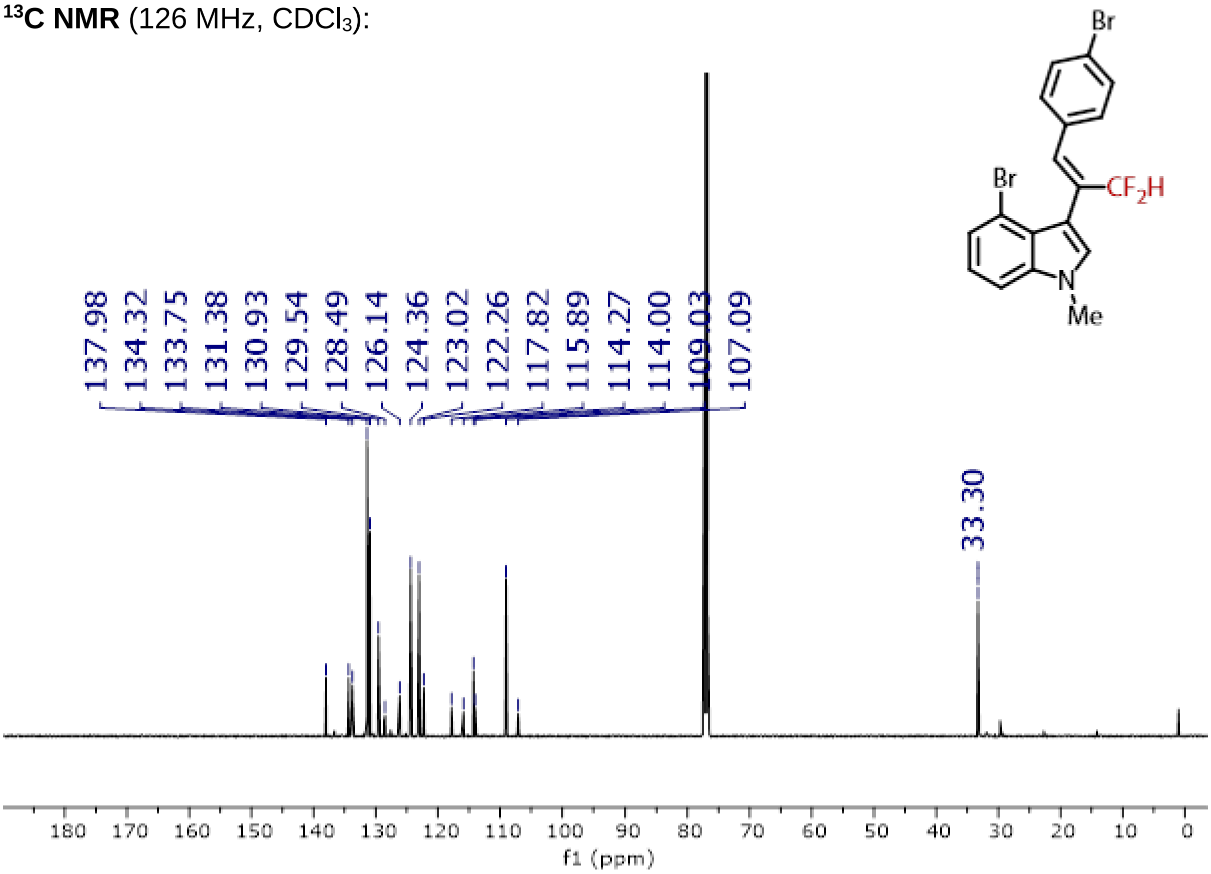

^{19}F NMR (376 MHz, $CDCl_3$):

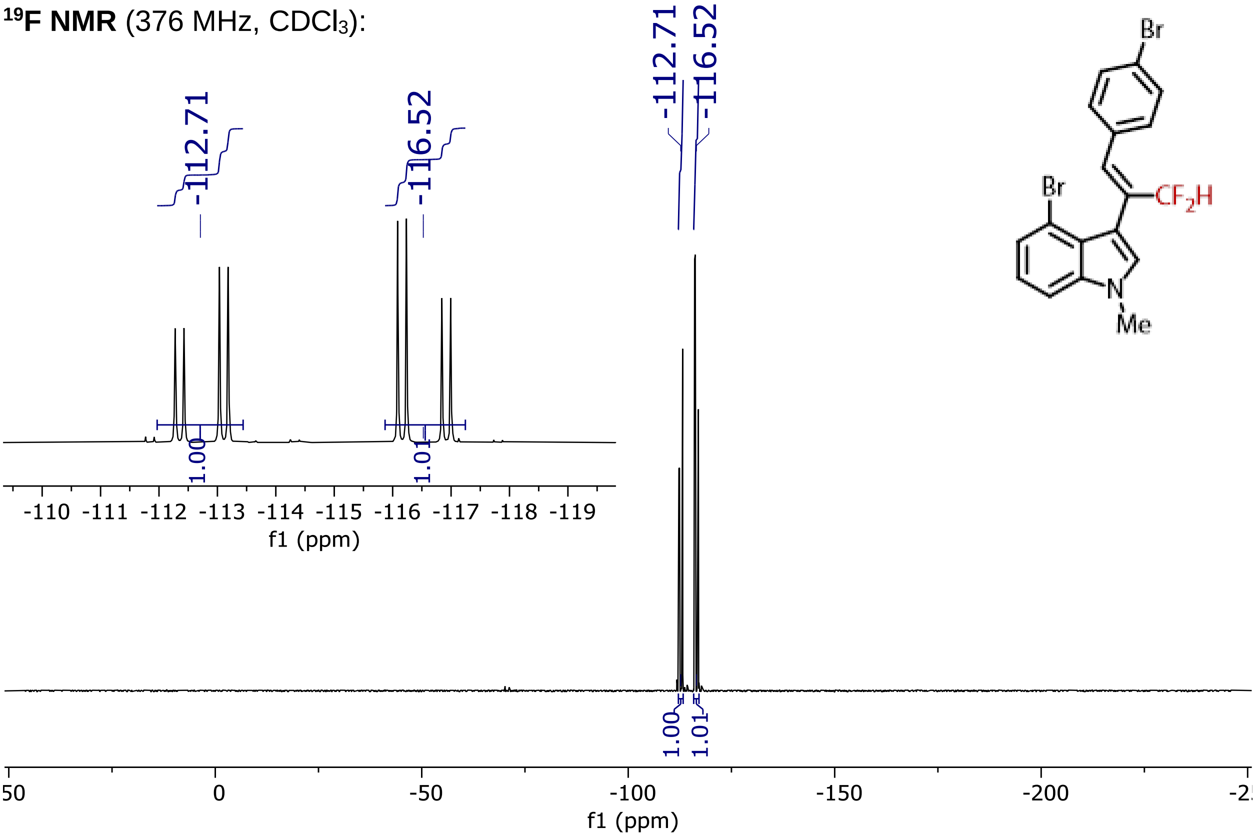

Dithiolation

3-(2-(difluoromethyl)-1,3-dithiolan-2-yl)-1-methyl-1H-indole, **7**

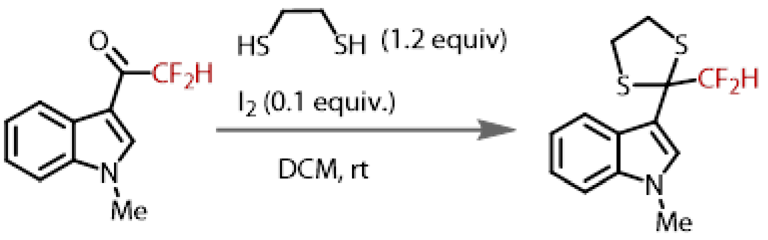

To a 20 mL vial, under air, was added **3a** (428 mg, 2 mmol, 1 equiv.) and CH_2Cl_2 (5 mL). 1,2-ethandithiol (0.22 mL, 2.4 mmol, 1.2 equiv.) and iodine (53 mg, 0.2 mmol, 0.1 equiv.) sequentially and the purple solution was allowed to stir overnight. The solution was concentrated directly onto silica and purified by chromatography on silica gel (7% Et_2O in pentane to 60% Et_2O in pentane) to yield the title material as a colourless solid (429 mg, 82%).

R_f = 0.3 (30% Et_2O in pentane)

^{1}H NMR (400 MHz, $CDCl_3$) δ: 7.92 (dq, *J* = 8.0, 0.9 Hz, 1H), 7.35 – 7.25 (m, 3H), 7.19 (ddd, *J* = 8.1, 6.7, 1.5 Hz, 1H), 6.22 (t, *J* = 57.4 Hz, 1H), 3.75 (s, 3H), 3.50 (t, *J* = 1.2 Hz, 4H).

^{13}C NMR (101 MHz, $CDCl_3$) δ: 137.9, 128.8, 126.2, 122.3, 121.2, 119.7, 116.6 (t, *J* = 250.9 Hz), 110.4, 109.9, 68.4 (t, *J* = 21.8 Hz), 40.1, 33.0.

^{19}F NMR (376 MHz, $CDCl_3$) δ: -114.2 (d, *J* = 57.4 Hz, 2F).

HRMS (ESI+) calc: [$M+Na^+$] ($C_{13}H_{13}NaNF_2S_2$) 308.0350; measured: 308.0360 = -3.4 ppm difference.

IR (neat) vmax/ cm^{-1}: 2928, 1529, 1066, 890, 738, 629.

^{1}**H NMR** (400 MHz, $CDCl_3$):

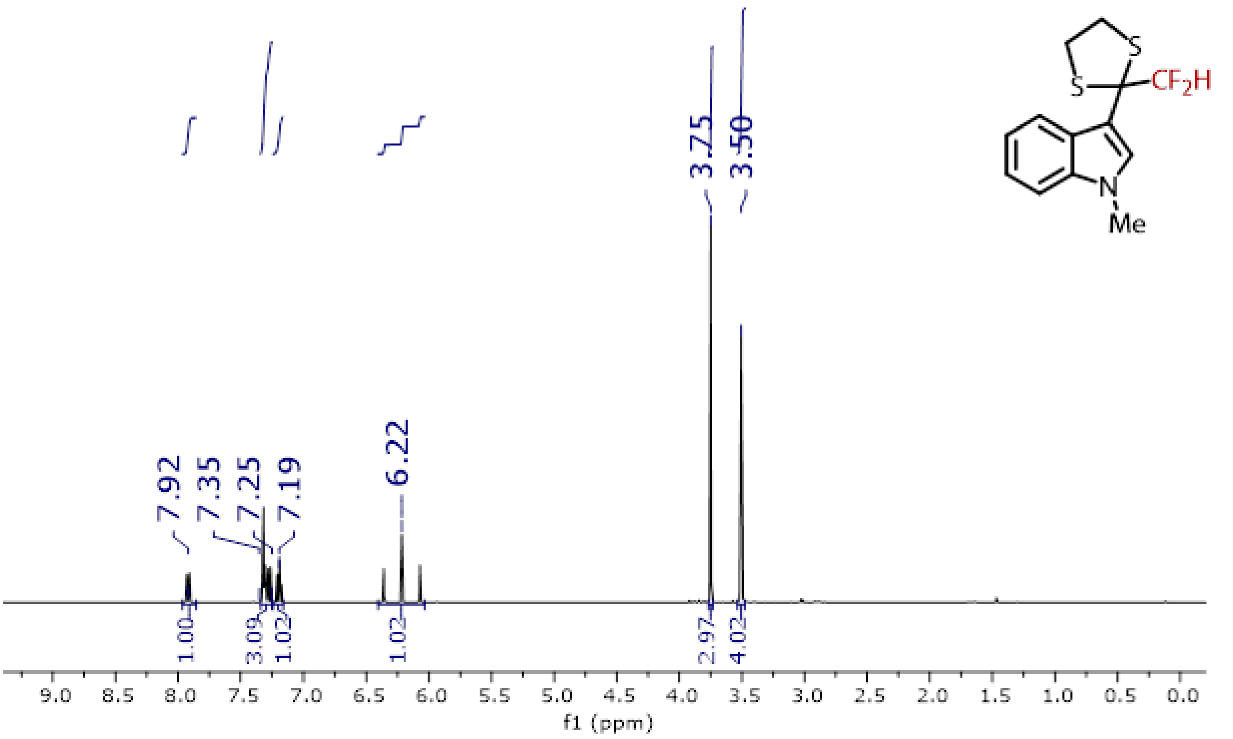

^{13}**C NMR** (131 MHz, $CDCl_3$):

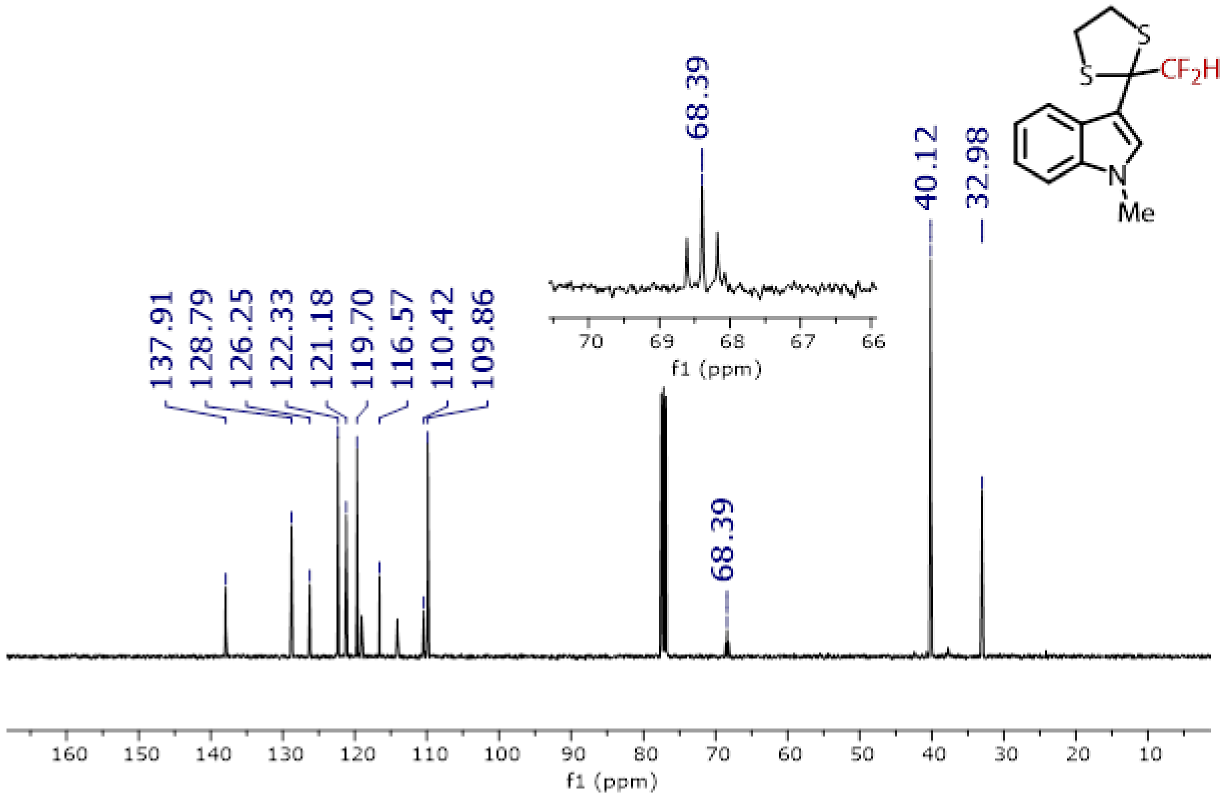

^{19}F NMR (376 MHz, $CDCl_3$):

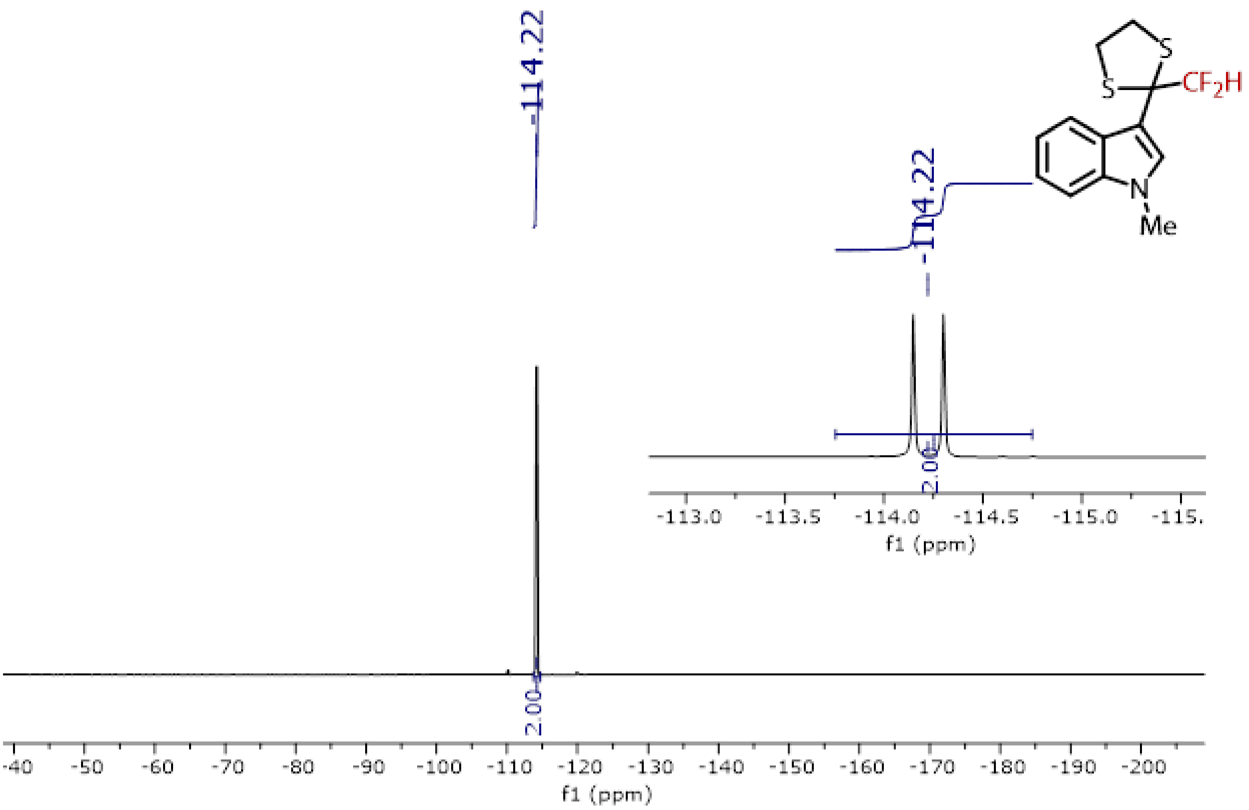

Methenylation

3-(2-(difluoromethyl)oxiran-2-yl)-1-methyl-1H-indole, **8**

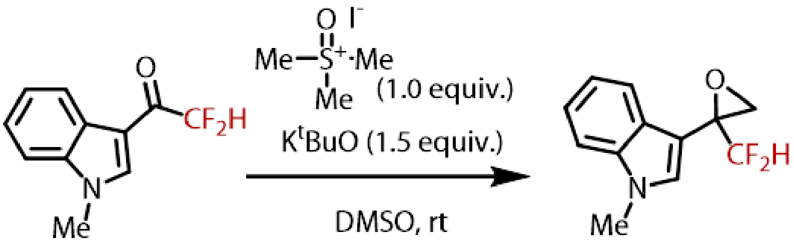

Under N_2, DMSO (3 mL) was added to K^{t}BuO (57 mg, 0.75 mmol, 1.5 equiv.). Trimethylsulfoxonium iodide (137 mg, 0.5 mmol, 1.0 equiv.) was added and the resulting solution was stirred for 30 minutes. **3a** (105 mg, 0.5 mmol, 1.0 equiv.) was added as a solution in DMSO (1 mL) and the resulting solutions stirred overnight. Et_2O was added along with H_2O and the layers separated. The aqueous layer was extracted once more with Et_2O and the organic extracts dried with $MgSO_4$ and concentrated *in vacuo* (100 mBar, 40°C). The crude material was subjected to Chromatography on silica gel (100% Et_2O) quickly to yield the title material as a yellow oil (76 mg, 68%). Note: **8** was observed to be unstable when stored at RT both neat and as a solution in $CDCl_3$. As such, ^{13}C NMR data was not obtainable.

R_f = 0.1 (100% Et_2O)

^{1}H NMR (400 MHz, $CDCl_3$) δ: 7.82 (ddt, *J* = 8.1, 1.1, 0.7 Hz, 1H), 7.36 – 7.20 (m, 2H), 7.16 – 7.07 (m, 2H), 6.06 (t, *J* = 56.1 Hz, 1H), 4.18 – 4.03 (m, 2H), 3.76 (s, 3H).

^{19}F NMR (376 MHz, $CDCl_3$) δ: -128.6 (dd, *J* = 281.1, 56.1 Hz), -132.8 (ddd, *J* = 281.2, 56.0, 2.3 Hz).

HRMS (APCI+) calc: [M+H$^+$] ($C_{12}H_{11}NOF_2$) 224.0881; measured: 224.0880 = 0.5 ppm difference.

IR (neat) vmax/ cm^{-1}: 2543, 1343, 1210, 934, 875, 764.

^{1}H NMR (400 MHz, $CDCl_3$):

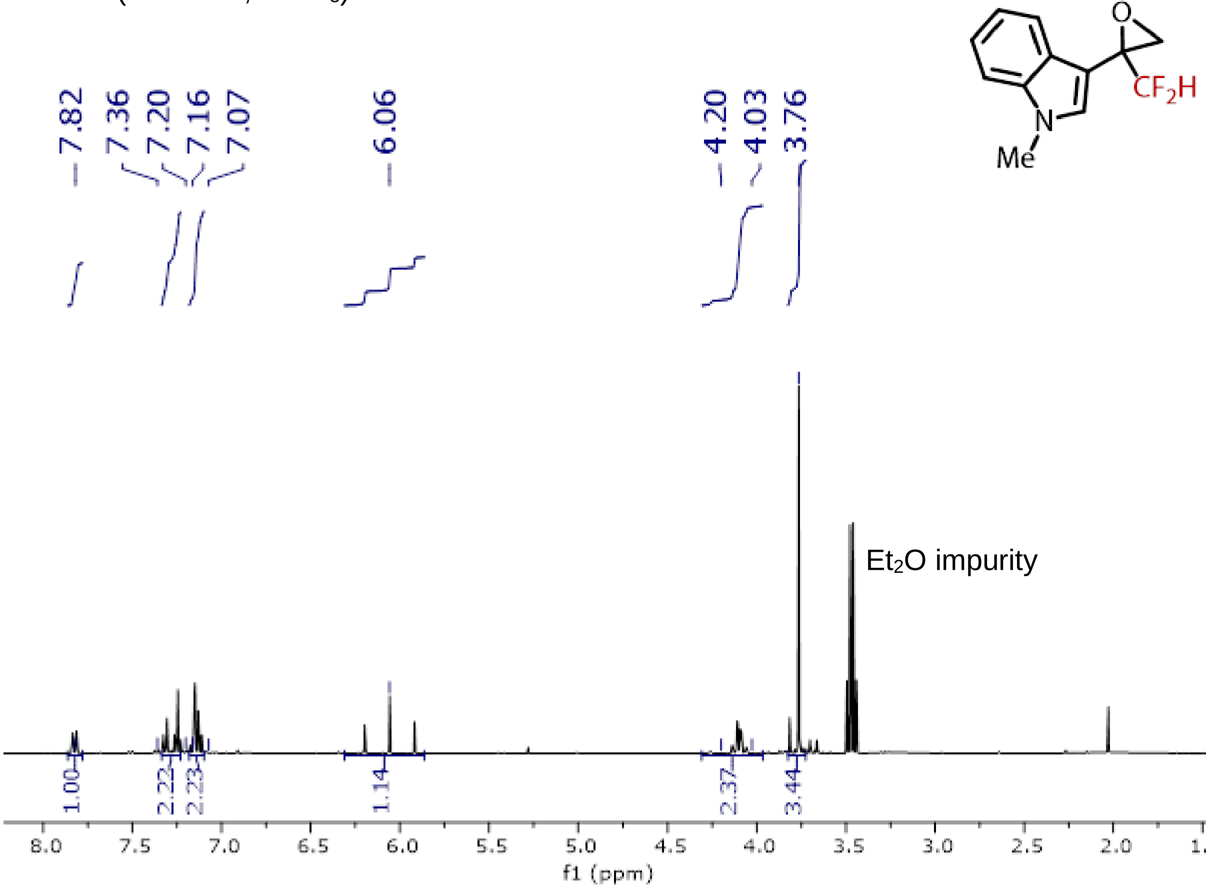

^{19}F NMR (376 MHz, $CDCl_3$):

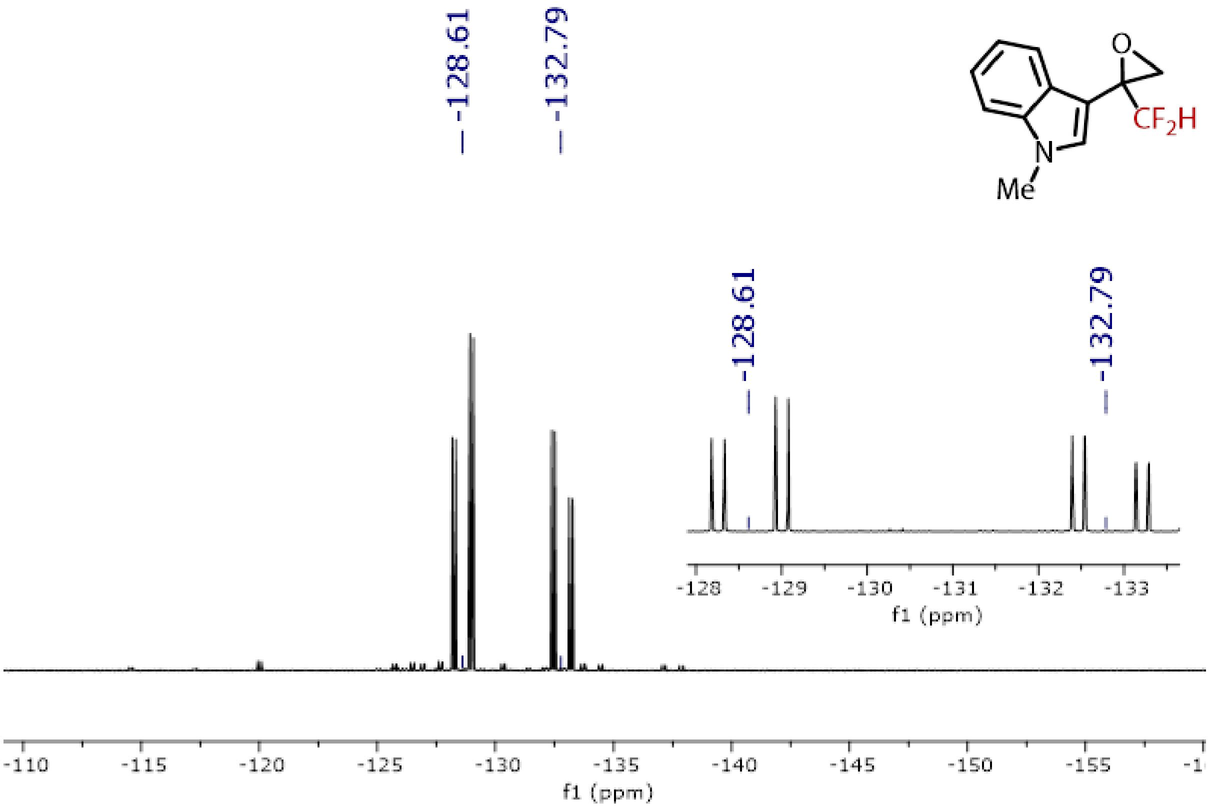

Determination of H-bonding strength (A value)

The solute hydrogen-bond (HB) acidity (*A*) of has been previously calculated by Sessler[23] using the spectroscopic technique outlined by Abraham.[24] By comparison of the ^{1}H NMR chemical shifts of the proton in question in both chloroform and dimethylsulfoxide (DMSO), *A* can be calculated using $A = 0.0065 + 0.133\Delta\delta$ where $\Delta\delta = \delta\,(DMSO) - \delta\,(CDCl_3)$.

Solutions of the compound of interest (10 mg/mL) were prepared in both $CDCl_3$ and DMSO-d_6 ensuring complete dissolution via sonication and vortex mixing at room temperature. ^{1}H NMR spectra were taken at the same temperature one after each other. Peaks were referenced to residual solvent signals for $CDCl_3$ and DMSO-d_6 respectively and δ CF_2**H** measured from the centre of the triplet (see worked example for entry 5 from Table S2).

- Worked example of entry 5:

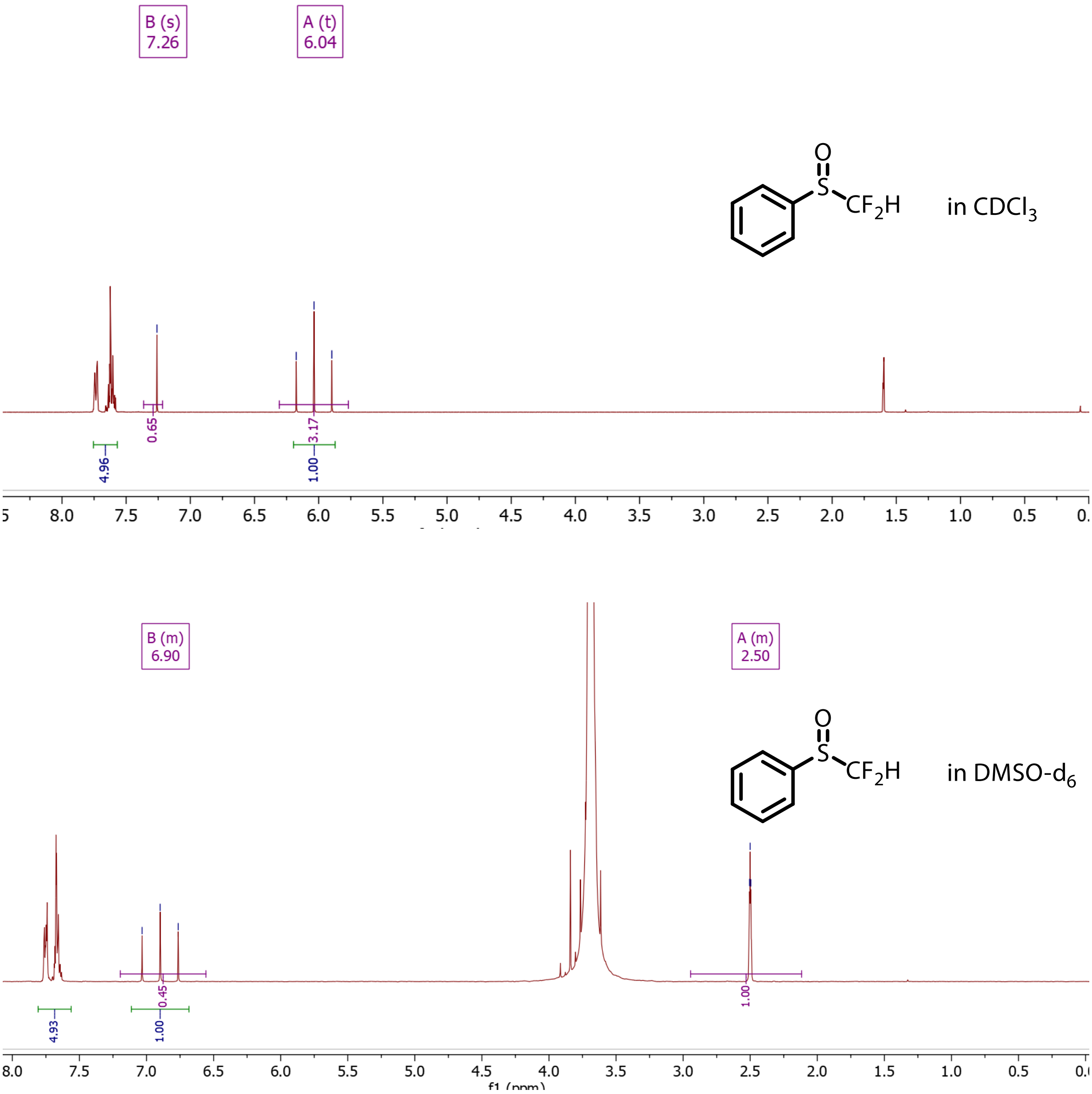

Figure S4. ^{1}H NMR spectra of difluoromethylphenyl sulfoxide in $CDCl_3$ (top) and DMSO-d_6 (bottom.)

Calculation:

δ CF_2**H** in $CDCl_3$ = 6.0363 ppm and δ CF_2H in DMSO-d_6 = 6.8980

Δδ = δ CF_2**H** in DMSO-d_6 - δ CF_2**H** in $CDCl_3$ = 6.8980 – 6.9363 = 0.8617

A = 0.0065 + 0.133Δδ = 0.0065 + (0.133 x 0.8617) = **0.121**

Compound	δ $CF_2\underline{\mathbf{H}}$		Δ	A_{NMR}
	$CDCl_3$	*DMSO-d_6*		
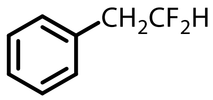	5.9253	6.2236	0.2983	**0.0462**
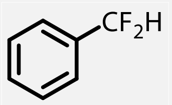	6.6508	7.0309	0.3801	**0.0571**
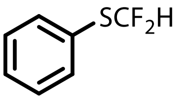	6.8339	7.4791	0.6452	**0.0923**
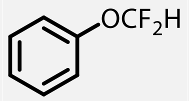	6.5145	7.1615	0.6470	**0.0926**
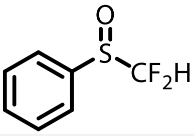	6.0363	6.8980	0.8617	**0.121**
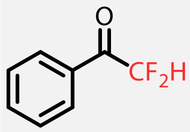	6.300	7.1700	0.8700	**0.122**
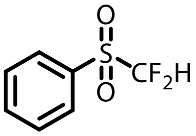	6.1919	7.3103	1.1184	**0.155**

Table S2. A values for a series of CF_2H-containing compounds.

Compound	δ $CF_2\underline{\mathbf{H}}$		Δ	A_{NMR}
	$CDCl_3$	*DMSO-d_6*		
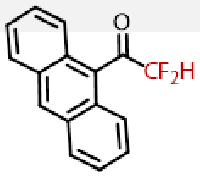	6.3437	7.0642	0.7205	**0.102**
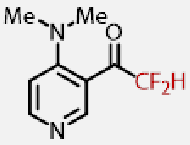	6.3530	7.1243	0.7713	**0.109**
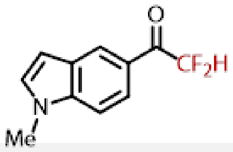	6.3996	7.2187	0.8191	**0.115**
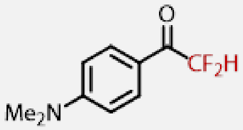	6.2568	7.0028	0.7460	**0.106**
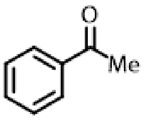	2.6126	2.5731	-0.0395	**0.00125**
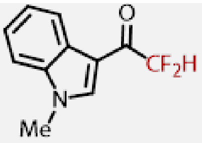	6.1094	6.7714	0.662	**0.0945**
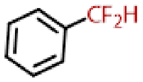	6.6508	7.0309	0.3801	**0.0571**

Table S3. A values for selected difluoromethyl ketones and other substrates.

Computational Studies

All DFT calculations were run using Gaussian 09W and Gaussview 5 software to the B3LYP/6-31G+p(d) level of theory. Conformational analysis was conducted using the scan functionality (dihedral angle as defined, increments of 20º) starting on the energy minimised structure of the desired molecule.

<u>Conformational analysis</u>

The energy of 4 acylated indoles containing an increasing number of fluorine atoms was calculated with progressive rotation around the OC-CH dihedral angle.

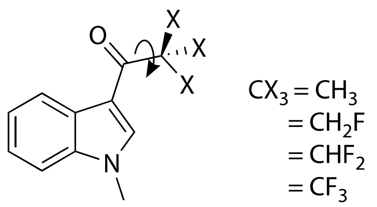

<u>$CX_3 = CH_3$</u>

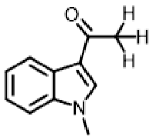

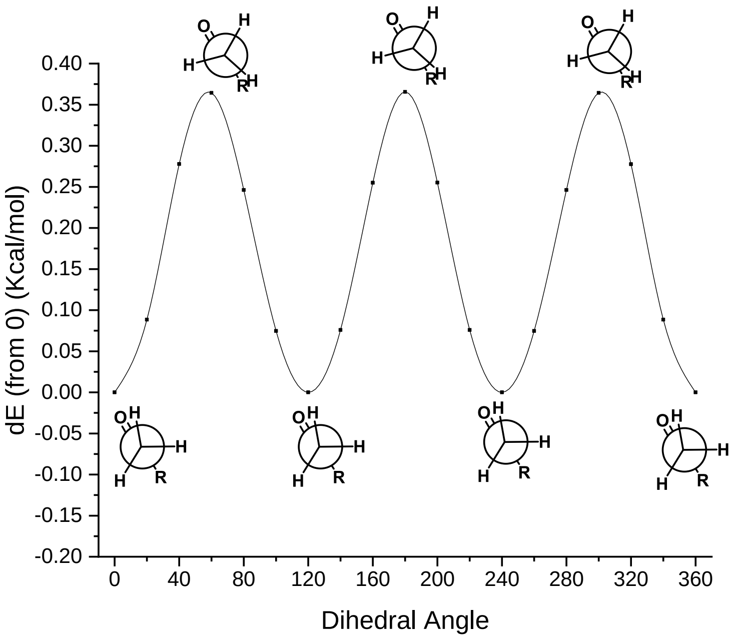

Figure S5. Graph of calculated ground state energies vs dihedral angle for 1-(1-methyl-1H-indol-3-yl)ethan-1-one.

$CX_3 = CF_3$

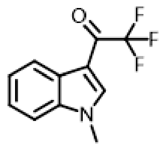

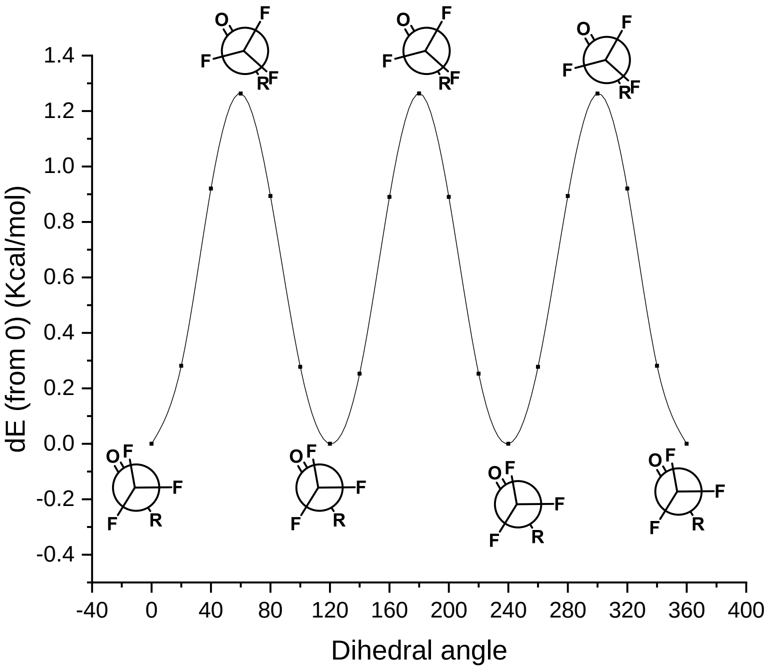

Figure S6. Graph of calculated ground state energies vs dihedral angle for **1a**.

For CH_3 and CF_3, conformations with eclipsing interactions between X and R lead to the highest energy conformers. This is due to unfavourable steric interactions between X and H-C(indole). The conformers with eclipsing interactions between X and O, led to the lowest energyy conformers, confirming this interaction is not destabilising.

$CX_3 = CFH_2$

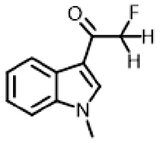

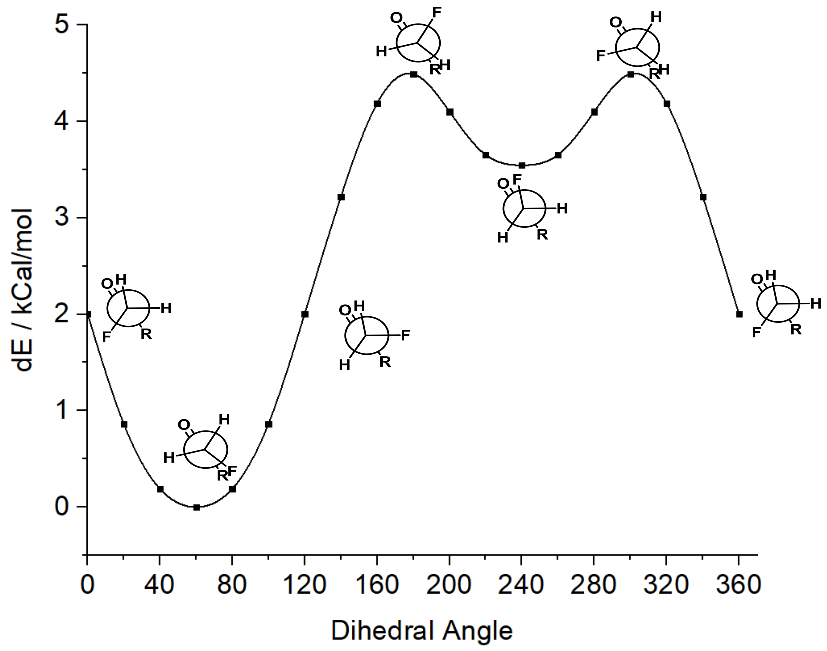

Figure S7. Graph of calculated ground state energies vs dihedral angle for **4**.

From the O-H eclipsed conformer (dihedral angle = 0) a decrease in energy occurs upon rotation toward the minimum at 60°; the conformer bearing the fluorine atom. Unlike the CF_3 and CH_3 compounds, the minimum energy conformer has an eclipsing interaction with the indole(H). However, in this case energy is gained by oppositely aligned dipoles between the oxygen and fluorine atoms. Further rotation leads to an increase in energy as the dipoles begin to align and repel each other. The two highest energy conformers include this alignment of dipoles but also an eclipsed interaction between the H and indole(H).

Analysis of the angle between the dipoles of oxygen and fluorine reveals the lowest energy conformer is indeed when they are pointing in opposite directions, *i.e.*, at 180°.

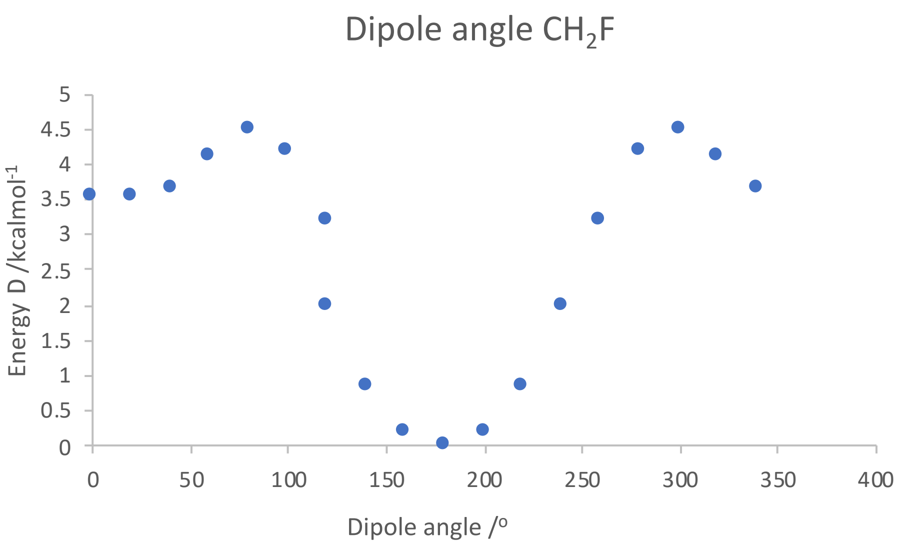

Figure S6. Graph of calculated ground state energies vs dipole angle for **4**.

<u>$CX_3 = CF_2H$</u>

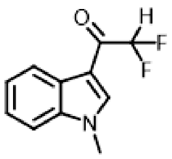

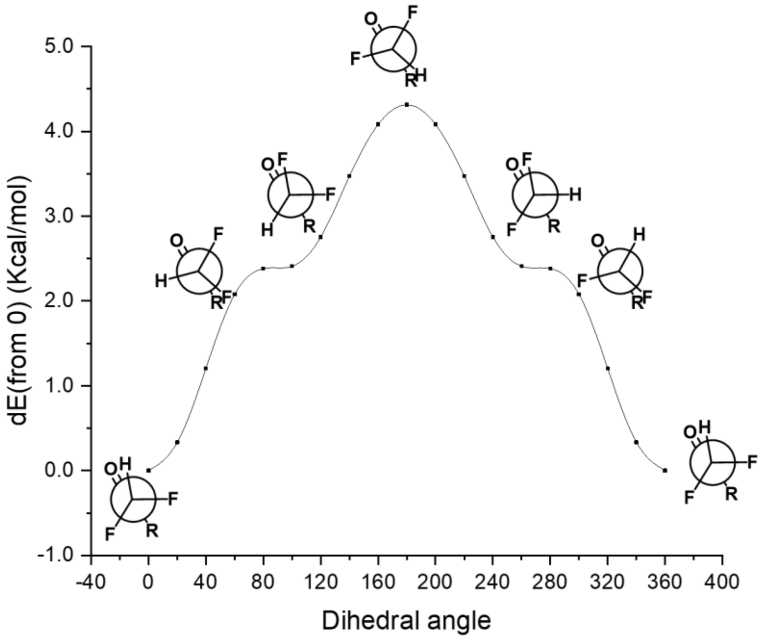

Figure S6. Graph of calculated ground state energies vs dihedral angle for **3a**.

From the O-H eclipsed conformer (dihedral angle = 0) an increase in energy occurs upon rotation. The highest energy conformer includes an eclipsing H-indole(H) interaction and the total alignment of the dipoles between the oxygen and two fluorine atoms. The levelling off between 80-100° and 260-300° is due to conformers without any indole(H) eclipsing interactions and with a perpendicular angle between the dipoles.

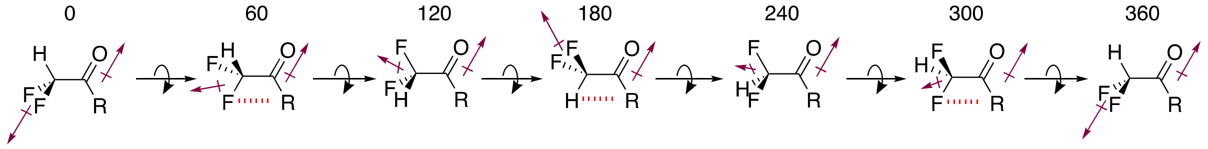

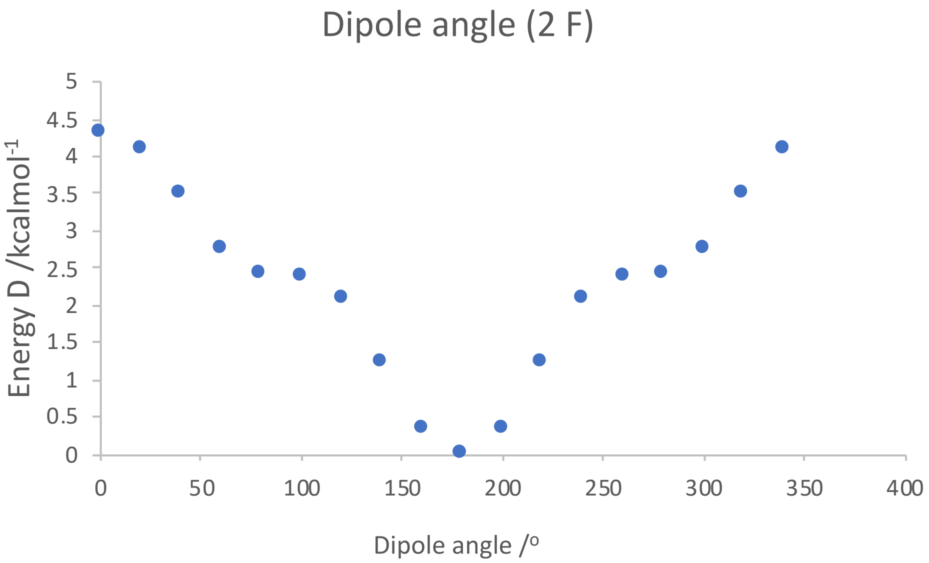

Figure S7. Graph of calculated ground state energies vs dipole angle for **3a**.

Overlaid plot

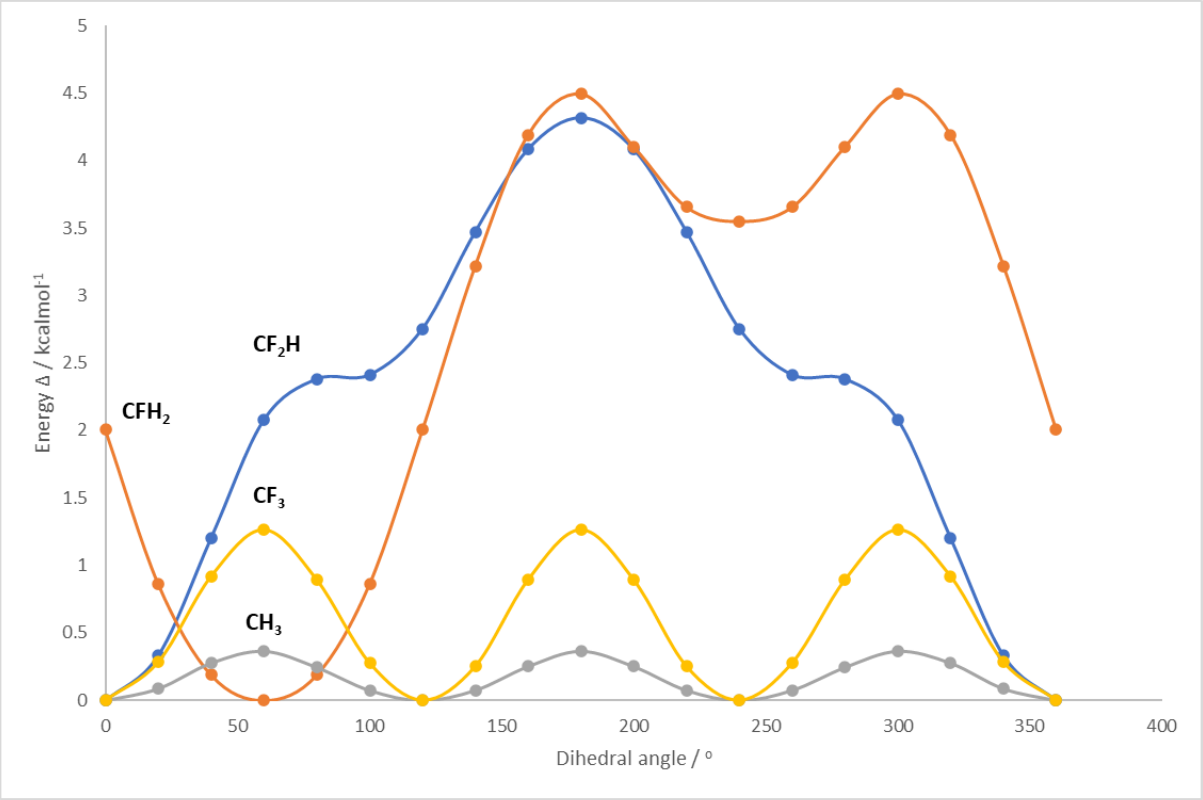

Figure S8. Overlaid graphs of calculated ground state energies vs dihedral angle for the series of fluorinated methyl ketones.

Mechanistic studies

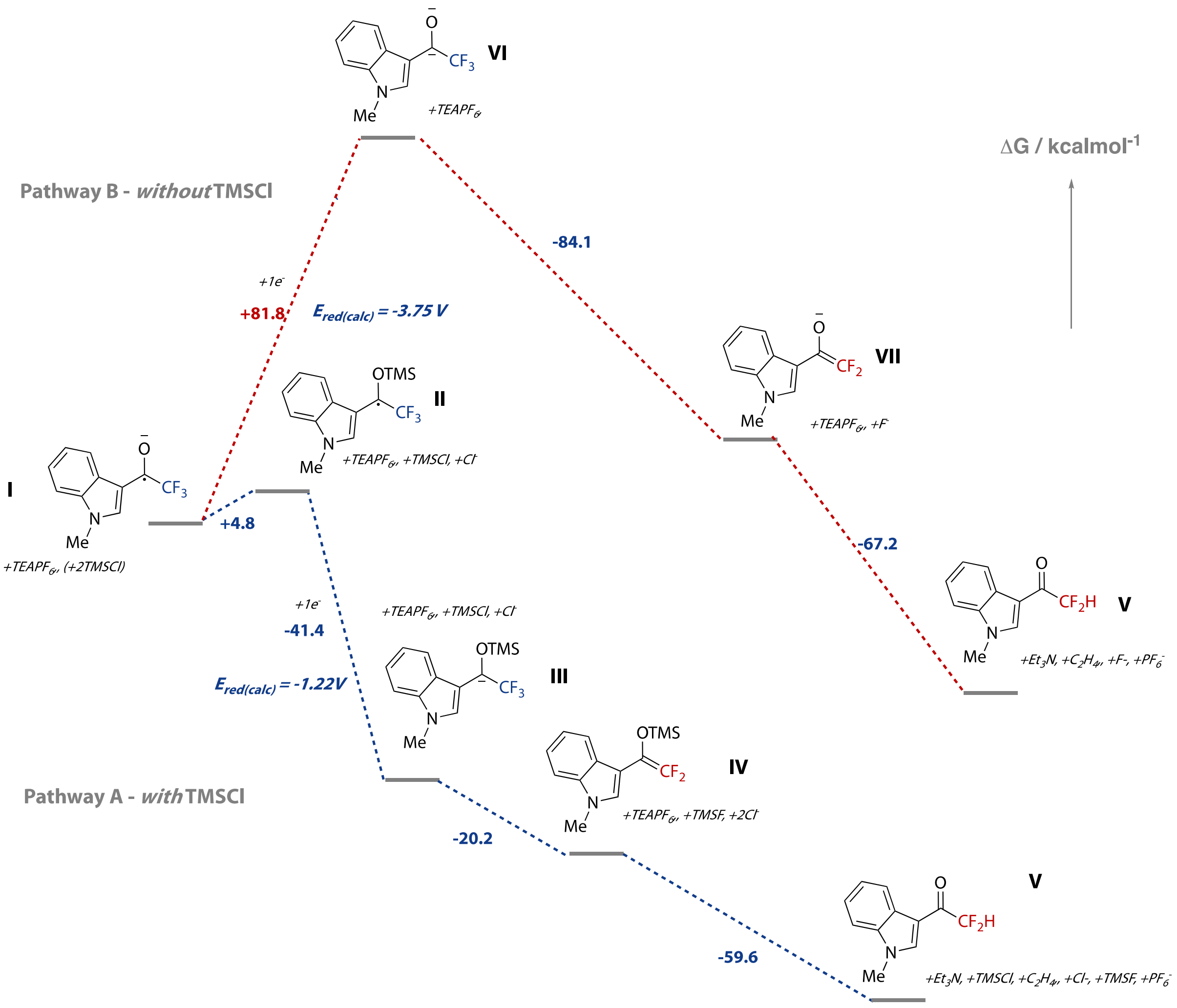

Figure S9. Calculated energy profile for reaction coordinate with and without TMSCl.

All DFT calculations were run using Gaussian 09W and Gaussview 5 software to the B3LYP/6-31G+p(d) level of theory. Structures were energy minimised and then frequency calculations carried out to compute Gibbs energies of formation in the gas phase. The total energy was calculated by the addition of the Gibbs energies for each species present (noted below the structure in question). Due to the additional complexities of modelling an electron transfer from a surface, we have not accounted for the energy of the second electron, and therefore the values shown should only be interpreted in a relative sense.

Key points to note:

a) In the absence of TMSCl, the reduced trifluoromethylketone **I** is unlikely to undergo a second one-electron reduction to yield dianion **VI** given the large energy barrier and very negative calculated reduction potential. It should be noted that during the course of reactions involving substrate **1a** applied potentials of *c.a.* -3.75 V were never observed (maximum applied potential was observed to be around -2.3 V).

b) Fluoride expulsion from **I** (not shown) was also examined and was found to have an energy barrier of >100 kcal/mol.

c) The loss of fluoride from **III** to **IV** is likely to be exergonic due to the formation of a strong Si-F bond in TMSF, which has been observed in crude reaction mixtures. TMSF is a gas at room temperature and pressure but appears to be soluble in MeCN.

Coordinates and energies for all species:

I

Zero-point correction= 0.166580 (Hartree/Particle)
Thermal correction to Energy= 0.180555
Thermal correction to Enthalpy= 0.181499
Thermal correction to Gibbs Free Energy= 0.123851
Sum of electronic and zero-point Energies= -853.587122
Sum of electronic and thermal Energies= -853.573148
Sum of electronic and thermal Enthalpies= -853.572204
Sum of electronic and thermal Free Energies= -853.629852

SCF Done: E(UB3LYP) = -853.753702558

Symbolic Z-matrix:
Charge = -1 Multiplicity = 2

C	-1.06514	-0.56132	-0.02653
C	-2.04716	0.46941	-0.05799
C	-3.41802	0.18702	-0.01366
C	-3.80653	-1.15243	0.02628
C	-2.85322	-2.18118	0.03147
C	-1.48477	-1.89466	0.00786
C	0.25386	0.06972	-0.03498
C	-0.00834	1.45249	-0.05624
H	-4.16219	0.97858	-0.01094
H	-4.86542	-1.39801	0.05799
H	-3.18405	-3.21603	0.06329
H	-0.73367	-2.6738	0.02351
H	0.6625	2.29167	-0.12137
C	-2.00938	2.95892	0.10463
H	-2.95498	3.03326	-0.43962
H	-2.20887	3.14698	1.17184
H	-1.34842	3.74768	-0.26211
N	-1.39591	1.67915	-0.12382
C	1.46663	-0.69317	-0.03118
C	2.80689	-0.00654	0.00495
O	1.51876	-1.96851	-0.00721
F	3.67098	-0.4623	-0.95548
F	3.48687	-0.20423	1.19001
F	2.77631	1.35476	-0.15517

II

Zero-point correction= 0.282551 (Hartree/Particle)
Thermal correction to Energy= 0.304538
Thermal correction to Enthalpy= 0.305482
Thermal correction to Gibbs Free Energy= 0.228826
Sum of electronic and zero-point Energies= -1262.772327
Sum of electronic and thermal Energies= -1262.750340
Sum of electronic and thermal Enthalpies= -1262.749396
Sum of electronic and thermal Free Energies= -1262.826052

SCF Done: E(UB3LYP) = -1263.05487799

Symbolic Z-matrix:
Charge = 0 Multiplicity = 2

C	-2.83186	-0.81851	0.01071
C	-1.49198	-1.21787	0.2461
C	-0.42293	-0.32068	0.20774
C	-0.70896	1.00832	-0.08147
C	-2.02627	1.42483	-0.33264
C	-3.08638	0.52529	-0.2911
C	-3.66221	-2.01684	0.11977
C	-2.76748	-3.05856	0.40632
H	0.59644	-0.6434	0.39151
H	0.09907	1.73166	-0.11956
H	-2.21916	2.46638	-0.56817
H	-4.09357	0.85458	-0.50222
H	-2.96001	-4.10433	0.57084
C	-0.30199	-3.37422	0.75761
H	0.40044	-3.33266	-0.08003
H	0.20574	-3.02137	1.65978
H	-0.59727	-4.41147	0.91008
N	-1.48204	-2.58036	0.48453
C	-5.0684	-2.0711	-0.01619
C	-5.8639	-3.32344	-0.14075
O	-5.76684	-0.93415	-0.25748
F	-6.44074	-3.45906	-1.36824
F	-6.91286	-3.35774	0.74391
F	-5.14801	-4.44657	0.07834
Si	-7.11571	-0.15466	0.44257
C	-8.69777	-0.81113	-0.32146
H	-8.87579	-1.85908	-0.07244
H	-9.56107	-0.23591	0.03076
H	-8.67387	-0.72889	-1.41218
C	-7.07552	-0.38474	2.30522
H	-7.87379	0.19508	2.78089
H	-7.21401	-1.42949	2.59293
H	-6.12693	-0.04282	2.72975
C	-6.87883	1.64659	-0.02729
H	-7.75465	2.238	0.25967
H	-6.01065	2.09199	0.46612
H	-6.74809	1.76474	-1.10702

III

Zero-point correction= 0.280859 (Hartree/Particle)
Thermal correction to Energy= 0.303257
Thermal correction to Enthalpy= 0.304201
Thermal correction to Gibbs Free Energy= 0.229029
Sum of electronic and zero-point Energies= -1262.839929
Sum of electronic and thermal Energies= -1262.817531
Sum of electronic and thermal Enthalpies= -1262.816587
Sum of electronic and thermal Free Energies= -1262.891759

SCF Done: E(RB3LYP) = -1263.12078802

Symbolic Z-matrix:
Charge = -1 Multiplicity = 1

C	-0.30269	1.63677	0.
C	0.37731	0.38464	-0.12144
C	1.76934	0.28444	-0.09694
C	2.50634	1.45402	0.06346
C	1.85981	2.69519	0.20161
C	0.47239	2.79491	0.17179
C	-1.71593	1.3345	-0.04733
C	-1.82217	-0.03827	-0.17521
H	2.26228	-0.67828	-0.19377
H	3.59145	1.40631	0.0864
H	2.45594	3.59422	0.33275
H	-0.00146	3.76349	0.27625
H	-2.6731	-0.73008	-0.25045
C	-0.37004	-2.03647	-0.37425
H	0.21787	-2.25033	-1.27328
H	0.16007	-2.43909	0.49601
H	-1.36333	-2.49472	-0.45686
N	-0.57731	-0.60364	-0.23069
C	-2.90174	2.18476	0.05744
C	-2.98197	3.45445	-0.35531
O	-4.00515	1.63187	0.64483
F	-4.06097	4.24041	-0.20604
F	-3.4589	-2.27974	-0.5161
F	-2.0101	4.14312	-0.97422
Si	-5.38553	0.85926	0.00782
C	-5.10013	0.35901	-1.77745
H	-4.48998	-0.55144	-1.79203
H	-6.0544	0.13314	-2.26821
H	-4.60462	1.13927	-2.36439
C	-5.68036	-0.63478	1.08855
H	-6.68981	-1.03813	0.94259
H	-4.95709	-1.41146	0.79999
H	-5.56349	-0.39922	2.15161
C	-6.79861	2.11015	0.14688
H	-7.74996	1.66587	-0.16865
H	-6.92117	2.4534	1.17944
H	-6.62154	2.99444	-0.47267

IV

Zero-point correction= 0.280127 (Hartree/Particle)
Thermal correction to Energy= 0.301196
Thermal correction to Enthalpy= 0.302140
Thermal correction to Gibbs Free Energy= 0.228478
Sum of electronic and zero-point Energies= -1162.894868
Sum of electronic and thermal Energies= -1162.873799
Sum of electronic and thermal Enthalpies= -1162.872854
Sum of electronic and thermal Free Energies= -1162.946517

SCF Done: E(RB3LYP) = -1163.17499452

Symbolic Z-matrix:
Charge = 0 Multiplicity = 1

C	-1.07692	-0.64835	0.
C	0.21238	-1.00321	0.47936
C	1.30336	-0.13199	0.40808
C	1.09219	1.11568	-0.16295
C	-0.17503	1.48478	-0.65261
C	-1.25699	0.61912	-0.57771
C	-1.93466	-1.78959	0.23071
C	-1.14484	-2.75085	0.82236
H	2.28284	-0.41546	0.77936
H	1.91813	1.81588	-0.235
H	-0.3051	2.46564	-1.09859
H	-2.22833	0.9103	-0.95694
H	-1.40522	-3.74529	1.15055
C	1.24527	-3.02895	1.55865
H	2.05119	-3.16865	0.8324
H	1.6467	-2.50819	2.4326
H	0.89103	-4.00977	1.87382
N	0.1432	-2.29102	0.976
C	-3.3528	-1.89321	-0.11463
C	-3.95681	-3.04359	-0.42596
O	-4.06404	-0.72654	-0.17349
F	-5.25317	-3.16103	-0.72176
F	-3.36137	-4.23789	-0.46925
Si	-5.29219	-0.12337	0.83691
C	-6.90972	-0.18311	-0.11155
H	-7.19832	-1.20869	-0.35535
H	-7.72567	0.26431	0.46619
H	-6.83442	0.36954	-1.05289
C	-5.37327	-1.1576	2.40371
H	-6.12115	-0.74944	3.09172
H	-5.6549	-2.19442	2.19975
H	-4.41584	-1.17013	2.93269
C	-4.80975	1.65202	1.20235
H	-5.55447	2.1432	1.83748
H	-3.84501	1.71096	1.71377
H	-4.73027	2.23949	0.28268

V

Zero-point correction= 0.180069 (Hartree/Particle)
Thermal correction to Energy= 0.192994
Thermal correction to Enthalpy= 0.193938
Thermal correction to Gibbs Free Energy= 0.139200
Sum of electronic and zero-point Energies= -754.271069
Sum of electronic and thermal Energies= -754.258144
Sum of electronic and thermal Enthalpies= -754.257200
Sum of electronic and thermal Free Energies= -754.311937

SCF Done: E(RB3LYP) = -754.451137898

Symbolic Z-matrix:
Charge = 0 Multiplicity = 1

C	-0.86809	-0.60811	-0.00003
C	-1.66226	0.56255	0.00005
C	-3.05657	0.52442	0.00006
C	-3.65711	-0.72947	0.00002
C	-2.88588	-1.9048	-0.00003
C	-1.49698	-1.86004	-0.00006
C	0.51244	-0.17036	-0.00002
C	0.48077	1.21777	-0.00002
H	-3.65482	1.42932	0.00009
H	-4.73974	-0.80071	0.00003
H	-3.38828	-2.86653	-0.00005
H	-0.90367	-2.76543	-0.00009
H	1.29912	1.92113	-0.00002
C	1.68388	-1.01635	-0.00003
N	-0.8034	1.66161	0.00011
C	-1.22579	3.05077	-0.00007
H	-1.82043	3.27209	-0.88975
H	-1.82178	3.27185	0.88876
H	-0.34567	3.69205	0.0007
O	1.65695	-2.23836	-0.00001
C	3.08032	-0.35892	-0.00002
F	3.24742	0.44427	-1.10277
F	3.24744	0.44413	1.10284
H	3.86133	-1.11853	-0.00008

VI

Zero-point correction= 0.164511 (Hartree/Particle)
Thermal correction to Energy= 0.178485
Thermal correction to Enthalpy= 0.179429
Thermal correction to Gibbs Free Energy= 0.122894
Sum of electronic and zero-point Energies= -853.458423
Sum of electronic and thermal Energies= -853.444449
Sum of electronic and thermal Enthalpies= -853.443504
Sum of electronic and thermal Free Energies= -853.500040

SCF Done: E(RB3LYP) = -853.622933915

Symbolic Z-matrix:
Charge = -2 Multiplicity = 1

C	-3.45055	0.49451	0.
C	-4.43829	1.51695	-0.10496
C	-5.80969	1.22741	-0.04302
C	-6.18739	-0.11688	0.00393
C	-5.23221	-1.14141	0.03174
C	-3.86038	-0.84206	0.0565
C	-2.13514	1.13825	0.03333
C	-2.41925	2.54099	0.03035
H	-6.56179	2.01229	-0.05831
H	-7.2484	-0.36549	0.02888
H	-5.56037	-2.17947	0.0686
H	-3.101	-1.61092	0.12904
H	-1.75686	3.34659	-0.24051
C	-4.46661	3.97233	0.18959
H	-5.42172	4.08643	-0.33048
H	-4.66274	4.01435	1.27827
H	-3.83447	4.8239	-0.06168
N	-3.80341	2.73664	-0.19823
C	-0.93444	0.37797	0.03591
C	0.39388	1.05944	0.00499
O	-0.8618	-0.91236	0.07614
F	1.22004	0.64008	-1.0382
F	1.18779	0.80572	1.12705
F	0.38158	2.42314	-0.0966

VII

Zero-point correction= 0.164121 (Hartree/Particle)
Thermal correction to Energy= 0.177362
Thermal correction to Enthalpy= 0.178306
Thermal correction to Gibbs Free Energy= 0.122858
Sum of electronic and zero-point Energies= -753.689346
Sum of electronic and thermal Energies= -753.676105
Sum of electronic and thermal Enthalpies= -753.675161
Sum of electronic and thermal Free Energies= -753.730608

SCF Done: E(RB3LYP) = -753.853466789

Symbolic Z-matrix:
Charge = -1 Multiplicity = 1

C	-2.15385	1.46154	0.
C	-3.11864	2.50894	0.0152
C	-4.49529	2.26175	0.05449
C	-4.91098	0.93395	0.06917
C	-3.97365	-0.11723	0.04921
C	-2.6054	0.13242	0.01569
C	-0.83956	2.07199	-0.03062
C	-1.06973	3.42904	-0.02936
H	-5.21906	3.07228	0.07369
H	-5.97377	0.70737	0.0989
H	-4.32977	-1.14409	0.06326
H	-1.86674	-0.65874	0.00738
H	-0.36261	4.24181	-0.05913
C	-3.02931	5.0149	0.05236
H	-3.76619	5.15616	-0.74654
H	-3.52916	5.18973	1.0139
H	-2.24824	5.76632	-0.06874
N	-2.43478	3.70446	-0.01247
C	0.44416	1.29339	-0.01371
C	1.62559	2.00257	-0.18591
O	0.37924	0.02336	0.04379
F	2.83591	1.43681	0.11885
F	1.72326	3.35742	0.13885

$TEAPF_6$

Zero-point correction= 0.298435 (Hartree/Particle)
Thermal correction to Energy= 0.318097
Thermal correction to Enthalpy= 0.319041
Thermal correction to Gibbs Free Energy= 0.250884
Sum of electronic and zero-point Energies= -1312.207637
Sum of electronic and thermal Energies= -1312.187975
Sum of electronic and thermal Enthalpies= -1312.187031
Sum of electronic and thermal Free Energies= -1312.255188

SCF Done: E(RB3LYP) = -1312.50607223

Symbolic Z-matrix:
Charge = 0 Multiplicity = 1

N	-2.01359	0.29791	-0.23644
C	-1.53285	-0.83926	-1.15369
C	-2.37394	-2.10494	-1.23988
H	-0.5152	-1.05558	-0.83742
H	-1.47067	-0.39004	-2.1455
H	-1.84897	-2.77664	-1.92418
H	-2.47785	-2.63287	-0.29426
H	-3.36558	-1.93486	-1.66382
C	-1.30915	1.5588	-0.79392
C	-1.33815	2.80022	0.08409
H	-1.79452	1.74922	-1.75329
H	-0.27983	1.26247	-0.98337
H	-0.88525	3.60718	-0.49729
H	-2.342	3.13134	0.3619
H	-0.73379	2.68648	0.98233
C	-3.51181	0.54241	-0.35703
C	-4.45697	-0.27887	0.51088
H	-3.74611	0.41509	-1.4154
H	-3.65929	1.59569	-0.12375
H	-5.47467	0.04095	0.27055
H	-4.40113	-1.3501	0.33165
H	-4.30842	-0.09351	1.57498
C	-1.62017	-1.27676	1.82639
C	-1.54316	0.1144	1.21387
H	-2.62191	-1.70142	1.87315
H	-0.94351	-1.97206	1.33302
H	-1.25841	-1.17813	2.8534
H	-0.50194	0.42683	1.21608
H	-2.11581	0.83558	1.798
P	2.43147	-0.08846	0.00335
F	1.56687	0.23394	-1.38605
F	1.48254	1.05176	0.77692
F	3.50092	1.06242	-0.38641
F	3.18925	-0.40351	1.40027
F	3.27662	-1.22611	-0.77985
F	1.25992	-1.22073	0.38666

TMSCl

Zero-point correction= 0.112781 (Hartree/Particle)
Thermal correction to Energy= 0.121319
Thermal correction to Enthalpy= 0.122263
Thermal correction to Gibbs Free Energy= 0.081335
Sum of electronic and zero-point Energies= -869.491095
Sum of electronic and thermal Energies= -869.482556
Sum of electronic and thermal Enthalpies= -869.481612
Sum of electronic and thermal Free Energies= -869.522541

SCF Done: E(RB3LYP) = -869.603875230

Symbolic Z-matrix:
Charge = 0 Multiplicity = 1

Si	0.	0.	-0.33622
C	0.	1.78896	-0.89954
H	0.	1.83586	-1.99415
H	-0.88395	2.3227	-0.53953
H	0.88395	2.3227	-0.53953
C	1.54928	-0.89448	-0.89954
H	2.45349	-0.39583	-0.53953
H	1.56954	-1.92687	-0.53953
H	1.5899	-0.91793	-1.99415
C	-1.54928	-0.89448	-0.89954
H	-1.56954	-1.92687	-0.53953
H	-2.45349	-0.39583	-0.53953
H	-1.5899	-0.91793	-1.99415
Cl	0.	0.	1.77168

Cl^-

Zero-point correction= 0.000000 (Hartree/Particle)
Thermal correction to Energy= 0.001416
Thermal correction to Enthalpy= 0.002360
Thermal correction to Gibbs Free Energy= -0.015023
Sum of electronic and zero-point Energies= -460.303727
Sum of electronic and thermal Energies= -460.302311
Sum of electronic and thermal Enthalpies= -460.301367
Sum of electronic and thermal Free Energies= -460.318750

SCF Done: E(RB3LYP) = -460.303727178

Symbolic Z-matrix:
Charge = -1 Multiplicity = 1
Cl 0. 0. 0.

TMSF

Zero-point correction= 0.113496 (Hartree/Particle)
Thermal correction to Energy= 0.121875
Thermal correction to Enthalpy= 0.122819
Thermal correction to Gibbs Free Energy= 0.082650
Sum of electronic and zero-point Energies= -509.150346
Sum of electronic and thermal Energies= -509.141967
Sum of electronic and thermal Enthalpies= -509.141023
Sum of electronic and thermal Free Energies= -509.181192

SCF Done: E(RB3LYP) = -509.263841886

Symbolic Z-matrix:
Charge = 0 Multiplicity = 1

C	0.	1.79428	-0.52325
H	-0.88215	2.32289	-0.15029
H	0.88215	2.32289	-0.15029
H	0.	1.88522	-1.61447
C	1.55389	-0.89714	-0.52325
C	-1.55389	-0.89714	-0.52325
H	1.57061	-1.92541	-0.15029
H	1.63265	-0.94261	-1.61447
H	2.45276	-0.39748	-0.15029
H	-1.63265	-0.94261	-1.61447
H	-1.57061	-1.92541	-0.15029
H	-2.45276	-0.39748	-0.15029
Si	0.	0.	0.01465
F	0.	0.	1.66206

F^-

Zero-point correction= 0.000000 (Hartree/Particle)
Thermal correction to Energy= 0.001416
Thermal correction to Enthalpy= 0.002360
Thermal correction to Gibbs Free Energy= -0.014159
Sum of electronic and zero-point Energies= -99.888693
Sum of electronic and thermal Energies= -99.887277
Sum of electronic and thermal Enthalpies= -99.886333
Sum of electronic and thermal Free Energies= -99.902852

SCF Done: E(RB3LYP) = -99.8886932053

Symbolic Z-matrix:
Charge = -1 Multiplicity = 1
F 0. 0. 0.

Et_3N

Zero-point correction= 0.205370 (Hartree/Particle)
Thermal correction to Energy= 0.214875
Thermal correction to Enthalpy= 0.215819
Thermal correction to Gibbs Free Energy= 0.171297
Sum of electronic and zero-point Energies= -292.277032
Sum of electronic and thermal Energies= -292.267528
Sum of electronic and thermal Enthalpies= -292.266584
Sum of electronic and thermal Free Energies= -292.311106

SCF Done: E(RB3LYP) = -292.482402760

Symbolic Z-matrix:
Charge = 0 Multiplicity = 1

N	0.00082	0.00021	0.01306
C	1.3101	0.50917	0.43761
C	2.46641	0.01641	-0.43098
H	1.50125	0.26424	1.49978
H	1.28214	1.5997	0.38267
H	3.41087	0.44875	-0.08622
H	2.57842	-1.07042	-0.40209
H	2.31404	0.30845	-1.47287
C	-0.21256	-1.38841	0.43691
C	-1.22097	-2.14099	-0.42978
H	-0.51721	-1.43269	1.49993
H	0.74519	-1.91023	0.3781
H	-1.31843	-3.17576	-0.08697
H	-2.21776	-1.69377	-0.3967
H	-0.89545	-2.1529	-1.47283
C	-1.09353	0.87941	0.44077
C	-1.24986	2.12415	-0.43146
H	-0.97171	1.17118	1.50129
H	-2.024	0.30939	0.39258
H	-2.09488	2.72739	-0.08521
H	-0.36443	2.76477	-0.40983
H	-1.43137	1.84201	-1.47143

C_2H_4

Zero-point correction= 0.051116 (Hartree/Particle)
Thermal correction to Energy= 0.054139
Thermal correction to Enthalpy= 0.055083
Thermal correction to Gibbs Free Energy= 0.029594
Sum of electronic and zero-point Energies= -78.542112
Sum of electronic and thermal Energies= -78.539089
Sum of electronic and thermal Enthalpies= -78.538145
Sum of electronic and thermal Free Energies= -78.563633

SCF Done: E(RB3LYP) = -78.5932276828

Symbolic Z-matrix:
Charge = 0 Multiplicity = 1

C	0.	-0.66648	0.00006
H	0.91942	-1.23984	0.00001
H	-0.91939	-1.23988	-0.00037
C	0.	0.66648	0.00006
H	-0.91942	1.23984	0.00001
H	0.91939	1.23988	-0.00037

PF_6^-

```
Zero-point correction=                           0.018175 (Hartree/Particle)
 Thermal correction to Energy=                    0.024487
 Thermal correction to Enthalpy=                  0.025431
 Thermal correction to Gibbs Free Energy=        -0.009144
 Sum of electronic and zero-point Energies=     -940.878439
 Sum of electronic and thermal Energies=        -940.872128
 Sum of electronic and thermal Enthalpies=      -940.871183
 Sum of electronic and thermal Free Energies=   -940.905758
```

SCF Done: E(RB3LYP) = -940.896614019

```
Symbolic Z-matrix:
 Charge = -1 Multiplicity = 1
 P              0.        0.        0.
 F              0.        0.        1.64576
 F              0.        1.64576   0.
 F              1.64576   0.        0.
 F              0.        0.       -1.64576
 F              0.       -1.64576   0.
 F             -1.64576   0.        0.
```

Total energies of intermediate species:

		I	II	III	IV	V
**with TMSCl**	**Total E of all species**	-3904.93024	-3904.92	-3904.99	-3905.02	-3905.11
	**dE / Ha/particle**		0.007649	-0.06571	-0.0321	-0.09458
	**dE / kcal/mol**		4.819181	-41.3954	-20.2227	-59.5857
		**I**	**VI**	**VII**	**V**	
**without TMSCl**	**Total E of all species**	-2165.88504	-2165.76	-2165.89	-2166	
	**dE / Ha/particle**		0.129812	-0.13342	-0.10664	
	**dE / kcal/mol**		81.78133	-84.0553	-67.1822	

CIF of 3ae

X-ray diffraction experiments on **3ae** were carried out at 100(2) K on a Bruker APEX II diffractometer using Mo-K_α radiation (λ = 0.71073 Å). Data collections were performed using a CCD area detector. Intensities were integrated in SAINT[25] and absorption corrections based on equivalent reflections were applied using SADABS.[26] The structure was solved using ShelXT[27] and refined by full matrix least squares against F^2 in ShelXL[27,28] using Olex2.[29] All of the non-hydrogen atoms were refined anisotropically. While all of the hydrogen atoms were located geometrically and refined using a riding model. The structure was refined as a two component non-merohedral twin. The crystal structure and refinement data are given in Table S4. Crystallographic data has been deposited with the Cambridge Crystallographic Data Centre as supplementary publication CCDC 2061359. Copies of the data can be obtained free of charge on application to CCDC, 12 Union Road, Cambridge CB2 1EZ, UK [fax(+44) 1223 336033, e-mail: deposit@ccdc.cam.ac.uk].

CCDC number	2061359
Empirical formula	$C_{11}H_8BrF_2NO$
Formula weight	288.09
Temperature/K	100(2)
Crystal system	monoclinic
Space group	$P2_1/c$
a/Å	7.3373(5)
b/Å	14.2535(9)
c/Å	9.9567(6)
α/°	90
β/°	90.050(4)
γ/°	90
Volume/Å^3	1041.29(12)
Z	4
ρ_{calc}g/cm^3	1.838
μ/mm^{-1}	3.951
F(000)	568.0
Crystal size/mm^3	0.42 × 0.18 × 0.1
Radiation	MoKα (λ = 0.71073)
2Θ range for data collection/°	4.99 to 56.106
Index ranges	-9 ≤ h ≤ 9, -18 ≤ k ≤ 18, 0 ≤ l ≤ 13
Reflections collected	2506
Independent reflections	2506 [R_{int} = ?, R_{sigma} = 0.0201]
Data/restraints/parameters	2506/0/148
Goodness-of-fit on F^2	1.184
Final R indexes [I>=2σ (I)]	R_1 = 0.0282, wR_2 = 0.0640
Final R indexes [all data]	R_1 = 0.0307, wR_2 = 0.0647
Largest diff. peak/hole / e Å^{-3}	0.88/-0.53

Table S4. Crystal data and structure refinement for **3ae**.

NMR Spectra of Novel Substrates

2,2,2-trifluoro-1-(4-pentylphenyl)ethan-1-one, **1j**

^{1}H NMR (400 MHz, $CDCl_3$):

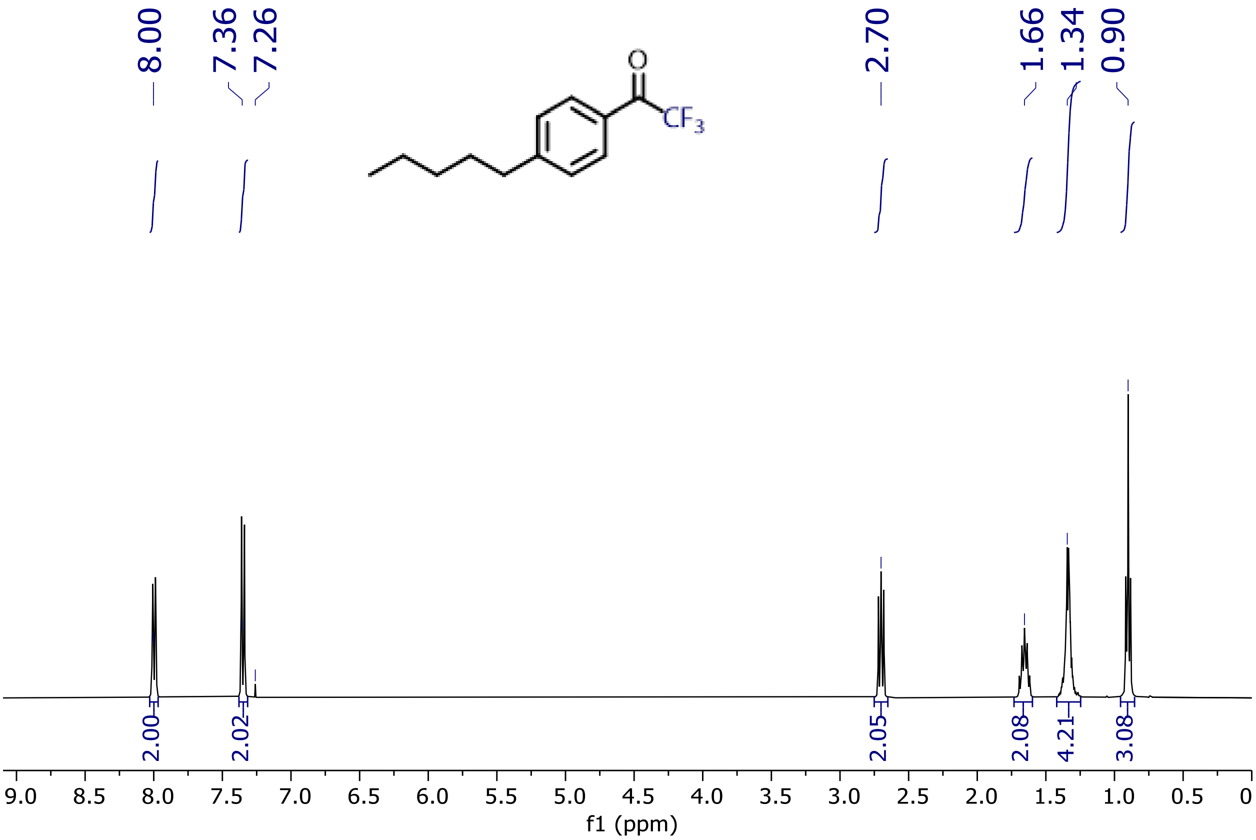

^{13}C NMR (101 MHz, $CDCl_3$):

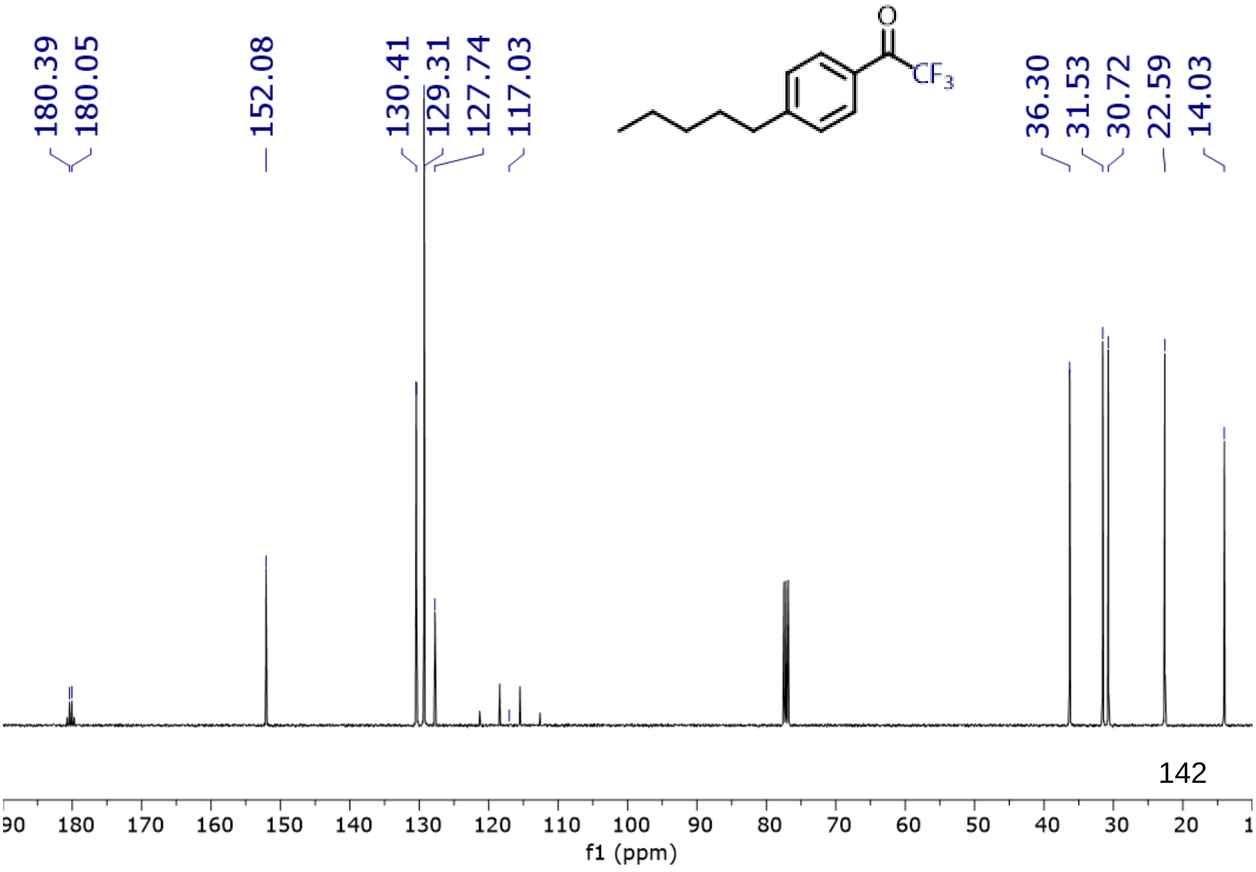

^{19}F NMR (376 MHz, $CDCl_3$):

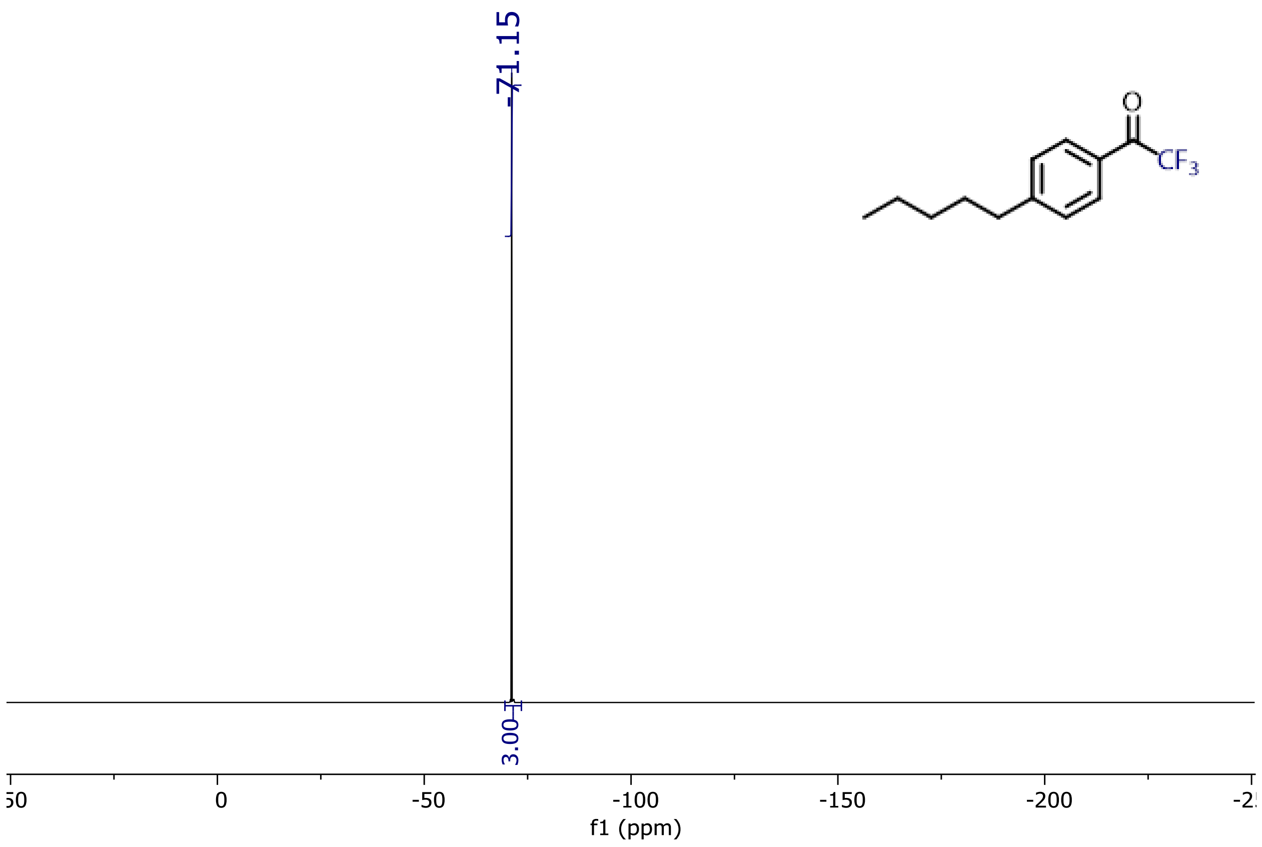

1-(4-(benzyloxy)-2-methylphenyl)-2,2,2-trifluoroethan-1-one, ***1k***

^{1}H NMR (400 MHz, $CDCl_3$):

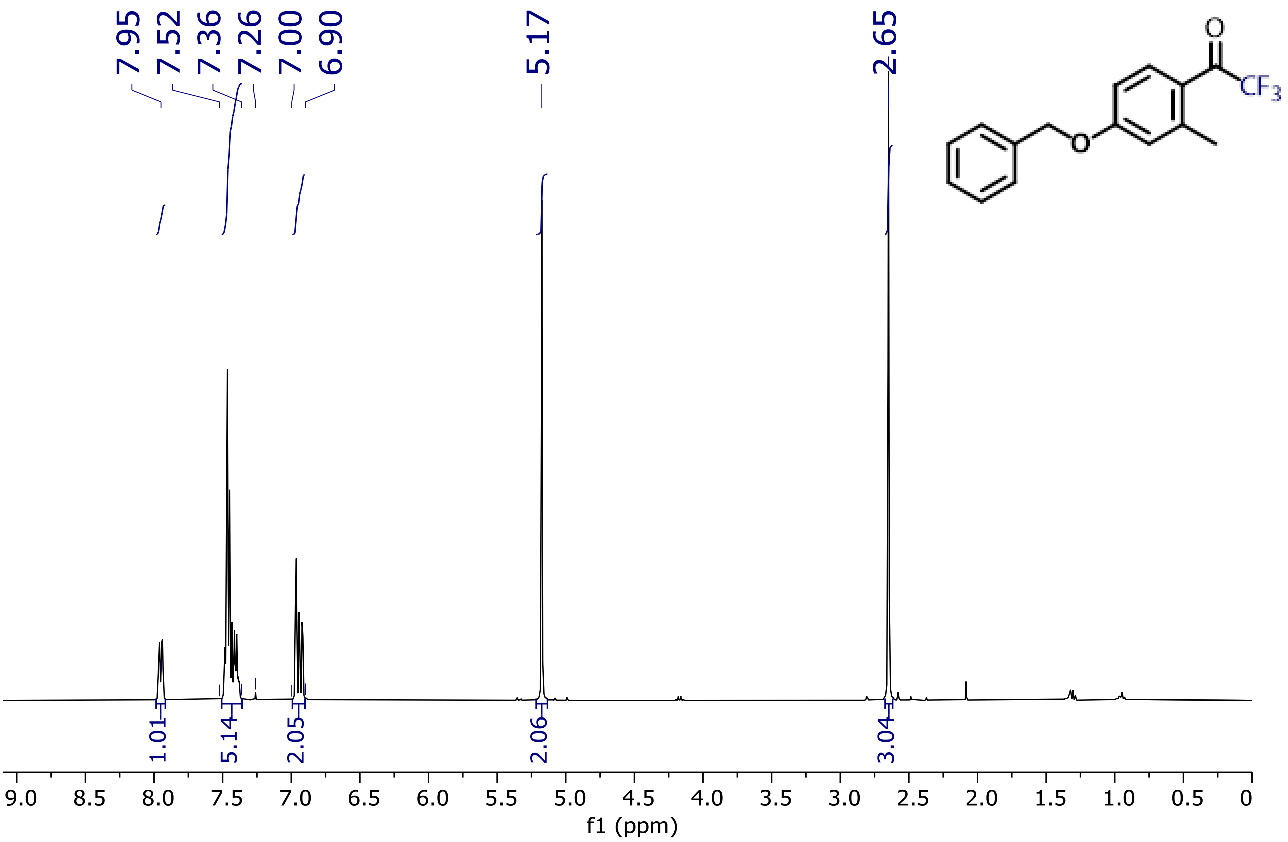

^{13}C NMR (101 MHz, $CDCl_3$):

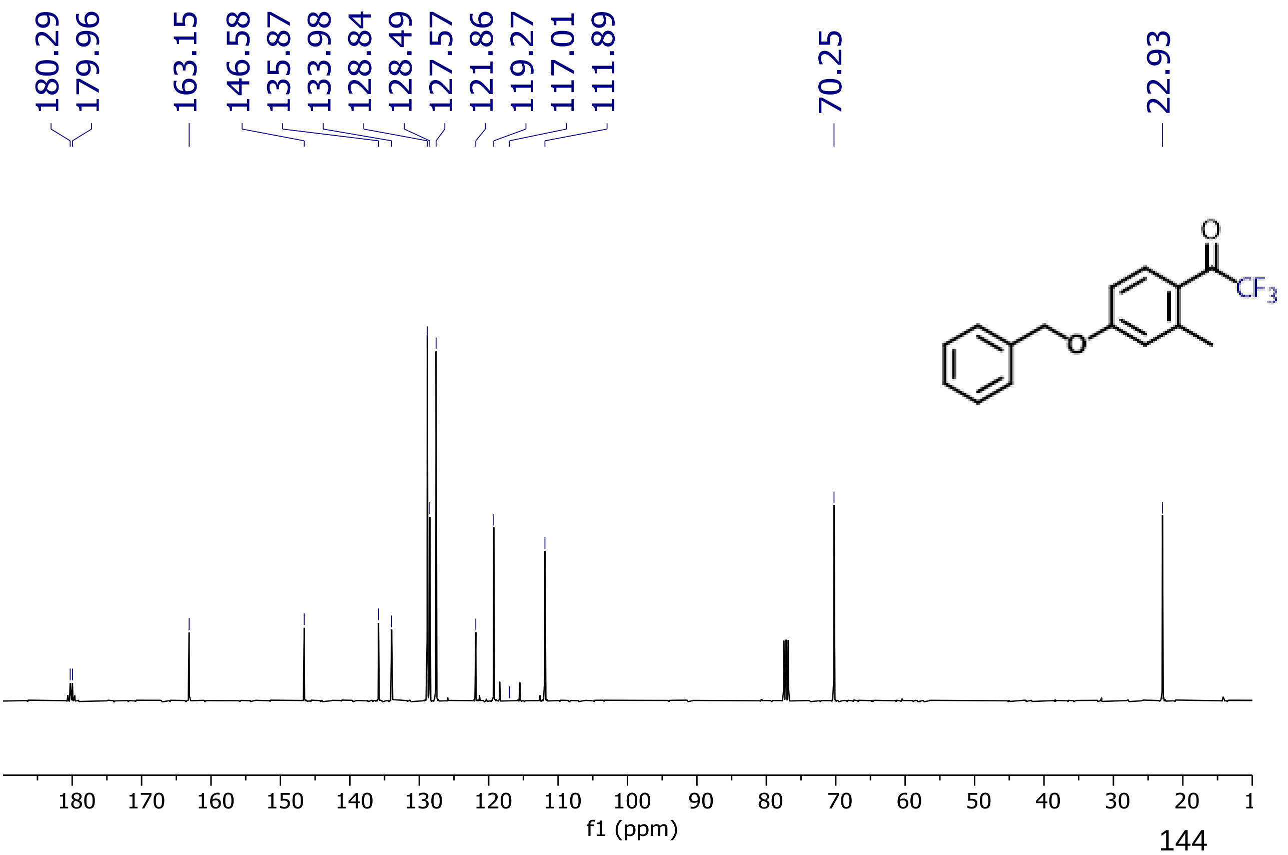

^{19}F NMR (376 MHz, $CDCl_3$):

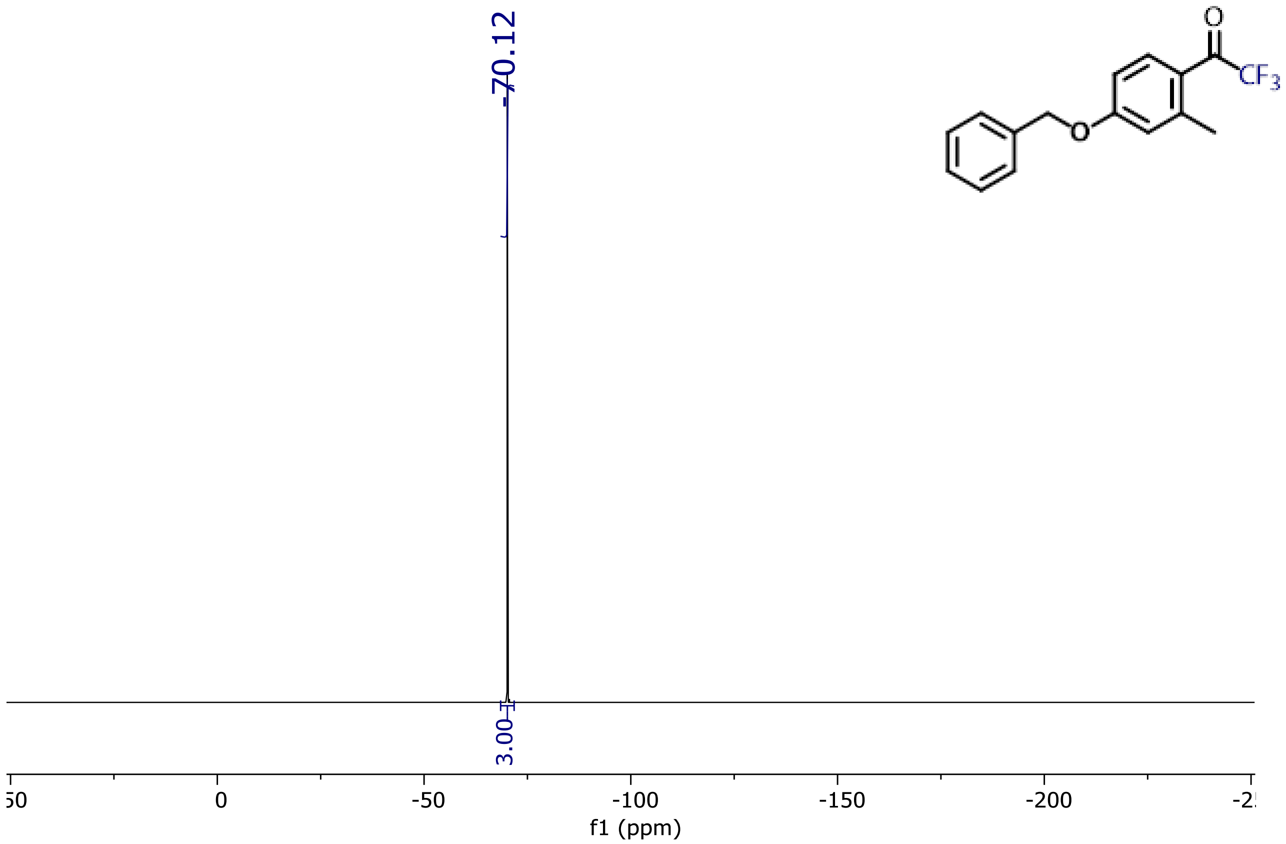

2,2,2-trifluoro-1-(4-(pyridin-2-yl)phenyl)ethan-1-one, **1l**

^{1}H NMR (400 MHz, $CDCl_3$):

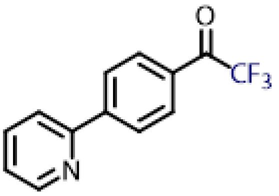

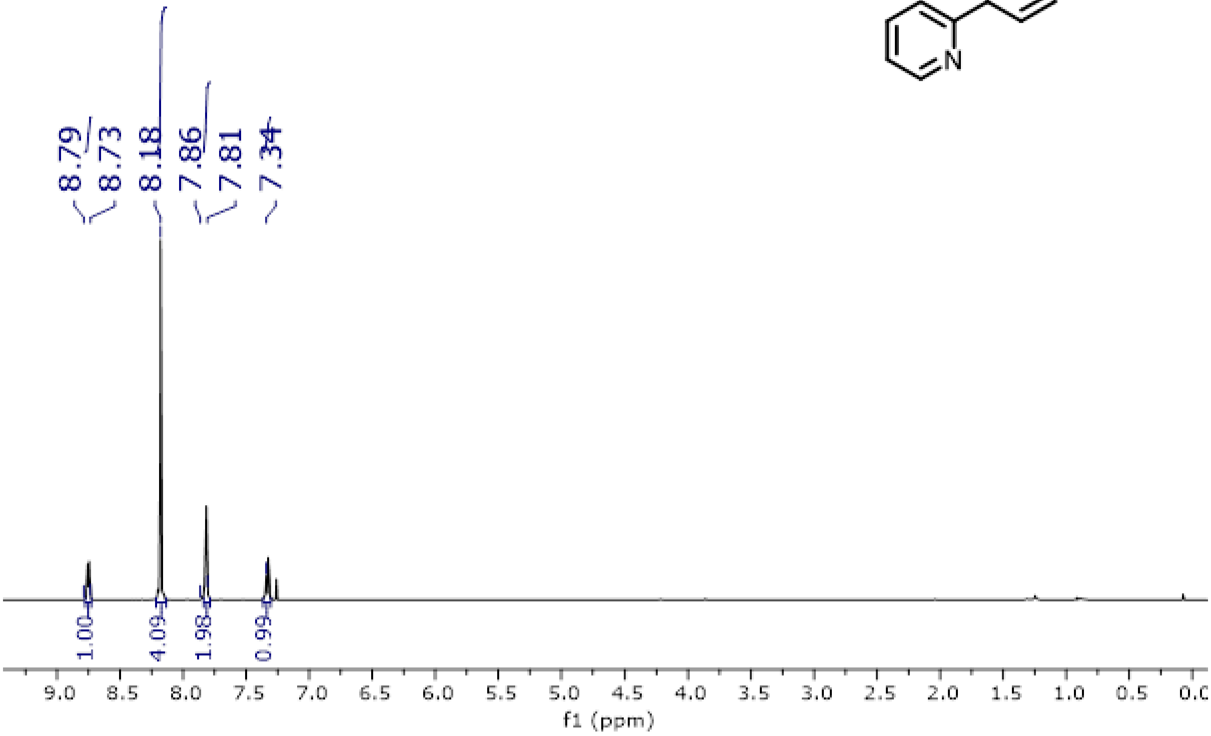

^{13}C NMR (126 MHz, $CDCl_3$):

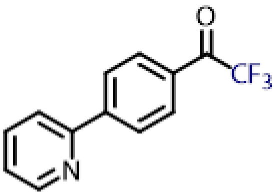

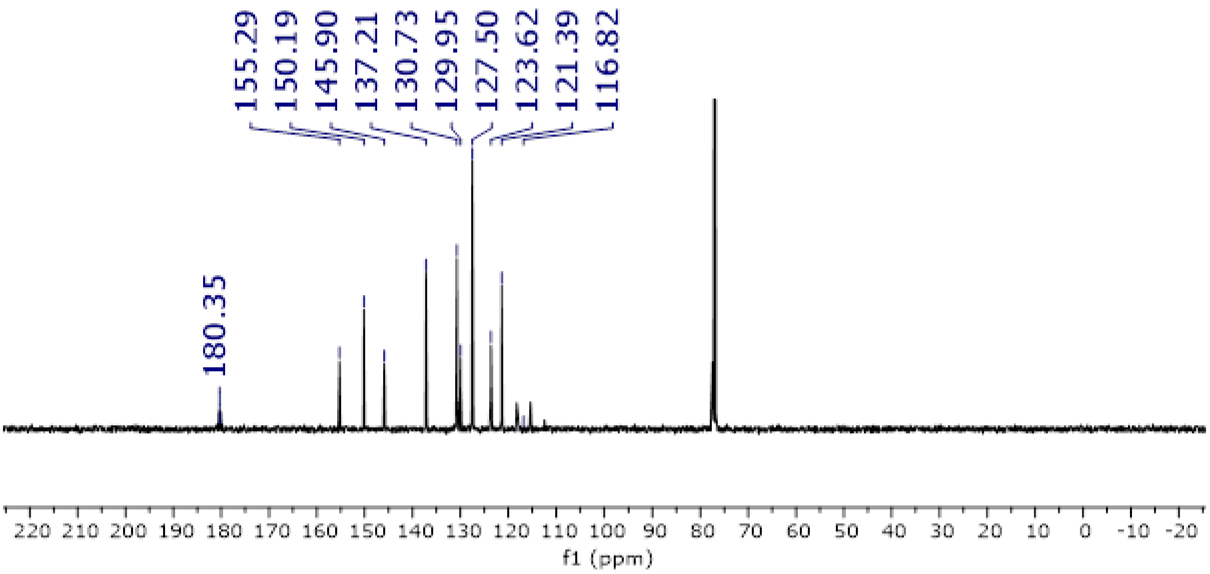

^{19}F NMR (377 MHz, $CDCl_3$):

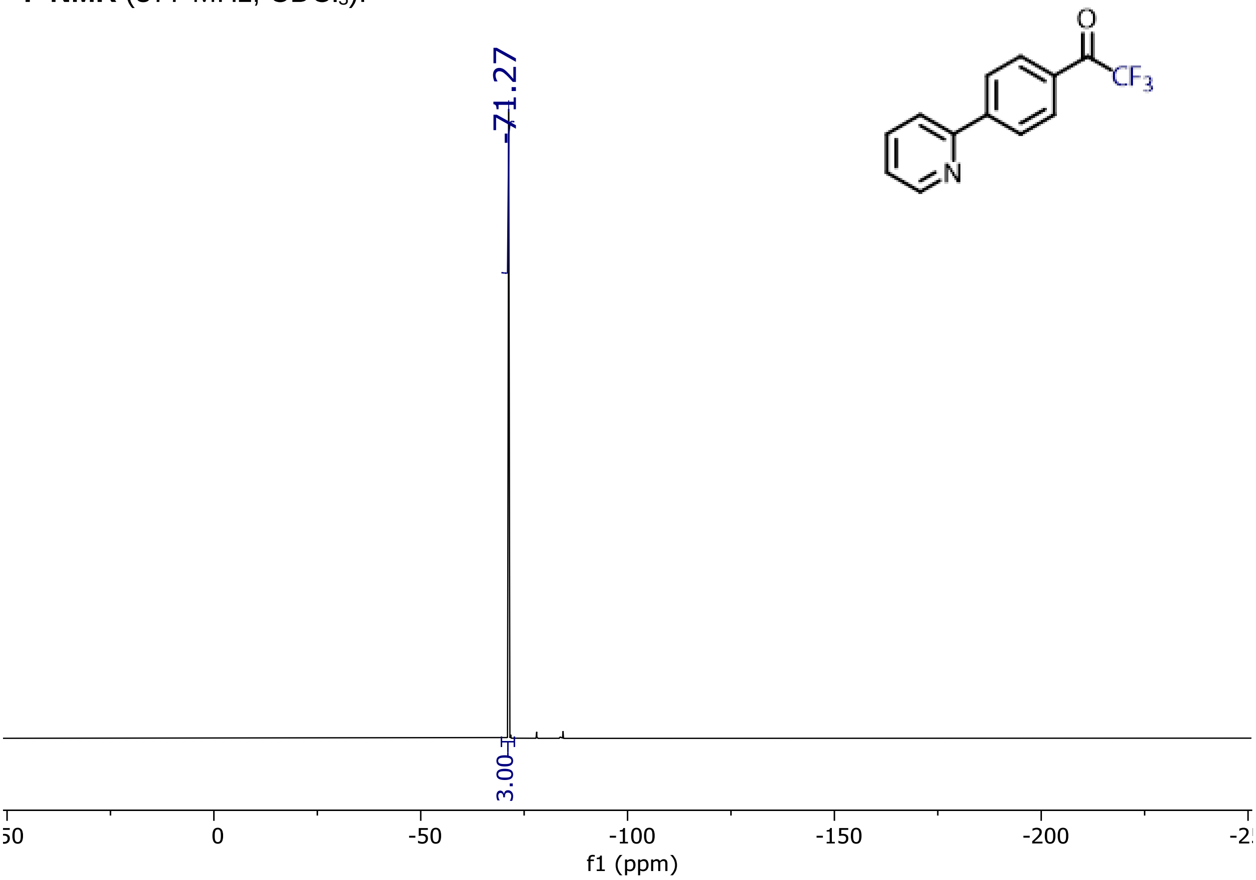

1-(4-(benzo[b]thiophen-3-yl)phenyl)-2,2,2-trifluoroethan-1-one, **1n**

^{1}H NMR (400 MHz, $CDCl_3$):

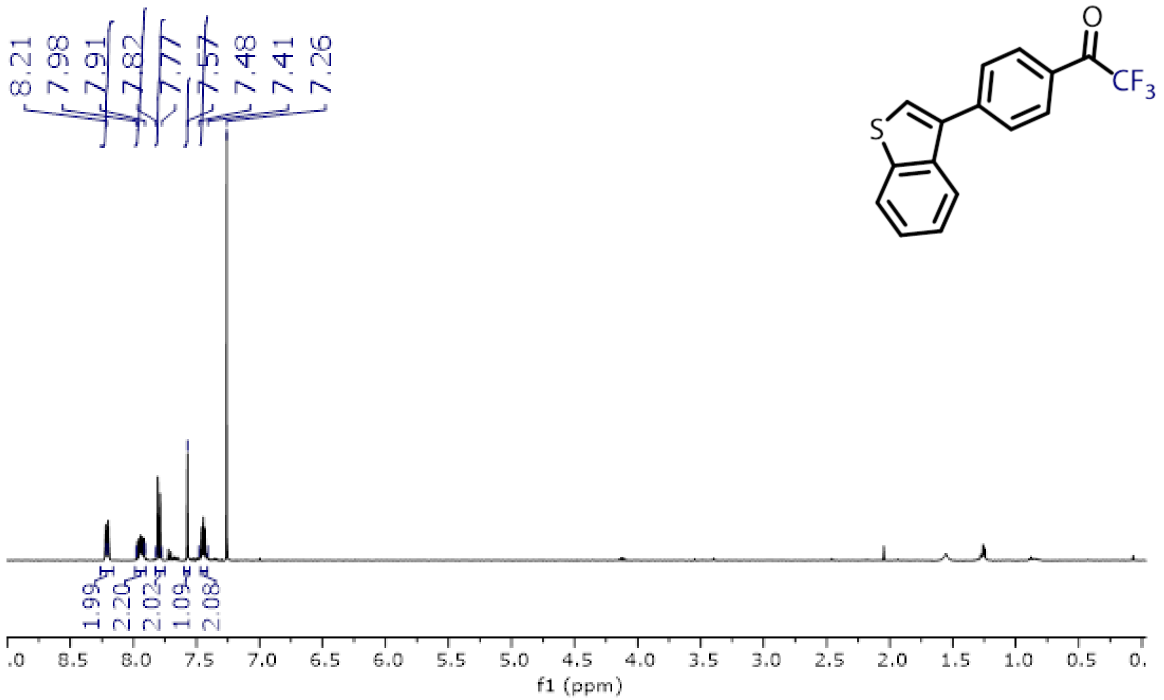

^{13}C NMR (131 MHz, $CDCl_3$):

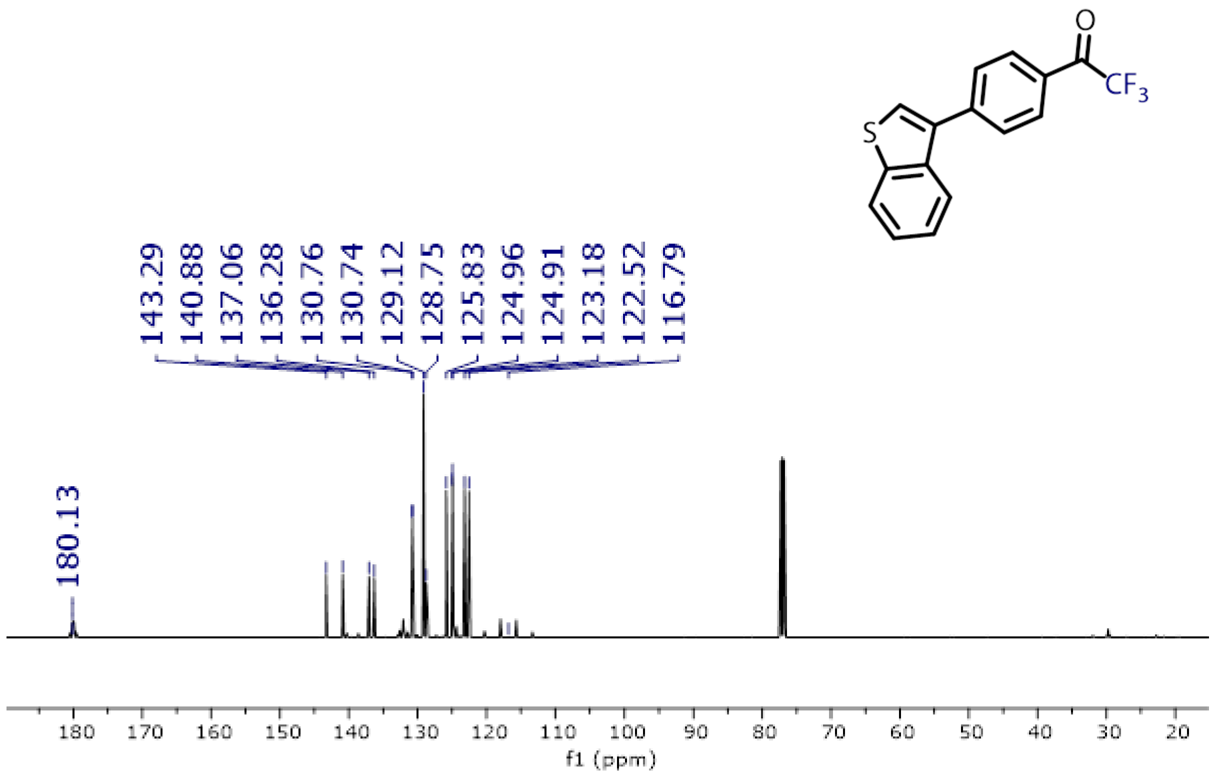

^{19}F NMR (376 MHz, $CDCl_3$):

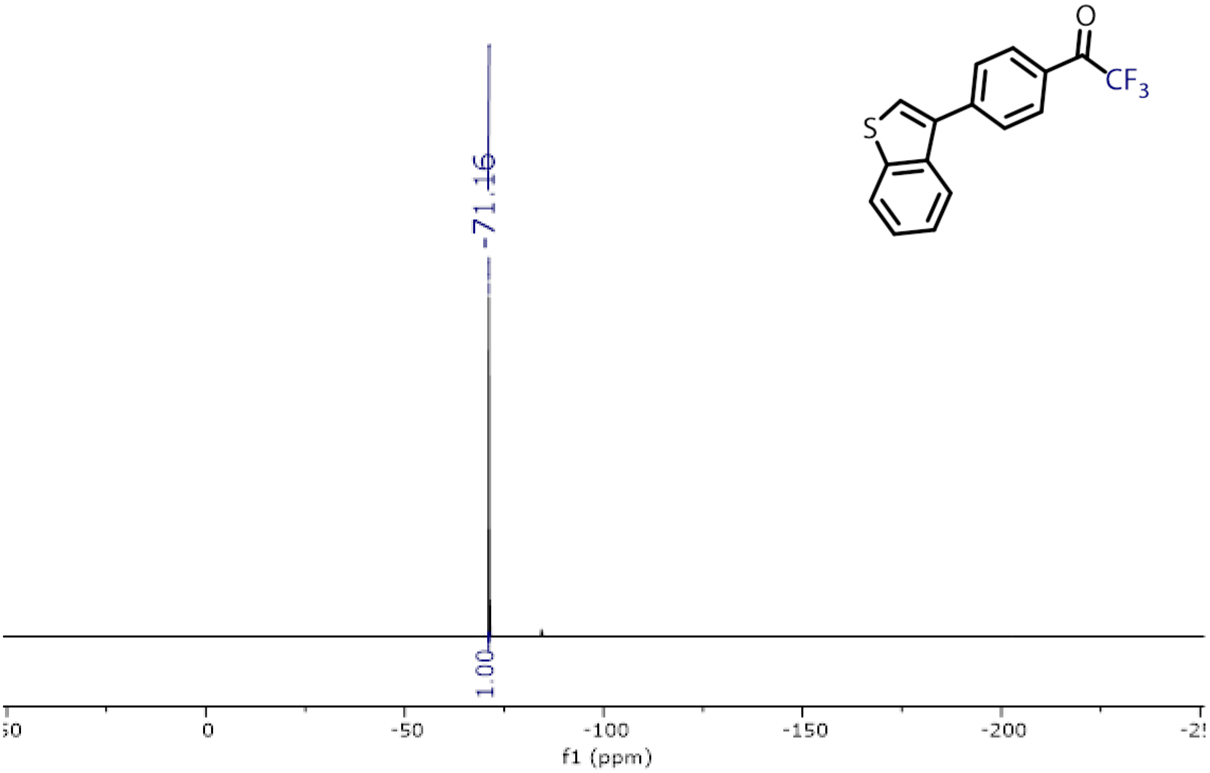

1-(3'-amino-[1,1'-biphenyl]-4-yl)-2,2,2-trifluoroethan-1-one **1o**

^{1}H NMR (400 MHz, $CDCl_3$):

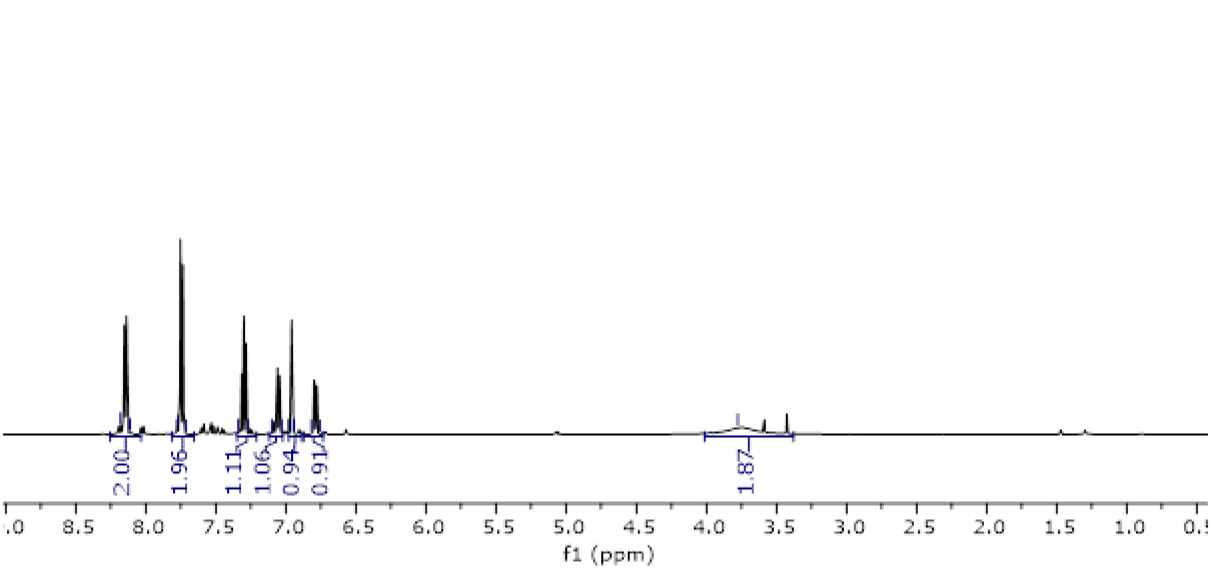

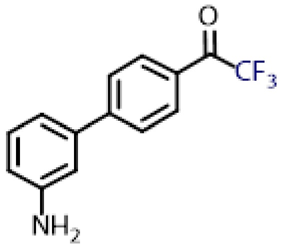

^{13}C NMR (131 MHz, $CDCl_3$):

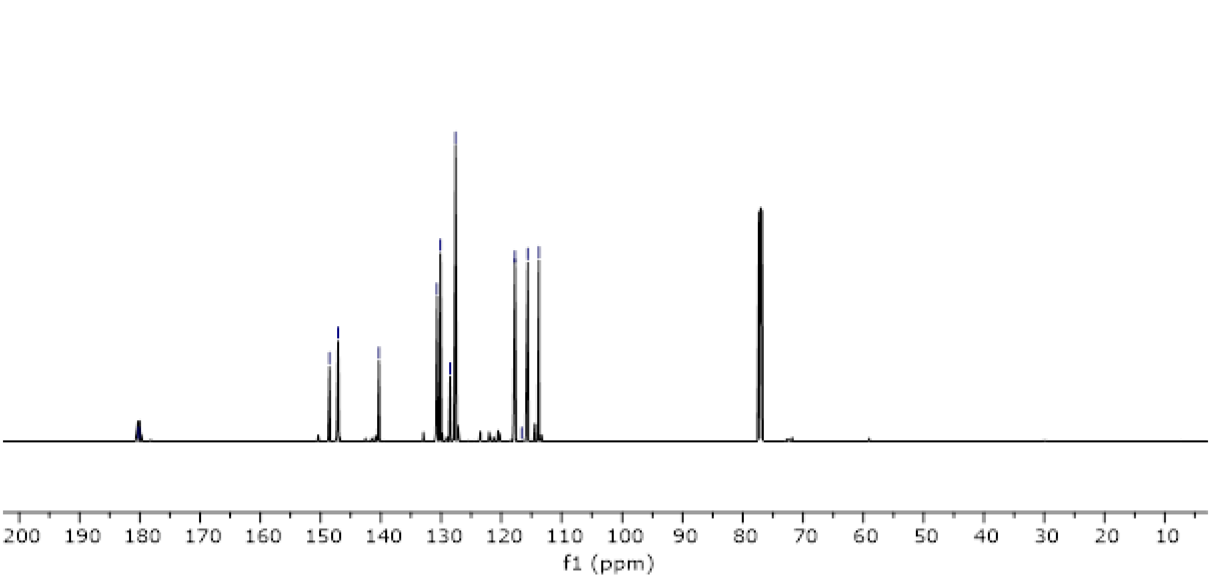

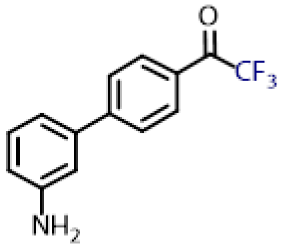

^{19}F NMR (376 MHz, $CDCl_3$):

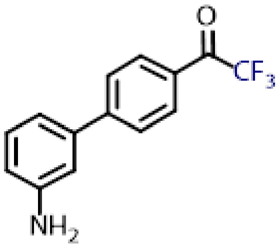

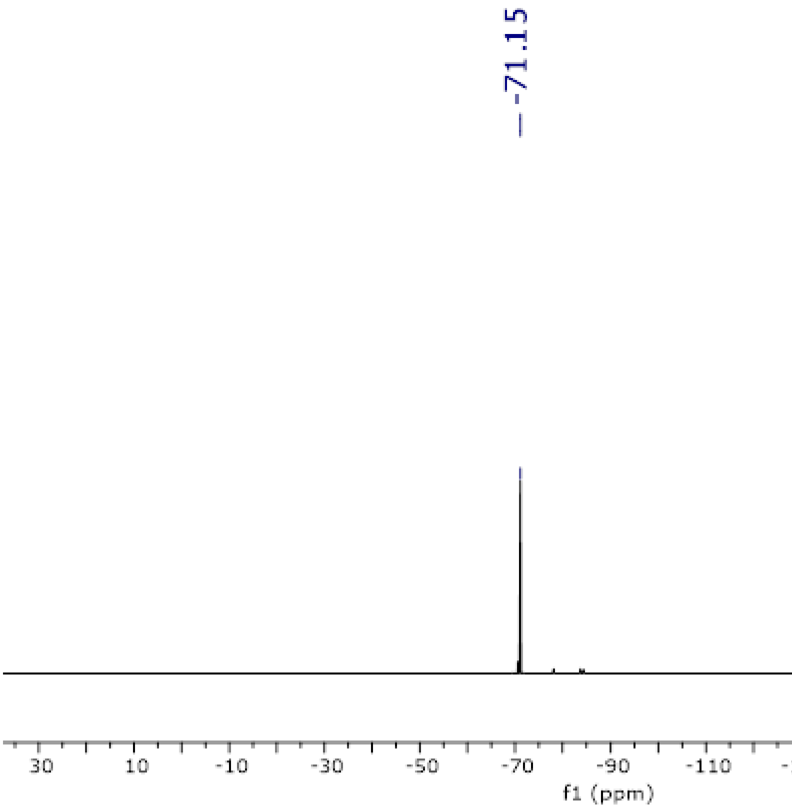

2,2,2-trifluoro-1-(4-(2-methoxypyrimidin-5-yl)phenyl)ethan-1-one, **1p**

^{1}H NMR (400 MHz, $CDCl_3$):

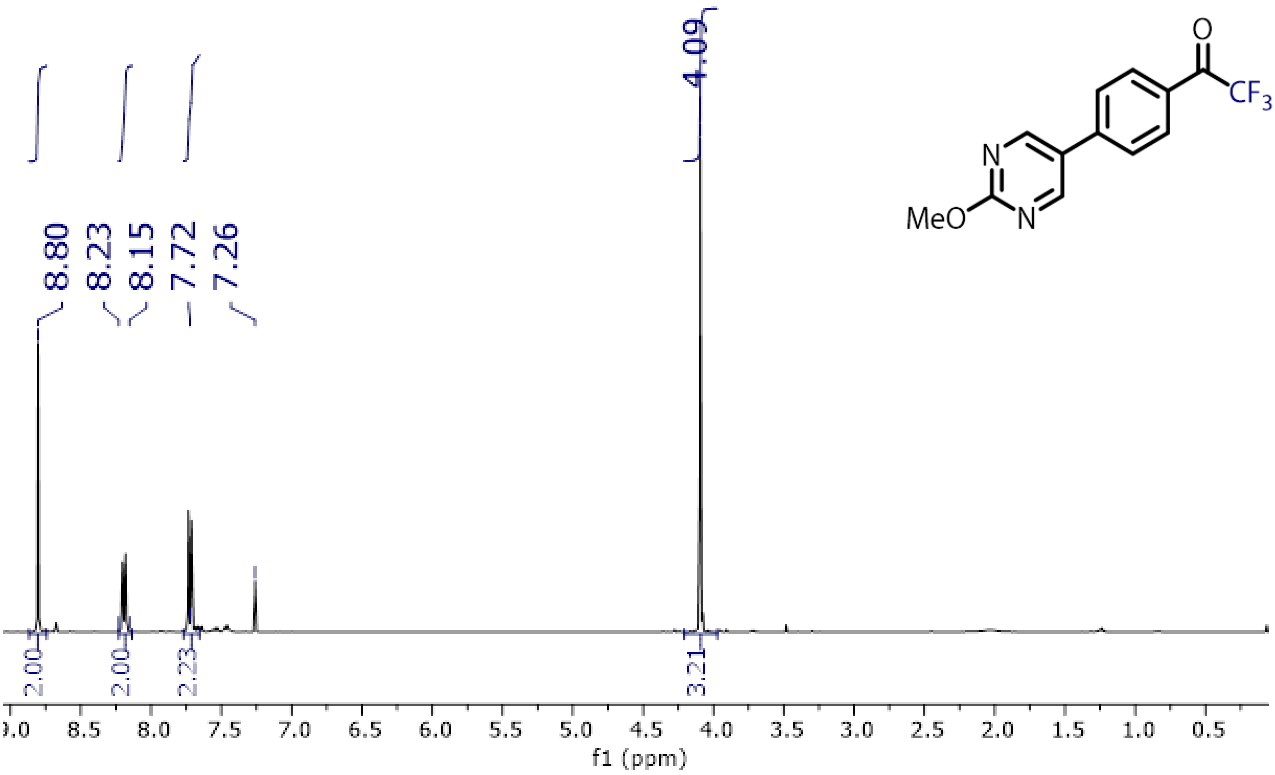

^{13}C NMR (131 MHz, $CDCl_3$):

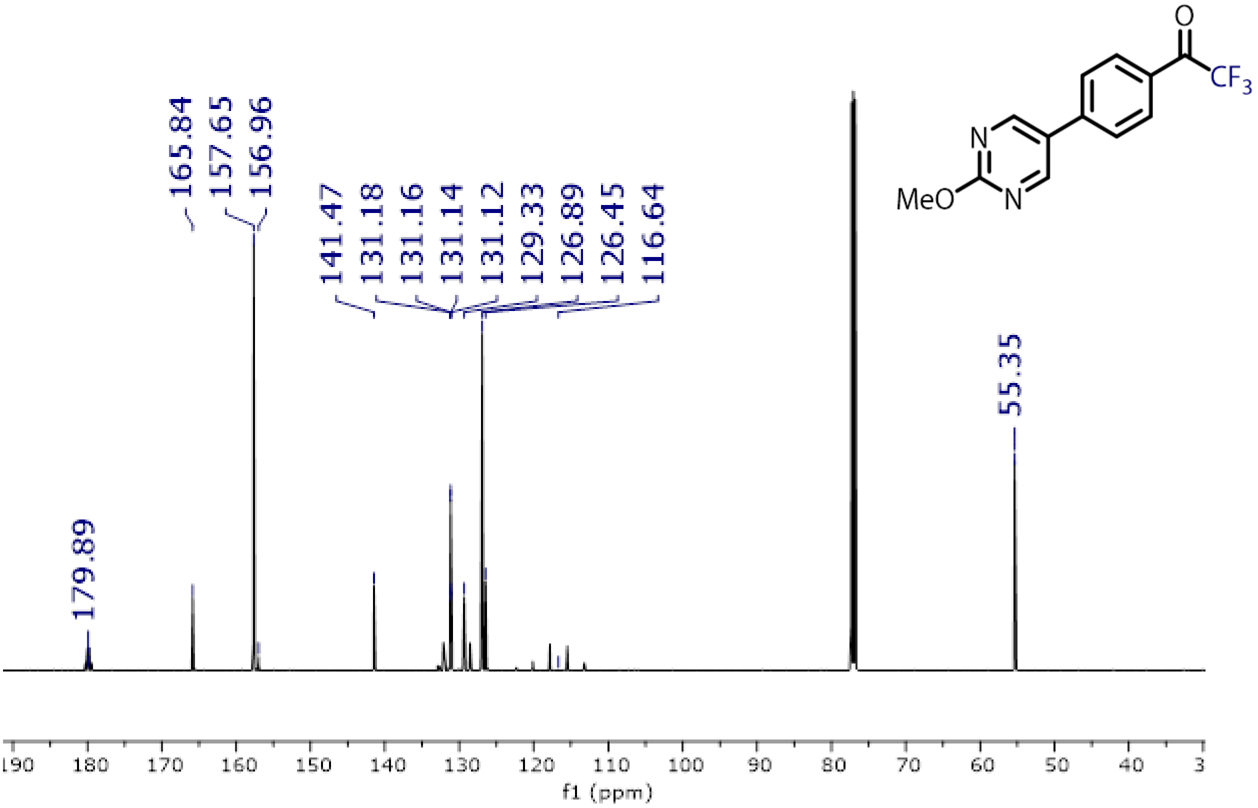

^{19}F NMR (376 MHz, $CDCl_3$):

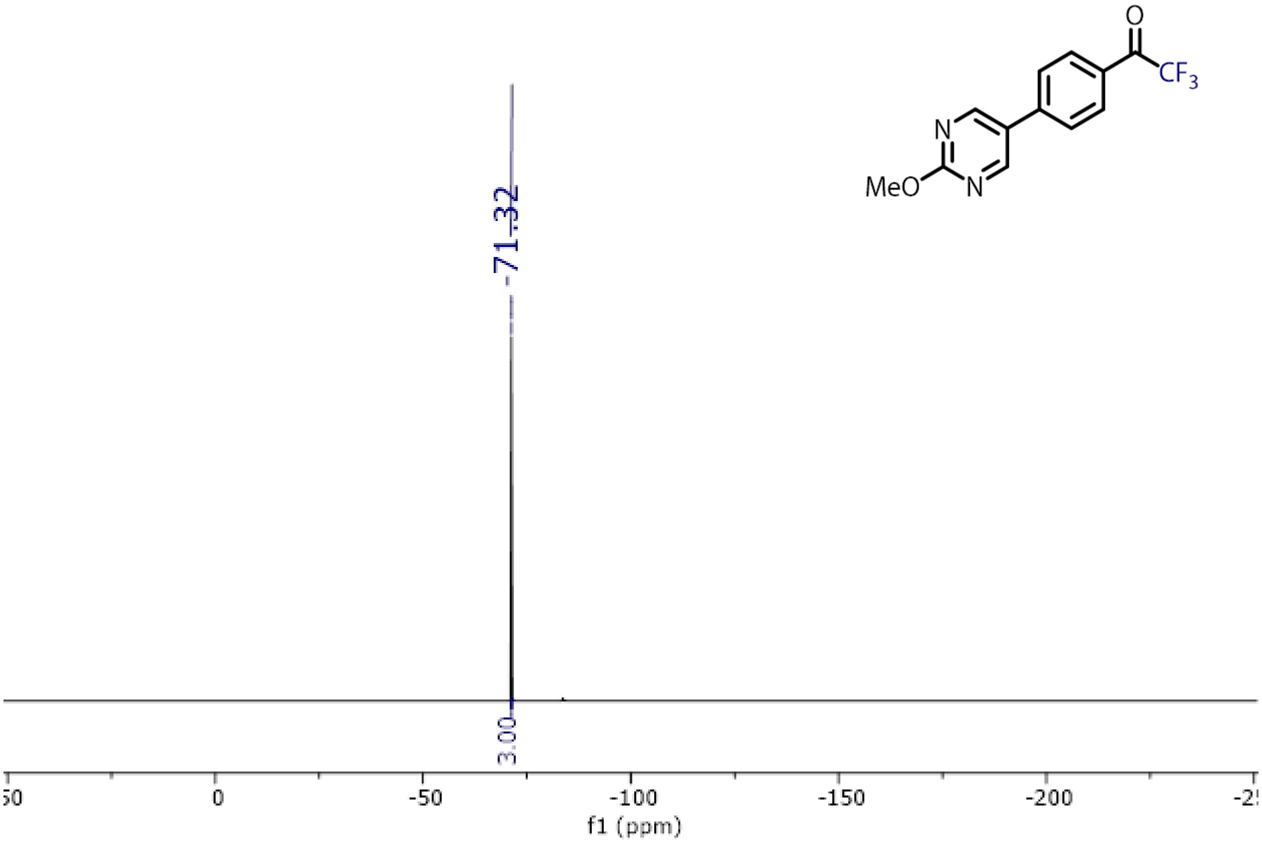

1-(3-(2,6-diphenylpyrimidin-4-yl)phenyl)-2,2,2-trifluoroethan-1-one, **1q**

^{1}H NMR (400 MHz, $CDCl_3$):

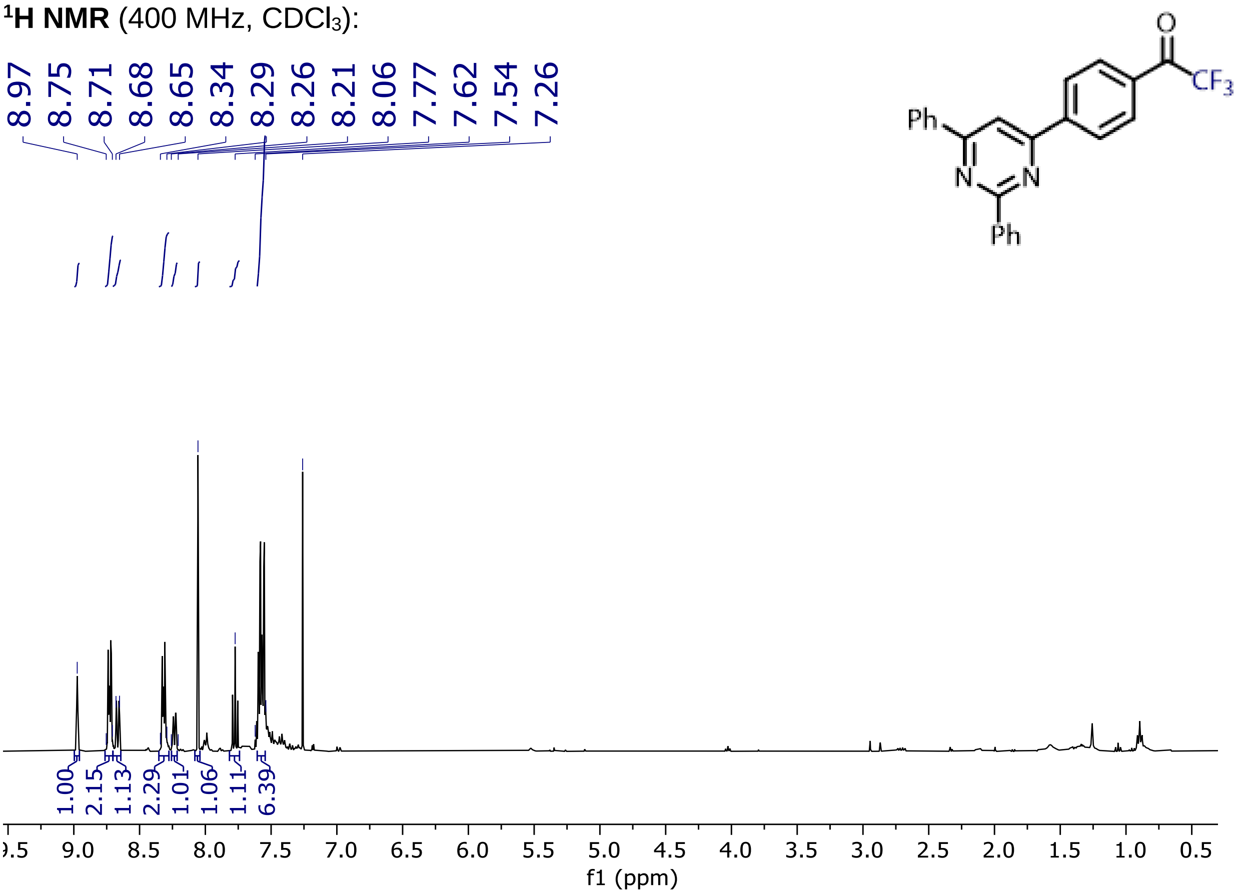

^{13}C NMR (101 MHz, $CDCl_3$):

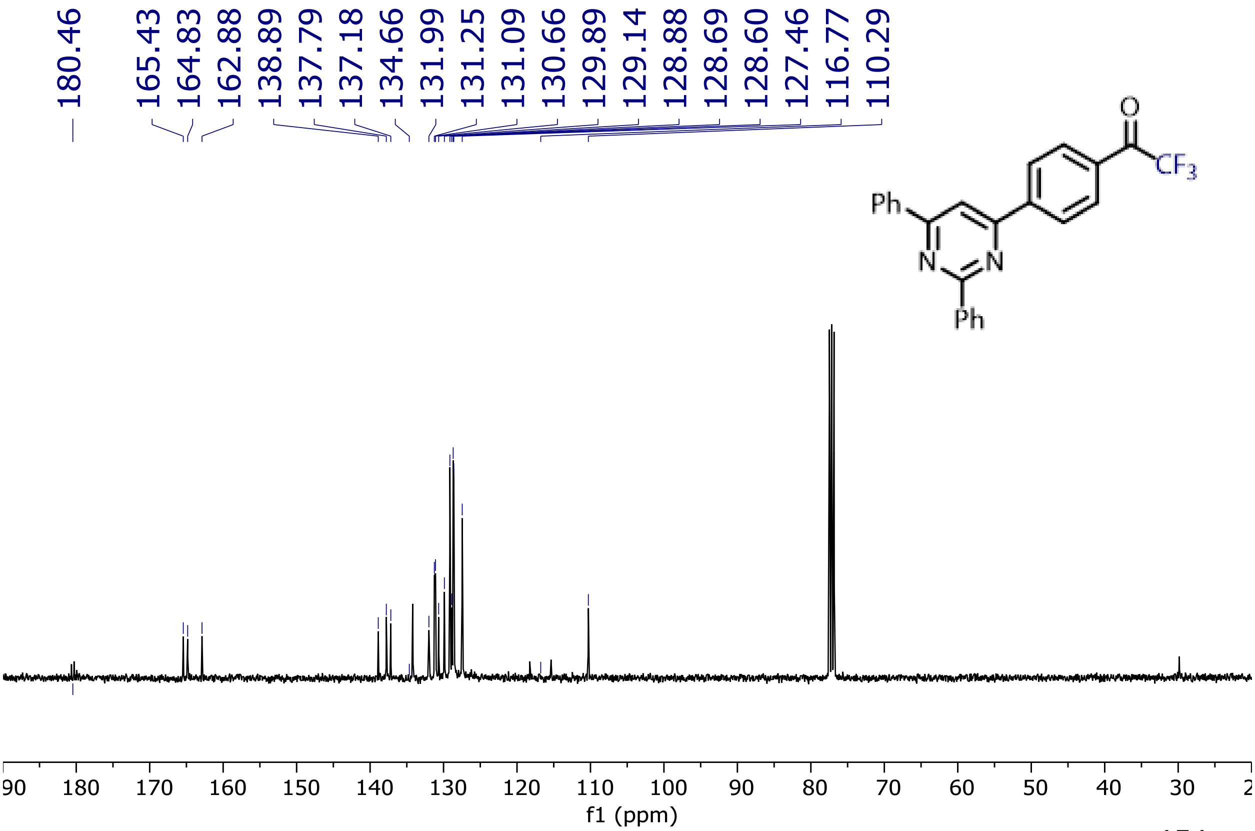

19F NMR (377 MHz, $CDCl_3$):

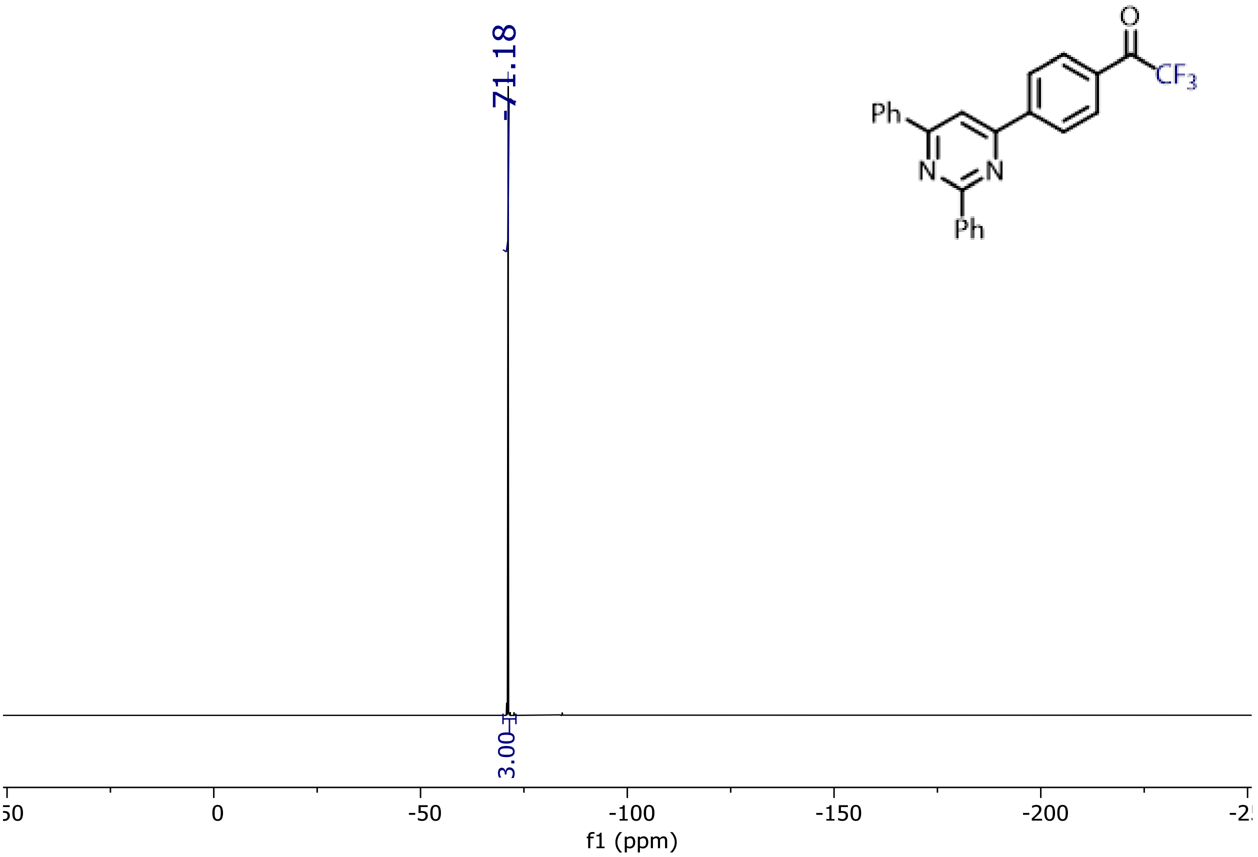

(E)-2,2,2-trifluoro-1-(4-styrylphenyl)ethan-1-one, **1s**

^{1}**H NMR** (400 MHz, $CDCl_3$):

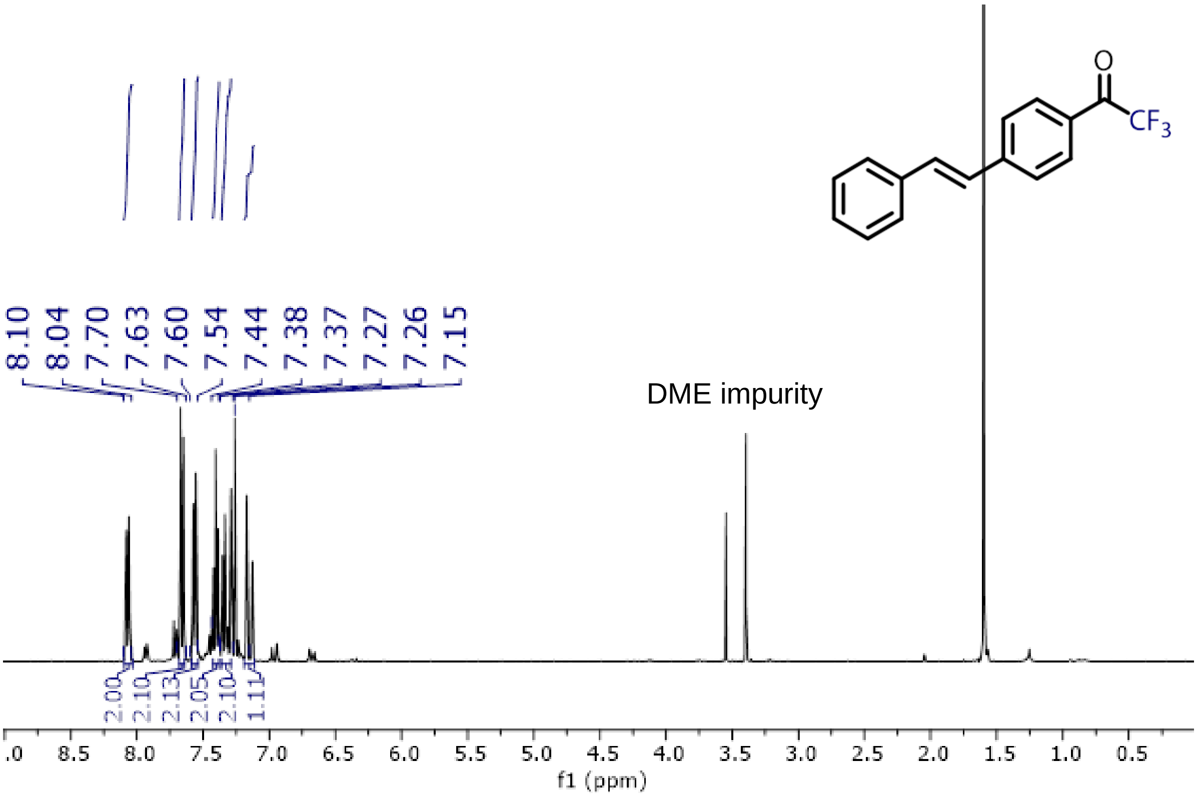

^{13}**C NMR** (131 MHz, $CDCl_3$):

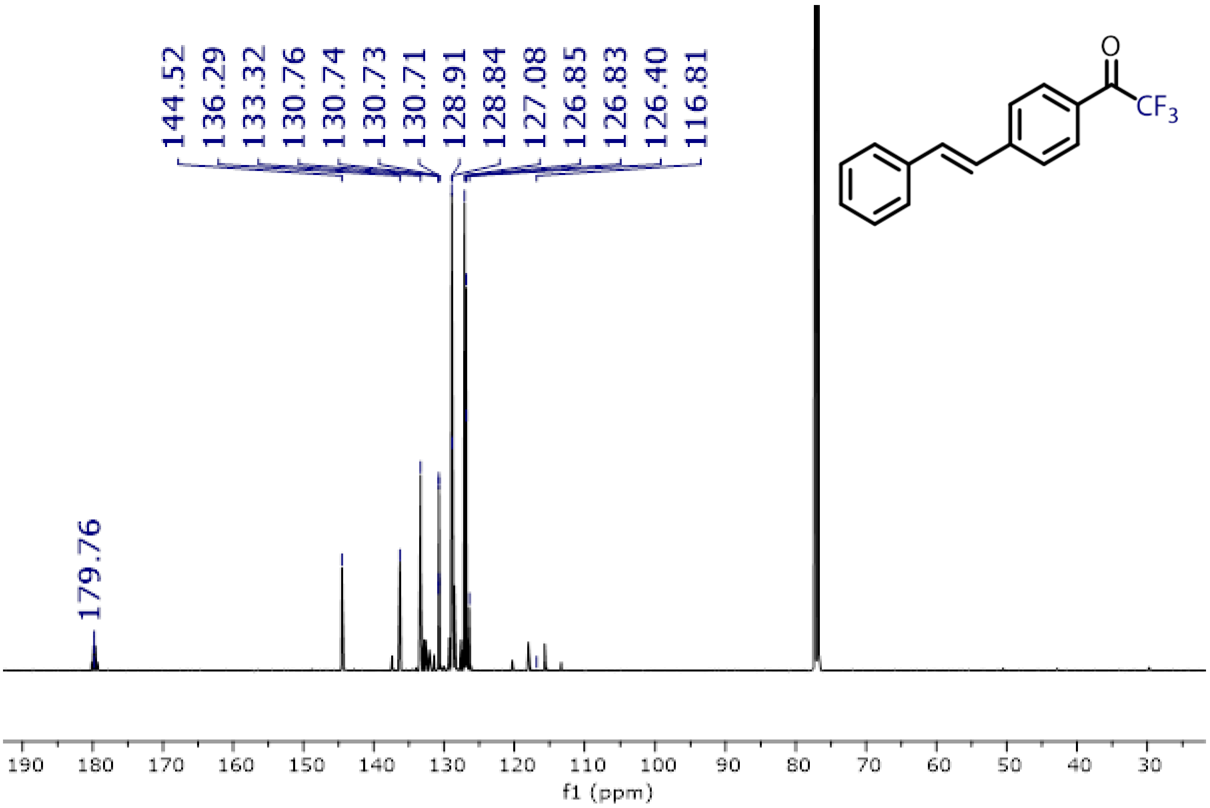

19F NMR (376 MHz, $CDCl_3$):

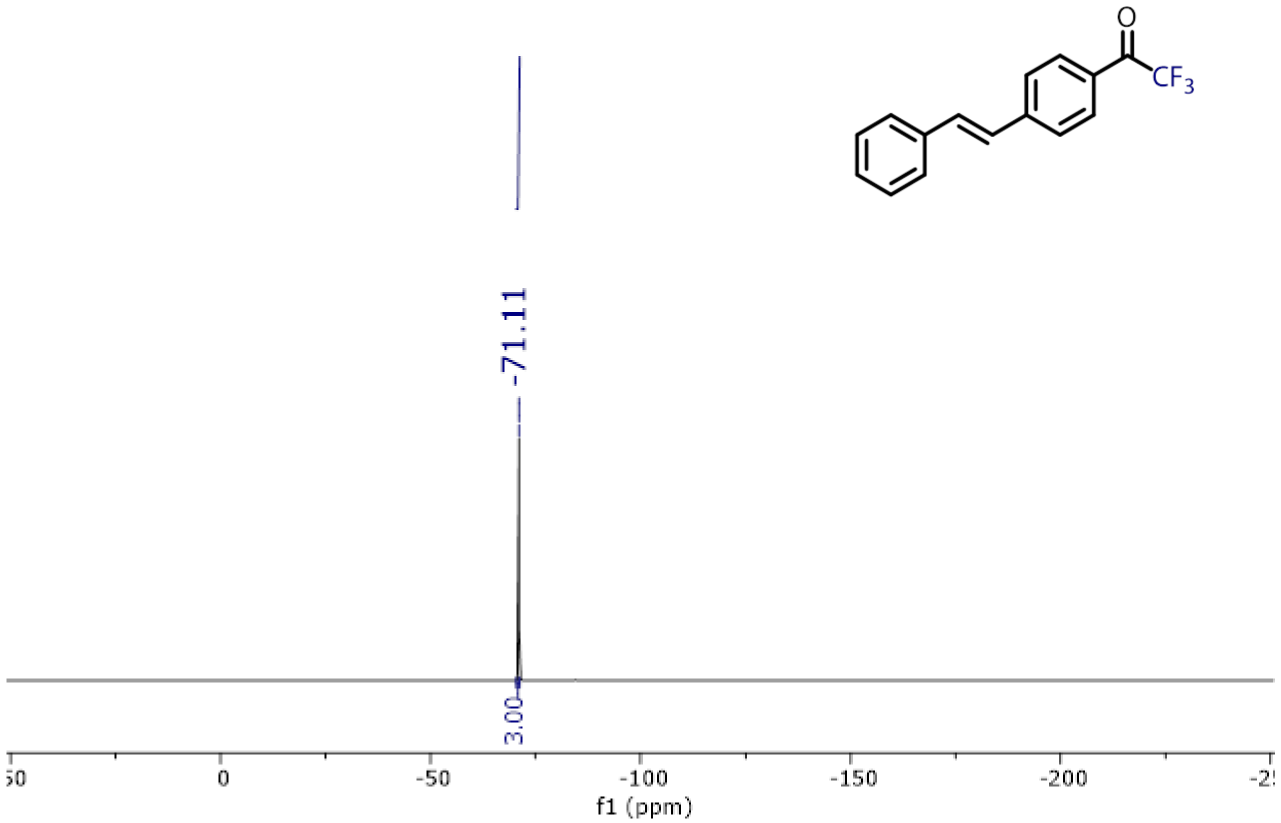

2,2,2-trifluoro-1-(1-(perfluoropyridin-4-yl)-1H-indol-3-yl)ethan-1-one, **1x**

^{1}H NMR (400 MHz, $CDCl_3$):

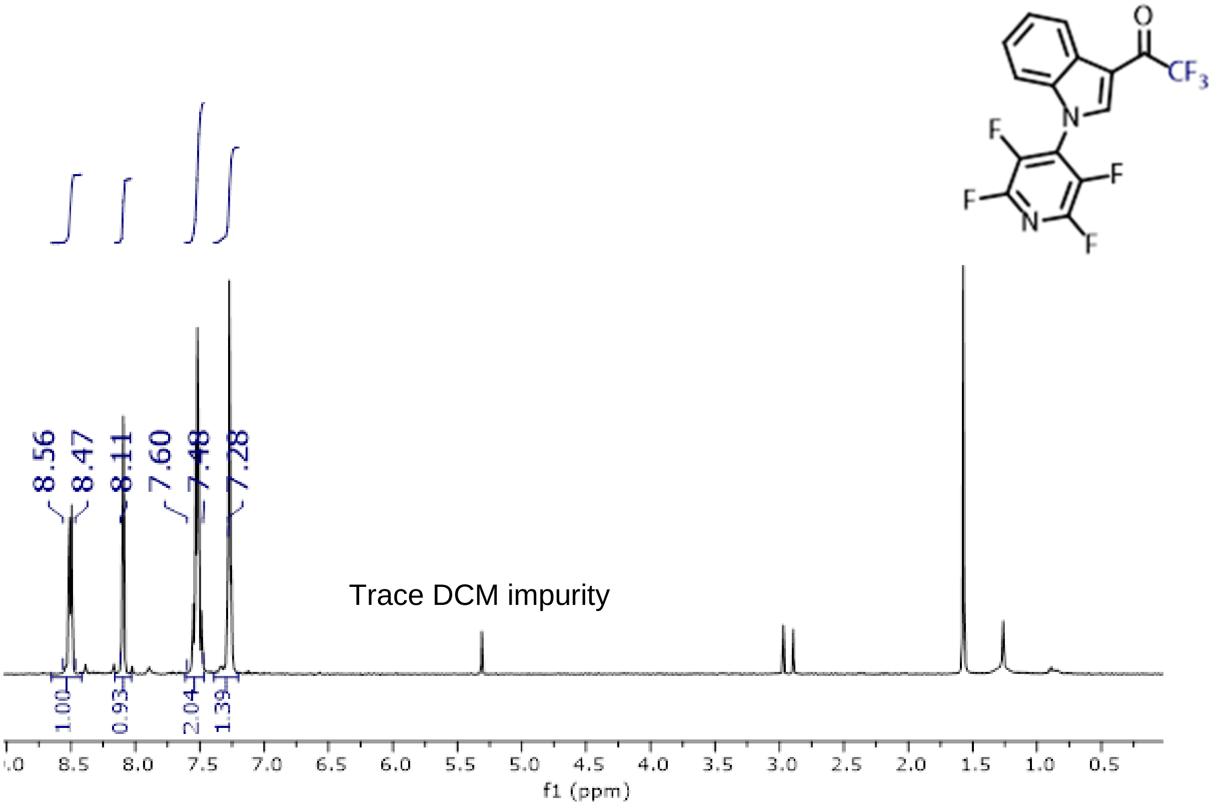

^{13}C NMR (131 MHz, $CDCl_3$):

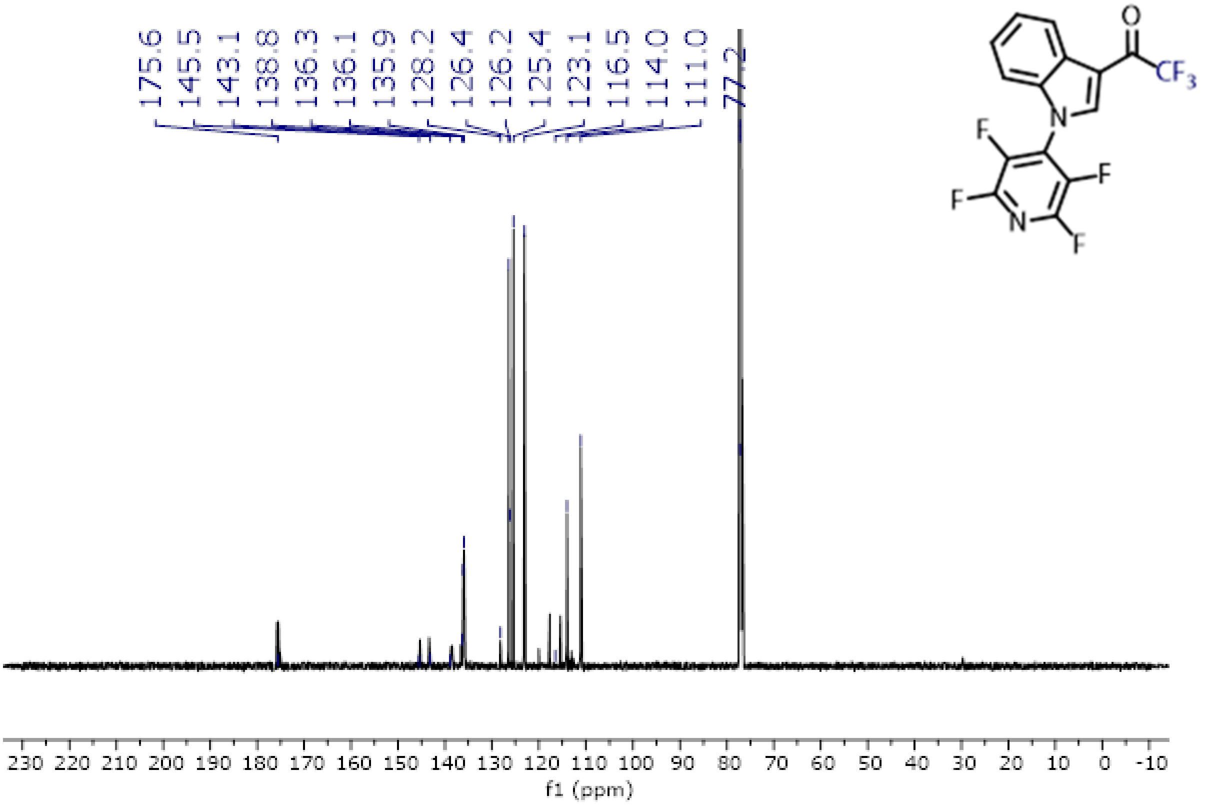

^{19}F NMR (376 MHz, $CDCl_3$):

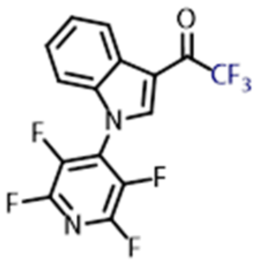

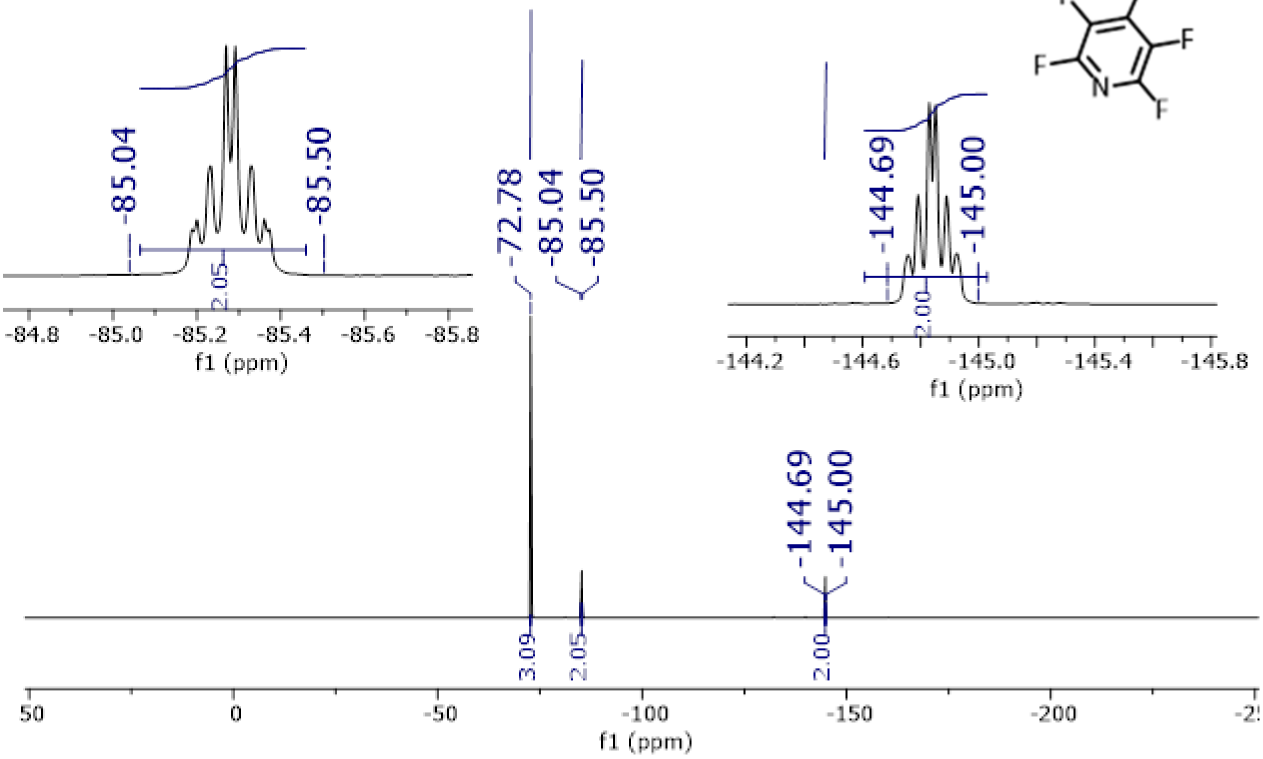

1-methyl-3-(2,2,2-trifluoroacetyl)-1H-indole-5-carbonitrile, **1ab**

^{1}H NMR (400 MHz, $CDCl_3$):

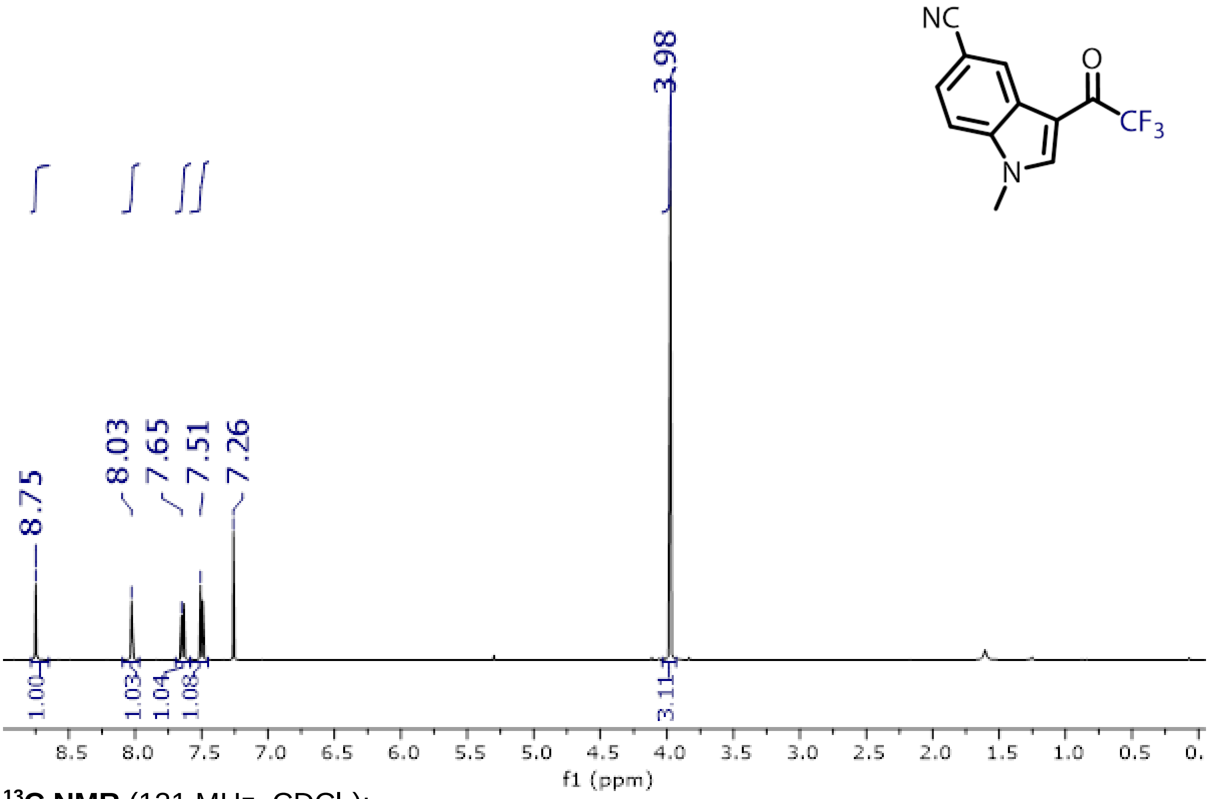

^{13}C NMR (131 MHz, $CDCl_3$):

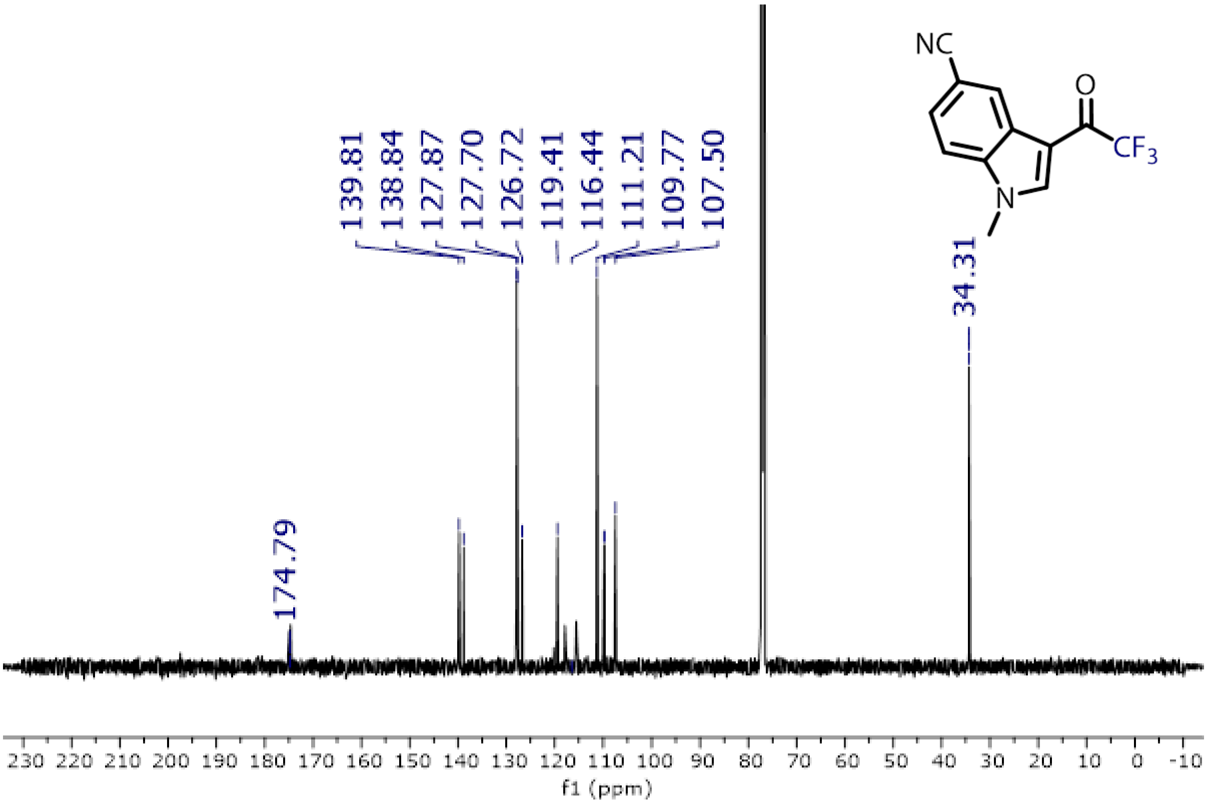

^{19}F NMR (376 MHz, $CDCl_3$):

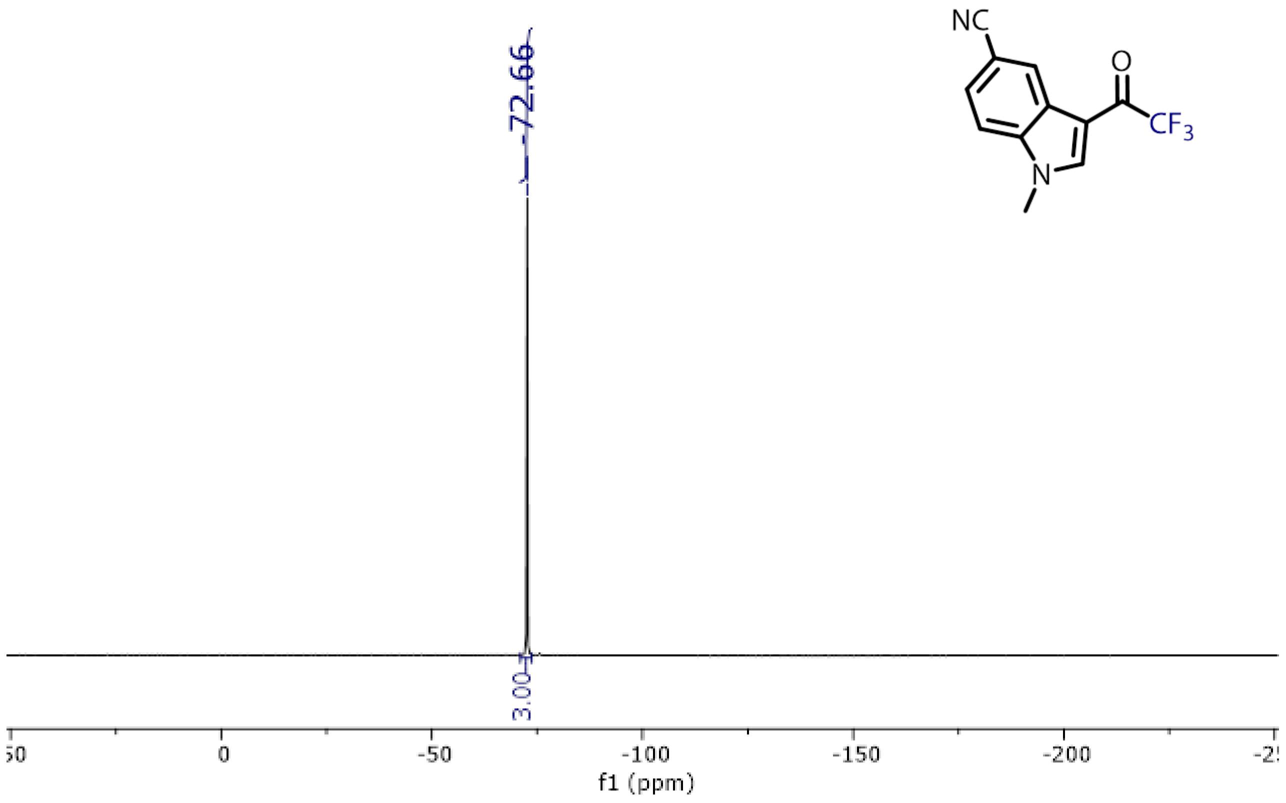

1-(4-chloro-1-methyl-1H-indol-3-yl)-2,2,2-trifluoroethan-1-one, **1ad**

^{1}H NMR (400 MHz, $CDCl_3$):

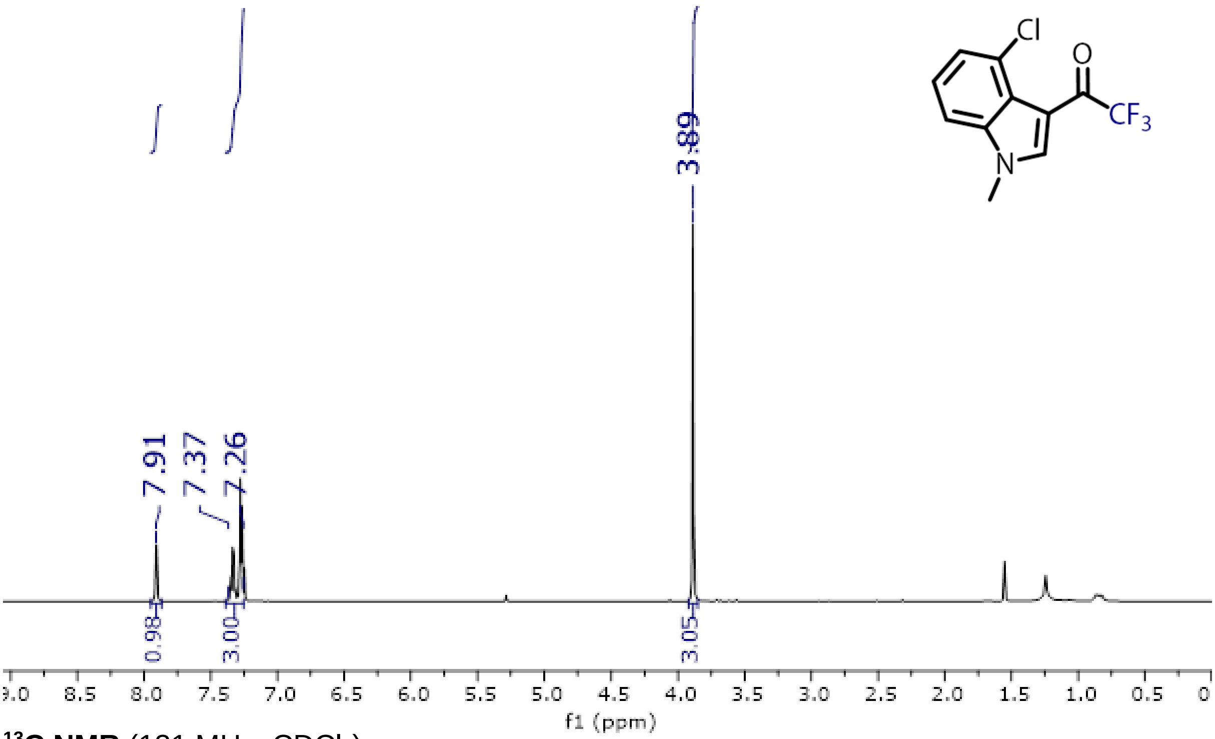

^{13}C NMR (131 MHz, $CDCl_3$):

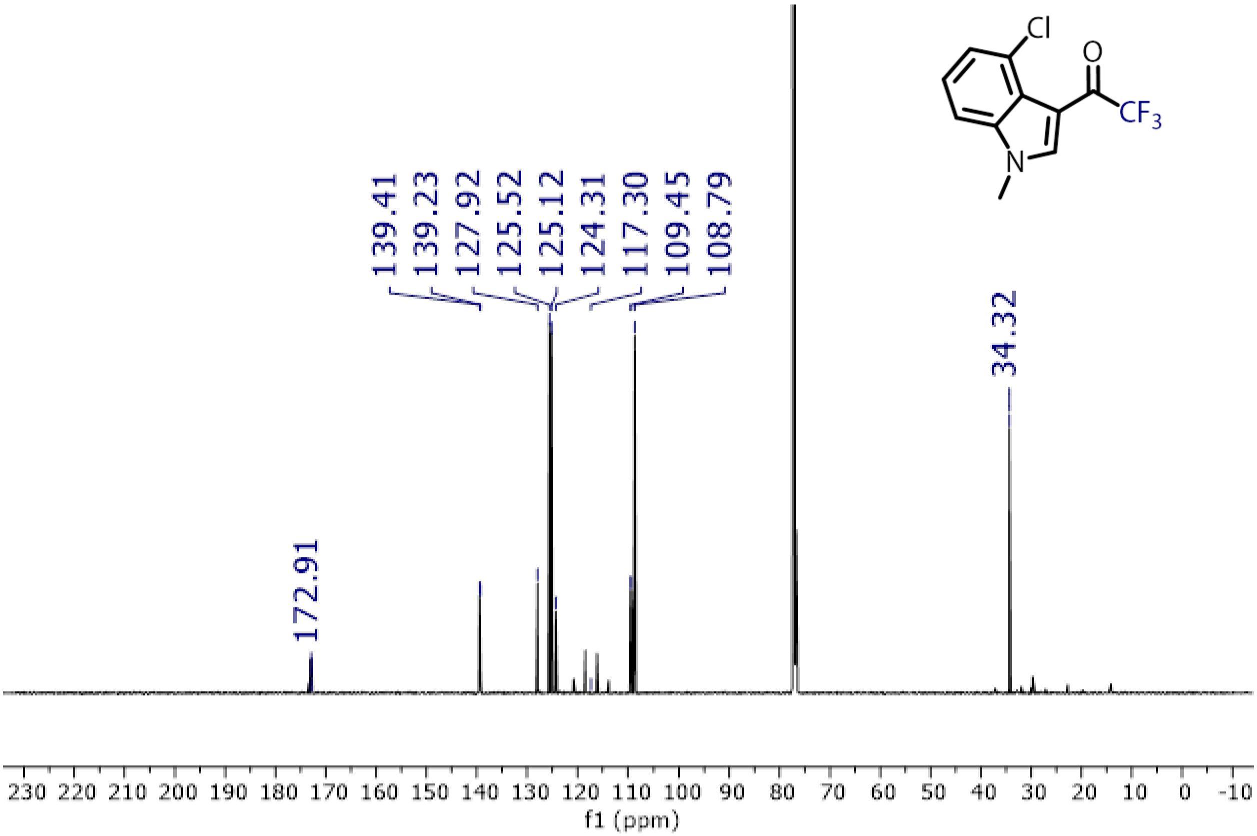

^{19}F NMR (376 MHz, $CDCl_3$):

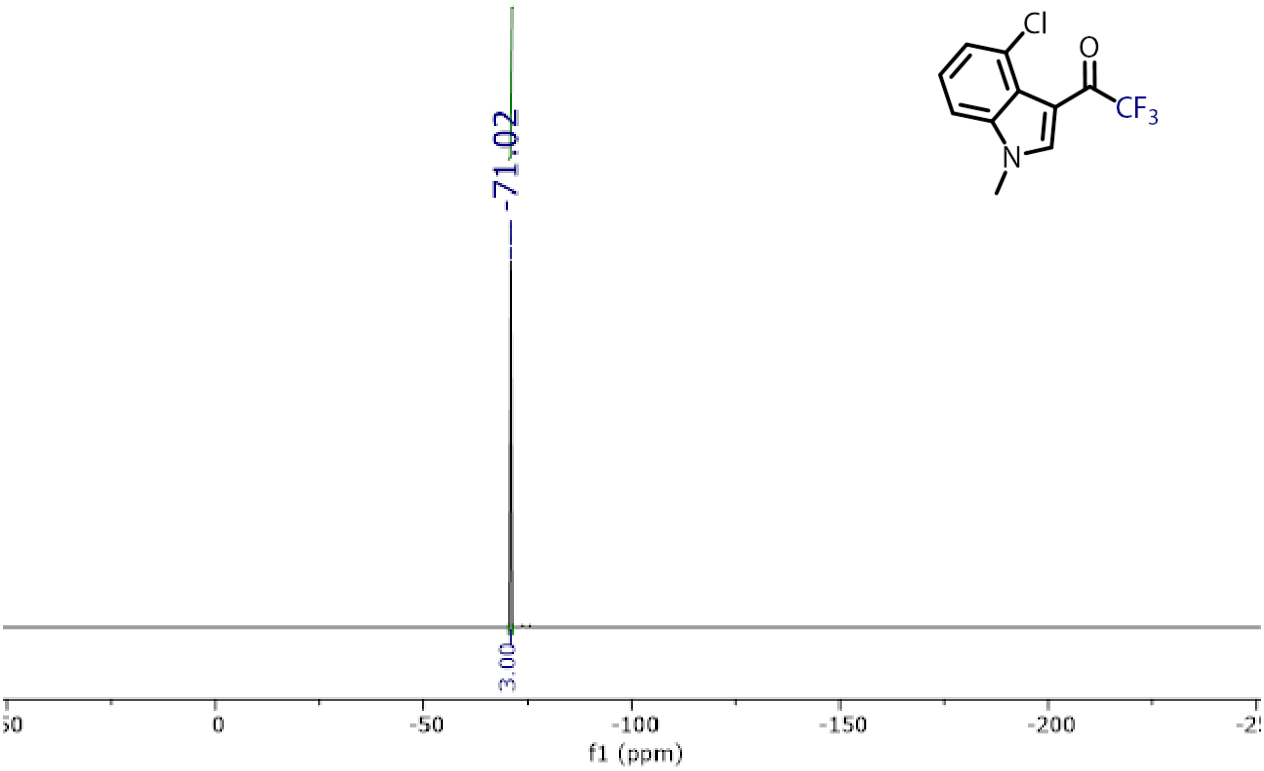

1-(4-bromo-1-methyl-1H-indol-3-yl)-2,2,2-trifluoroethan-1-one, **1ae**

^{1}H NMR (400 MHz, $CDCl_3$):

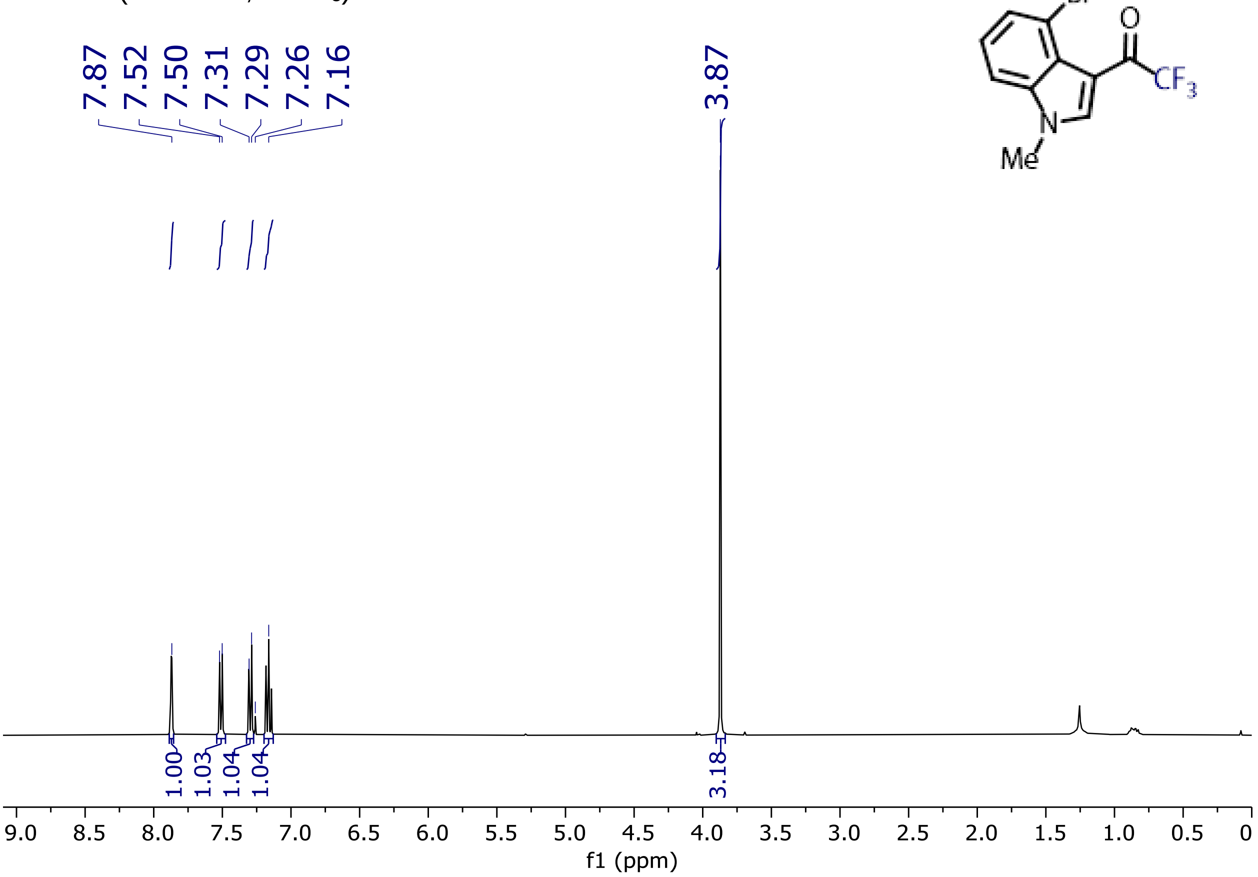

^{13}C NMR (101 MHz, $CDCl_3$):

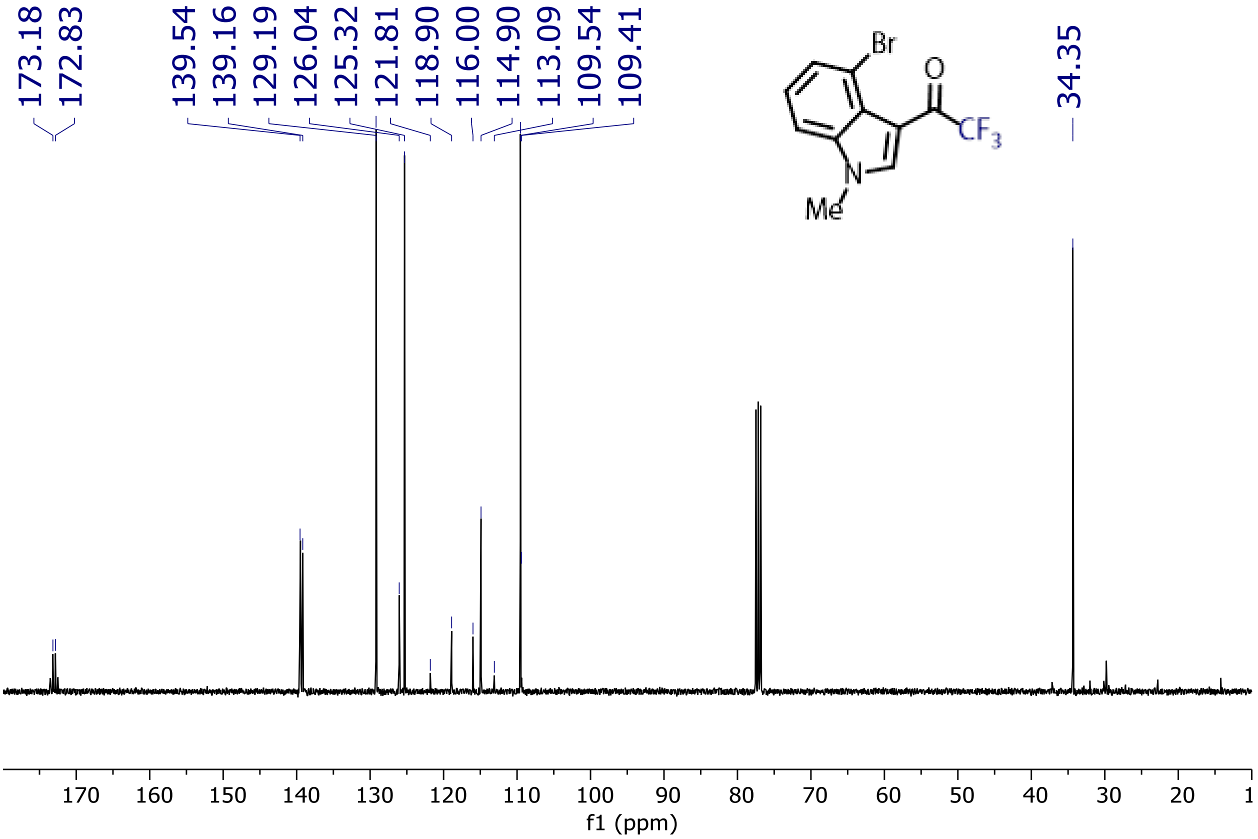

^{19}F NMR (376 MHz, $CDCl_3$):

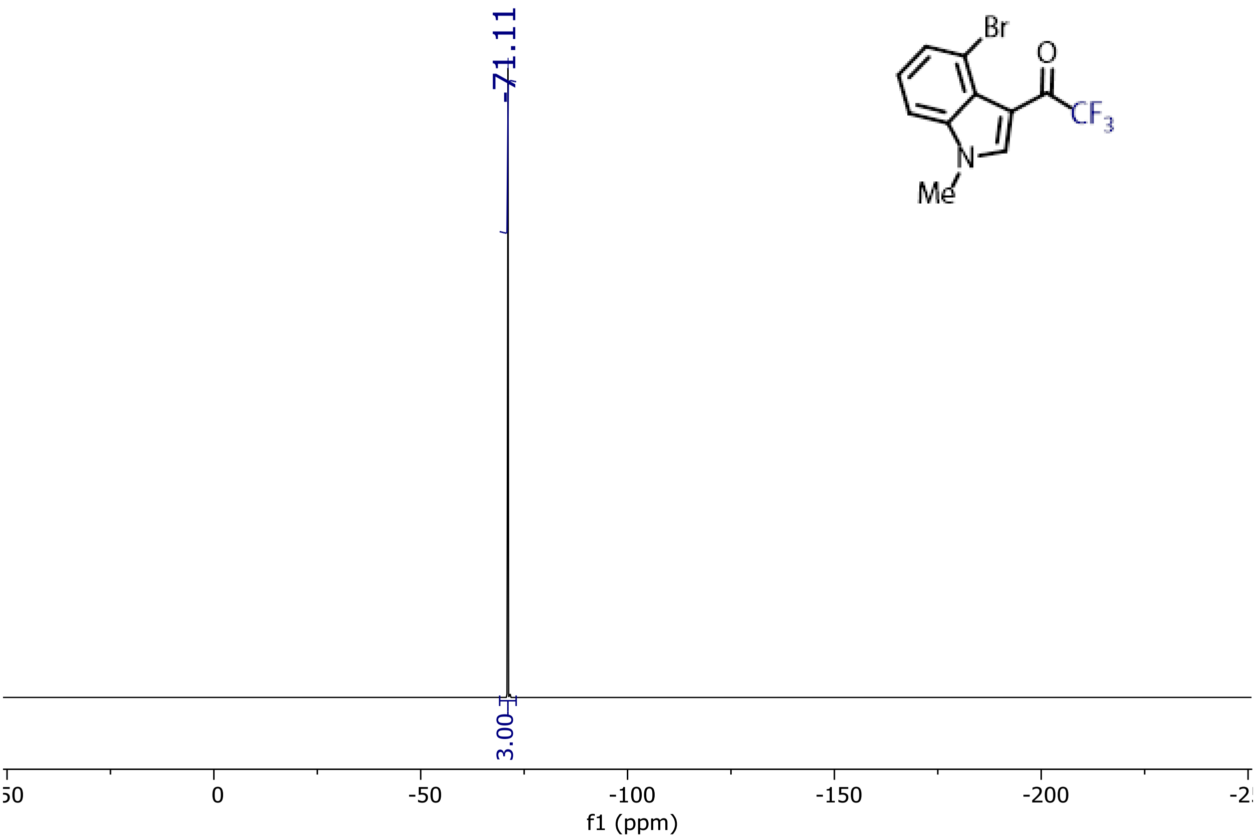

2,2,2-trifluoro-1-(5-iodo-1-methyl-1H-indol-3-yl)ethan-1-one, **1ag**

^{1}H NMR (400 MHz, $CDCl_3$):

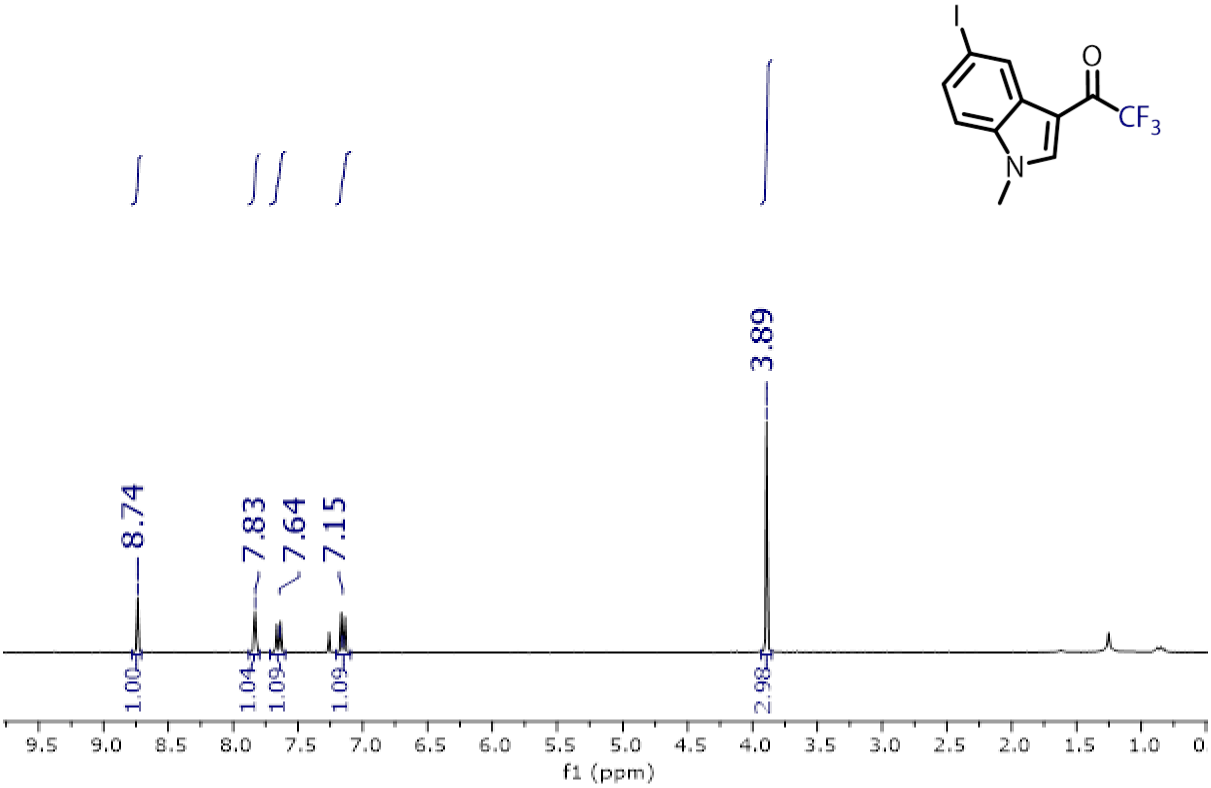

^{13}C NMR (131 MHz, $CDCl_3$):

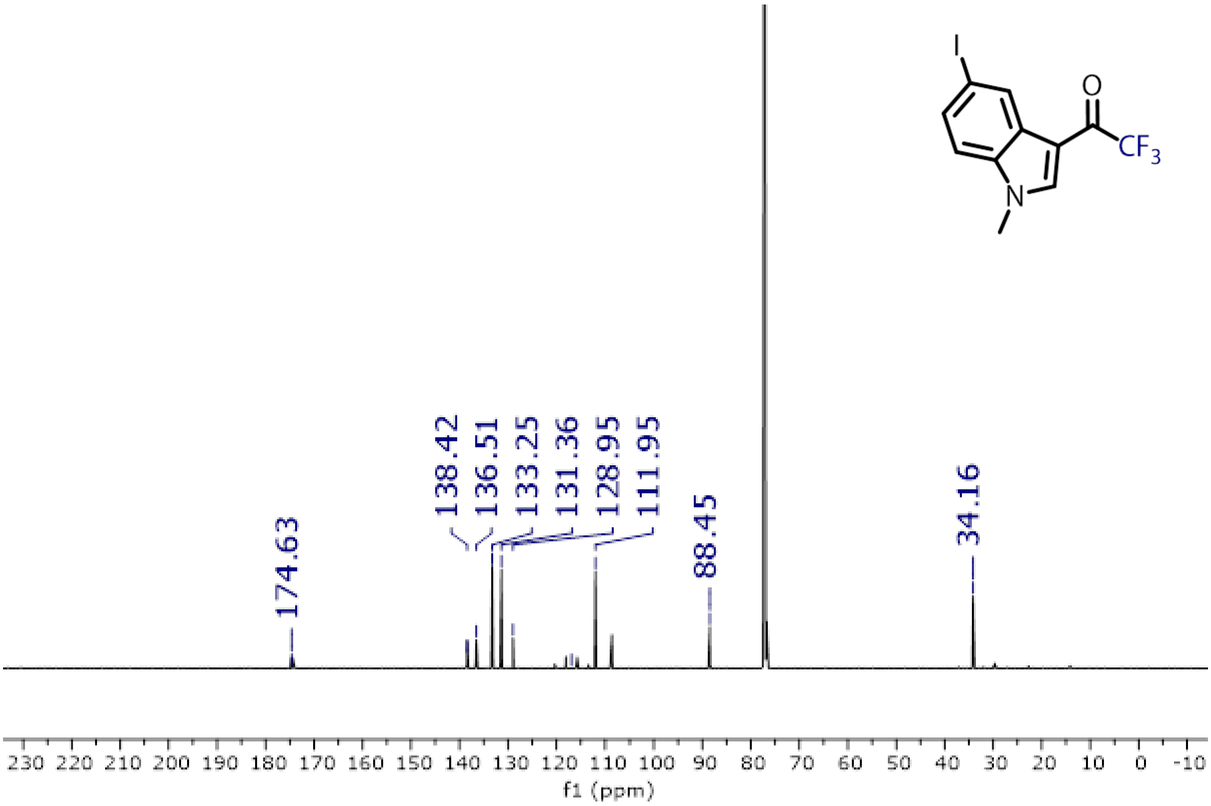

^{19}F NMR (376 MHz, $CDCl_3$):

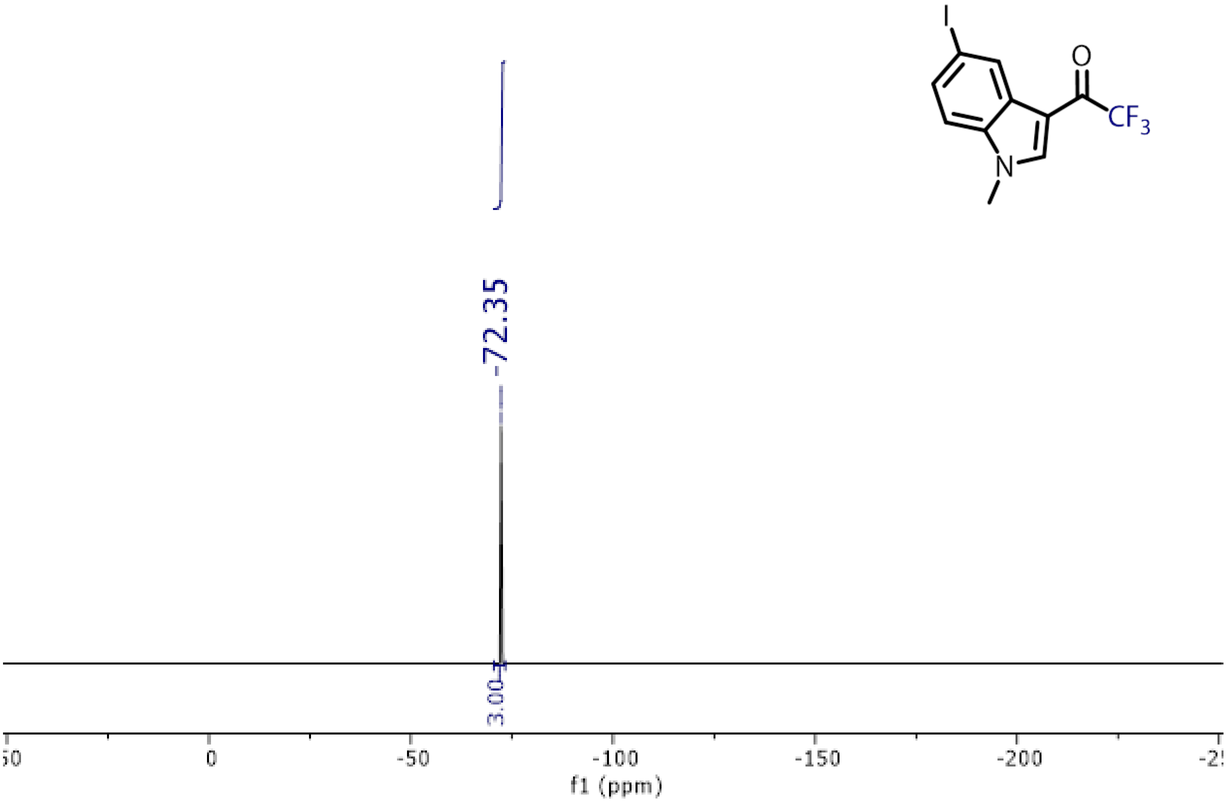

2,2,2-trifluoro-1-(1-methyl-2-(thiophen-2-yl)-1H-indol-3-yl)ethan-1-one, **1ah**

^{1}H NMR (400 MHz, $CDCl_3$):

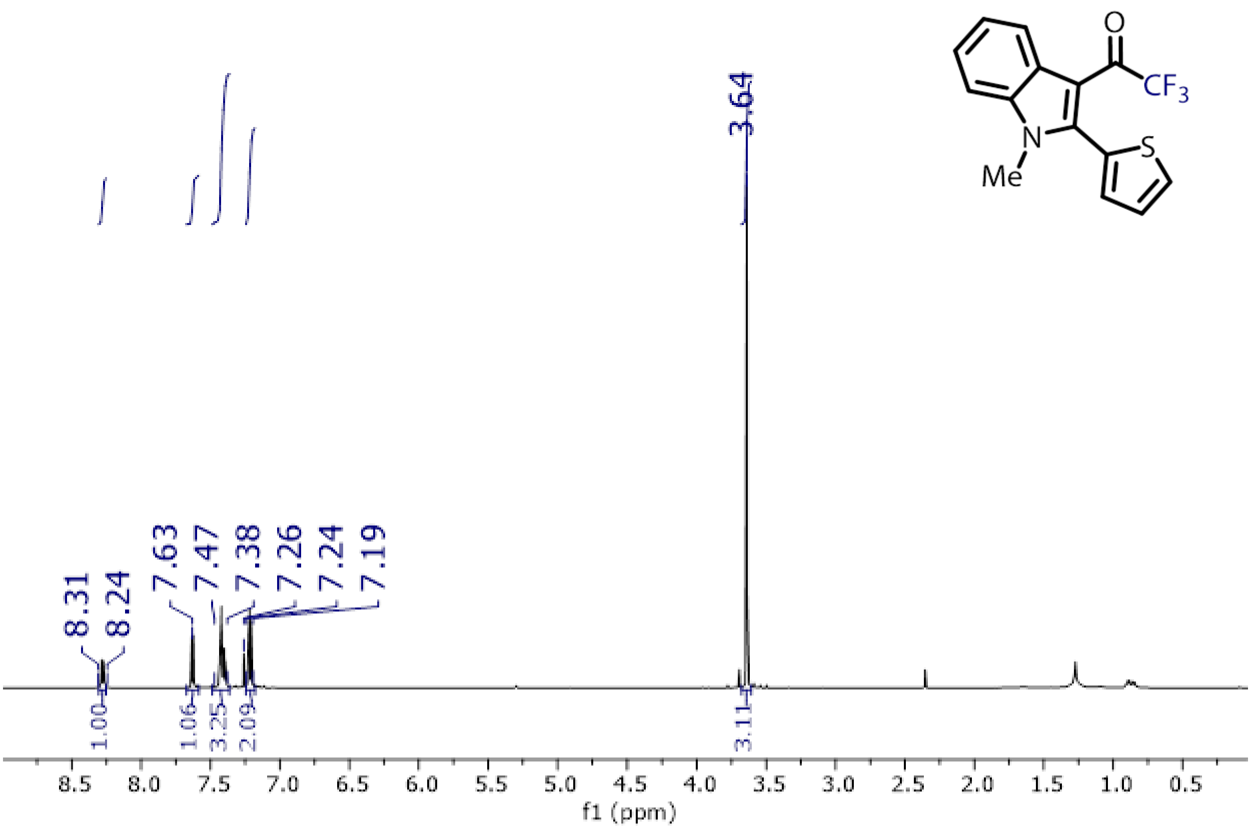

^{13}C NMR (131 MHz, $CDCl_3$):

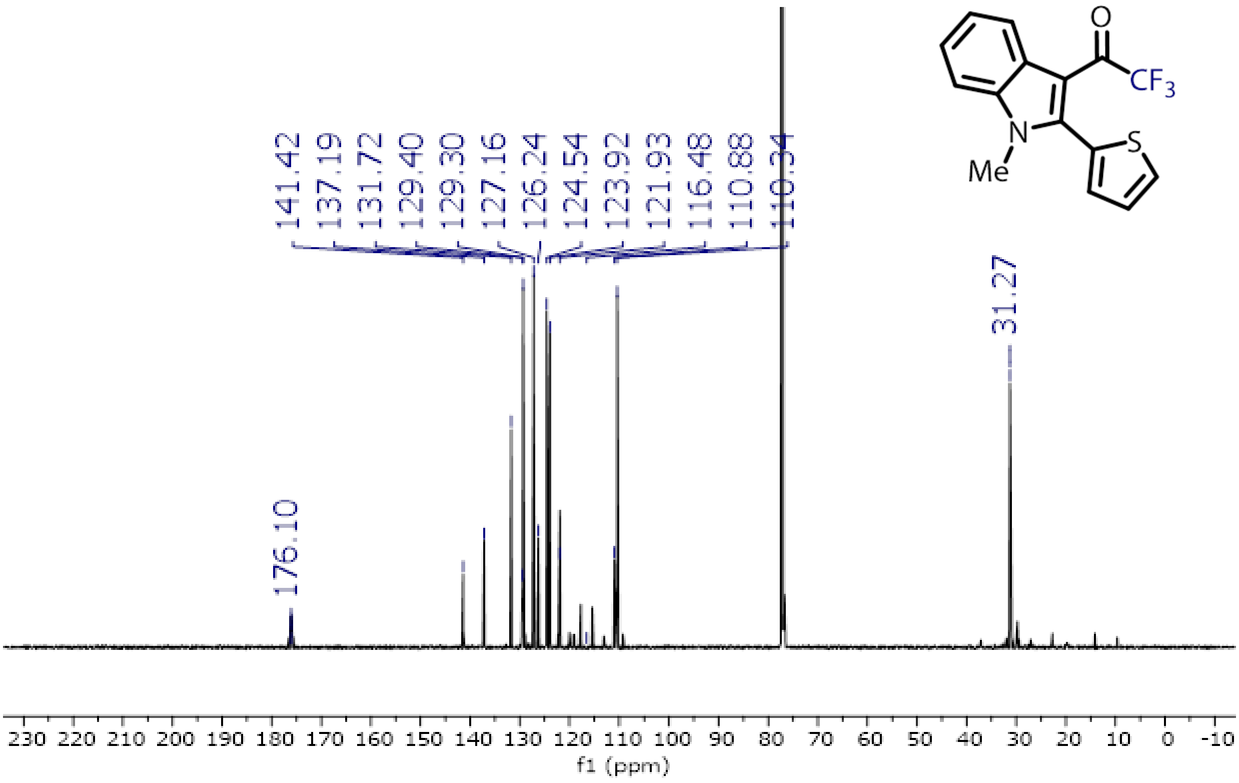

^{19}F NMR (376 MHz, $CDCl_3$):

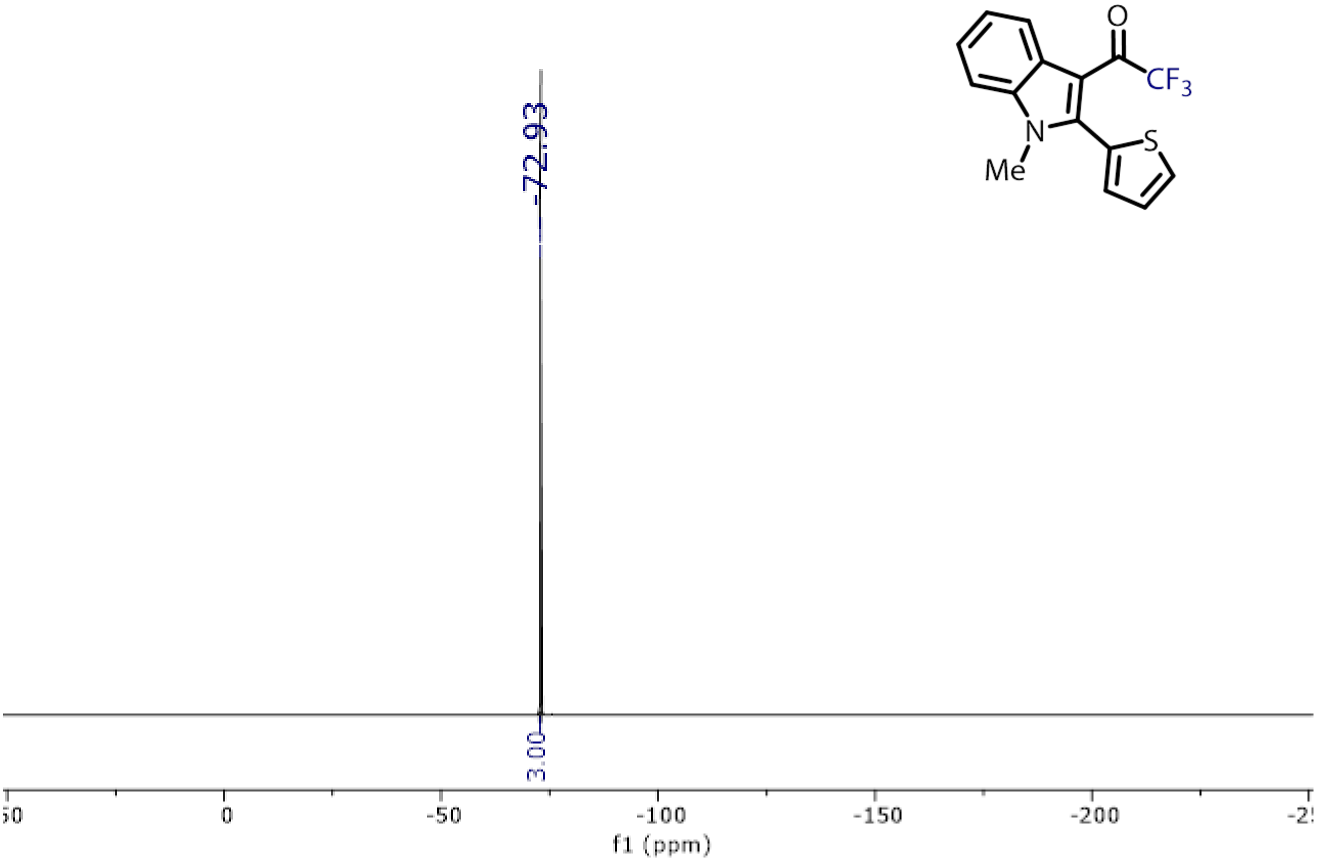

(E)-2,2,2-trifluoro-N-phenethyl-1-phenylethan-1-imine, ***1au***

^{1}H NMR (400 MHz, $CDCl_3$):

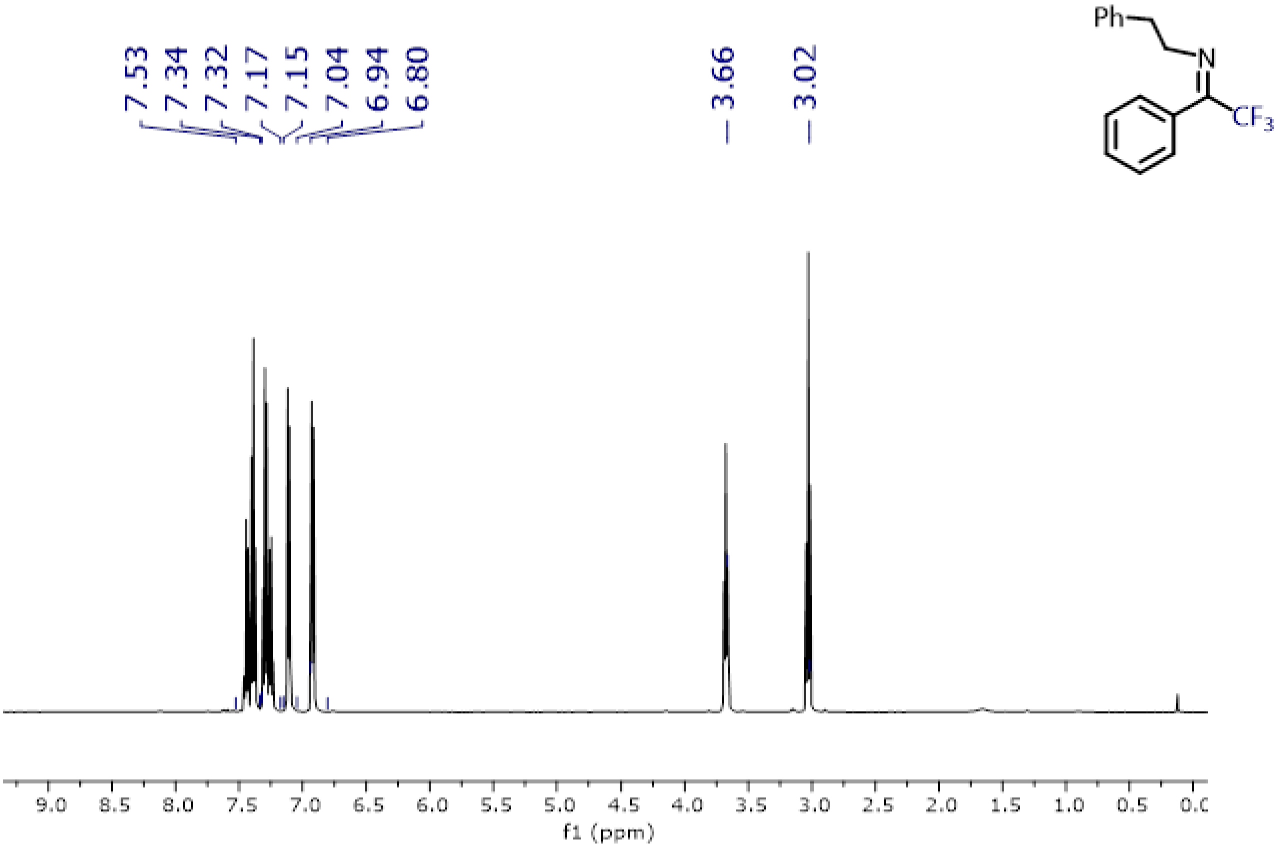

^{13}C NMR (131 MHz, $CDCl_3$):

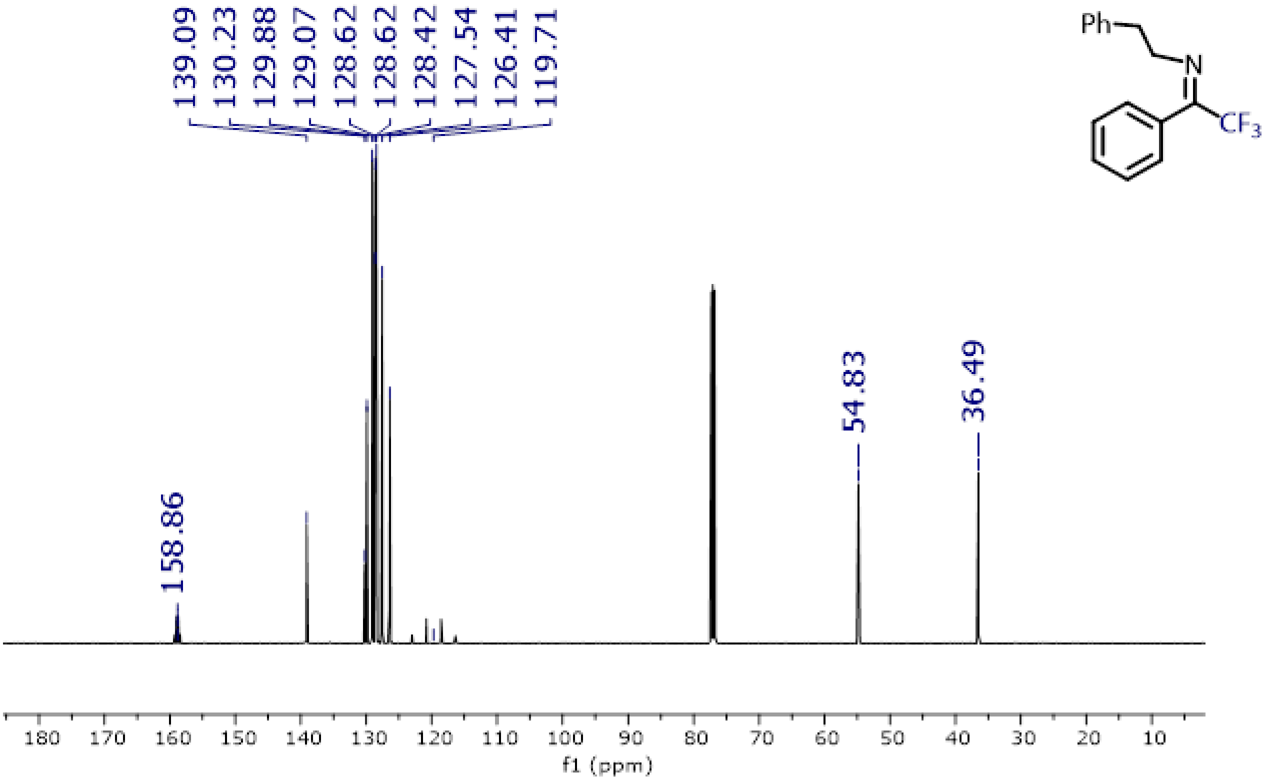

^{19}F NMR (376 MHz, $CDCl_3$):

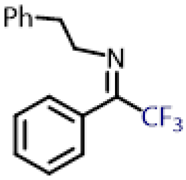

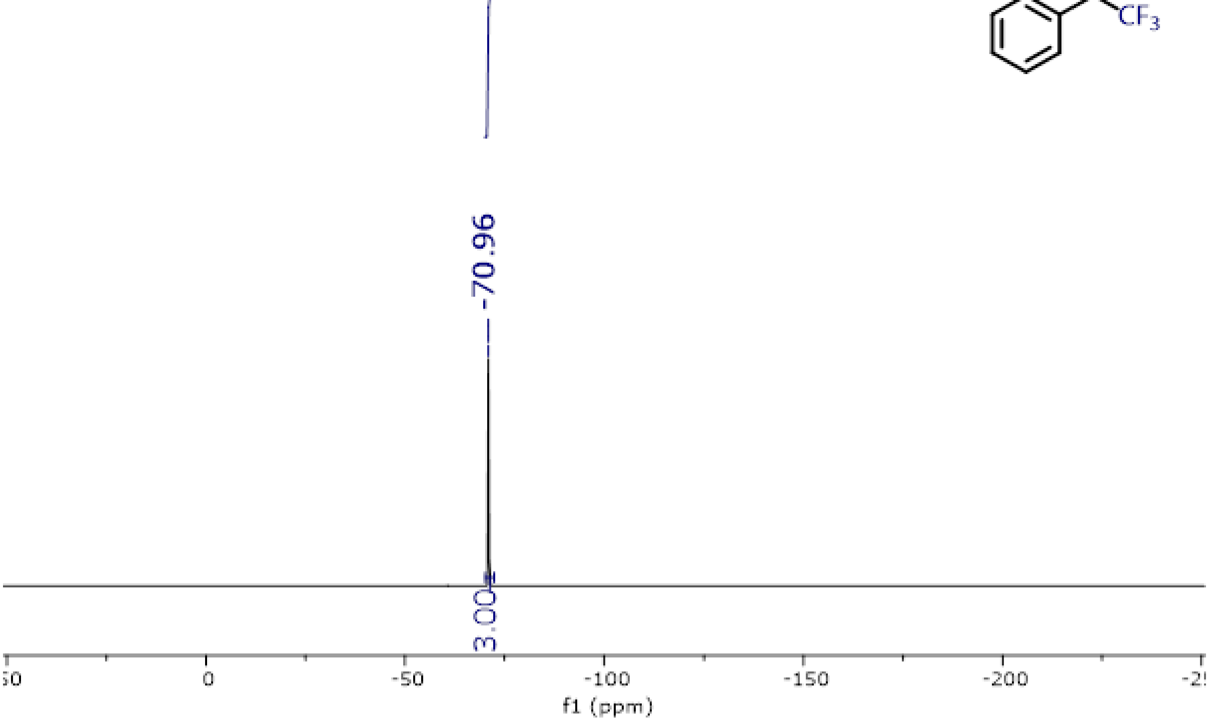

(E)-2,2,2-trifluoro-N-(4-methoxybenzyl)-1-phenylethan-1-imine, ***1av***

^{1}H NMR (400 MHz, $CDCl_3$):

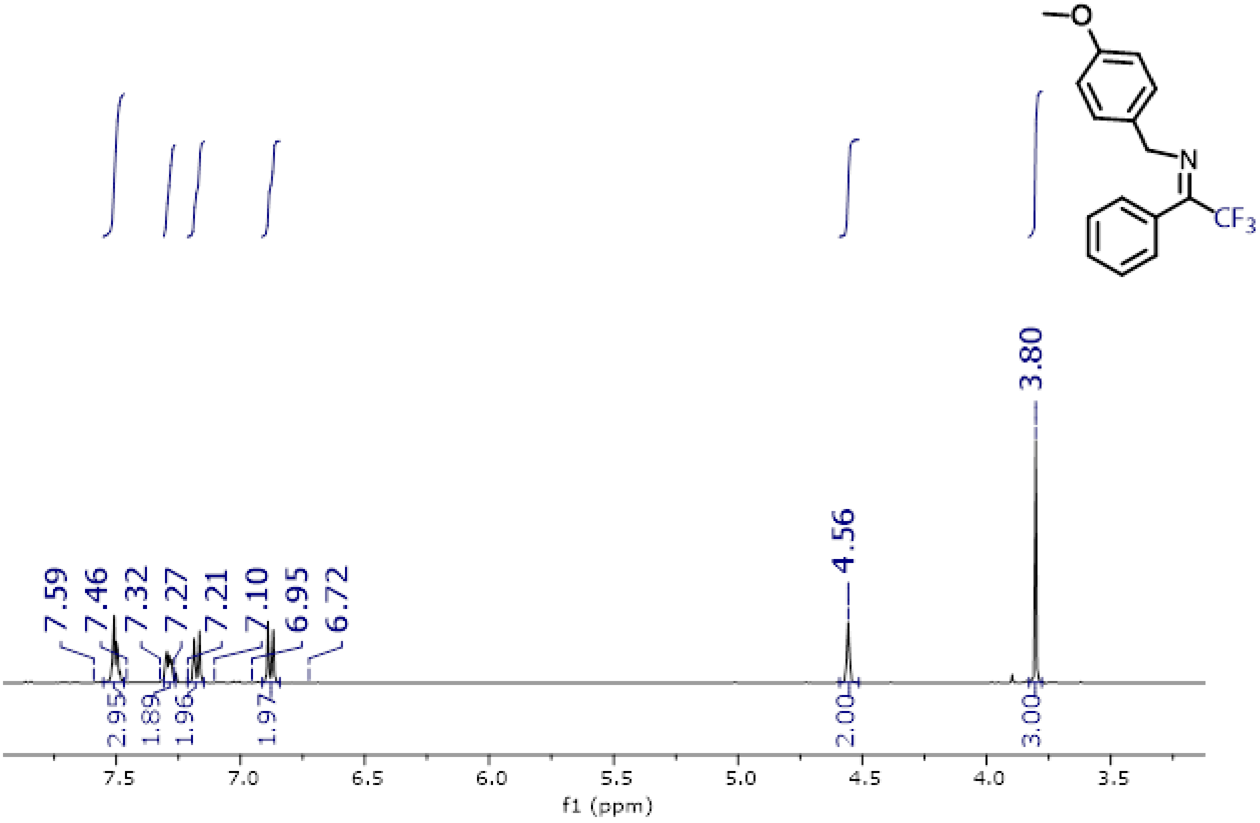

^{13}C NMR (131 MHz, $CDCl_3$):

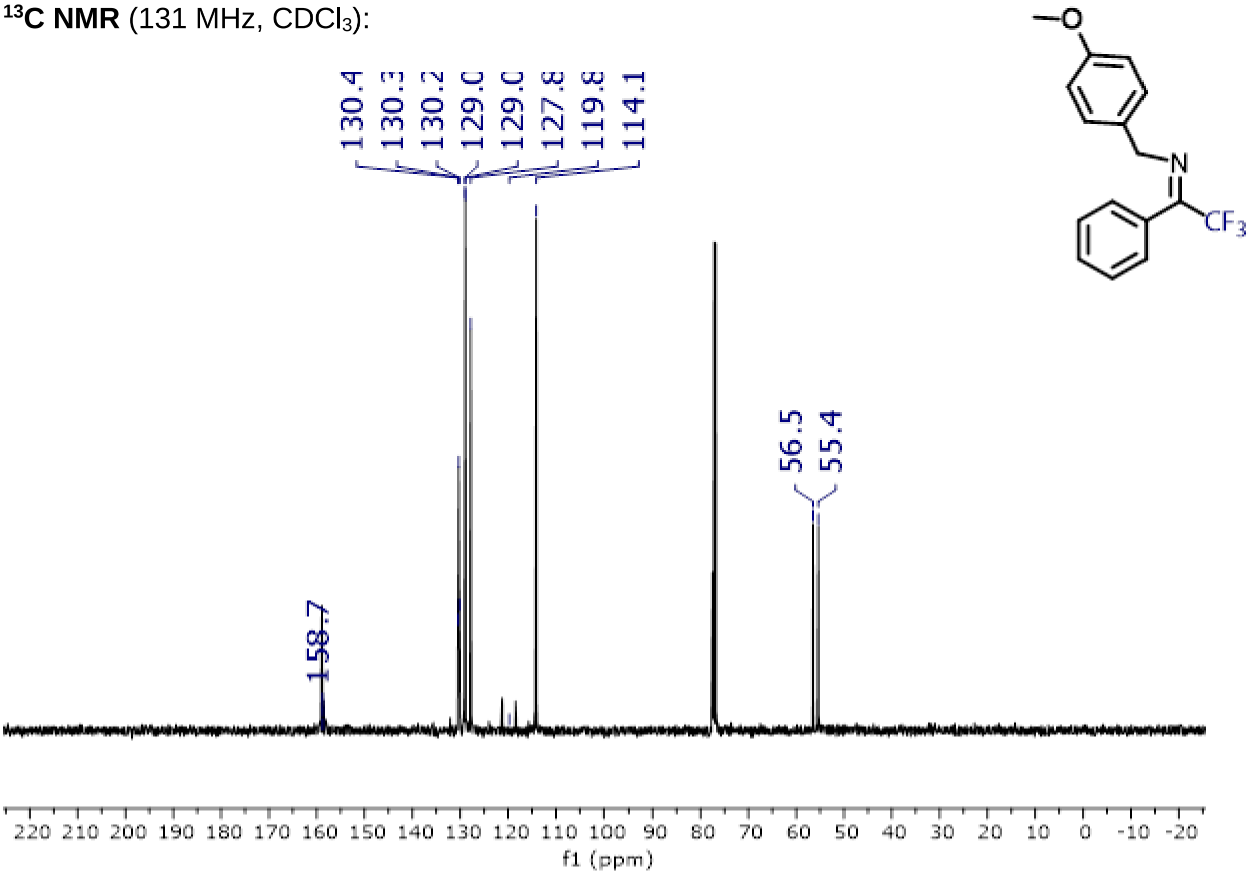

^{19}F NMR (376 MHz, $CDCl_3$):

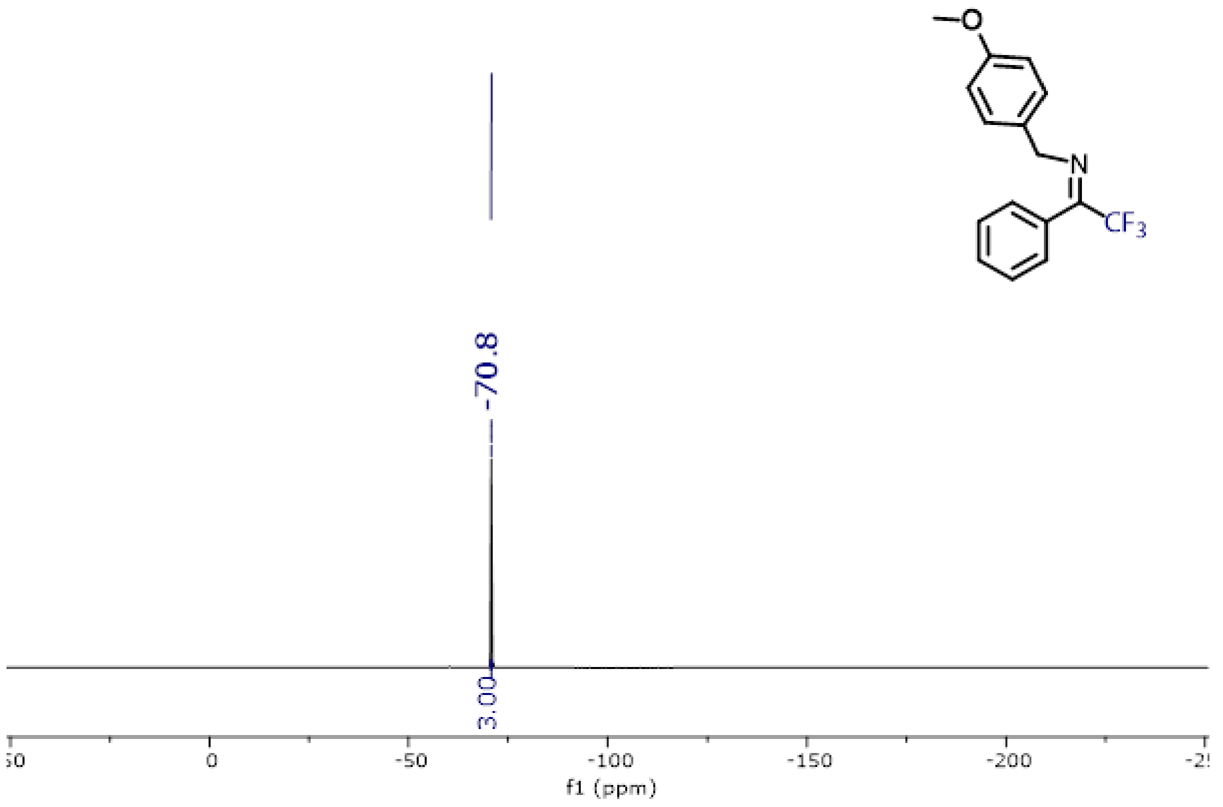

NMR Spectra of Novel Products

2,2-difluoro-1-(4-pentylphenyl)ethan-1-one, **3j**

^{1}H NMR (400 MHz, $CDCl_3$):

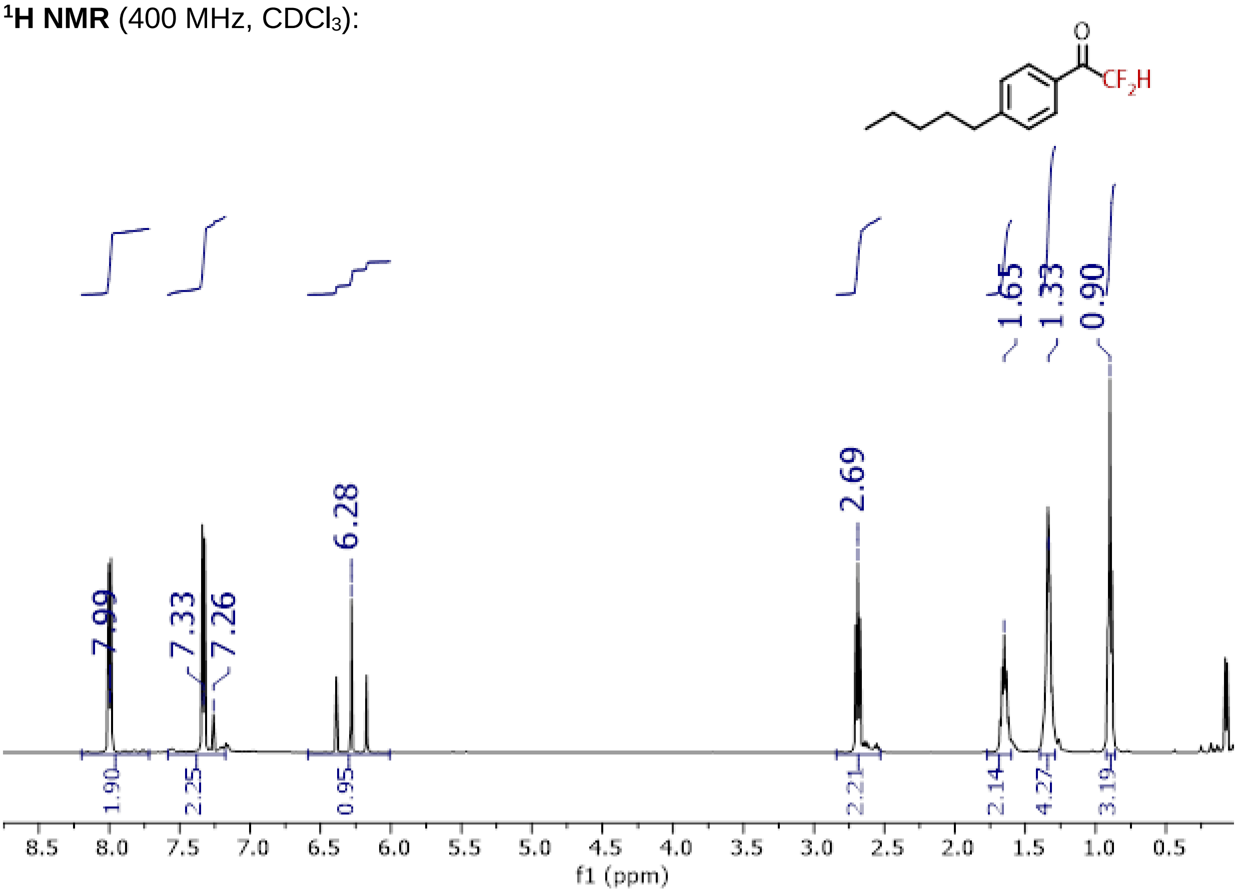

^{13}C NMR (131 MHz, $CDCl_3$):

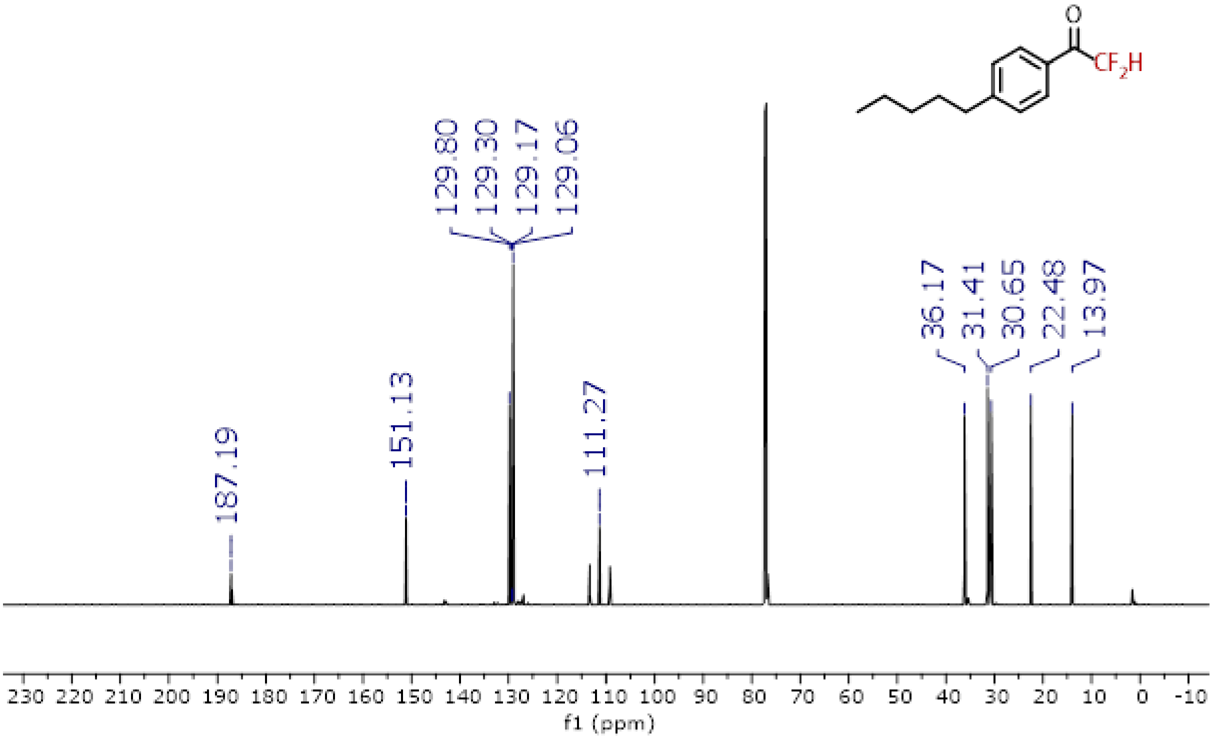

^{19}F NMR (376 MHz, $CDCl_3$):

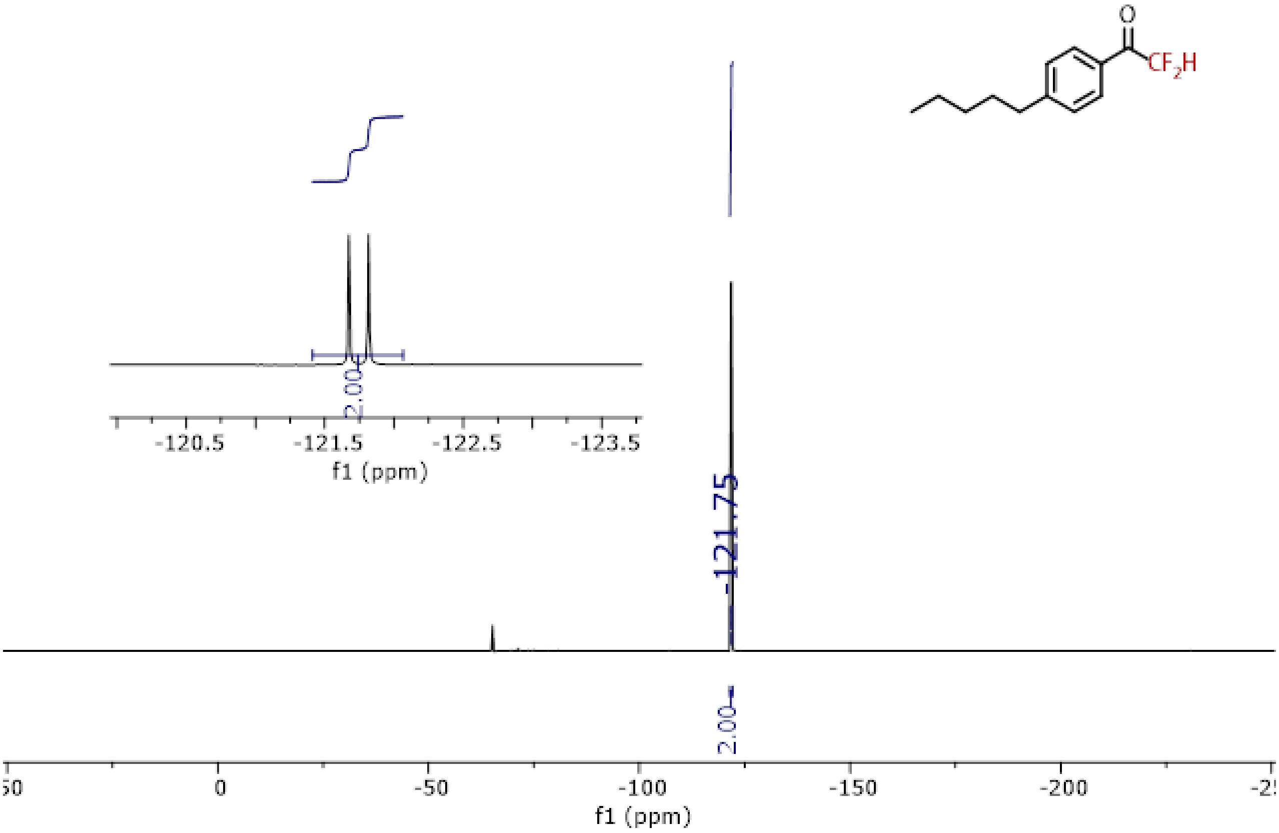

2,2-difluoro-1-(2-methyl-4-phenoxyphenyl)ethan-1-one, **3k**

^{1}H NMR (400 MHz, $CDCl_3$):

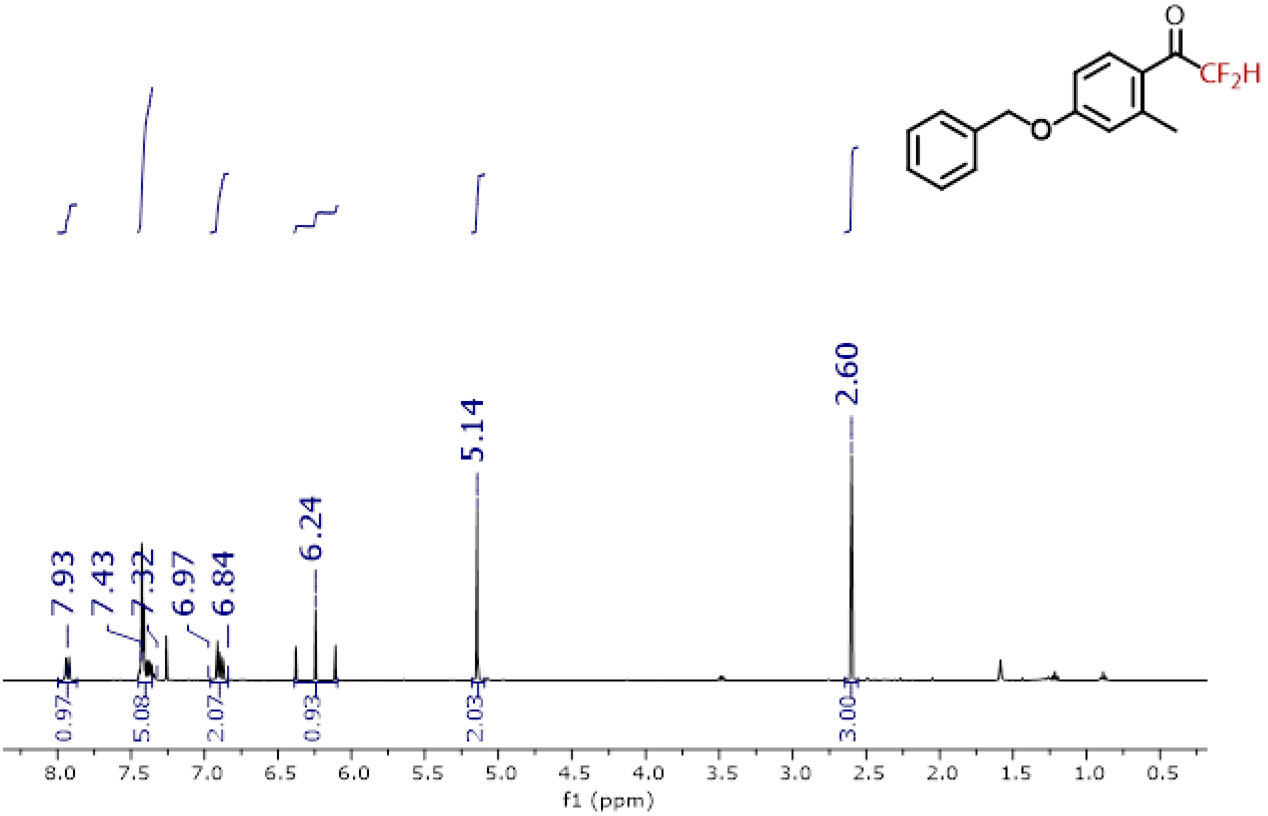

^{13}C NMR (131 MHz, $CDCl_3$):

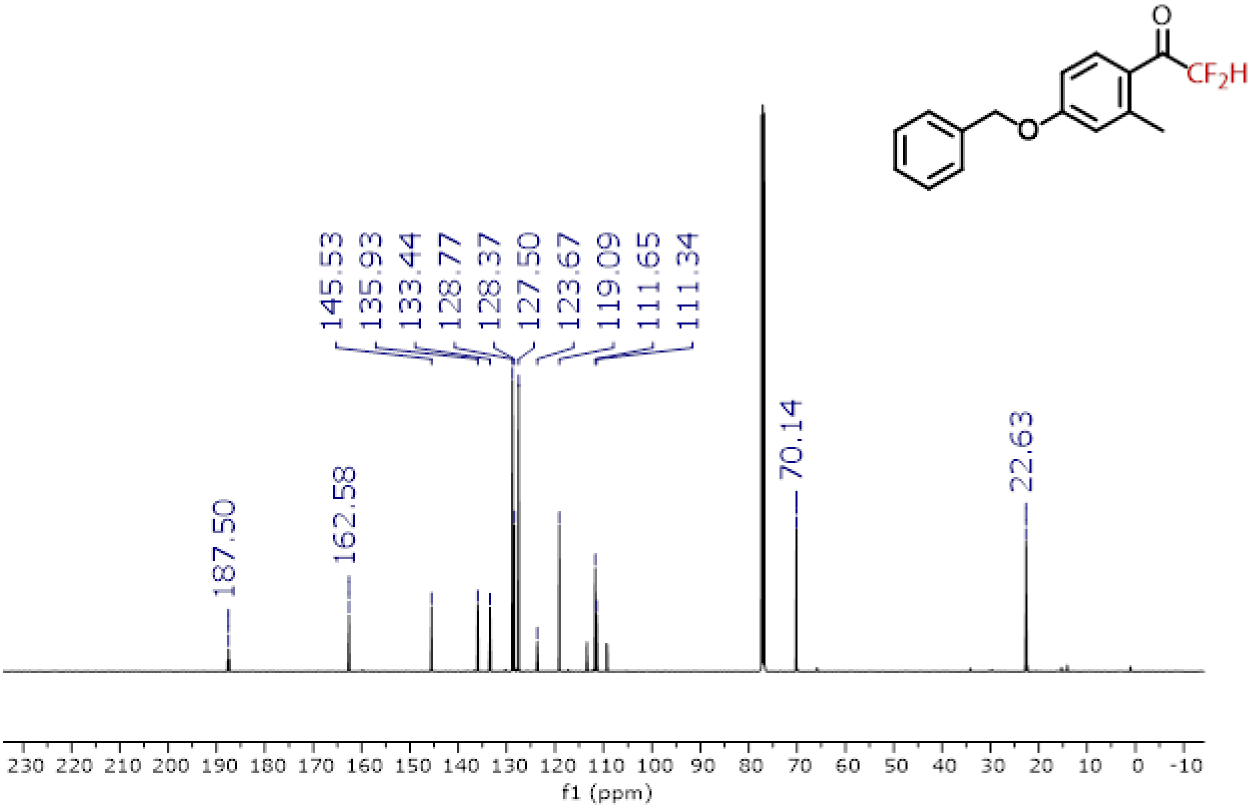

^{19}F NMR (376 MHz, $CDCl_3$):

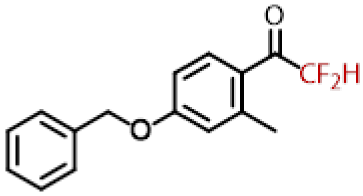

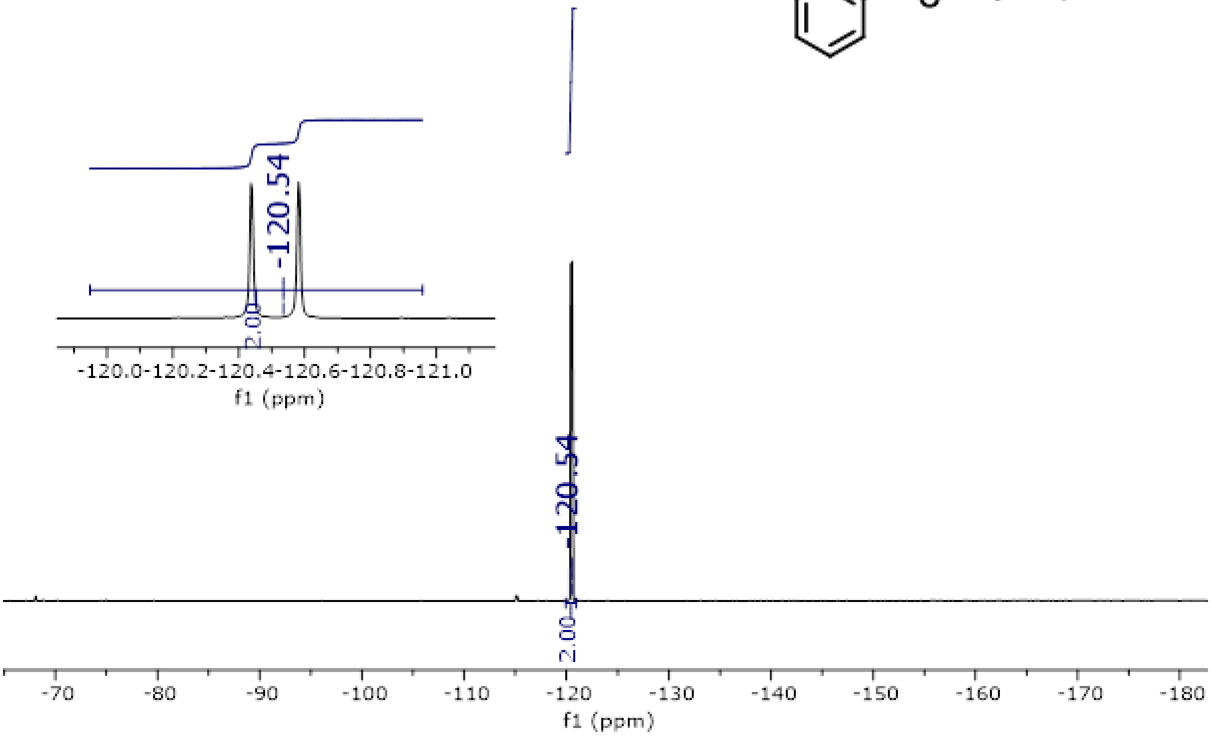

2,2-Difluoro-1-(4-(pyridin-2-yl)phenyl)ethan-1-one, **3l**

^{1}**H NMR** (400 MHz, $CDCl_3$):

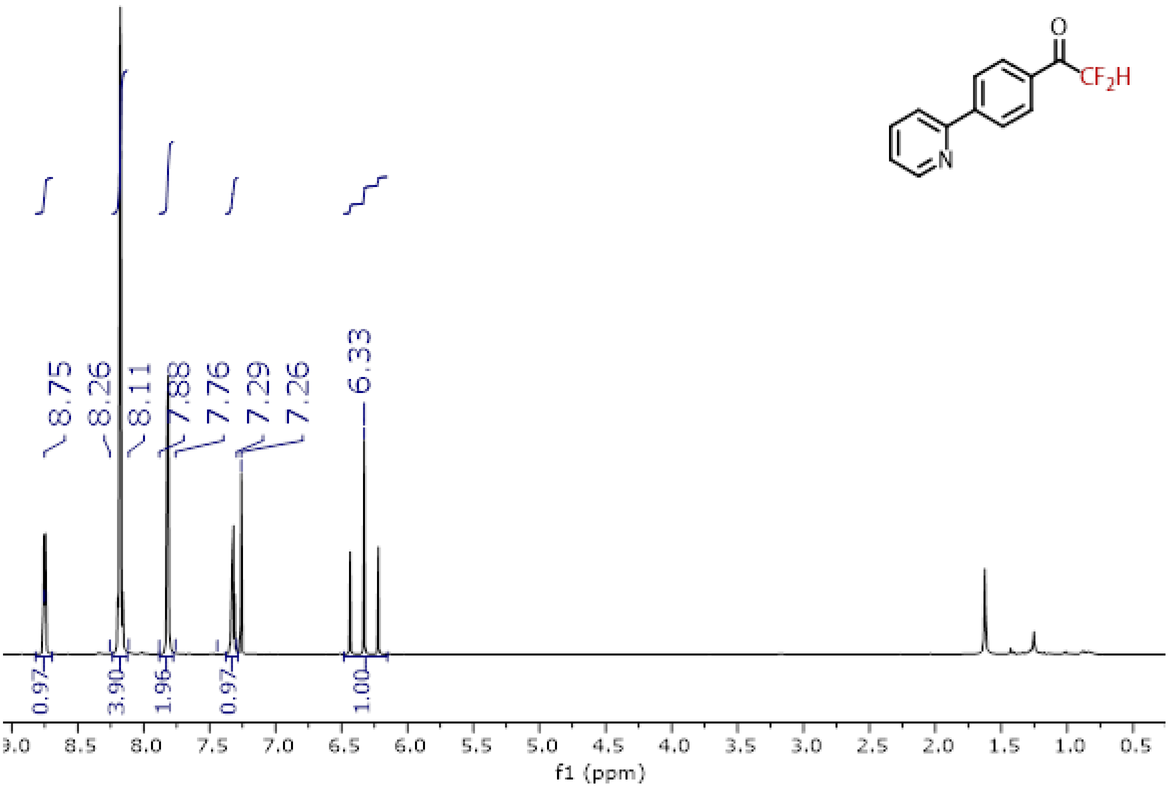

^{13}**C NMR** (131 MHz, $CDCl_3$):

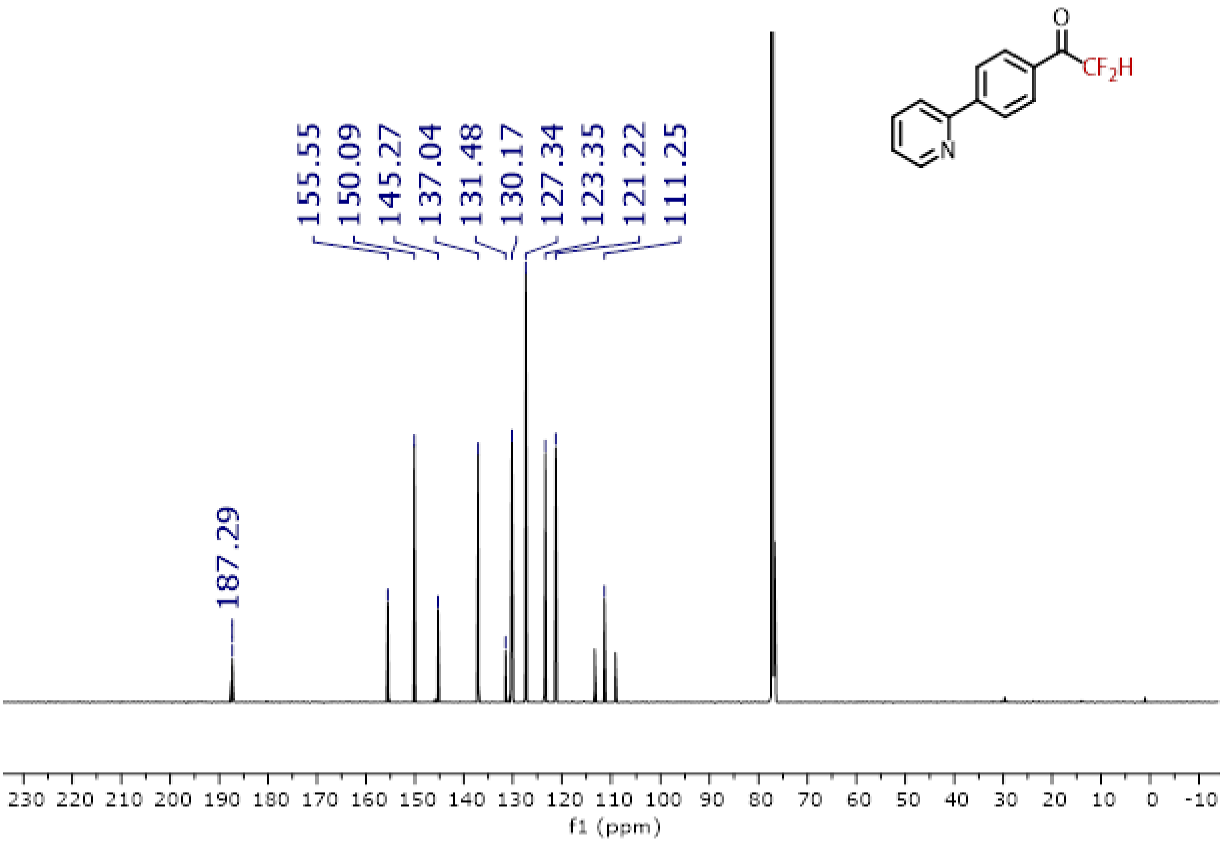

^{19}F NMR (376 MHz, $CDCl_3$):

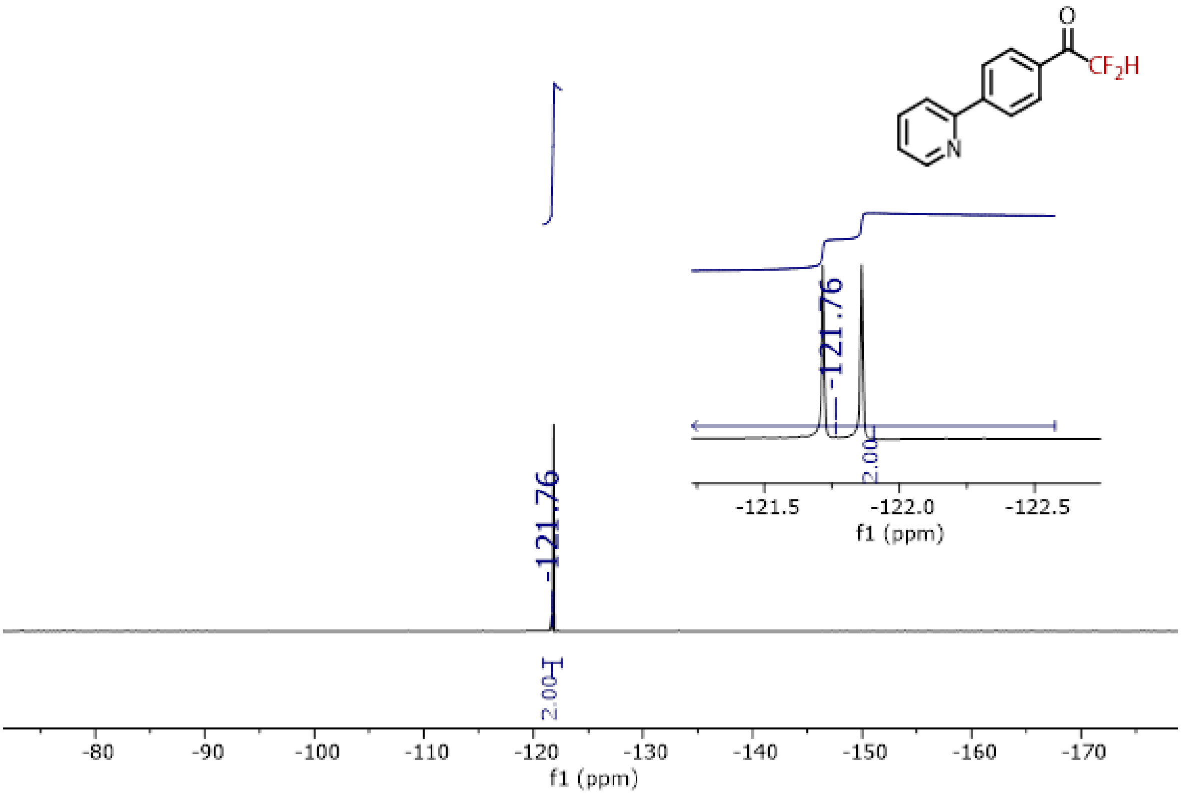

1-(4-(benzo[b]thiophen-3-yl)phenyl)-2,2-difluoroethan-1-one, **3n**

^{1}H NMR (400 MHz, $CDCl_3$):

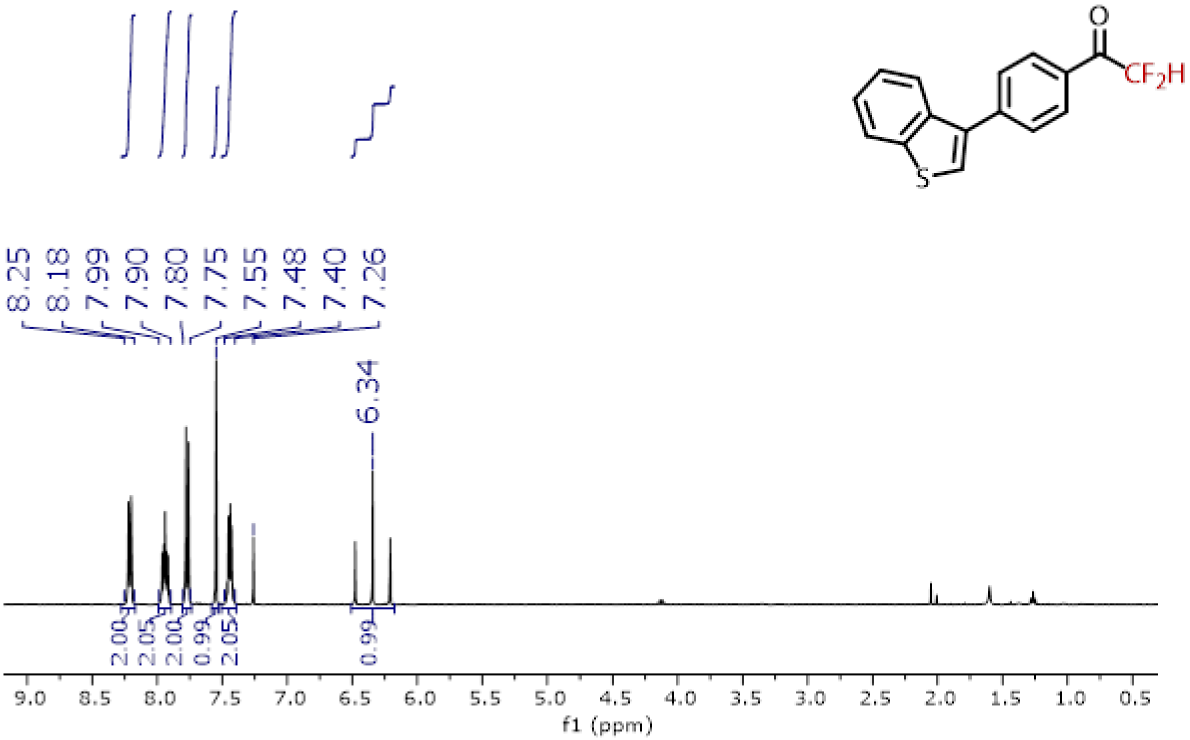

^{13}C NMR (131 MHz, $CDCl_3$):

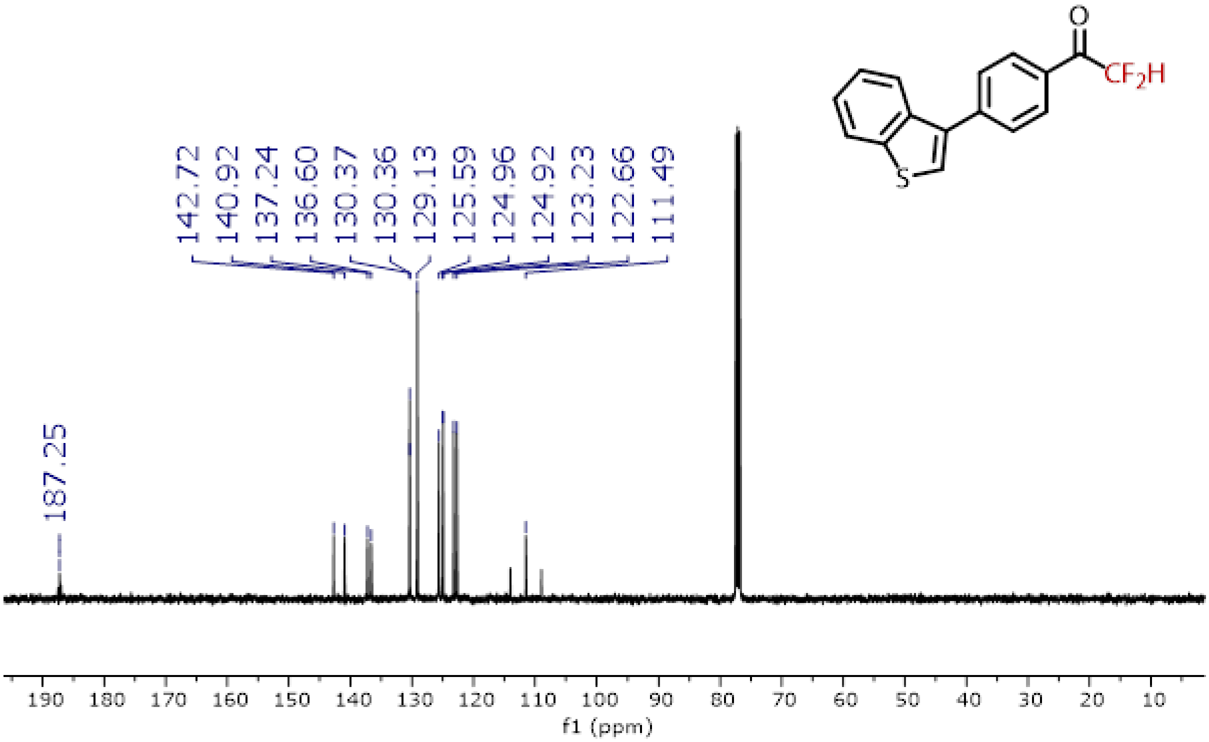

^{19}F NMR (376 MHz, $CDCl_3$):

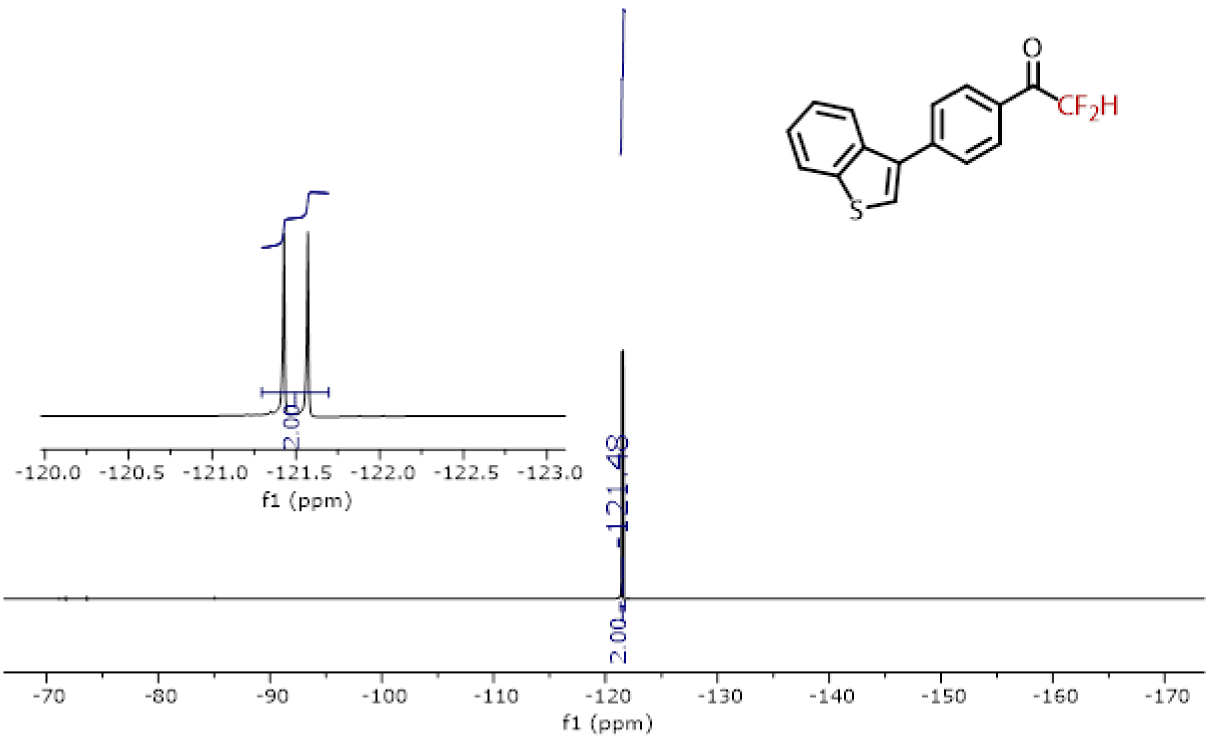

1-(3'-amino-[1,1'-biphenyl]-4-yl)-2,2-difluoroethan-1-one **3o**

^{1}H NMR (400 MHz, $CDCl_3$):

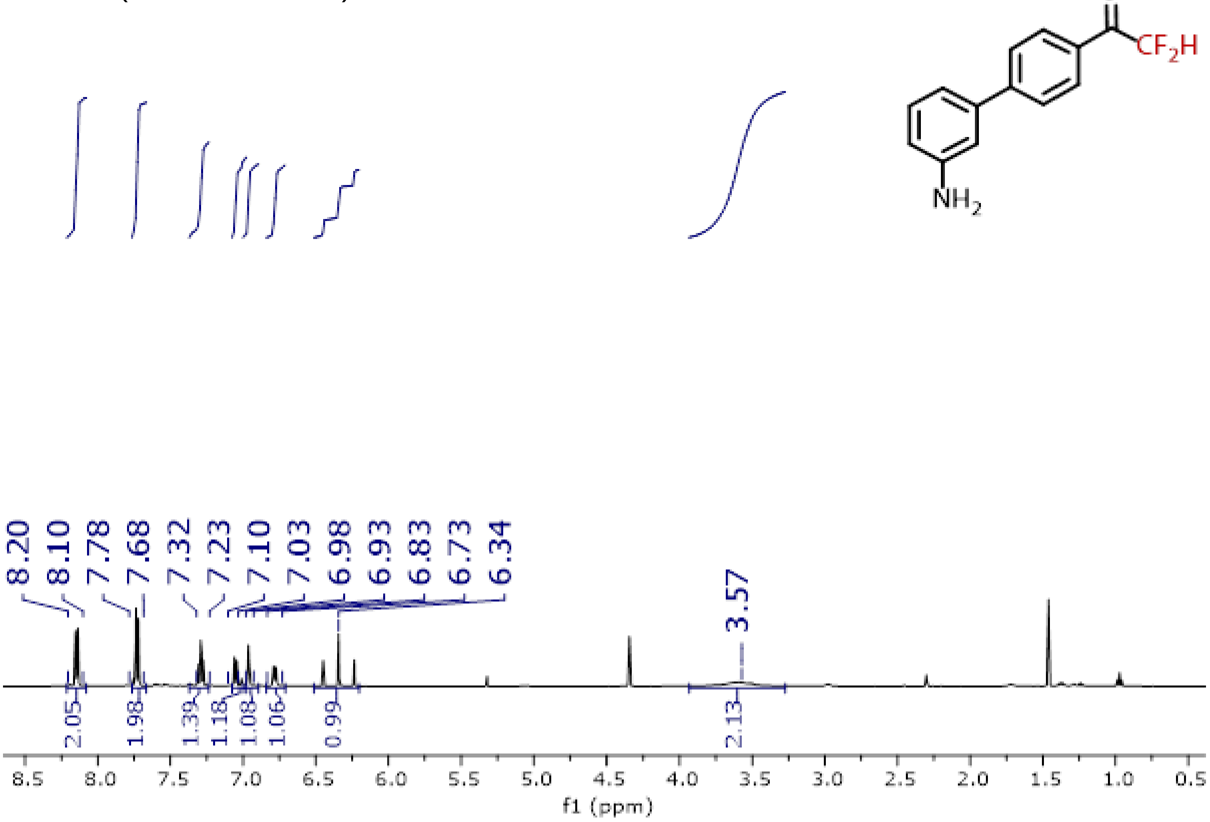

^{13}C NMR (131 MHz, $CDCl_3$):

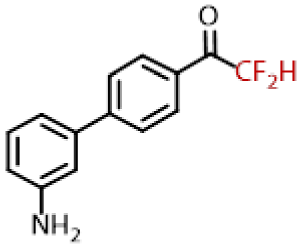

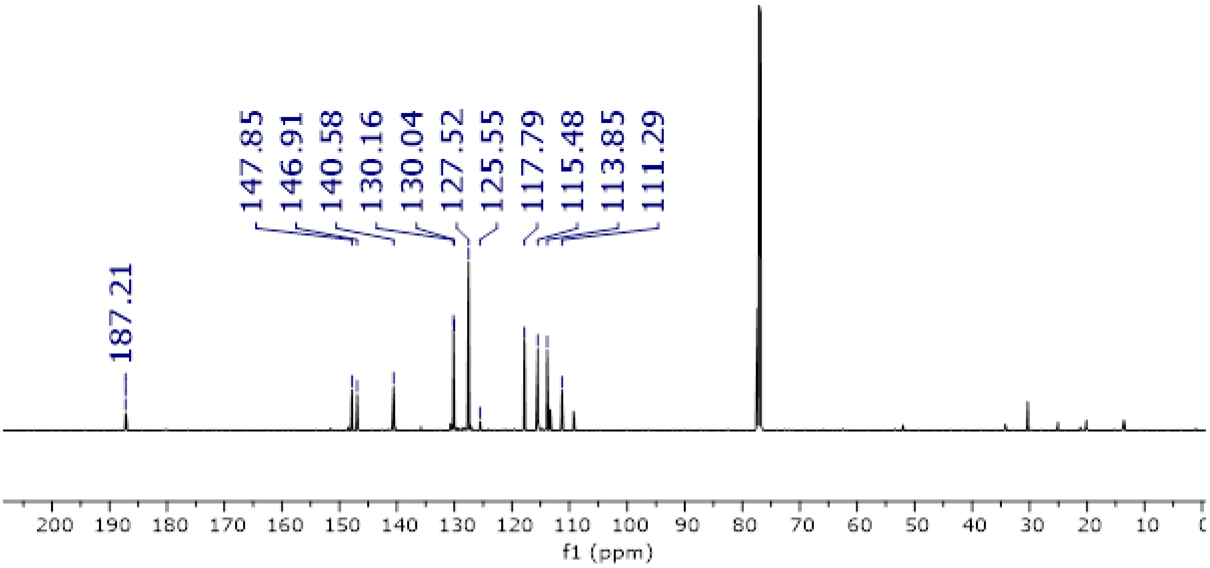

^{19}F NMR (376 MHz, $CDCl_3$):

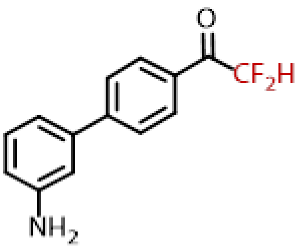

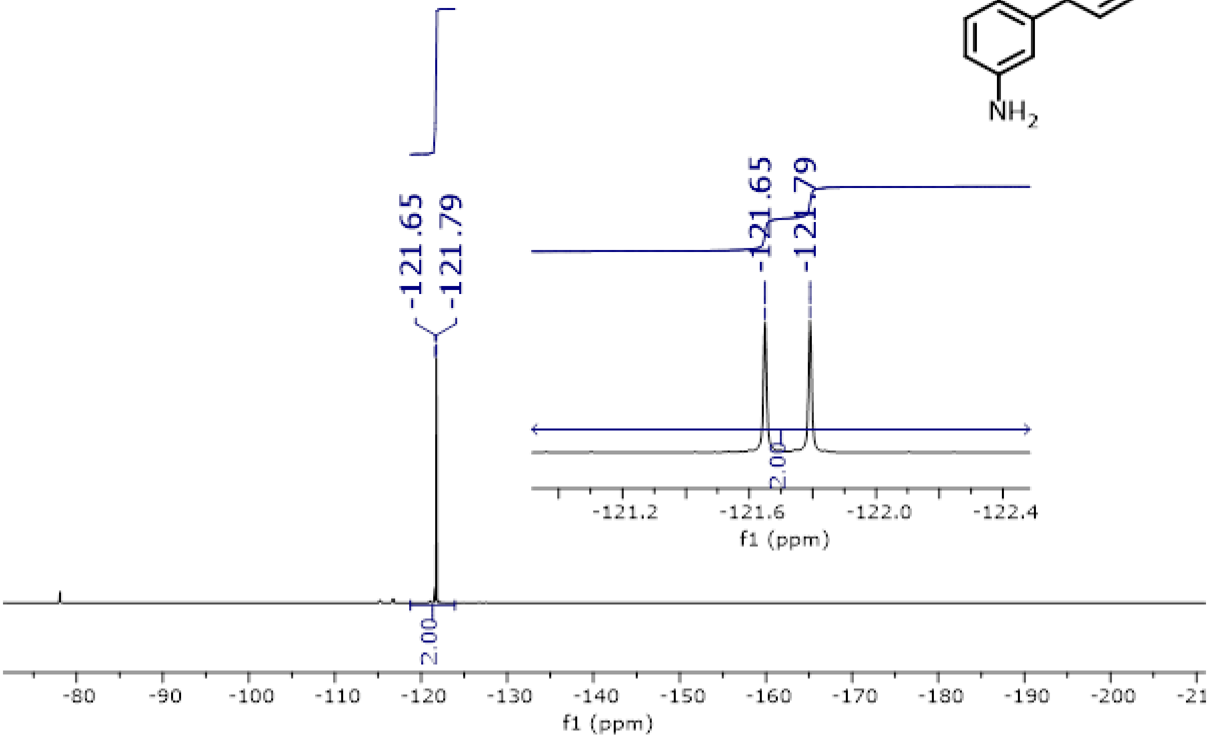

2,2-difluoro-1-(4-(2-methoxypyrimidin-5-yl)phenyl)ethan-1-one, **3p**

^{1}H NMR (400 MHz, $CDCl_3$):

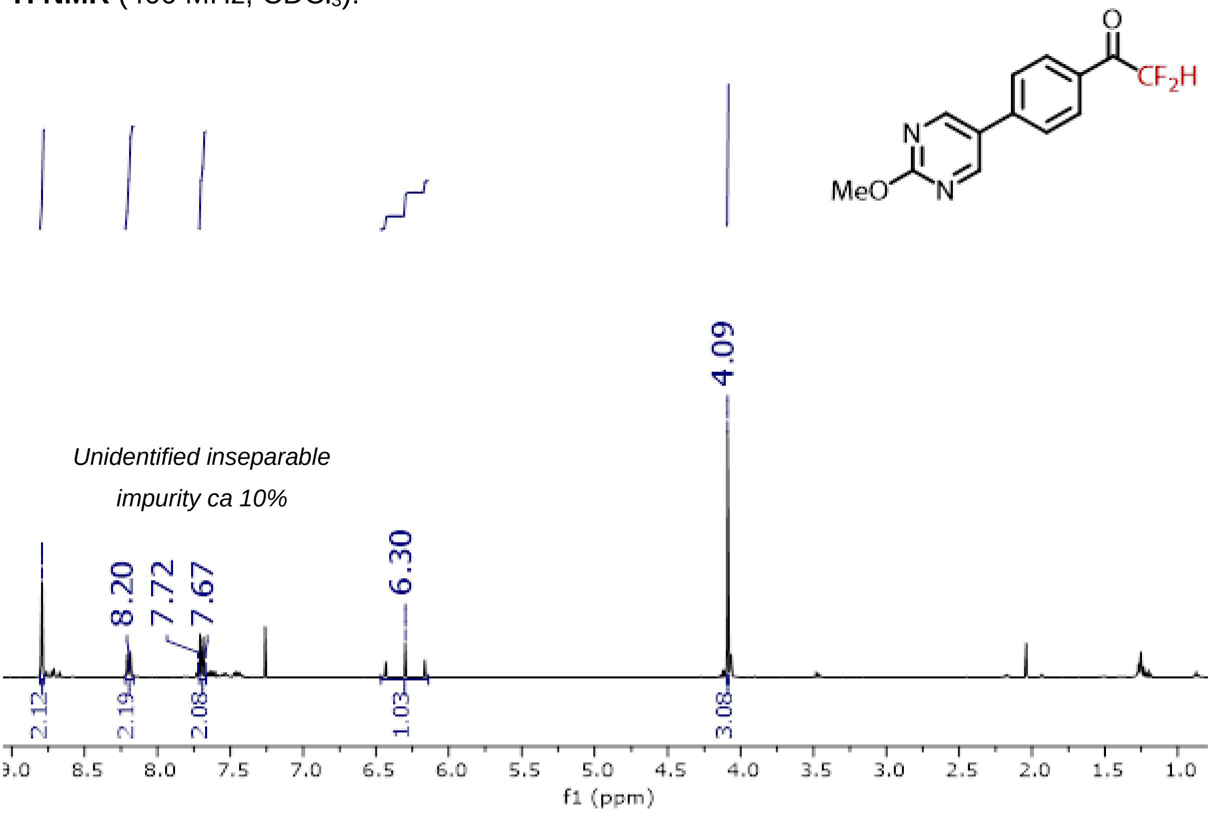

^{13}C NMR (131 MHz, $CDCl_3$):

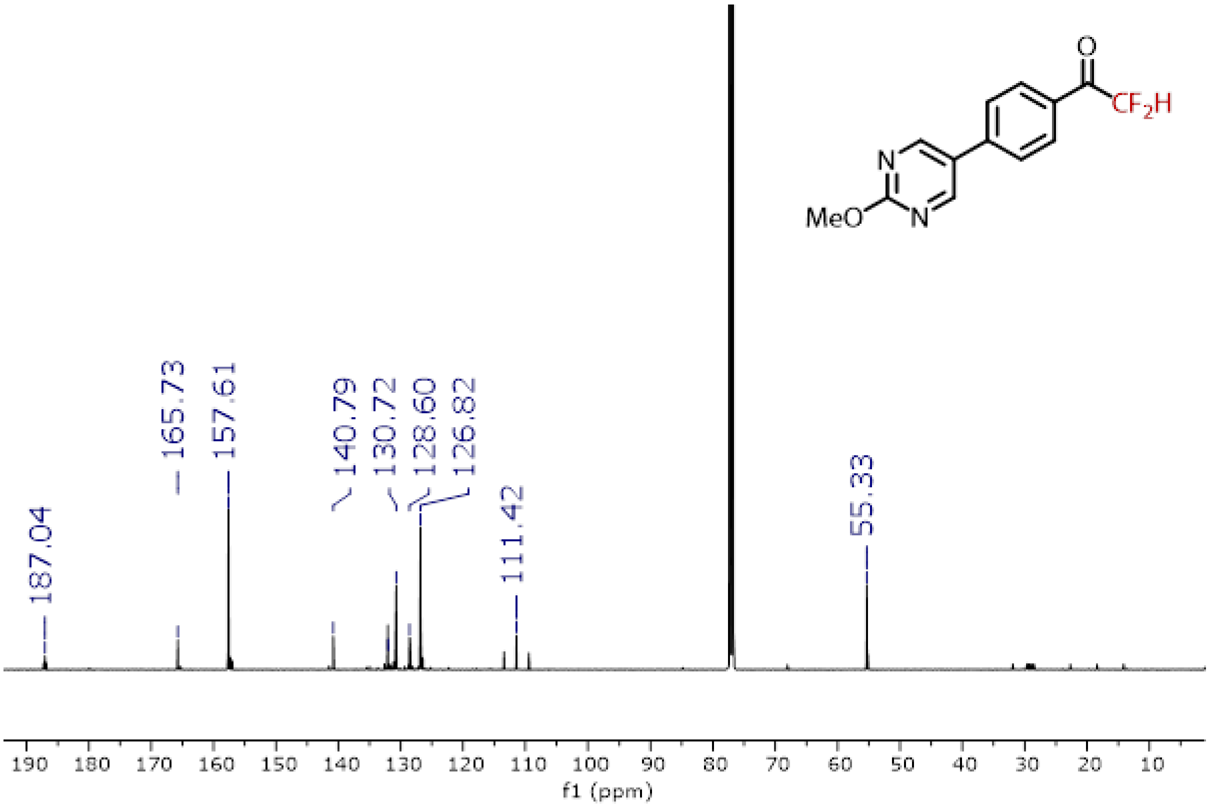

^{19}F NMR (376 MHz, $CDCl_3$):

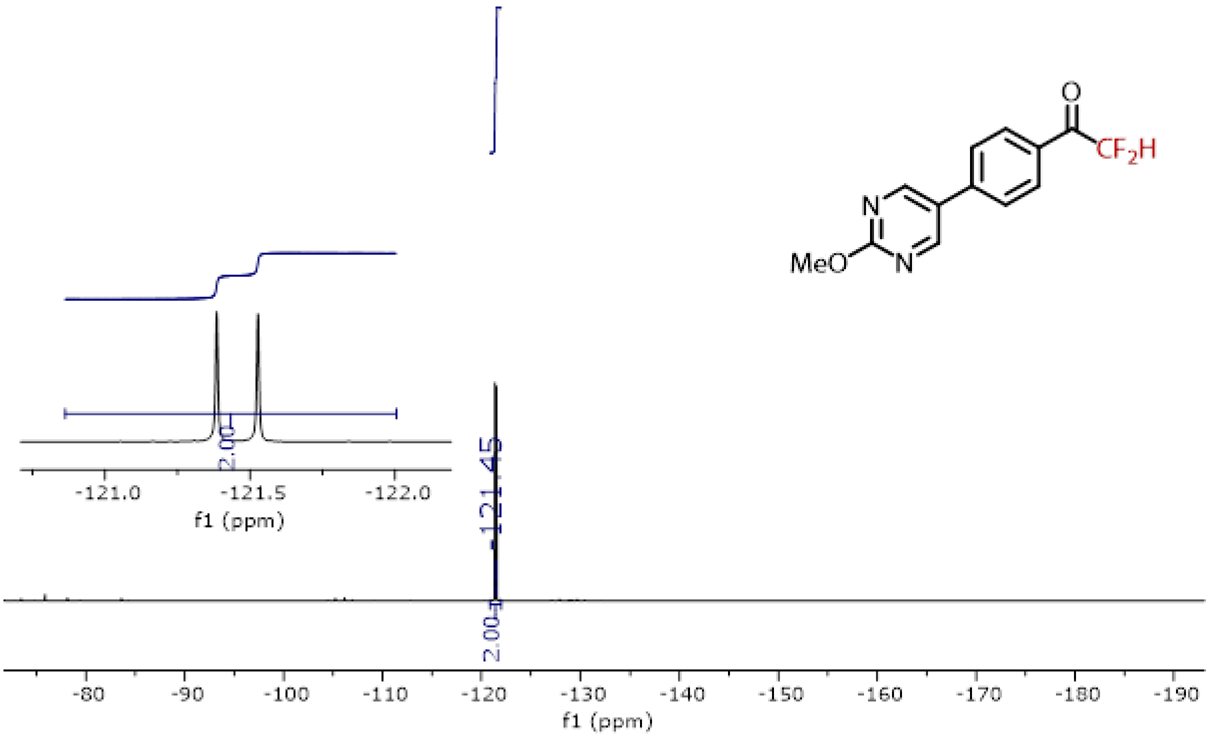

1-(4-(2,6-diphenylpyrimidin-4-yl)phenyl)-2,2-difluoroethan-1-one, **3q**

[1]**H NMR** (400 MHz, $CDCl_3$):

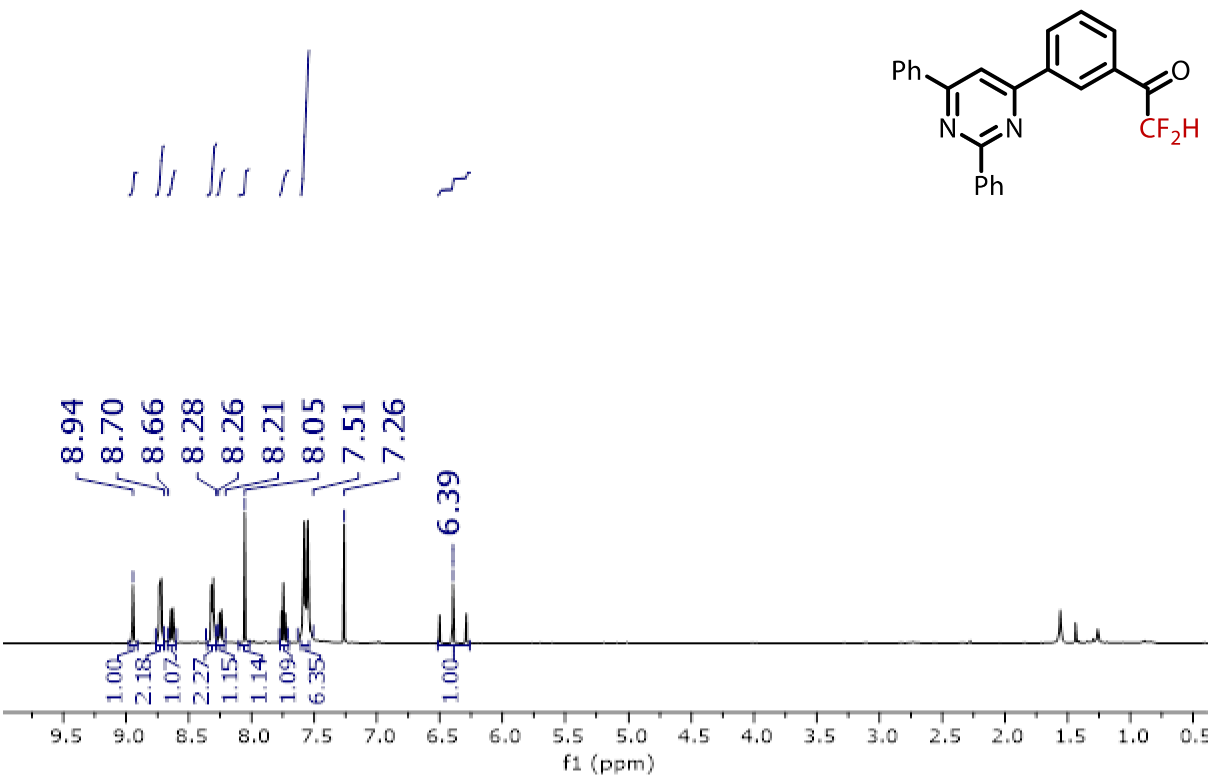

[13]**C NMR** (131 MHz, $CDCl_3$):

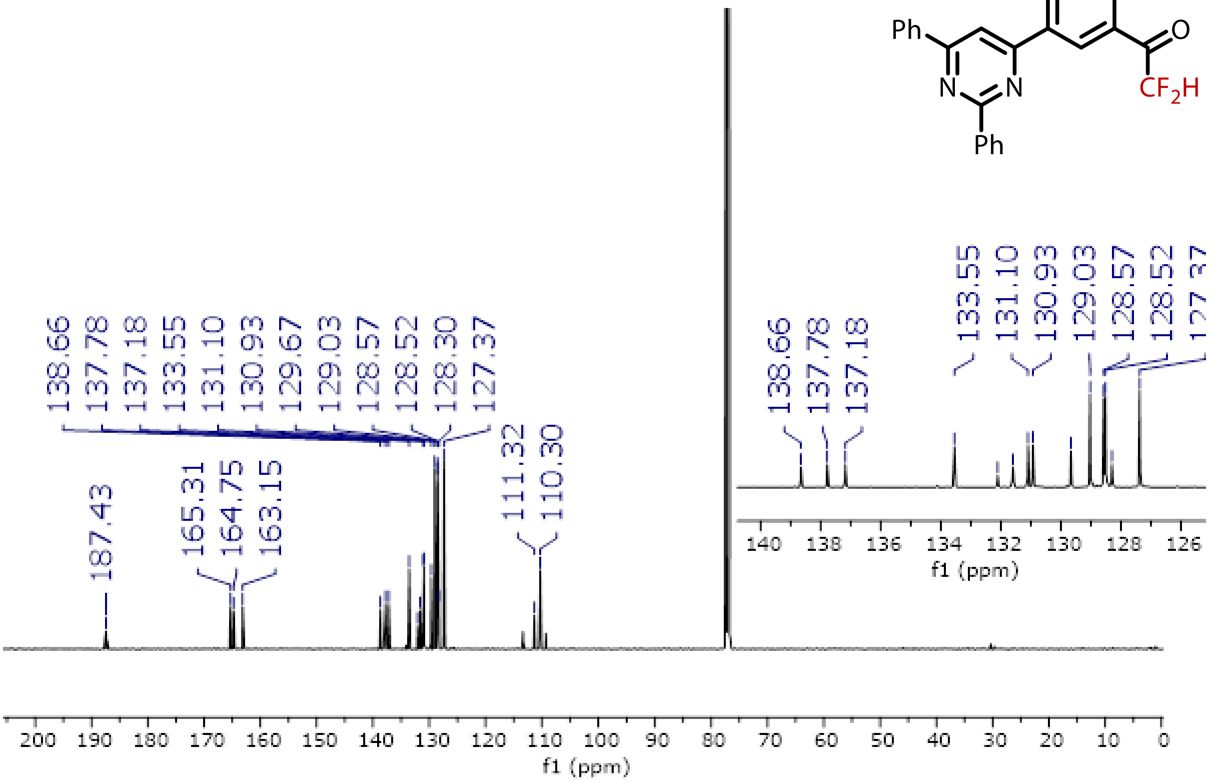

^{19}F NMR (376 MHz, $CDCl_3$):

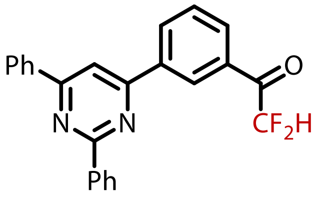

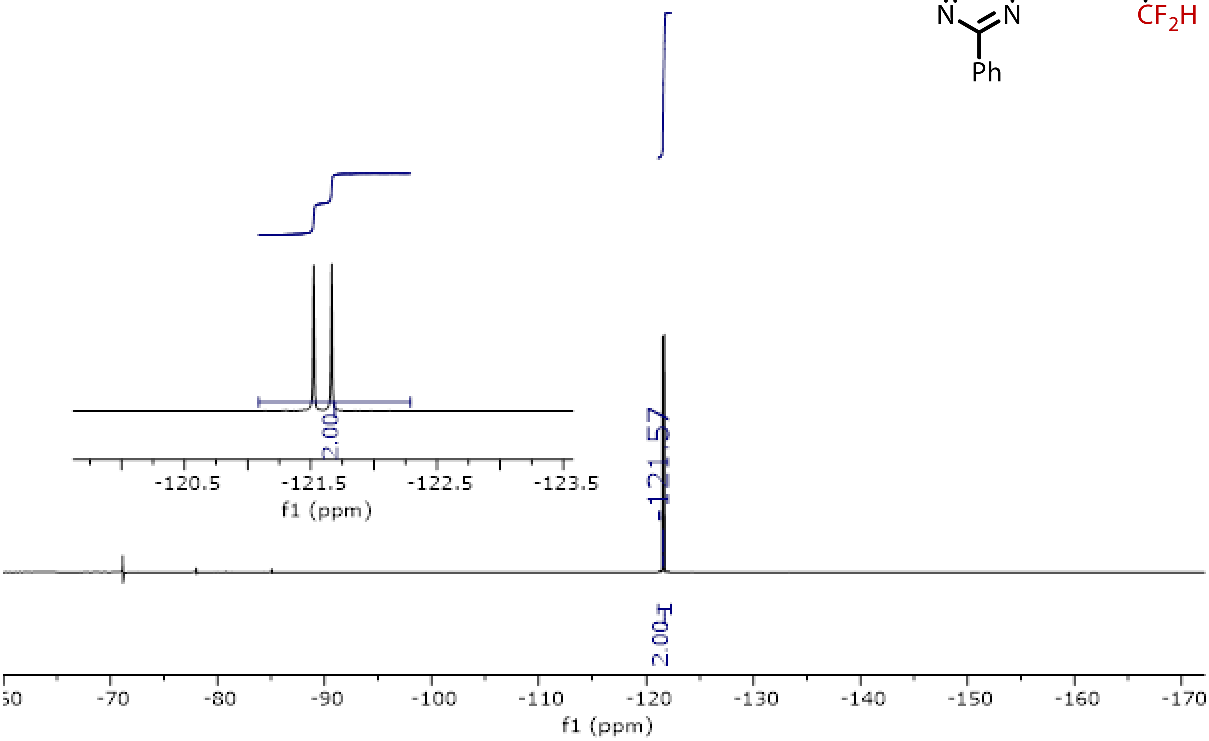

(E)-1-(4-(4-(dibutylamino)styryl)phenyl)-2,2-difluoroethan-1-one, **3r**

^{1}H NMR (400 MHz, $CDCl_3$):

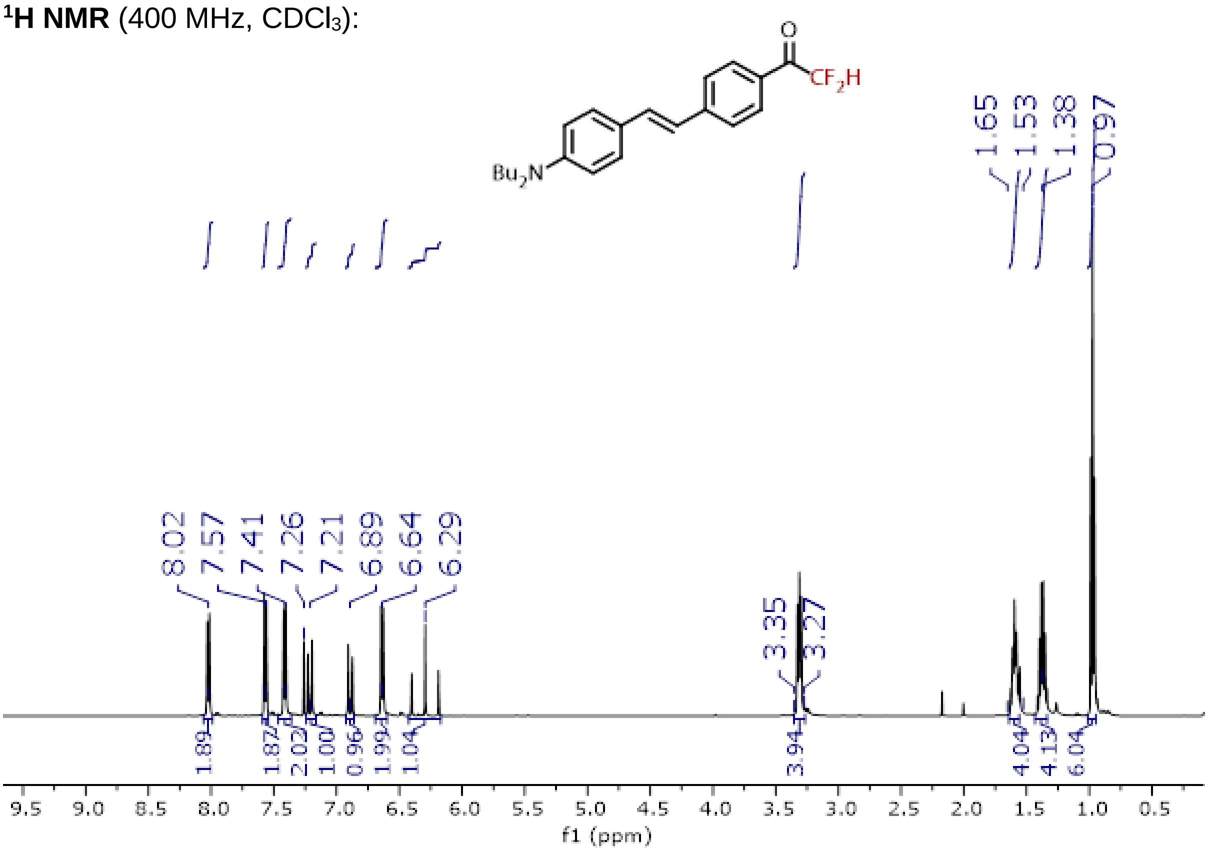

^{13}C NMR (131 MHz, $CDCl_3$):

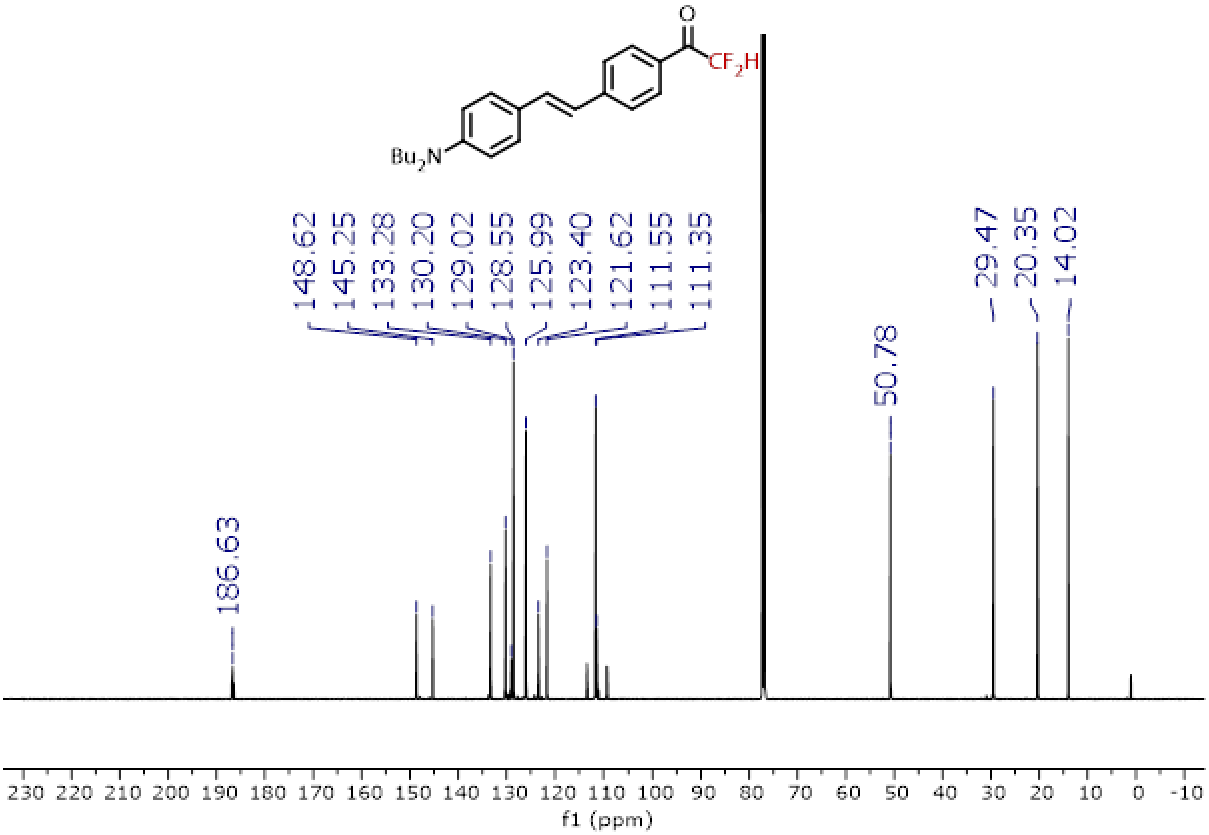

^{19}F NMR (376 MHz, $CDCl_3$):

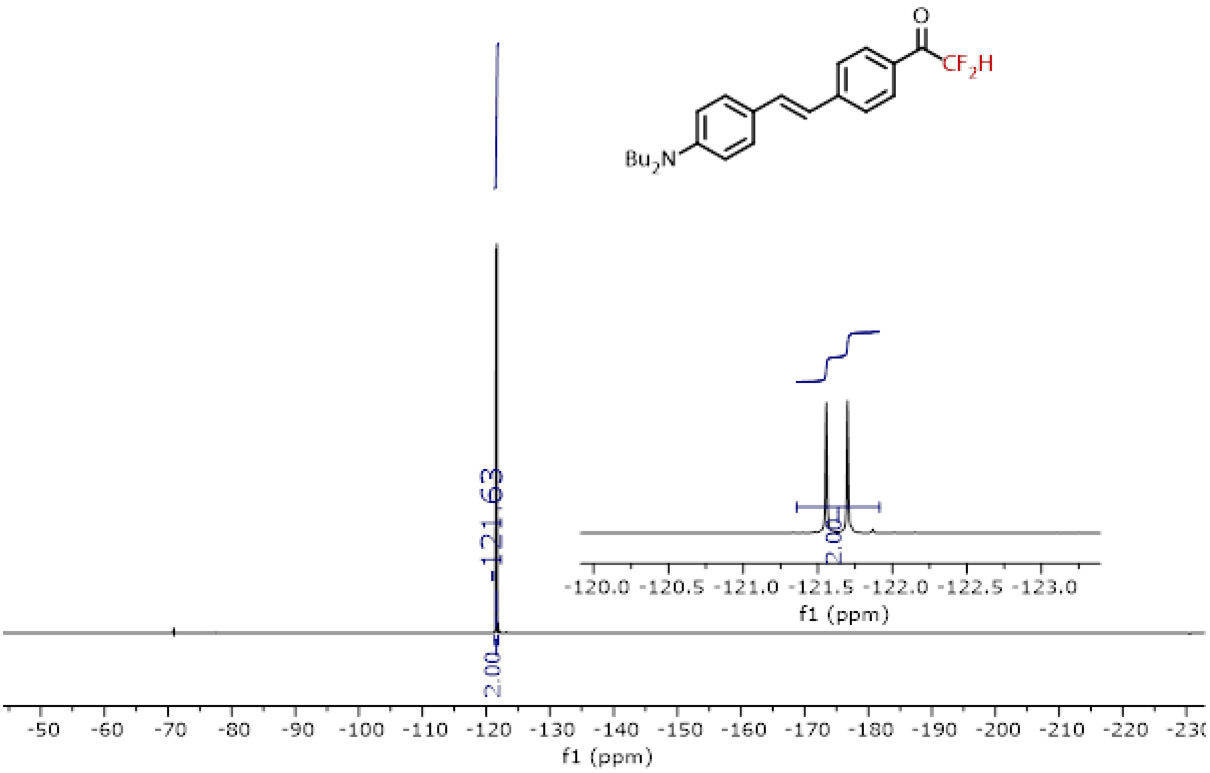

2,2-Difluoro-1-(4-(pyridin-2-yl)phenyl)ethan-1-one, **3s**

^{1}H NMR (400 MHz, $CDCl_3$):

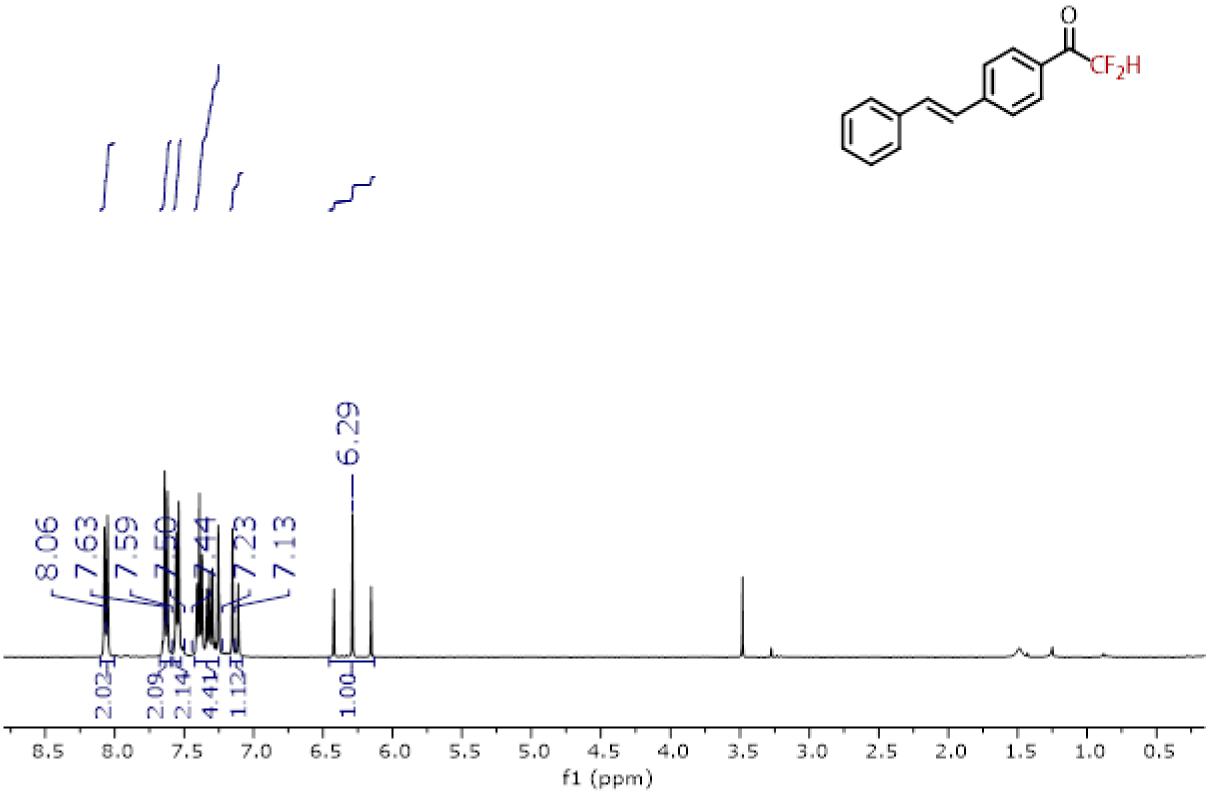

^{13}C NMR (131 MHz, $CDCl_3$):

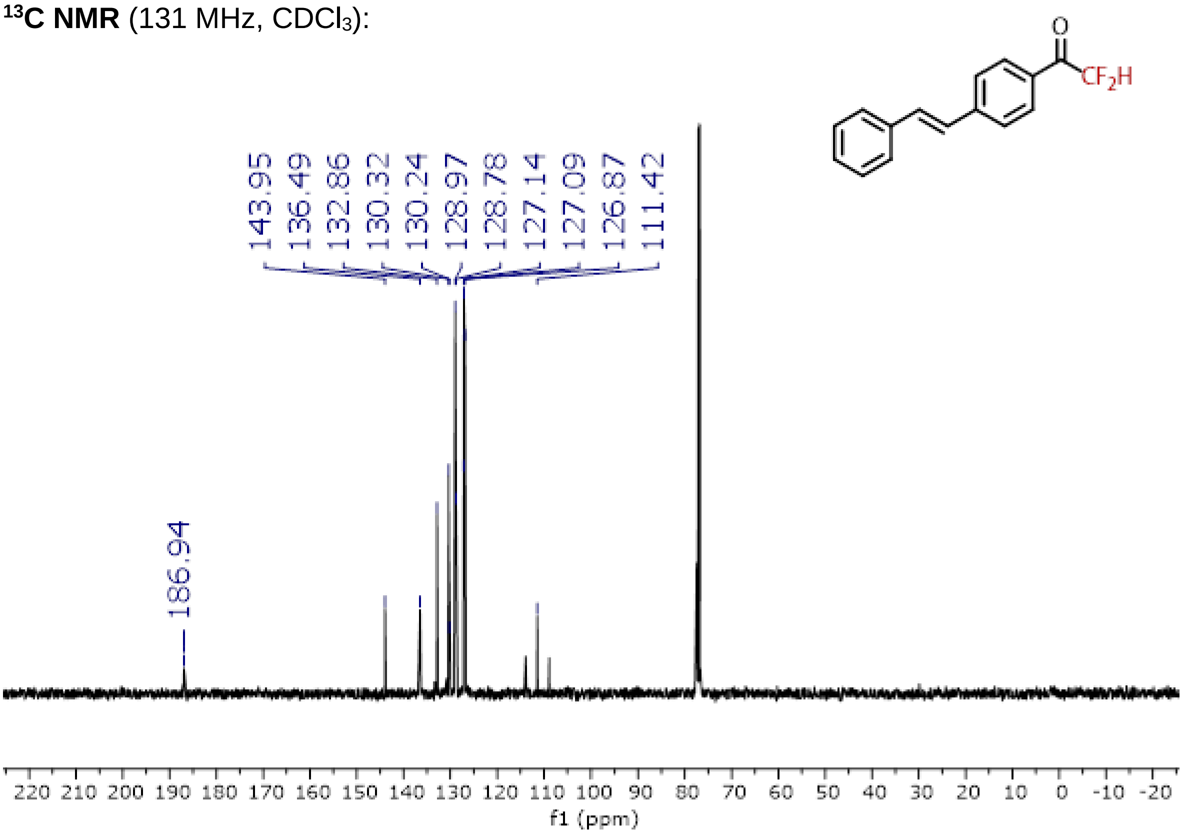

^{19}F NMR (376 MHz, $CDCl_3$):

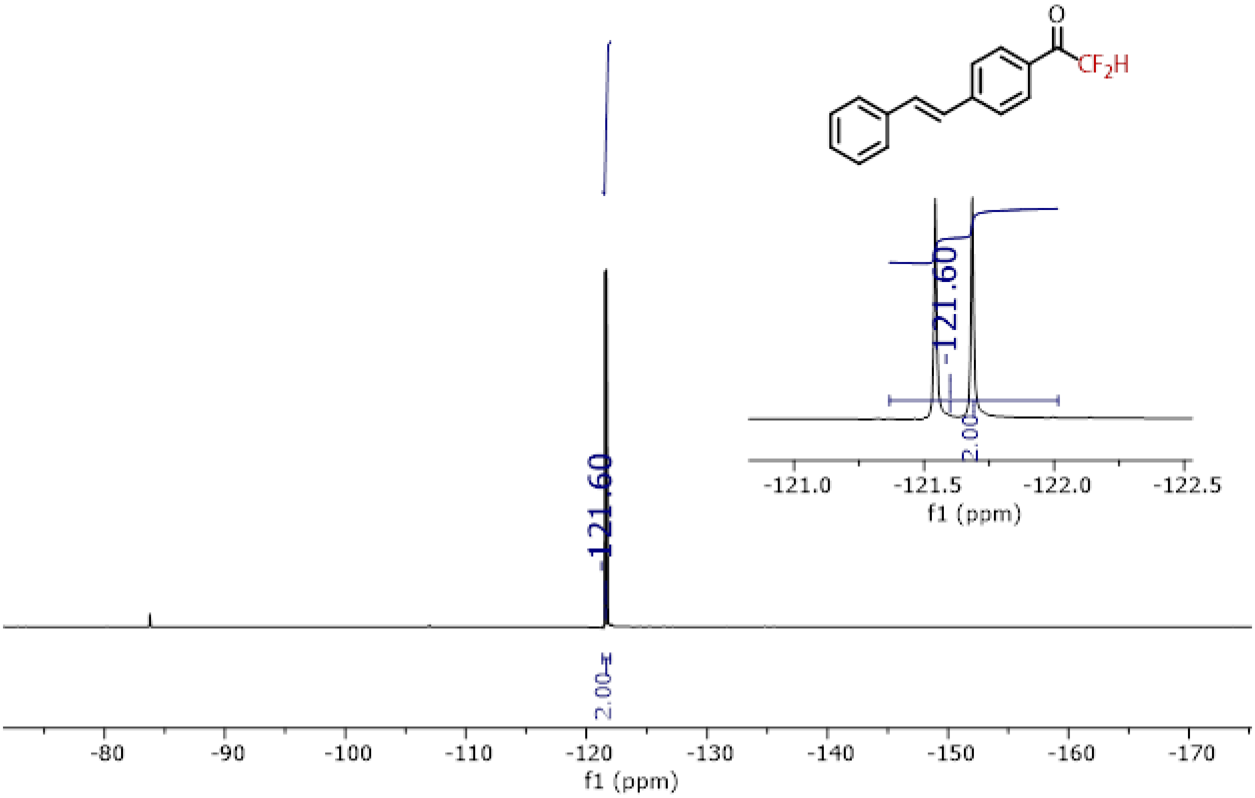

1-(Anthracen-9-yl)-2,2-difluoroethan-1-one, **3t**

^{1}H NMR (400 MHz, $CDCl_3$):

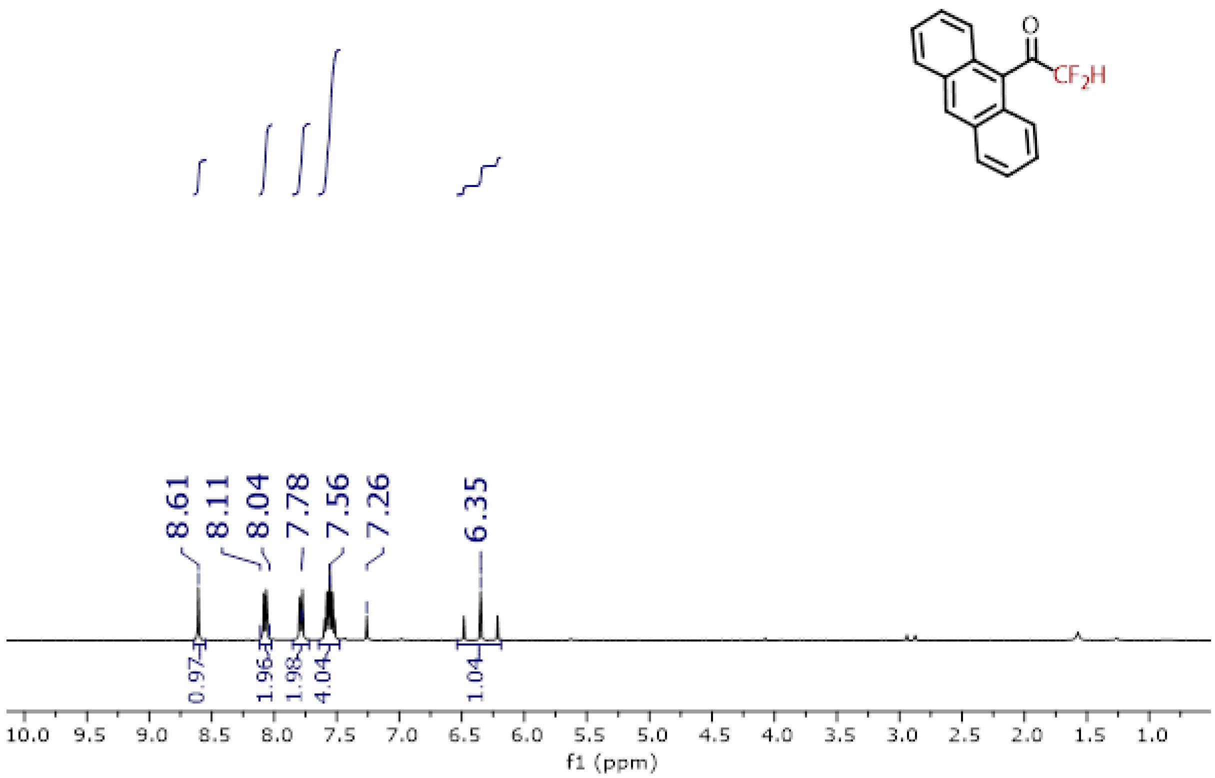

^{13}C NMR (131 MHz, $CDCl_3$):

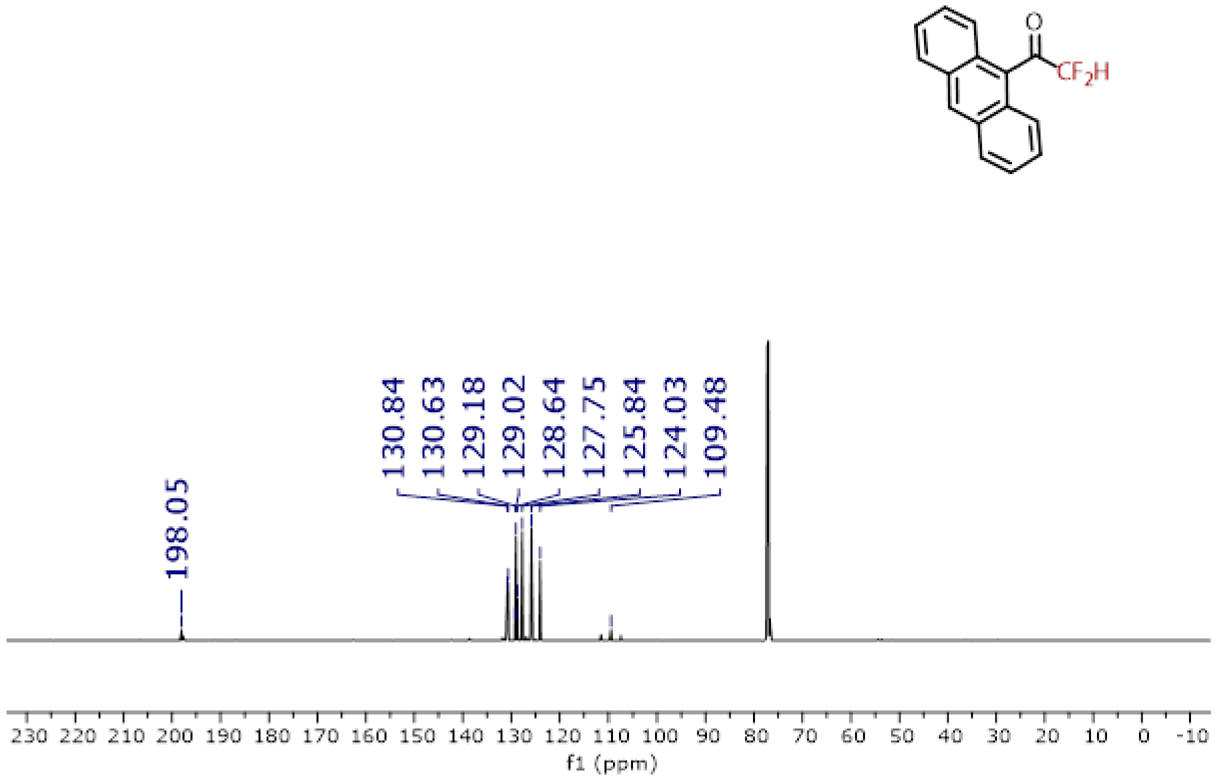

^{19}F NMR (376 MHz, $CDCl_3$):

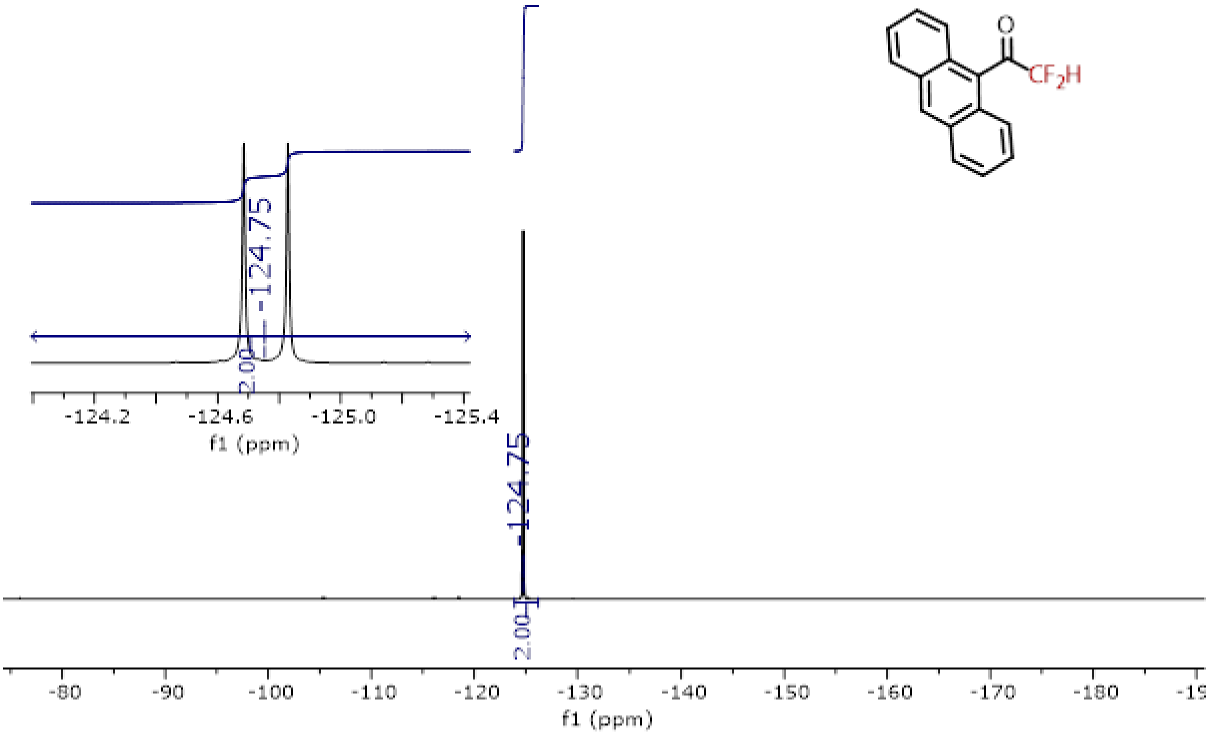

1,1'-(4-(dimethylamino)naphthalene-1,3-diyl)bis(2,2-difluoroethan-1-one), **3v**

^{1}H NMR (400 MHz, $CDCl_3$):

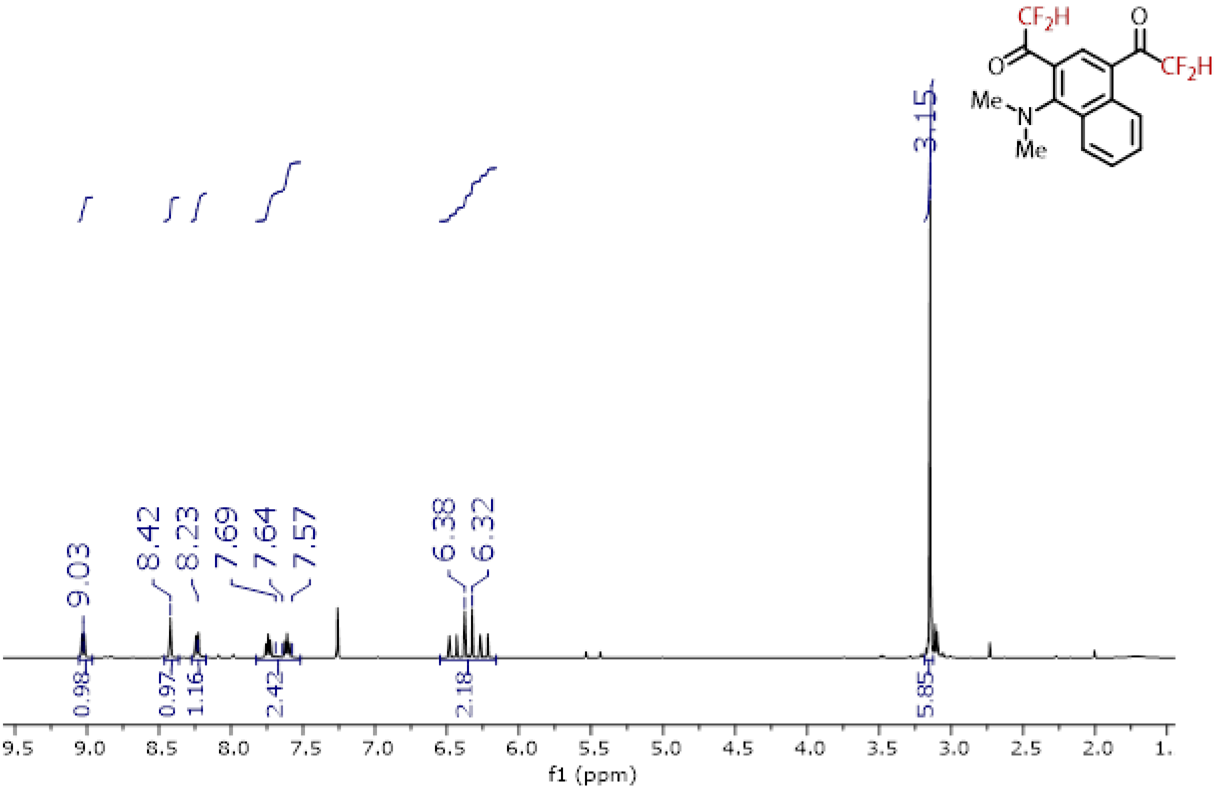

^{13}C NMR (131 MHz, $CDCl_3$):

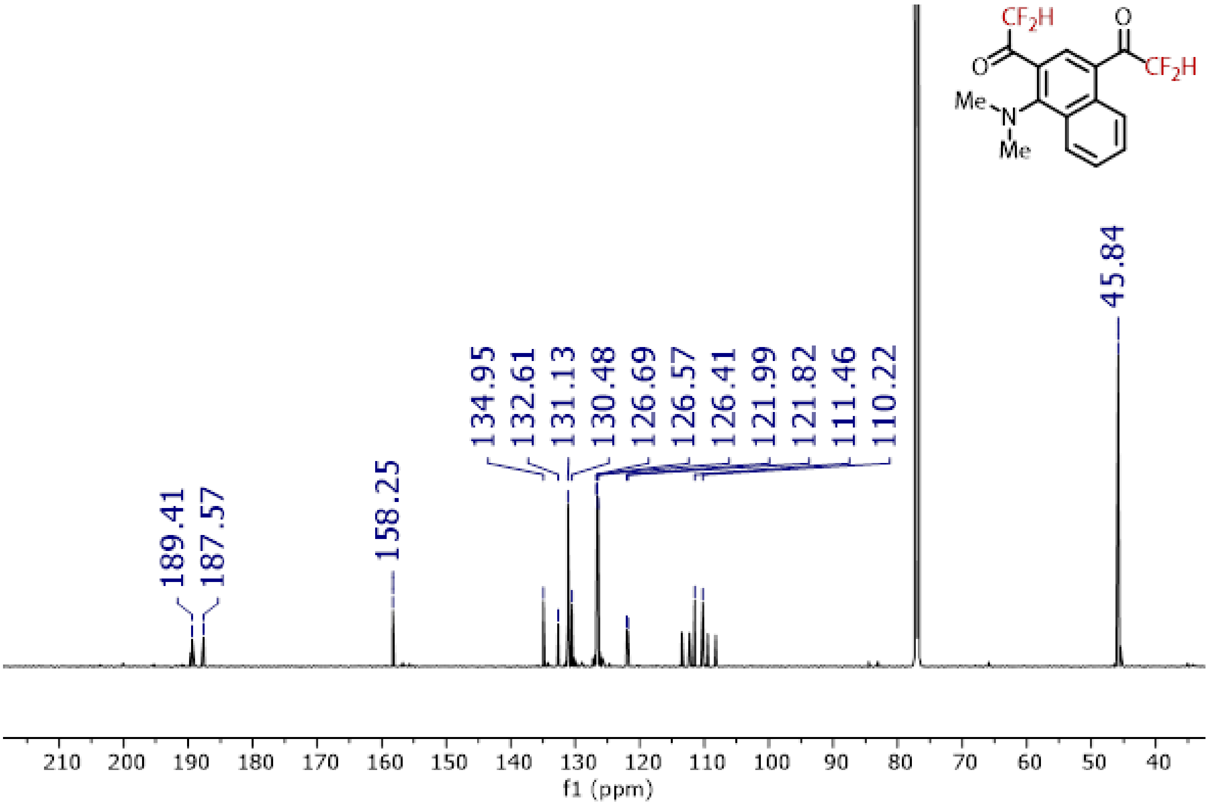

^{19}F NMR (376 MHz, $CDCl_3$):

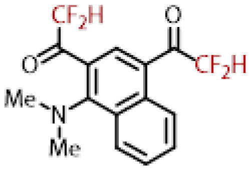

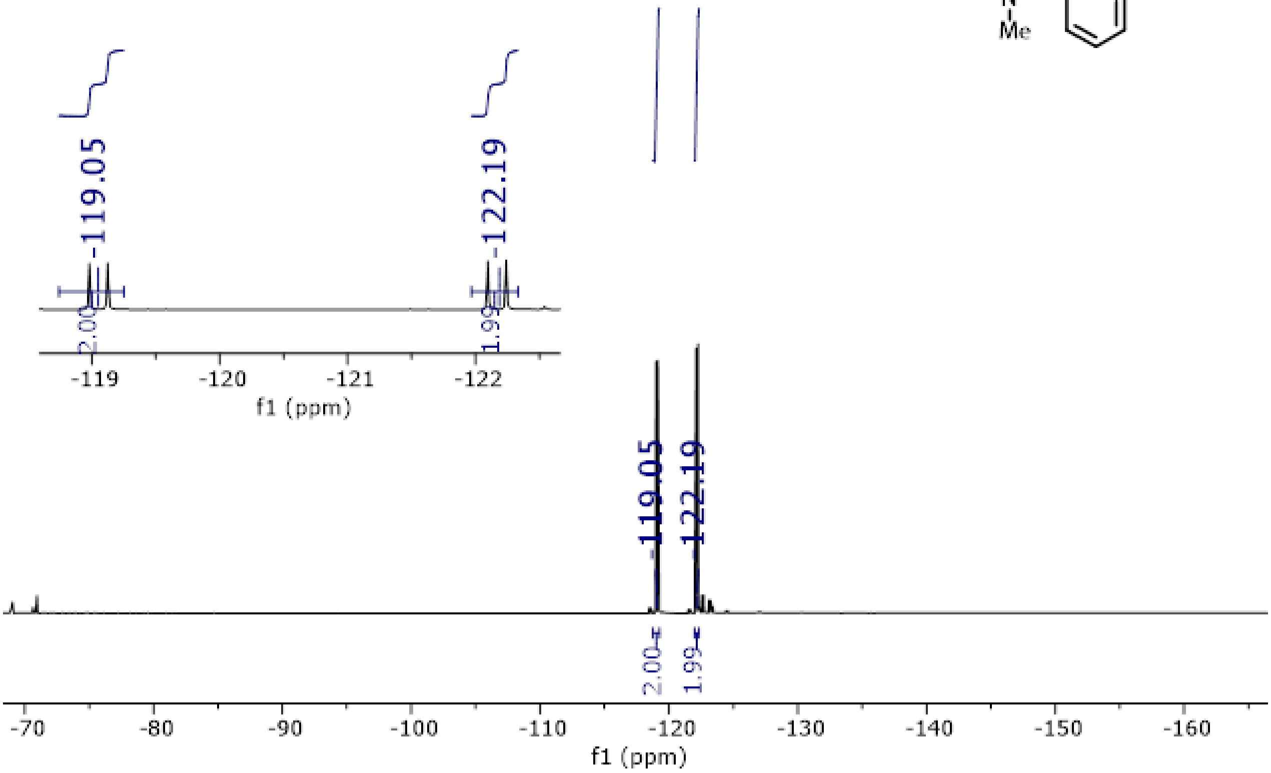

tert-Butyl 3-(2,2-difluoroacetyl)-1H-indole-1-carboxylate, **3w**

^{1}H NMR (400 MHz, $CDCl_3$):

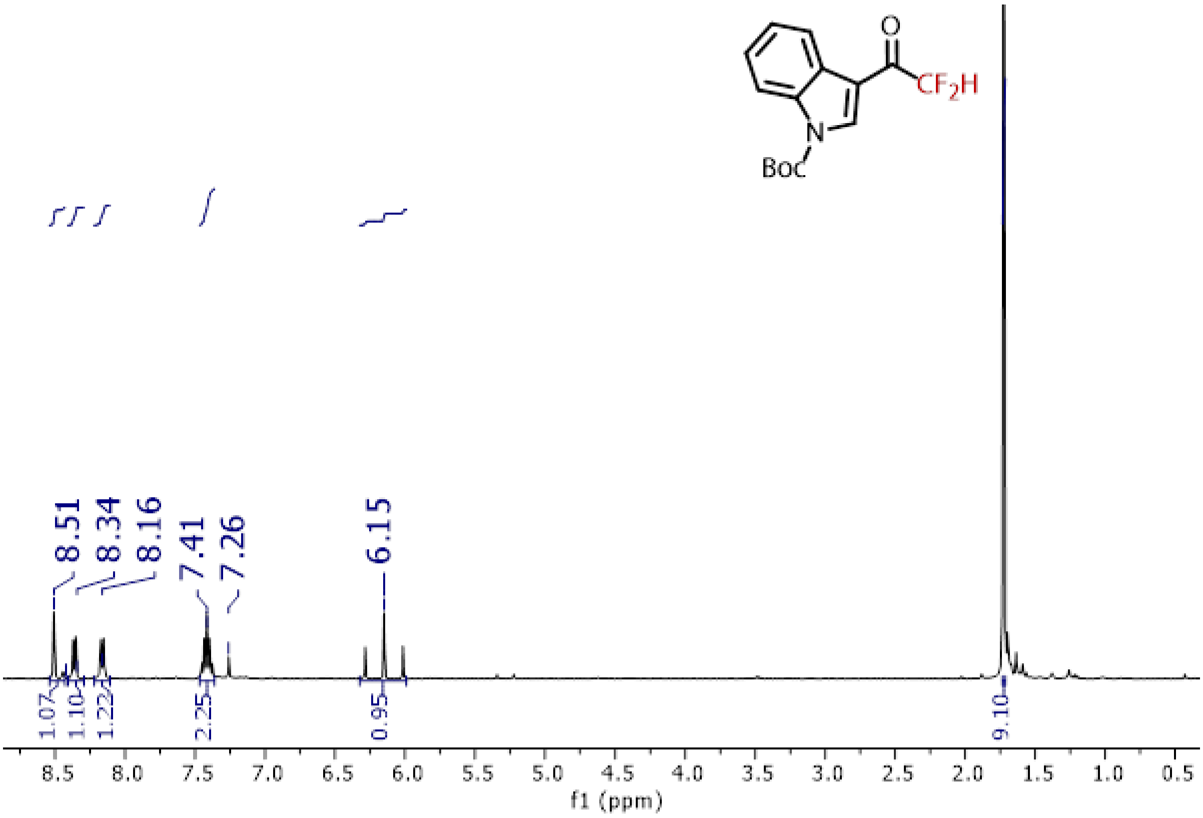

^{13}C NMR (131 MHz, $CDCl_3$):

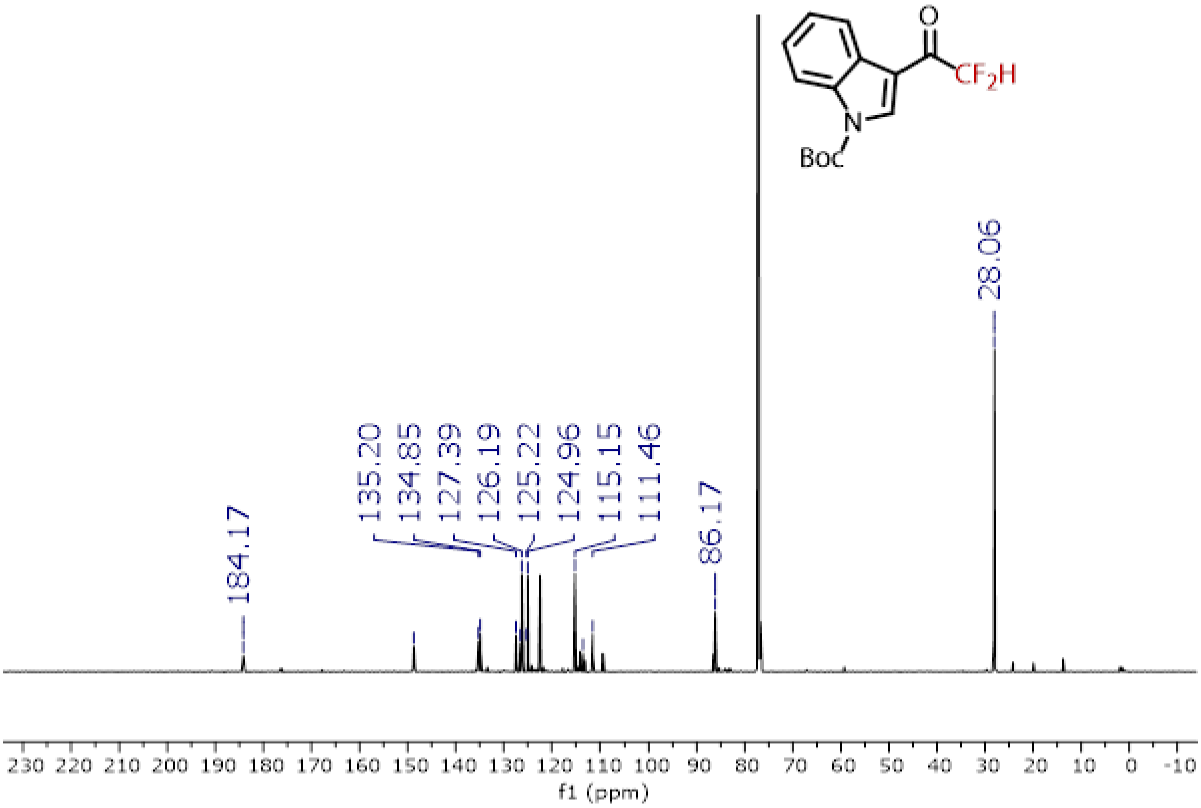

^{19}F NMR (376 MHz, $CDCl_3$):

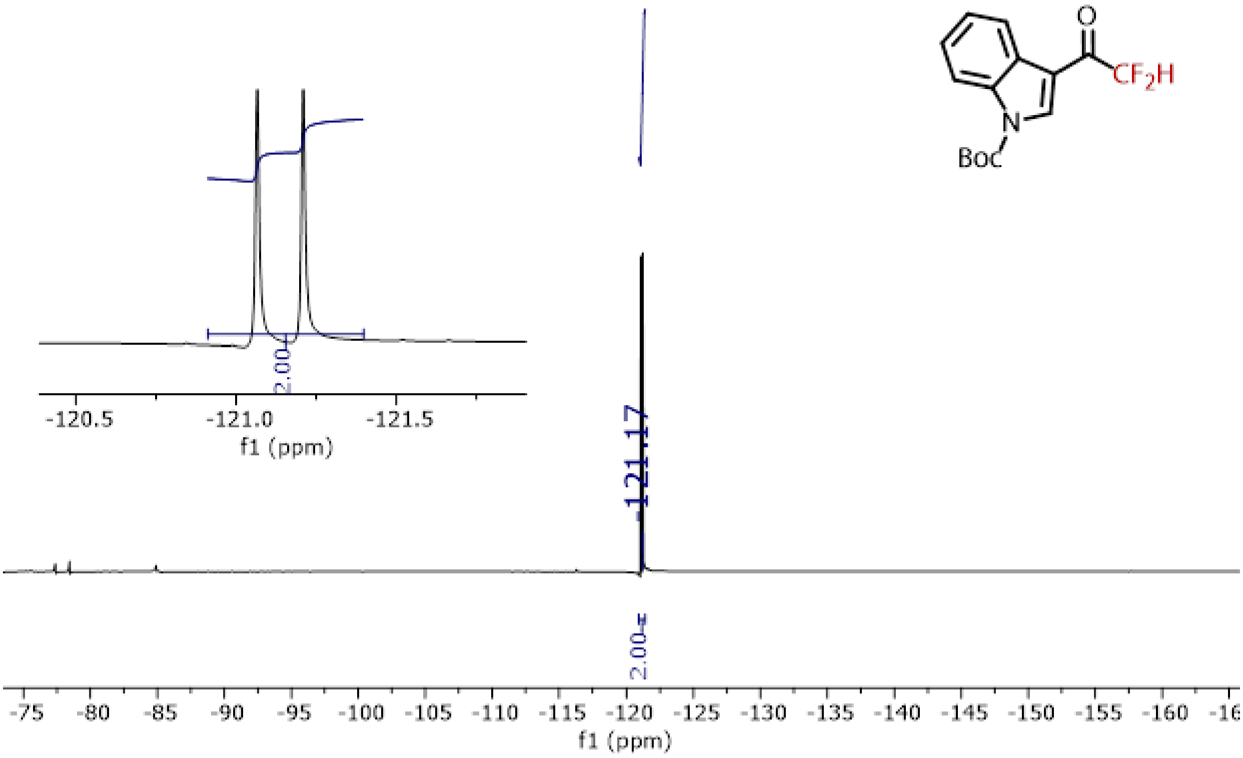

2,2-Difluoro-1-(1-(perfluoropyridin-4-yl)-1H-indol-3-yl)ethan-1-one, **3x**

^{1}H NMR (400 MHz, $CDCl_3$):

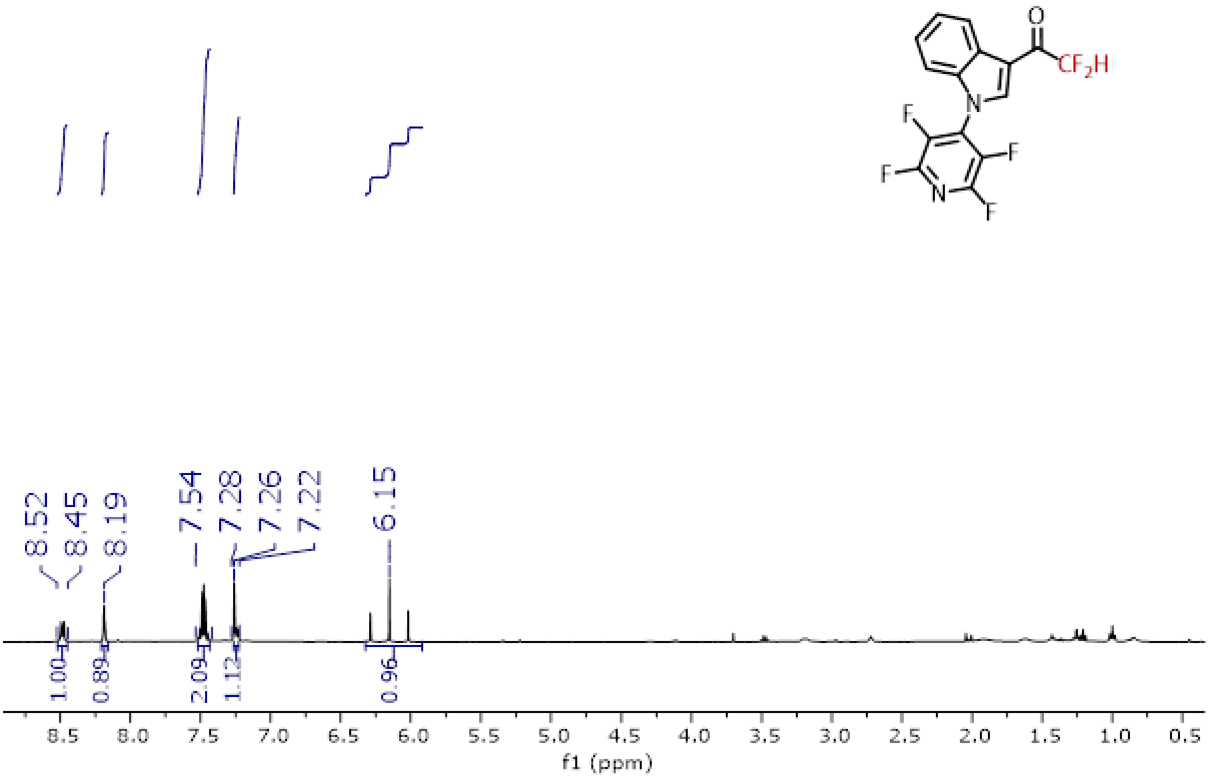

^{13}C NMR (131 MHz, $CDCl_3$):

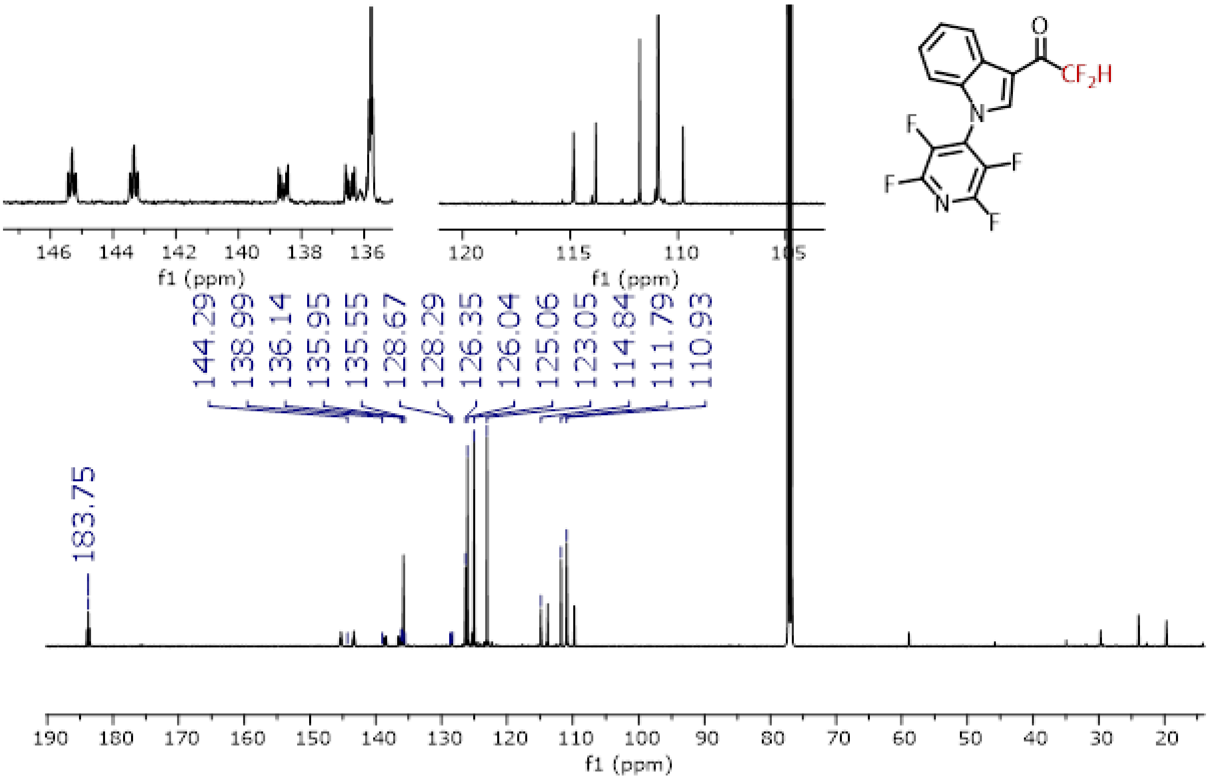

^{19}**F NMR** (376 MHz, $CDCl_3$):

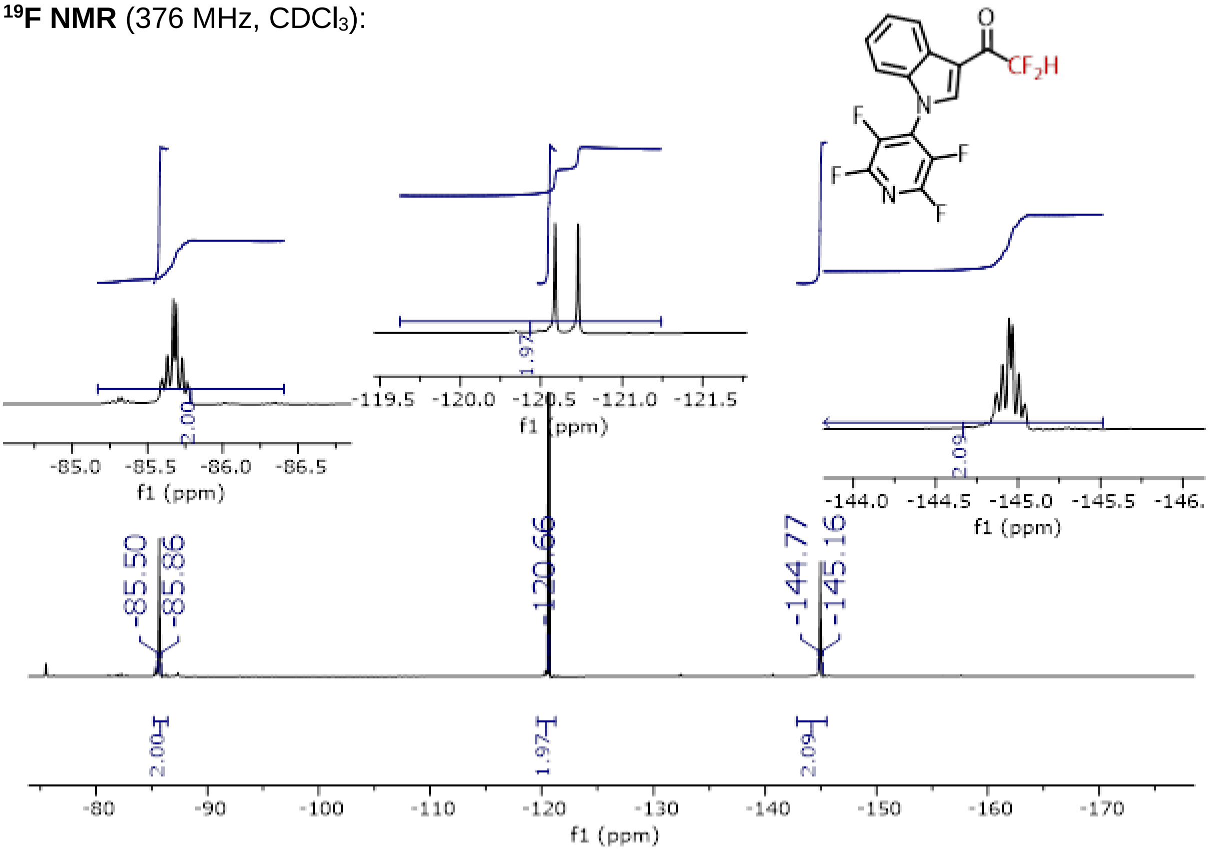

1-(1-benzyl-1H-indol-3-yl)-2,2-difluoroethan-1-one, **3y**

^{1}H NMR (400 MHz, $CDCl_3$):

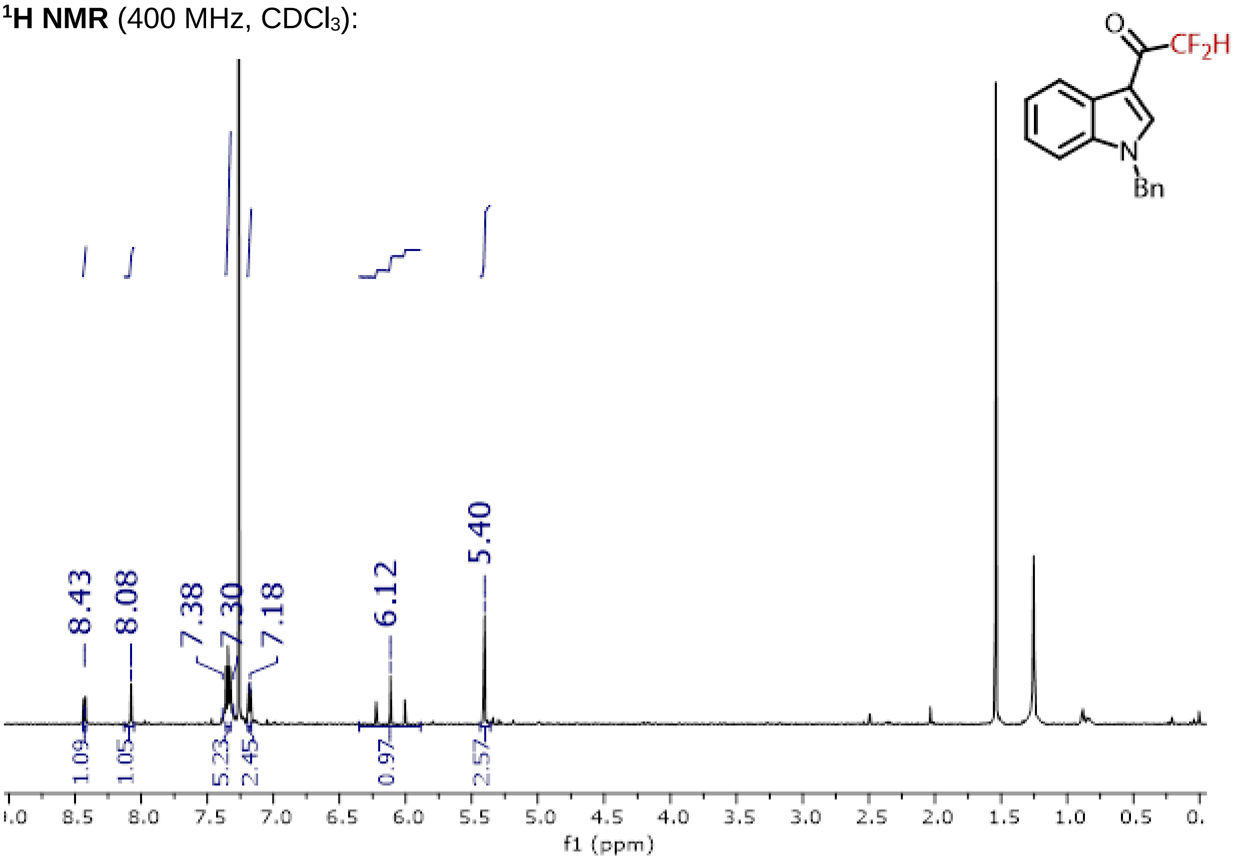

^{13}C NMR (131 MHz, $CDCl_3$):

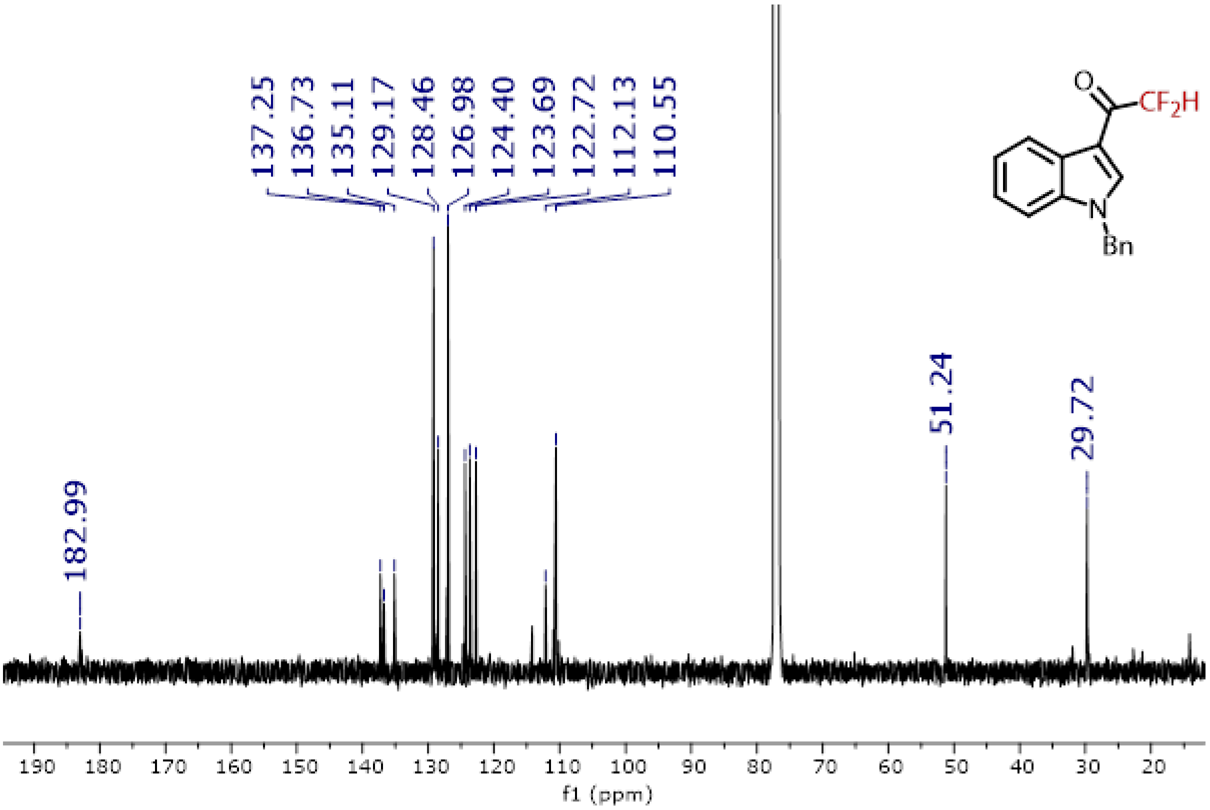

^{19}F NMR (376 MHz, $CDCl_3$):

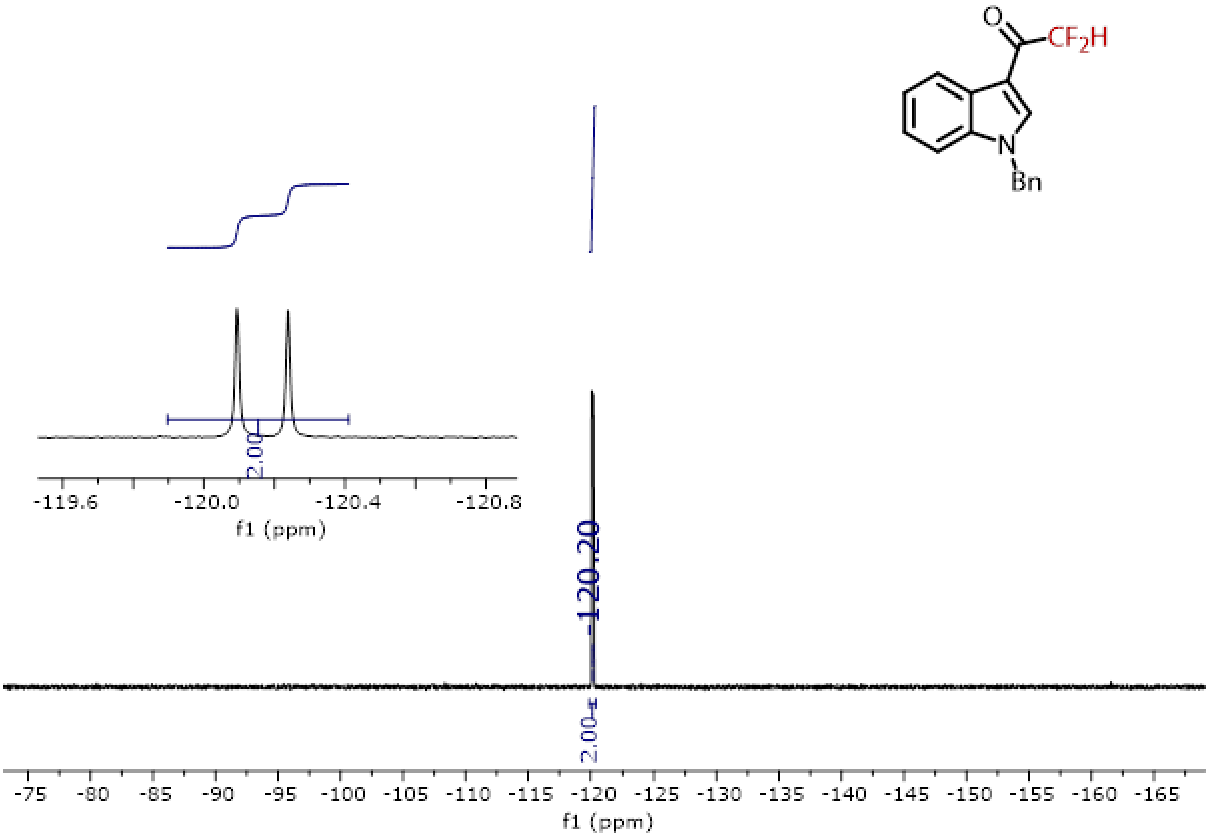

2,2-difluoro-1-(6-methoxy-1-methyl-1H-indol-3-yl)ethan-1-one , **3aa**

^{1}H NMR (400 MHz, $CDCl_3$):

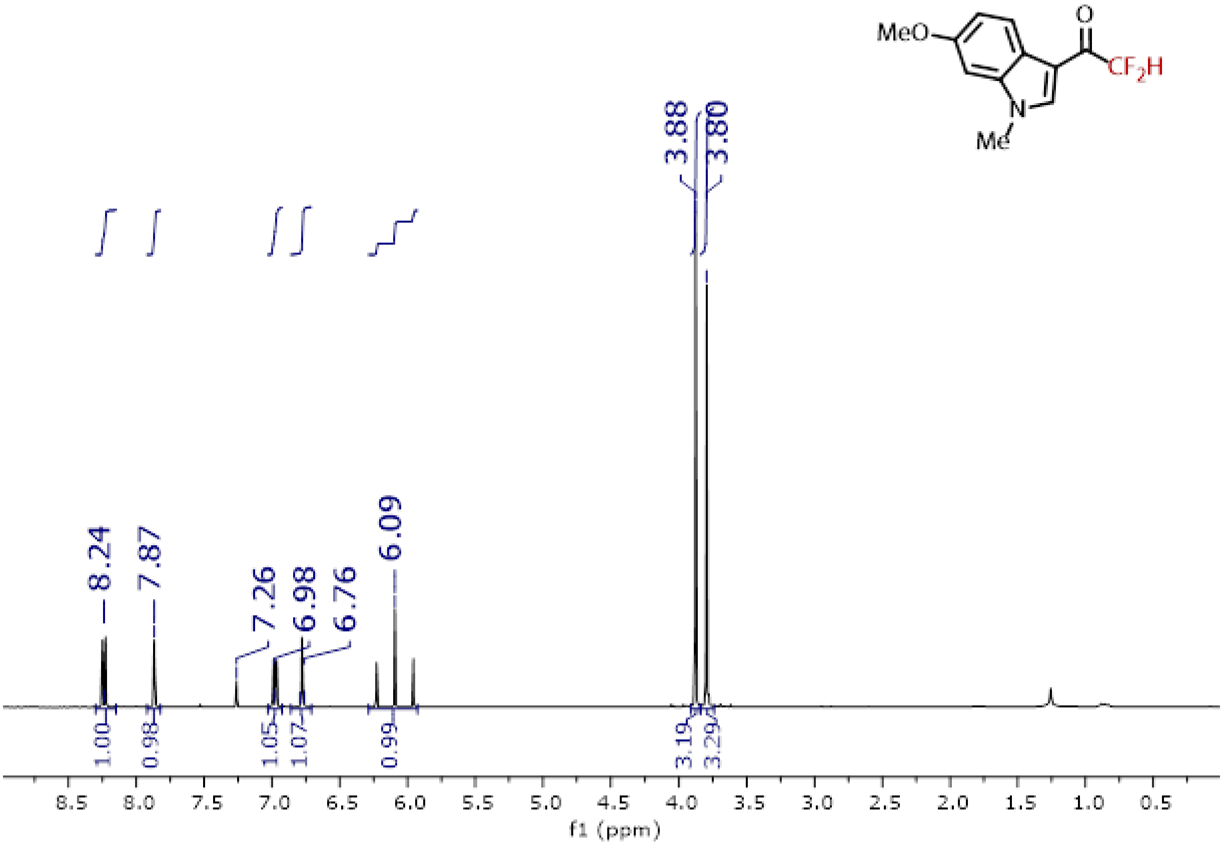

^{13}C NMR (131 MHz, $CDCl_3$):

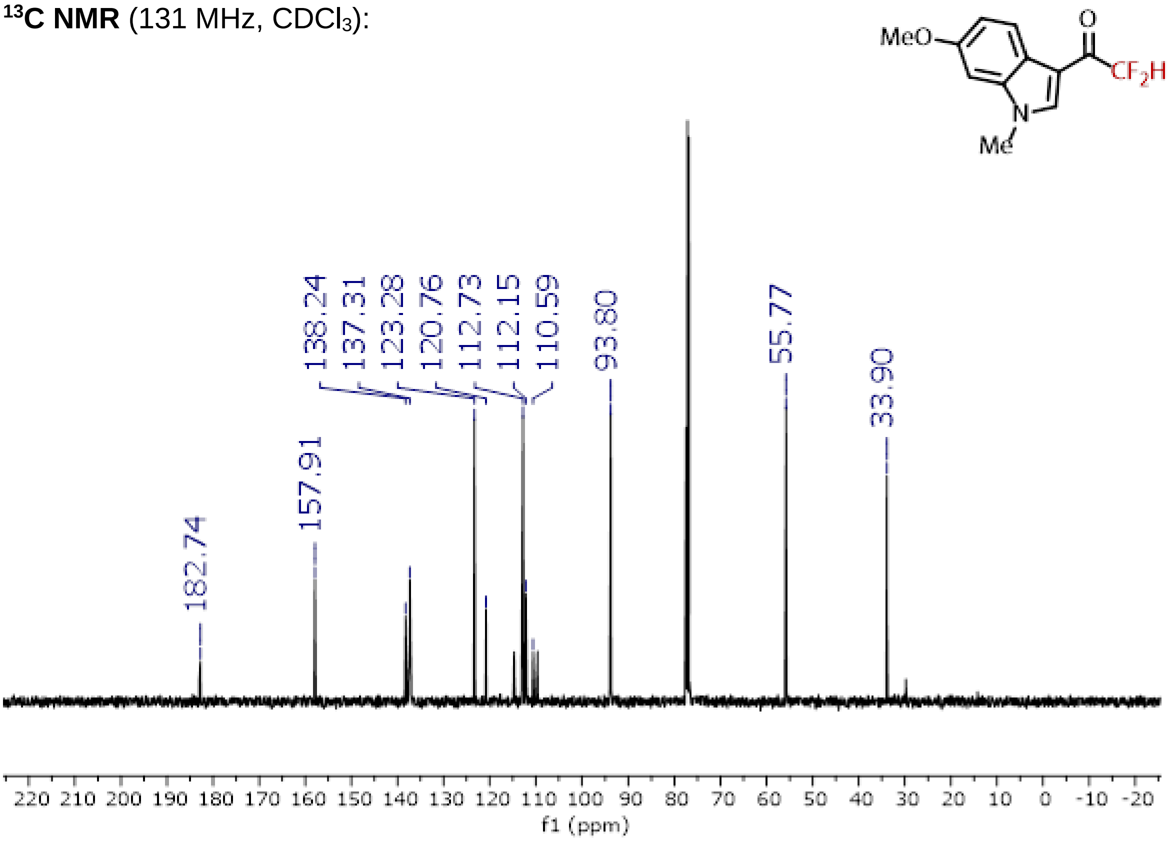

^{19}F NMR (376 MHz, $CDCl_3$):

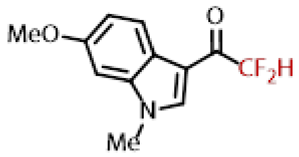

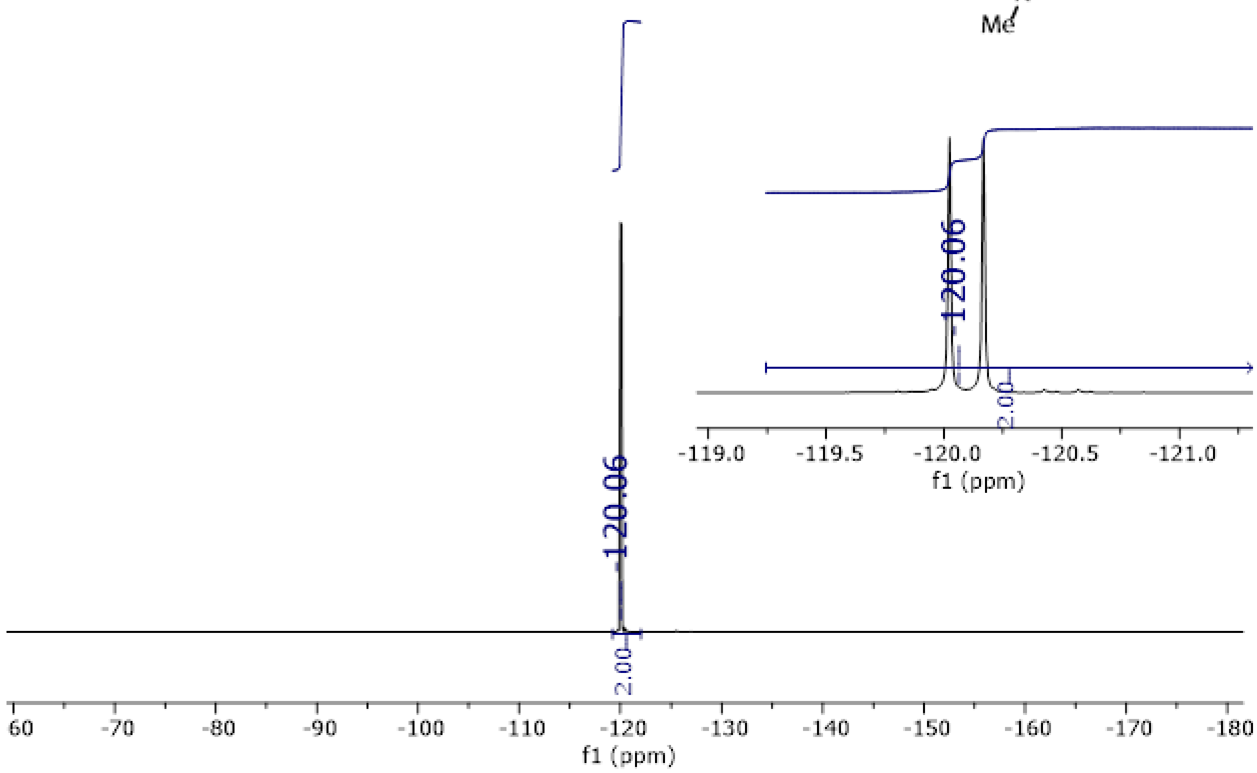

3-(2,2-difluoroacetyl)-1-methyl-1H-indole-5-carbonitrile, **3ab**

^{1}H NMR (400 MHz, $CDCl_3$):

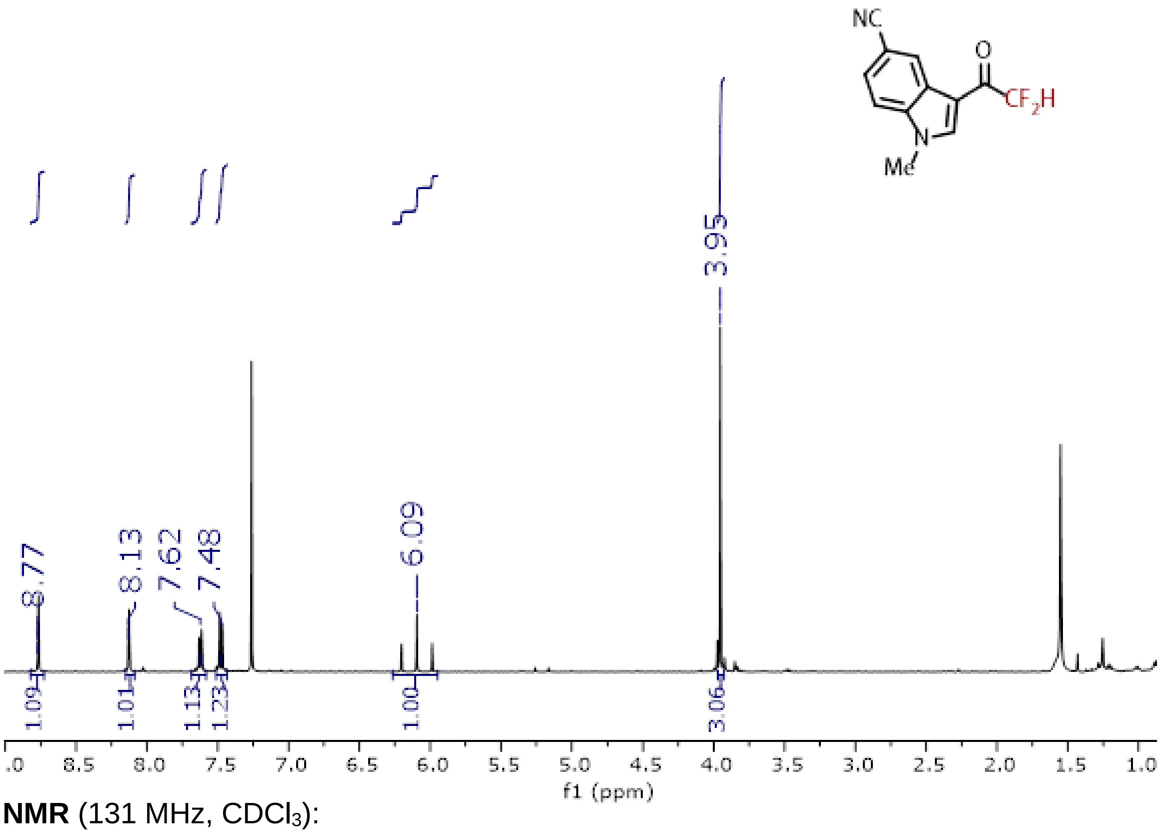

^{13}C NMR (131 MHz, $CDCl_3$):

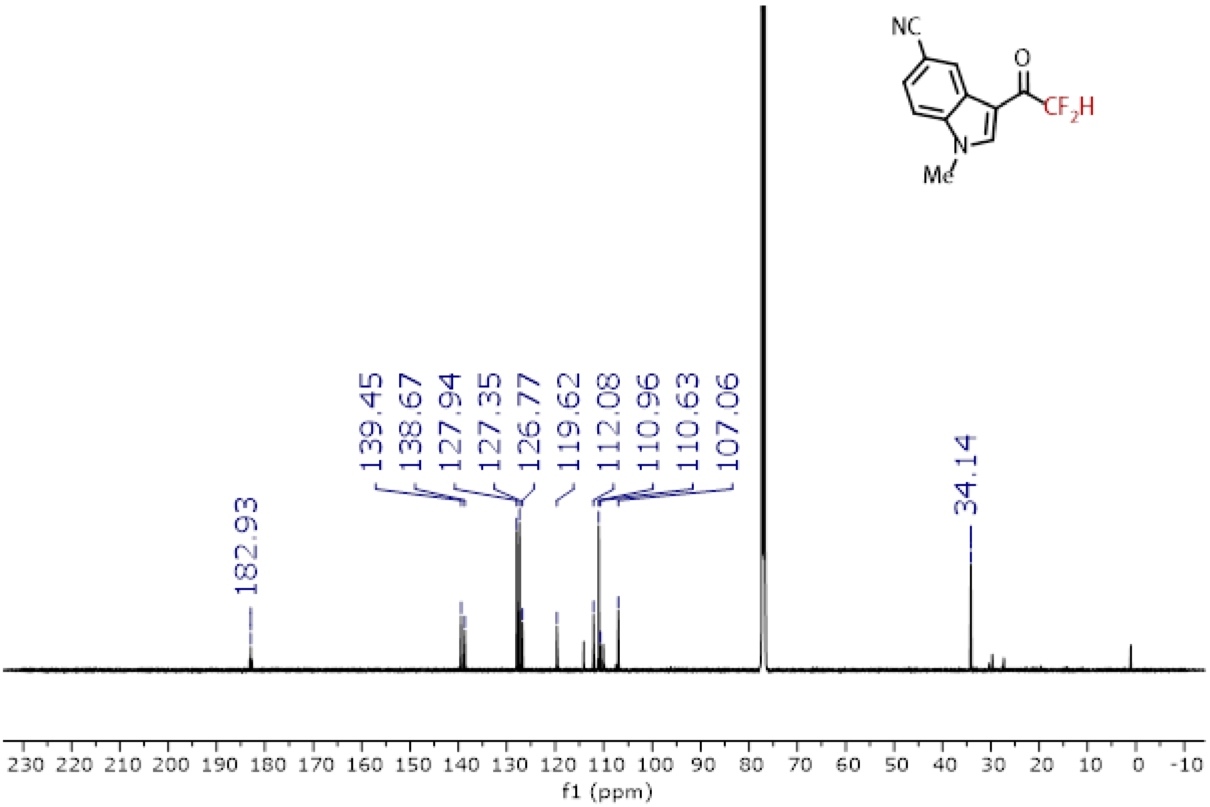

^{19}F NMR (376 MHz, $CDCl_3$):

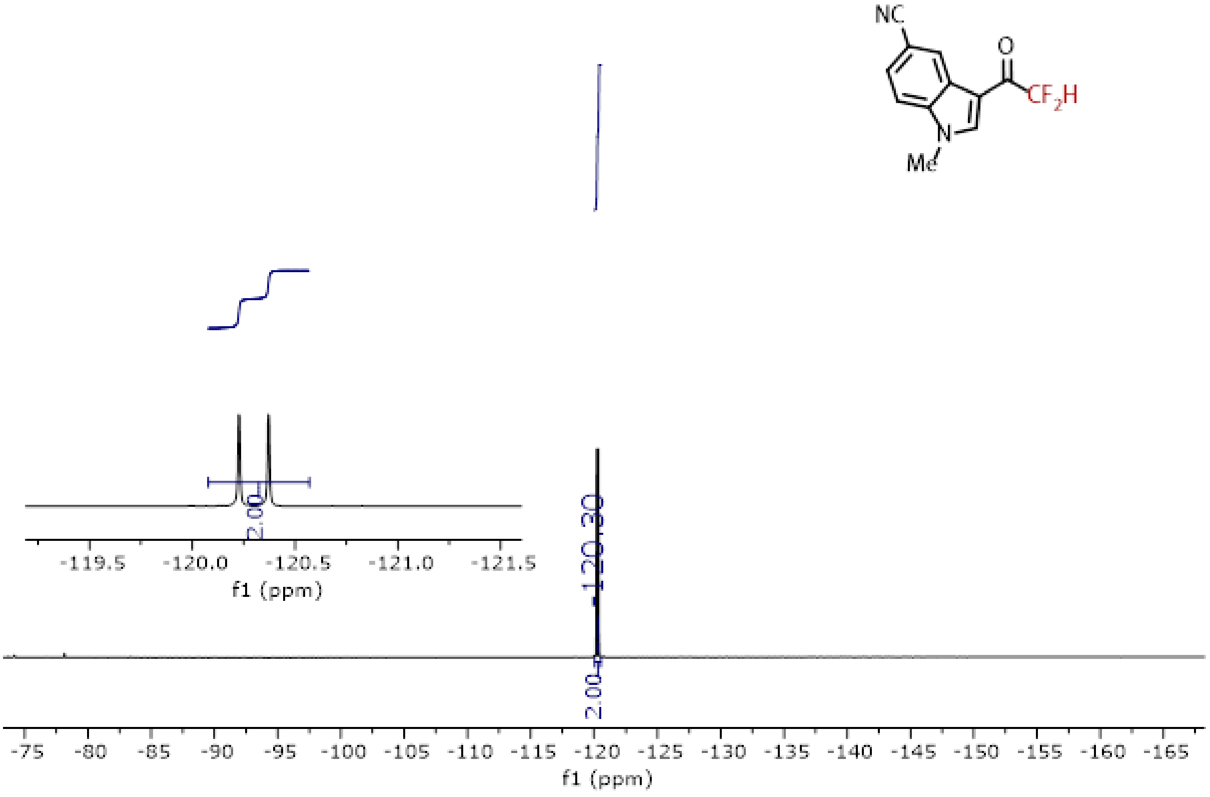

2,2-difluoro-1-(4-fluoro-1-methyl-1H-indol-3-yl)ethan-1-one, **3ac**

^{1}H NMR (400 MHz, $CDCl_3$):

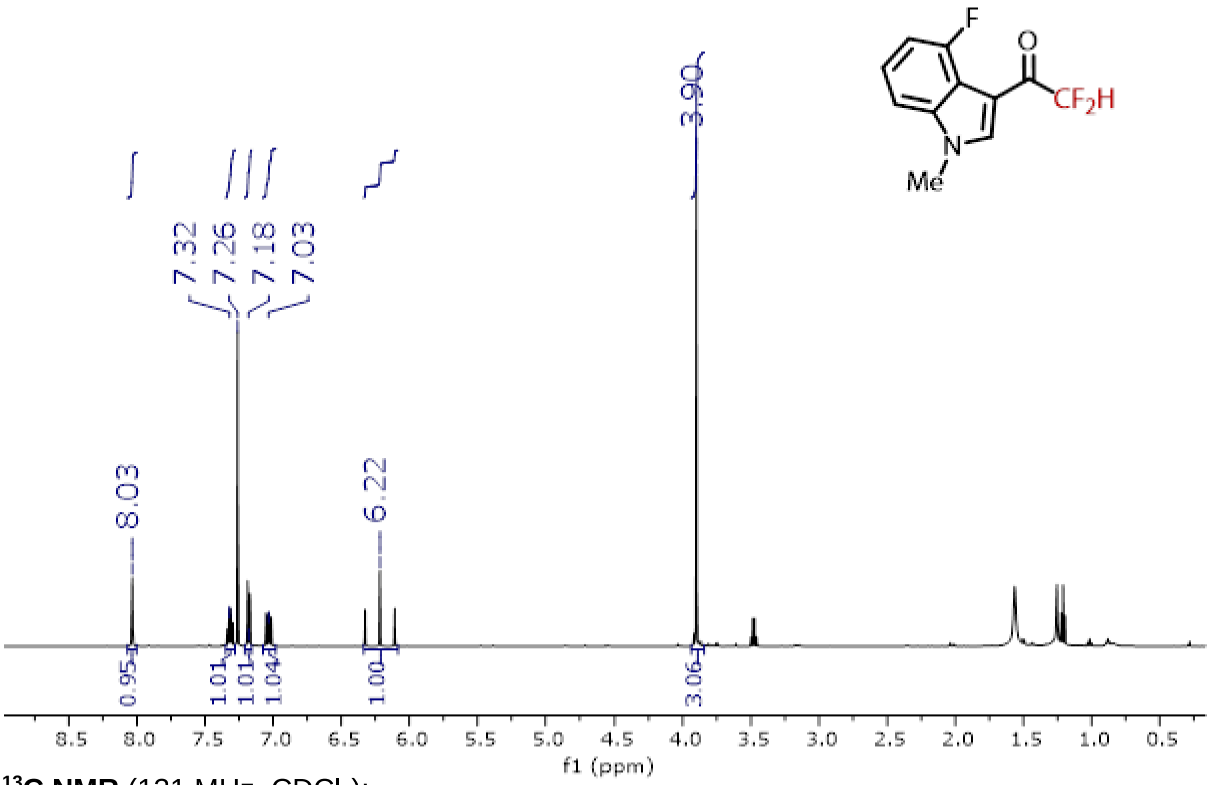

^{13}C NMR (131 MHz, $CDCl_3$):

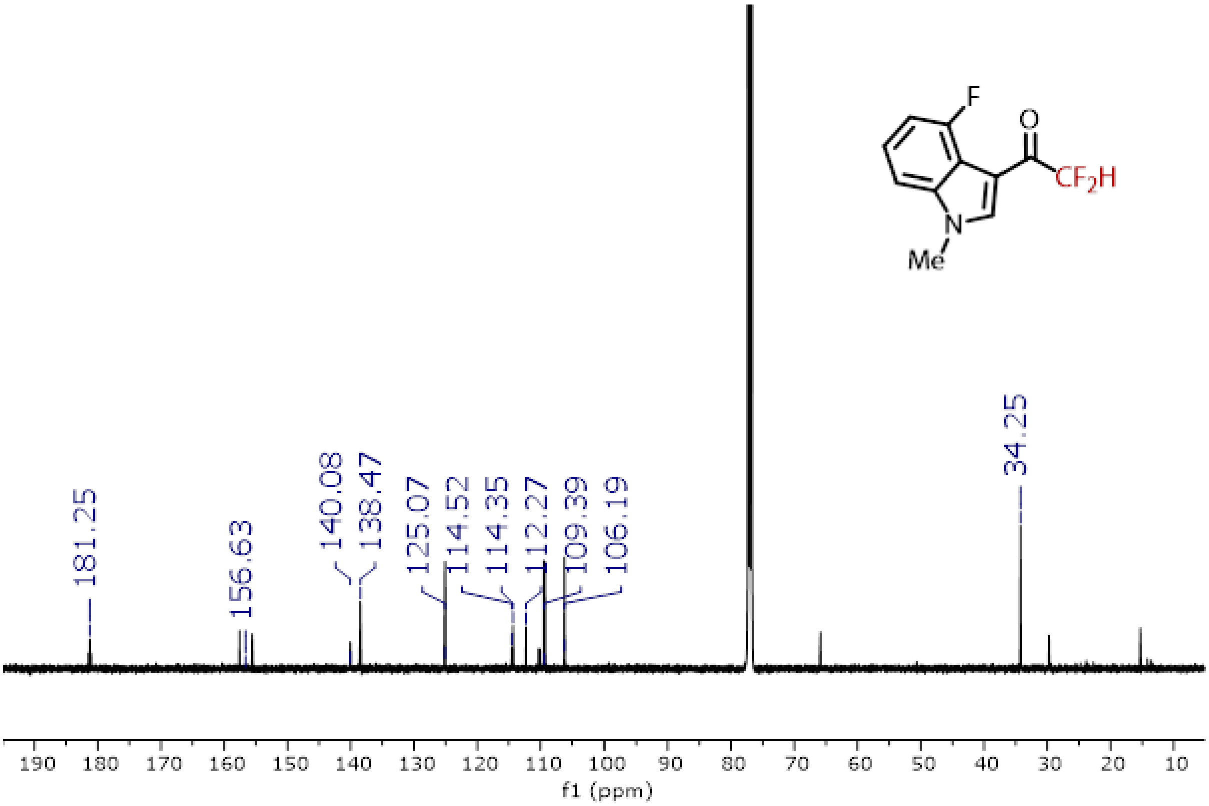

^{19}F NMR (376 MHz, $CDCl_3$):

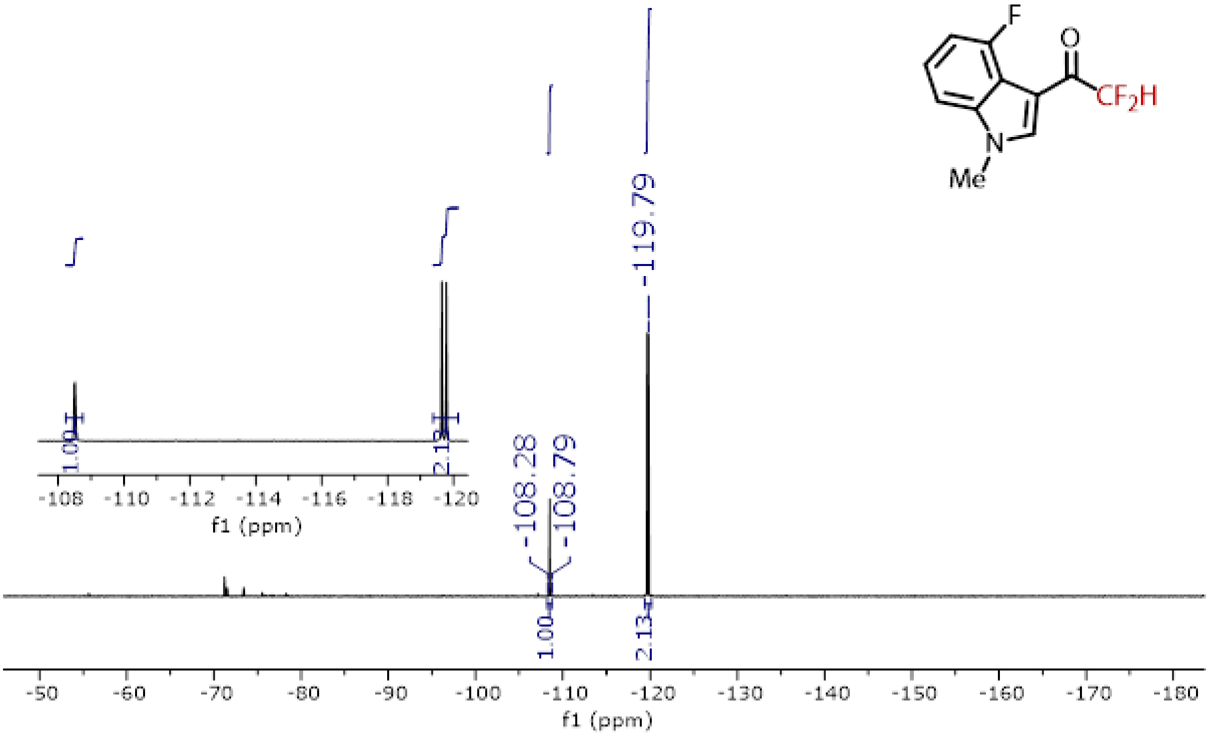

1-(4-chloro-1-methyl-1H-indol-3-yl)-2,2-difluoroethan-1-one, **3ad**

^{1}H NMR (400 MHz, $CDCl_3$):

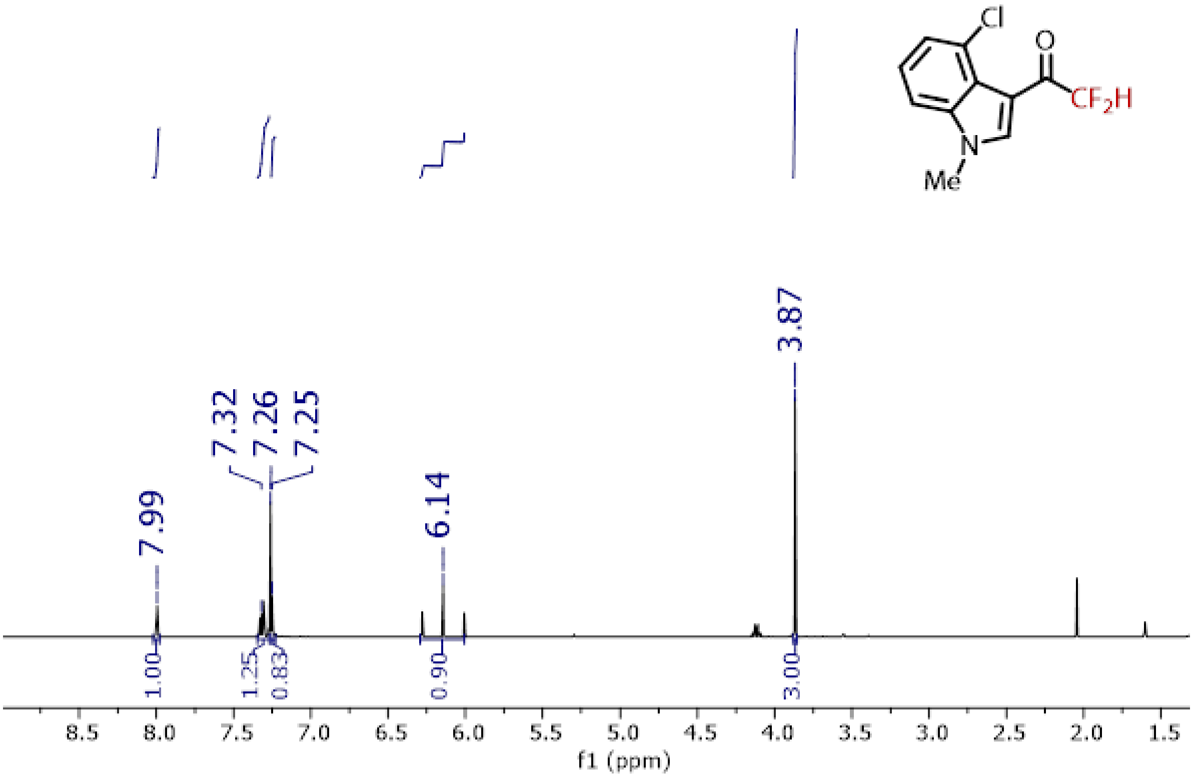

^{13}C NMR (131 MHz, $CDCl_3$):

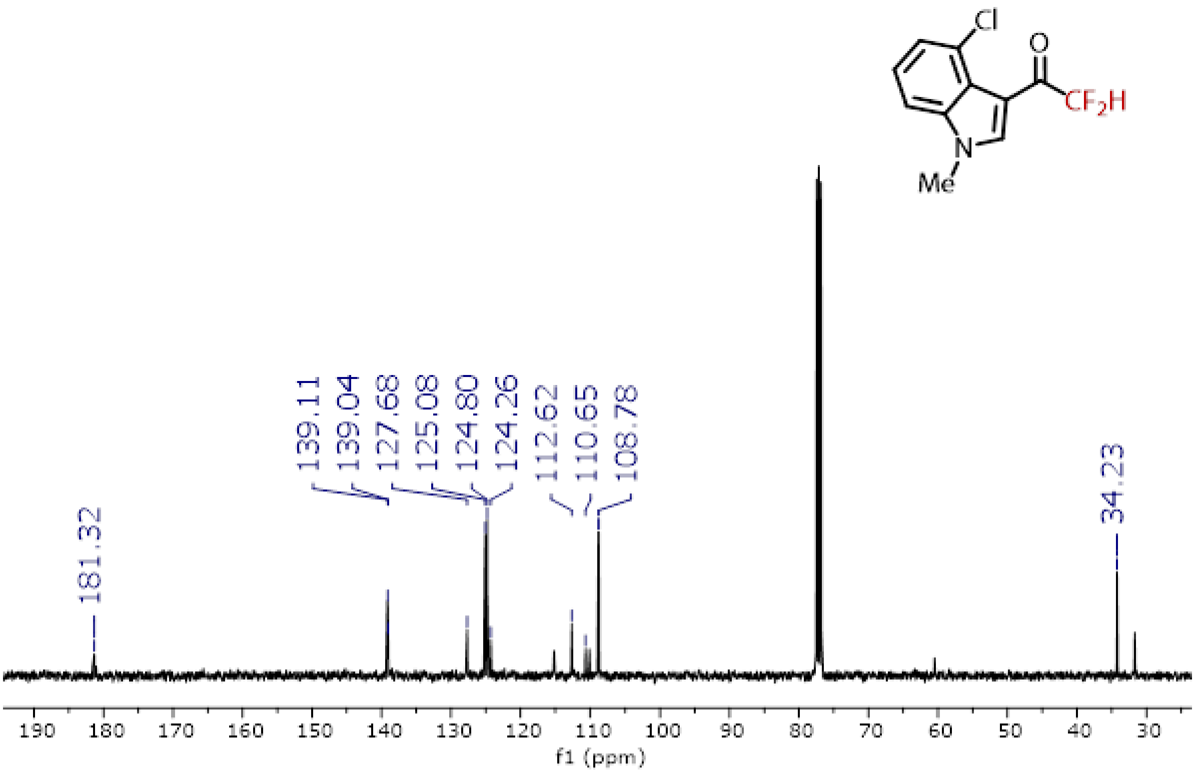

^{19}F NMR (376 MHz, $CDCl_3$):

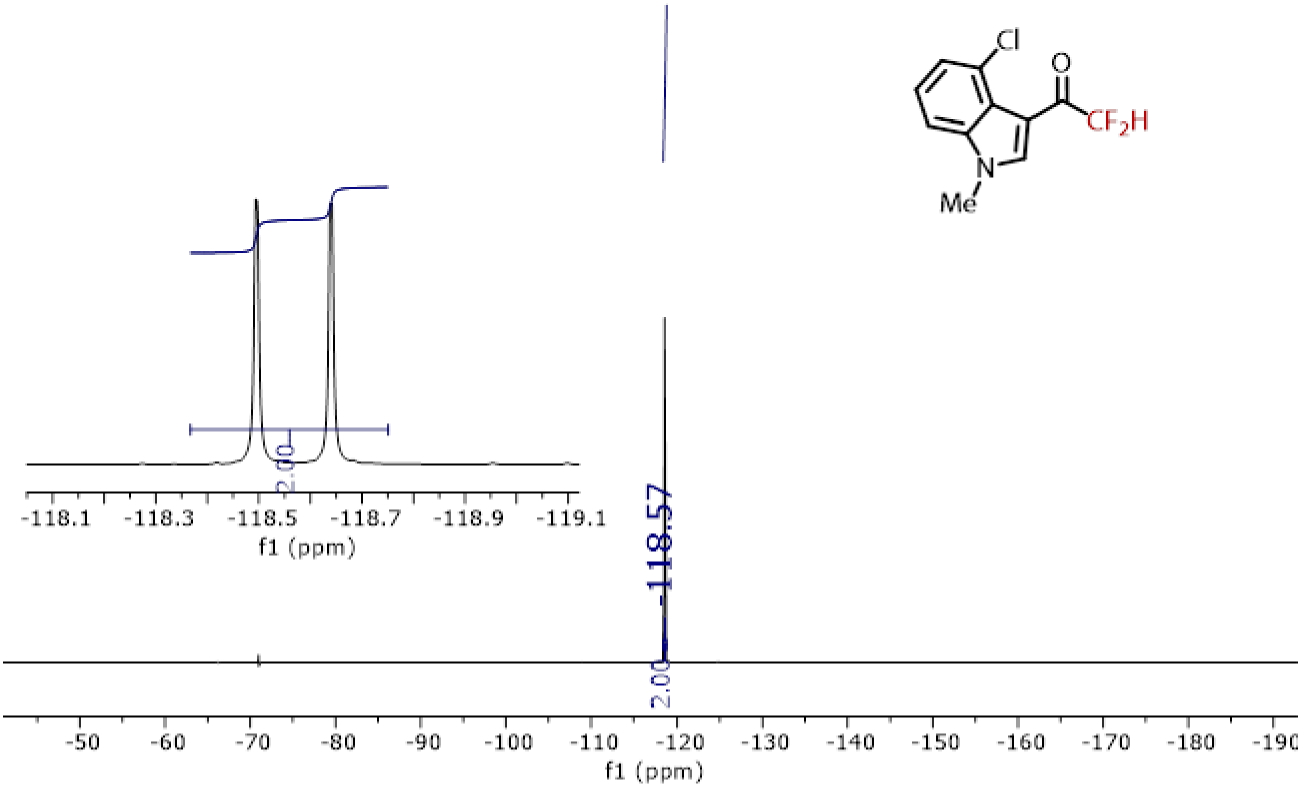

1-(4-Bromo-1-methyl-1H-indol-3-yl)-2,2-difluoroethan-1-one, **3ae**

^{1}**H NMR** (400 MHz, $CDCl_3$):

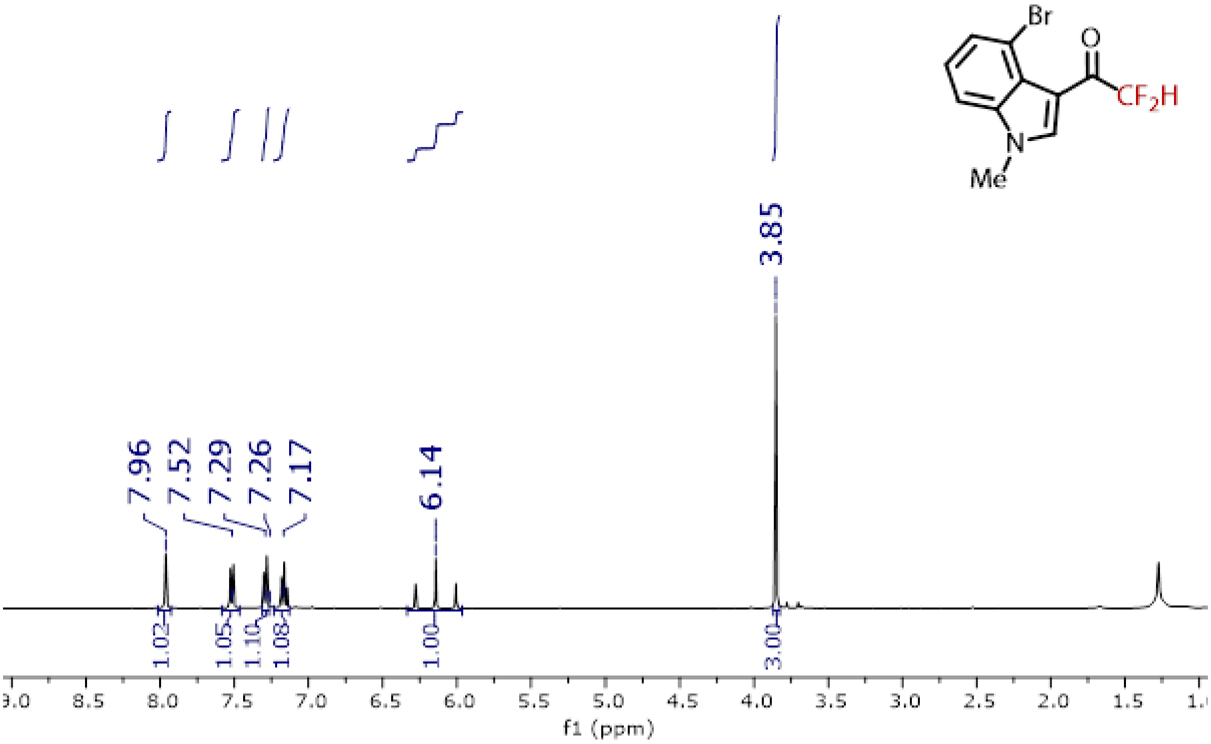

^{13}**C NMR** (131 MHz, $CDCl_3$):

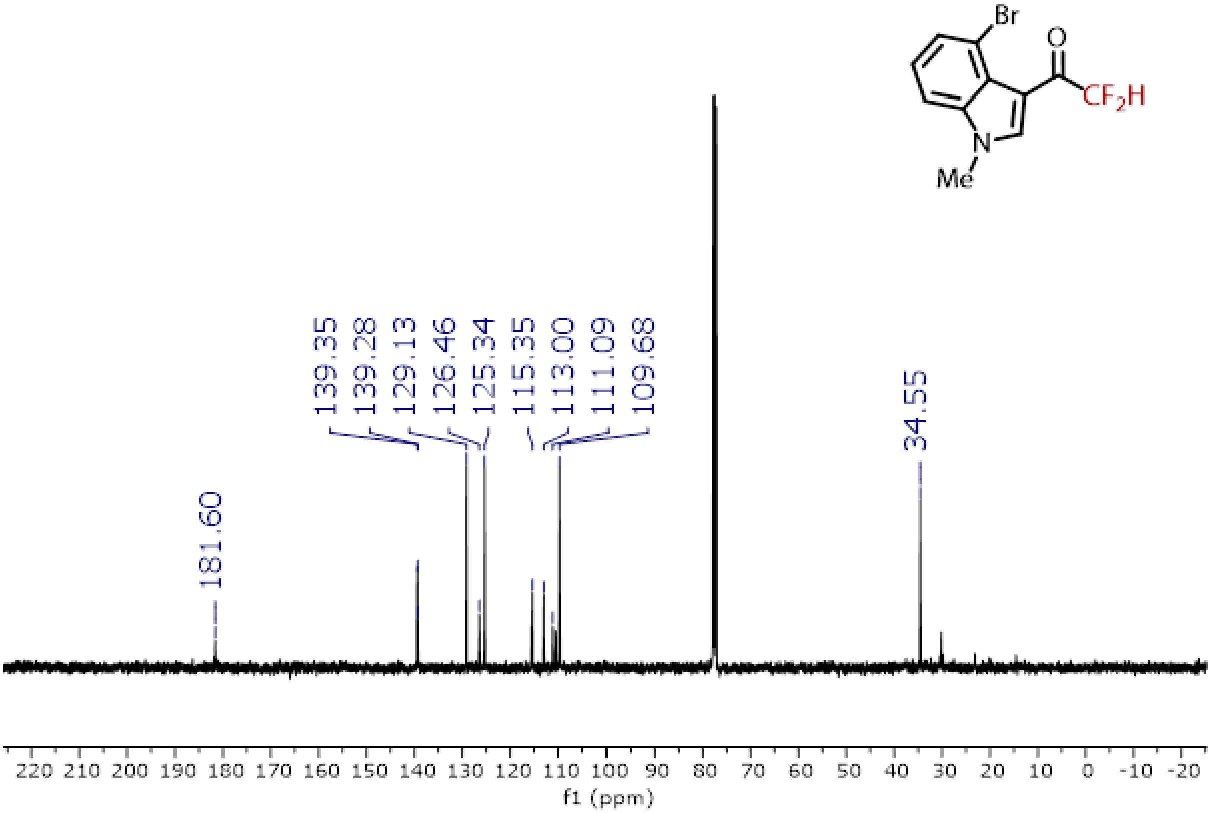

^{19}F NMR (376 MHz, $CDCl_3$):

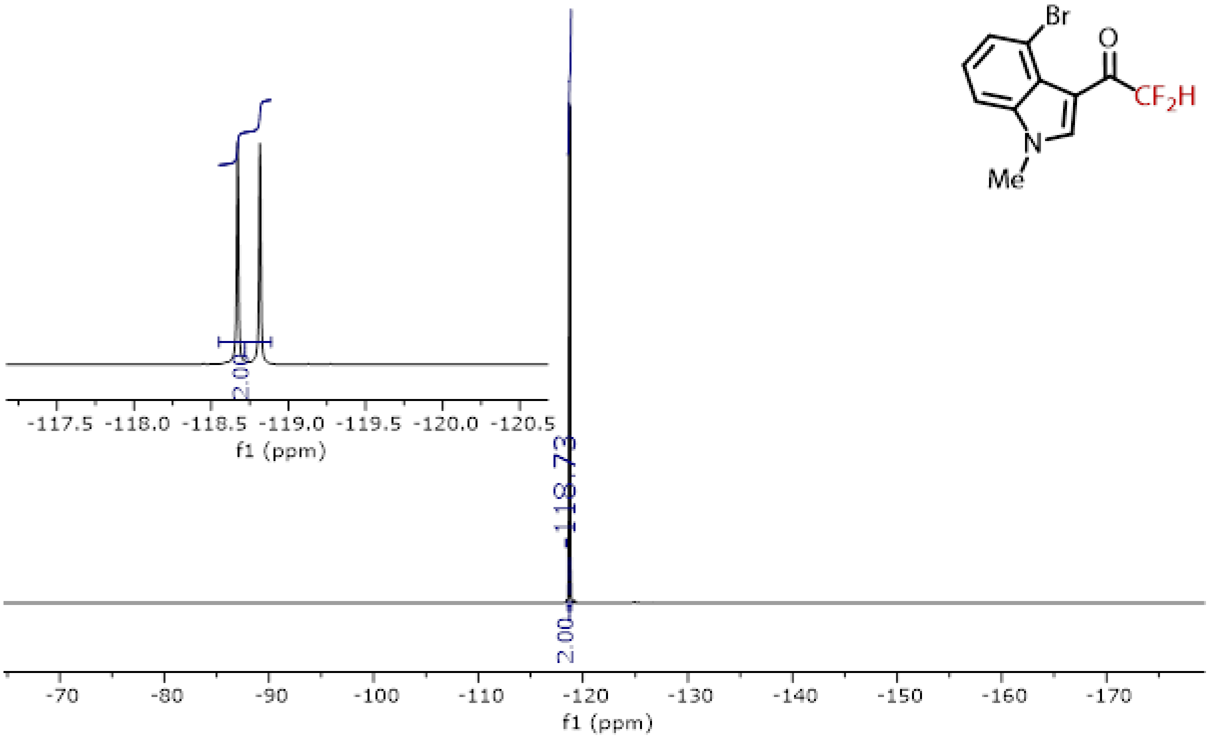

1-(5-Bromo-1-methyl-1H-indol-3-yl)-2,2-difluoroethan-1-one, **3ae**

^{1}H NMR (400 MHz, $CDCl_3$):

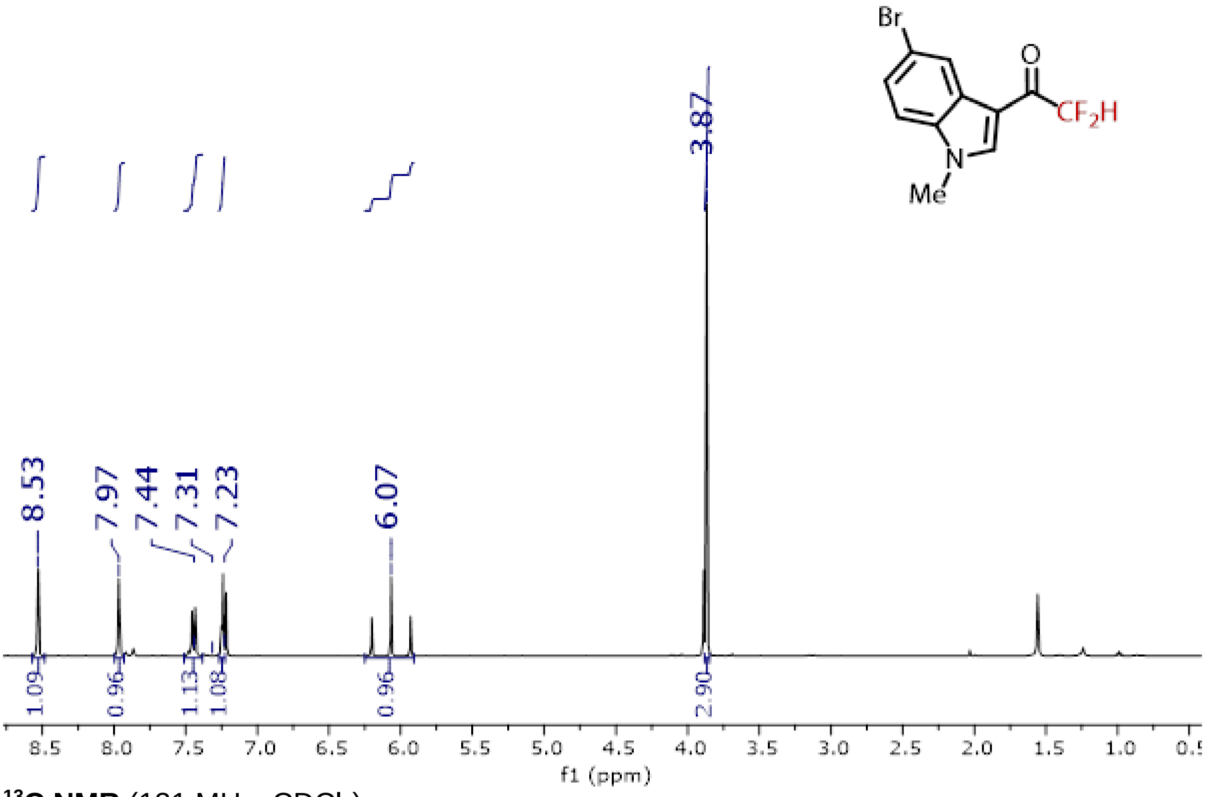

^{13}C NMR (131 MHz, $CDCl_3$):

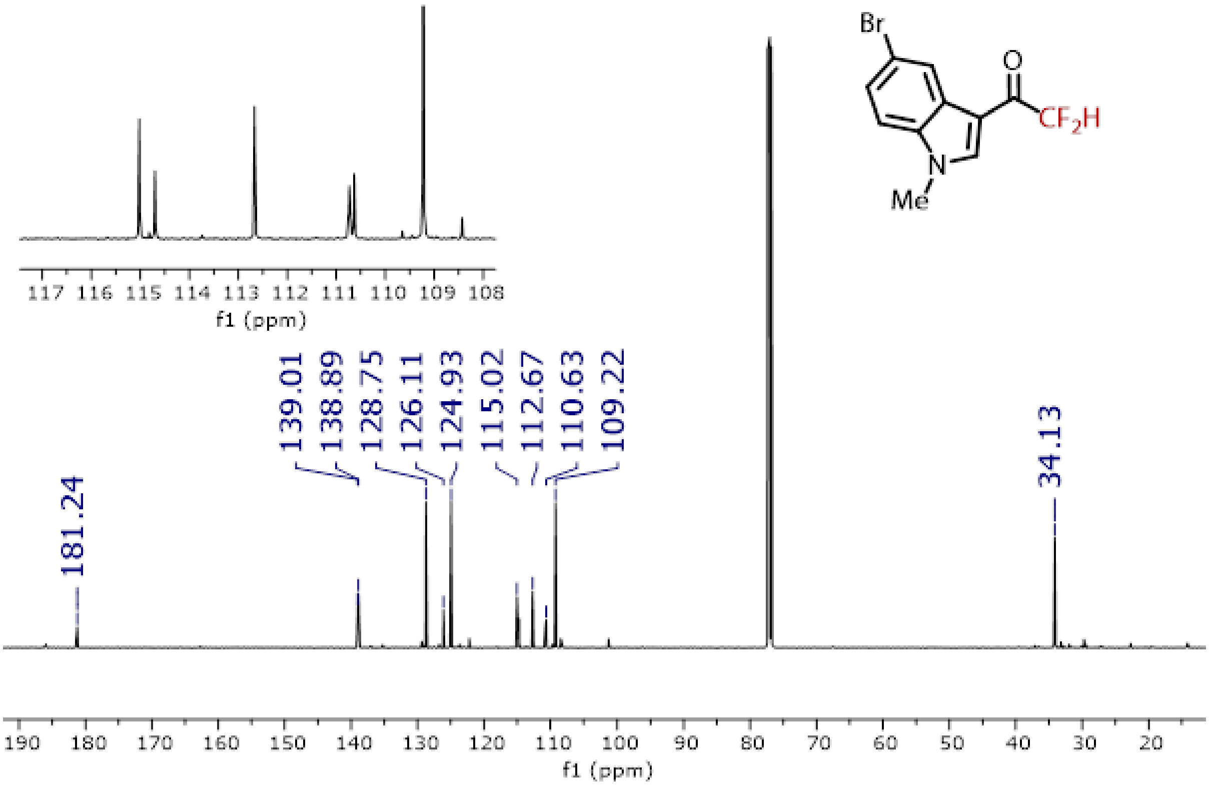

^{19}F NMR (376 MHz, $CDCl_3$):

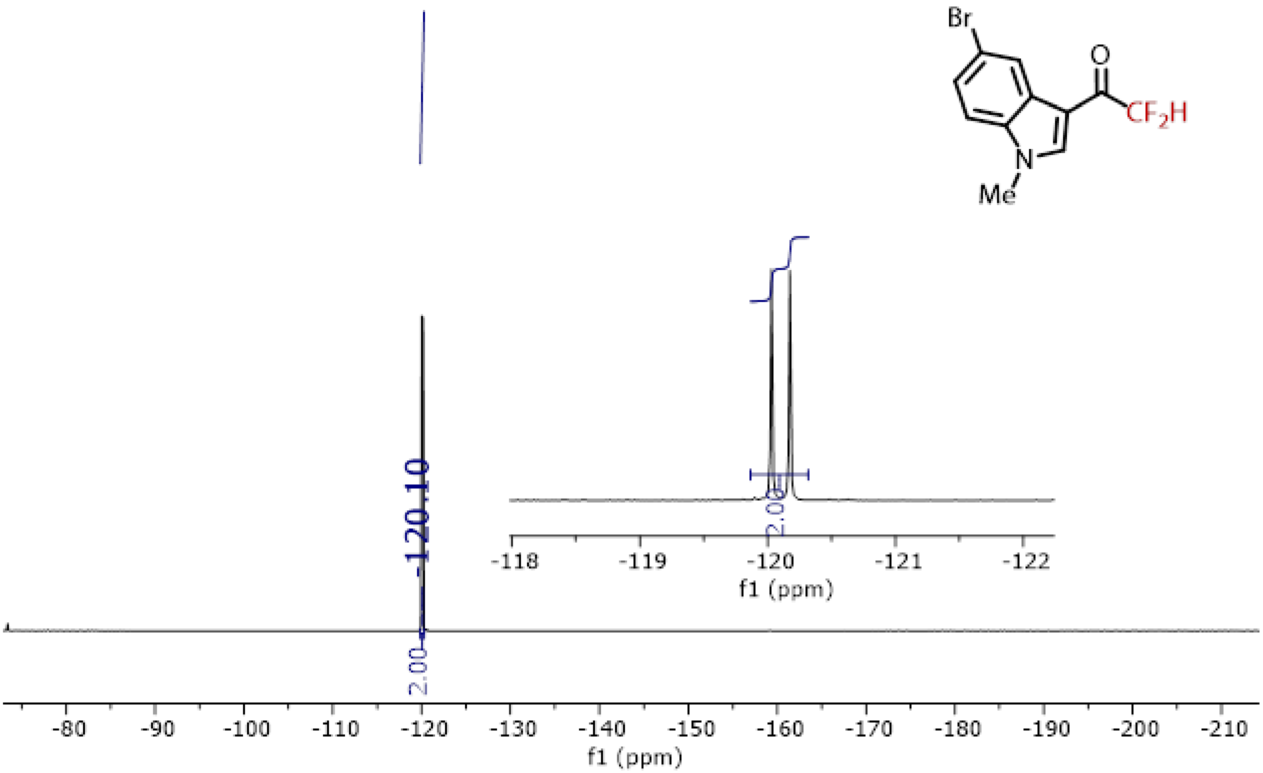

2,2-difluoro-1-(5-iodo-1-methyl-1H-indol-3-yl)ethan-1-one, **3ag**

^{1}H NMR (400 MHz, $CDCl_3$):

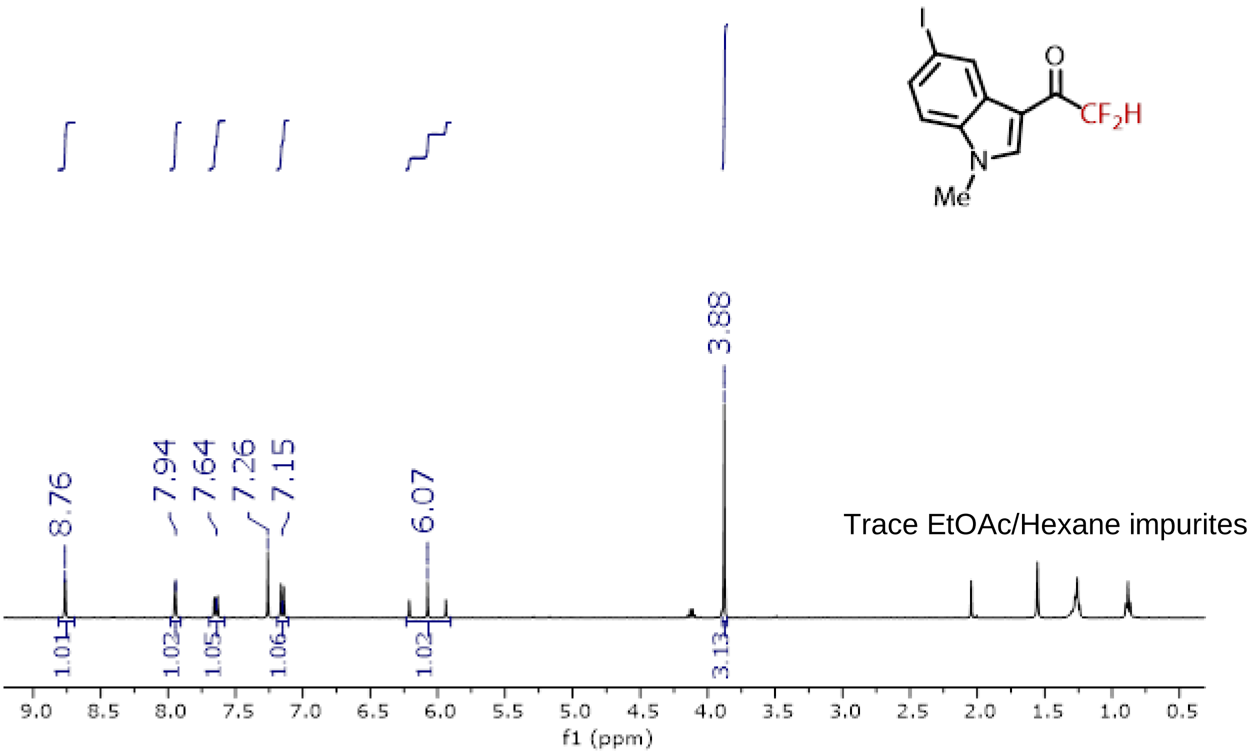

^{13}C NMR (131 MHz, $CDCl_3$):

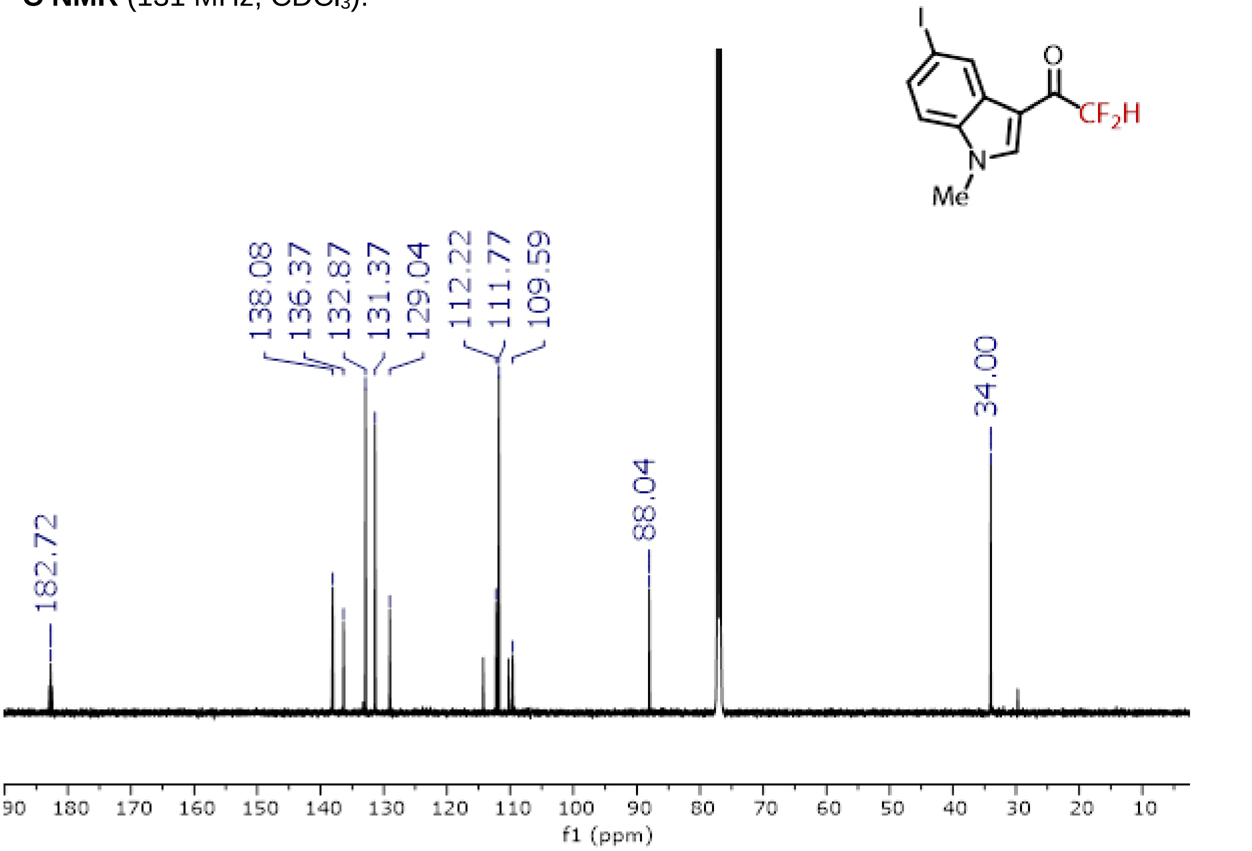

^{19}F NMR (376 MHz, $CDCl_3$):

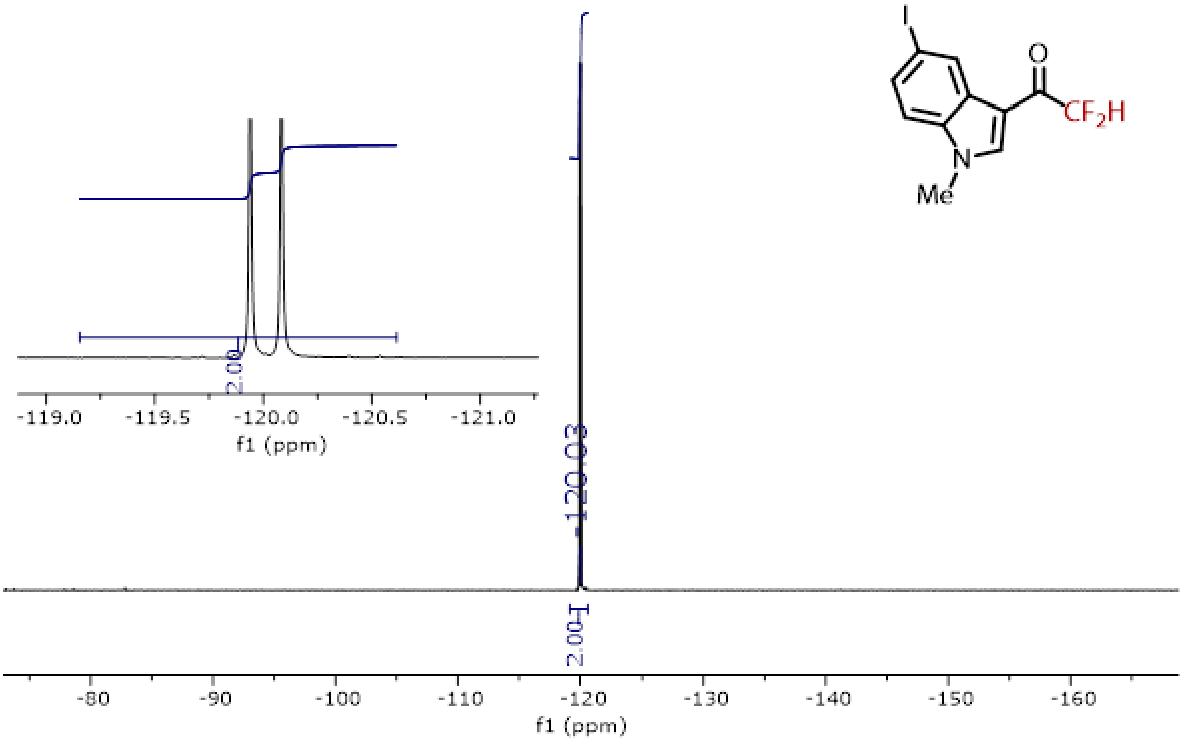

2,2-difluoro-1-(1-methyl-2-(thiophen-2-yl)-1H-indol-3-yl)ethan-1-one, **3ah**

^{1}H NMR (400 MHz, $CDCl_3$):

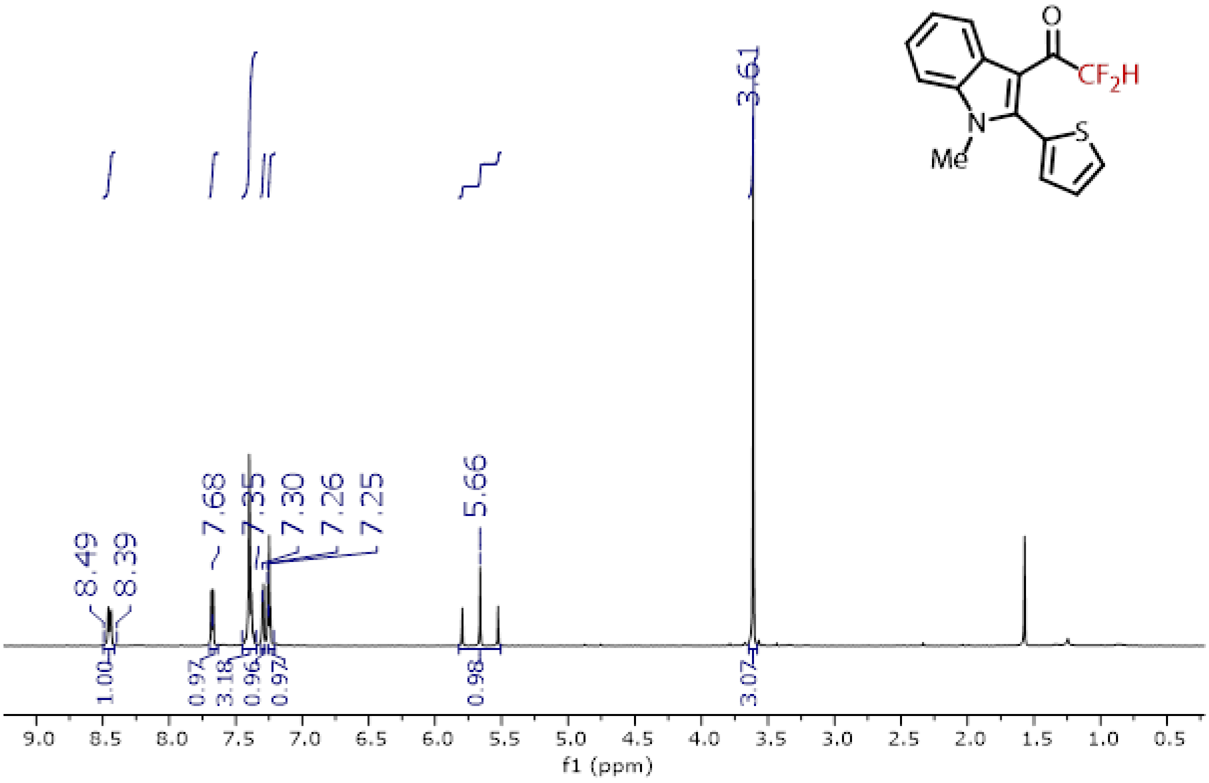

^{13}C NMR (131 MHz, $CDCl_3$):

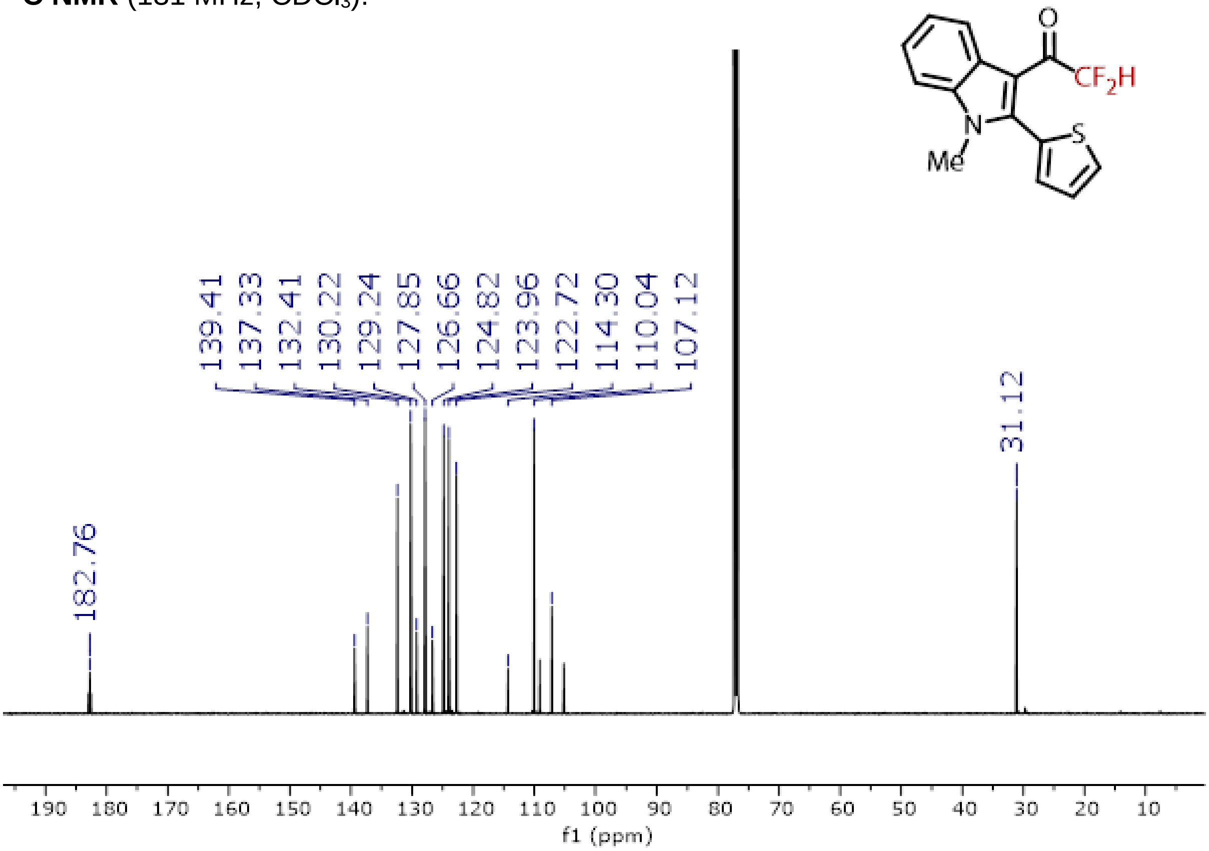

^{19}F NMR (376 MHz, $CDCl_3$):

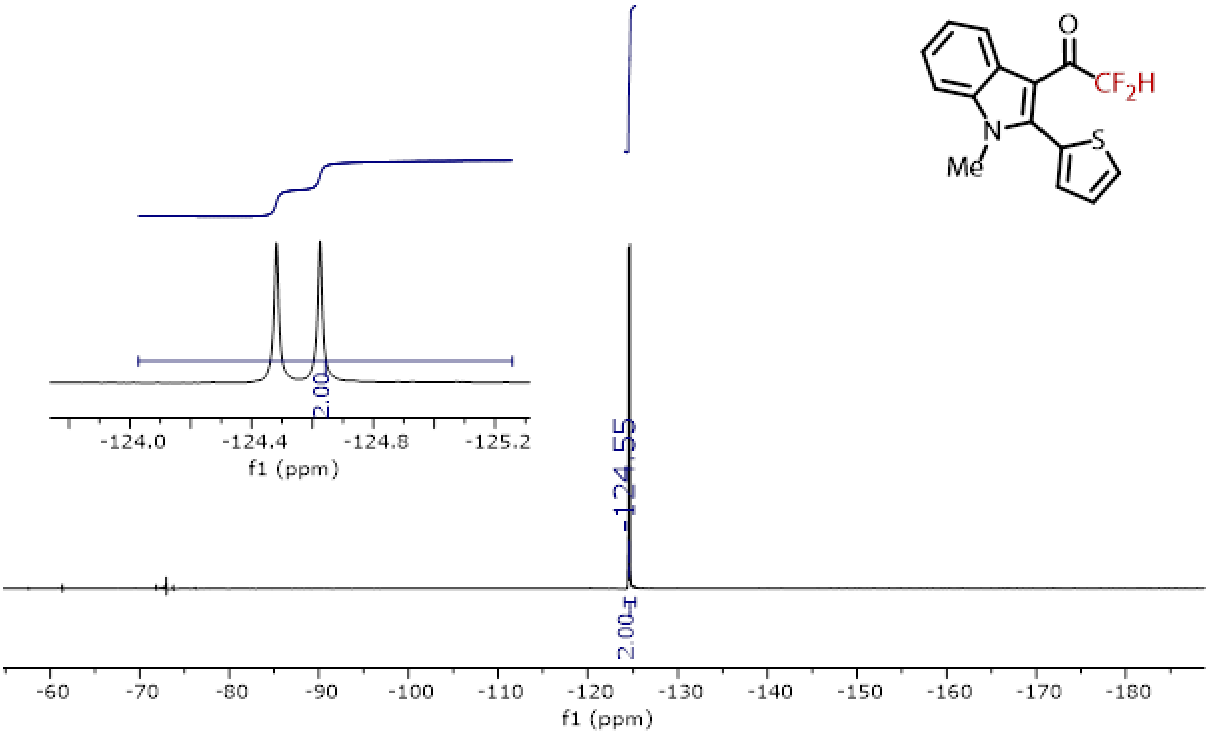

2,2-difluoro-1-(1-methyl-2-phenyl-1H-indol-3-yl)ethan-1-one, **3ai**

^{1}H NMR (400 MHz, $CDCl_3$):

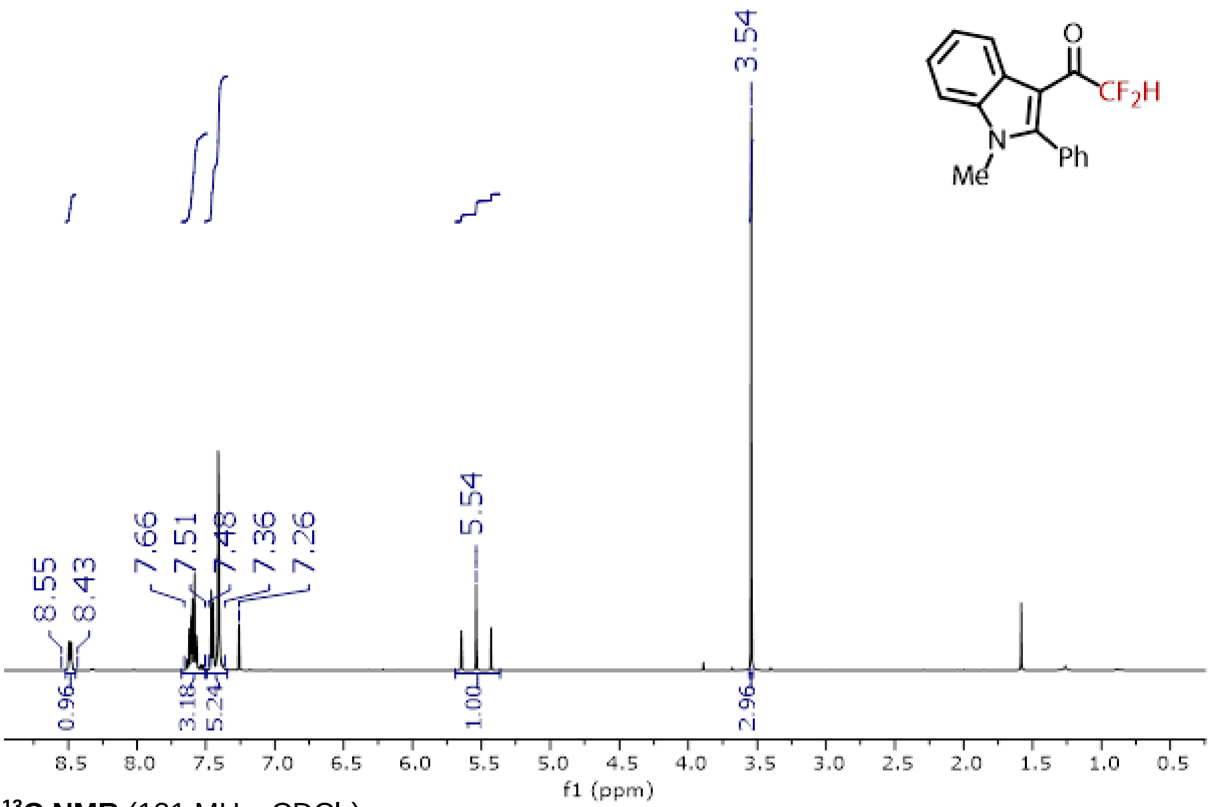

^{13}C NMR (131 MHz, $CDCl_3$):

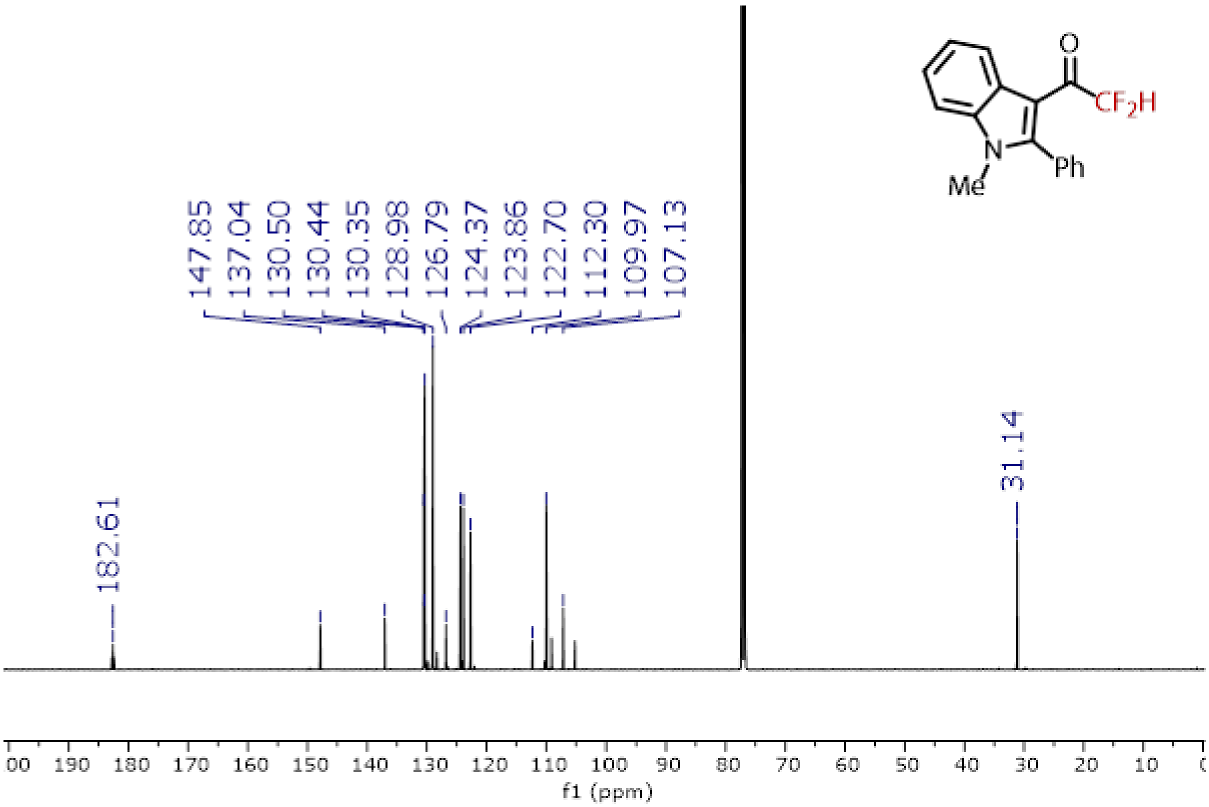

^{19}F NMR (376 MHz, $CDCl_3$):

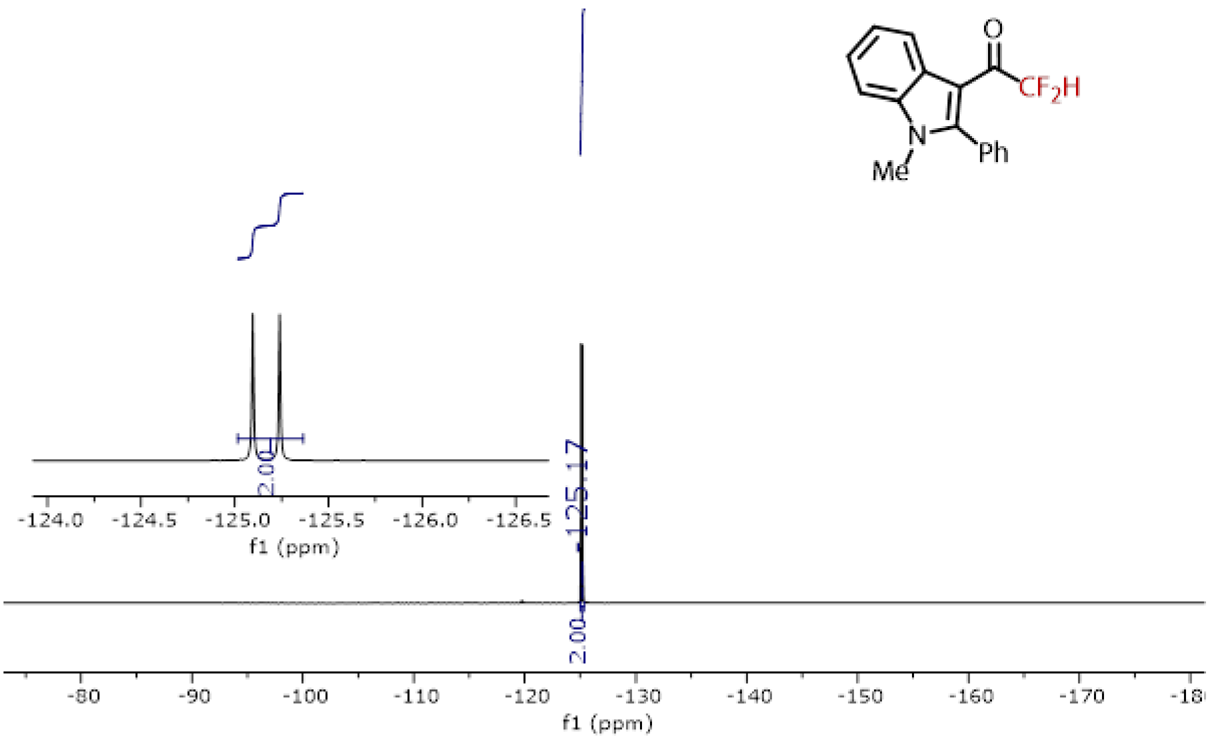

2,2-difluoro-1-(1-methyl-1H-indol-5-yl)ethan-1-one, **3aj**

^{1}H NMR (400 MHz, $CDCl_3$):

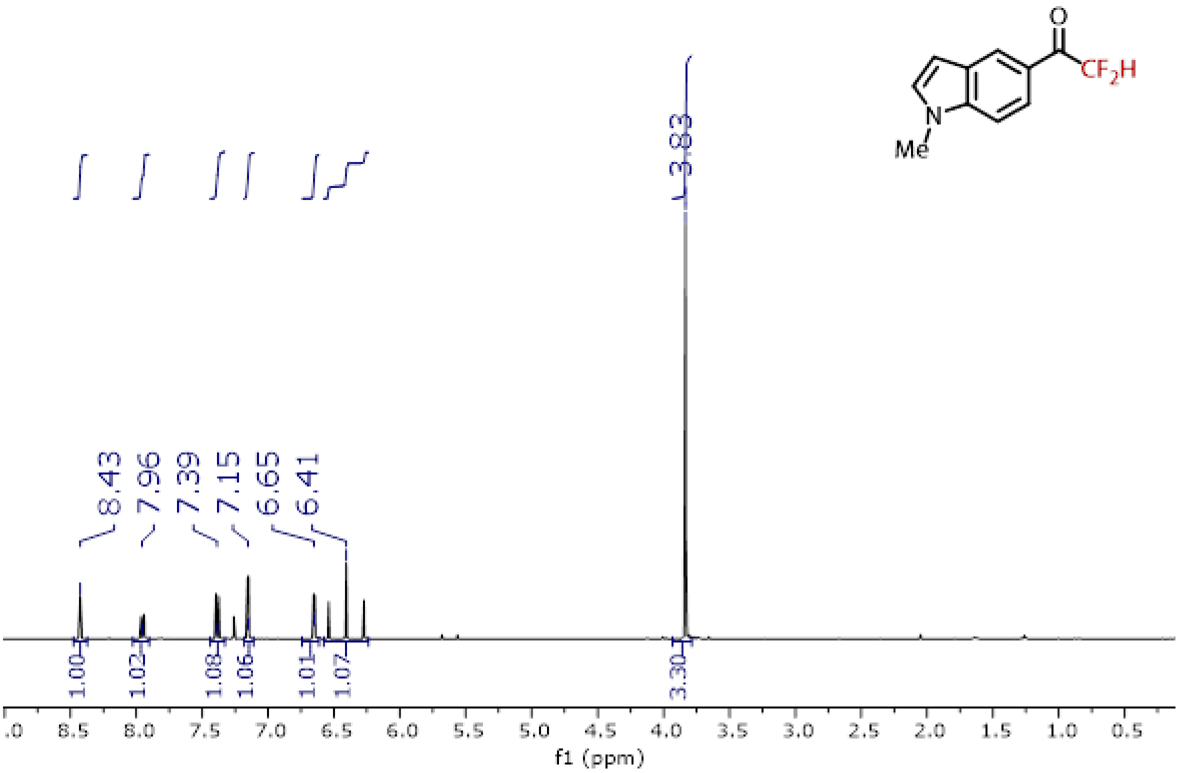

^{13}C NMR (131 MHz, $CDCl_3$):

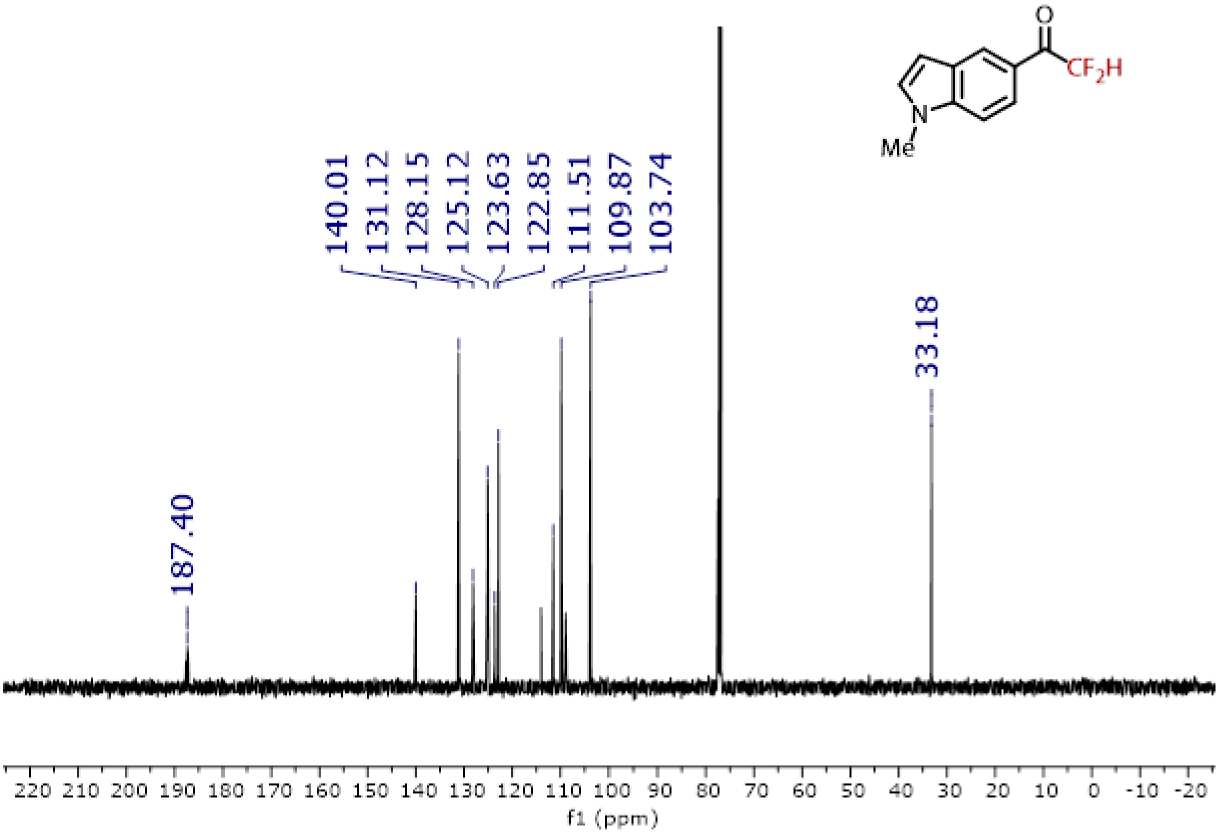

^{19}F NMR (376 MHz, $CDCl_3$):

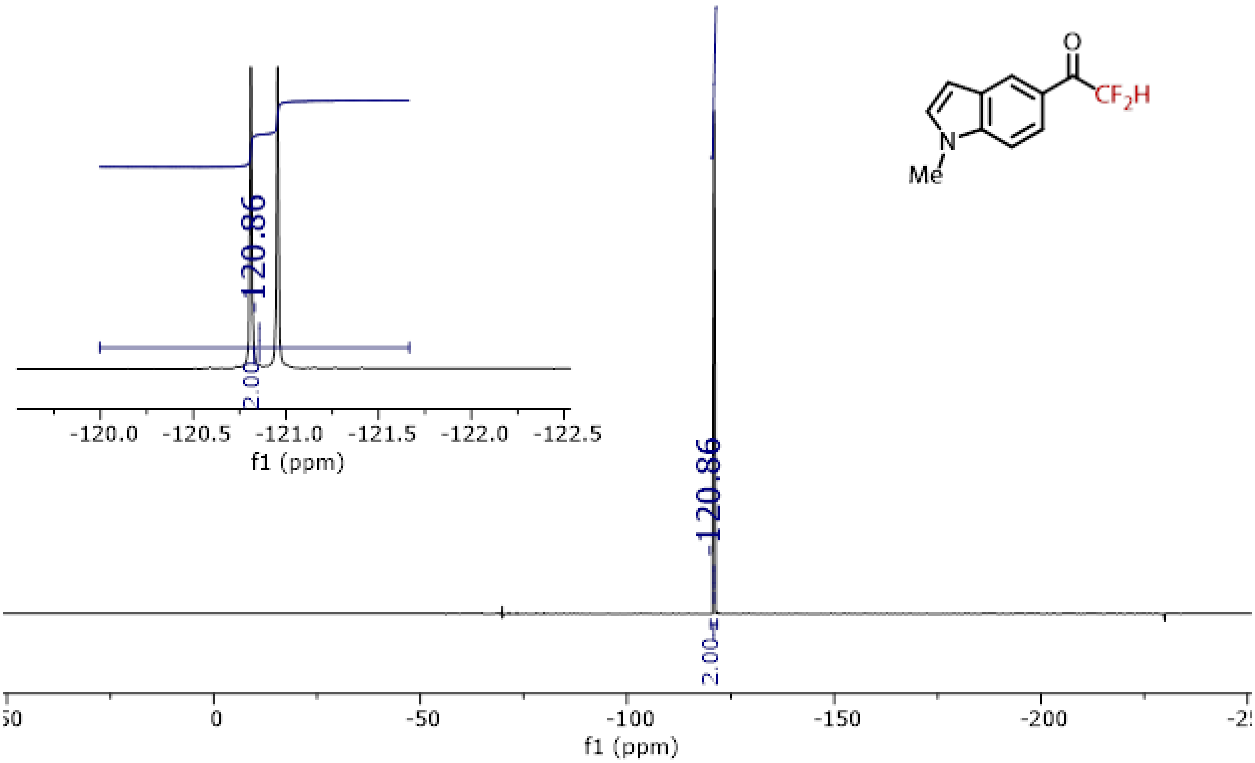

2,2-difluoro-1-(9-methyl-9H-carbazol-3-yl)ethan-1-one, **3ak**

^{1}H NMR (400 MHz, $CDCl_3$):

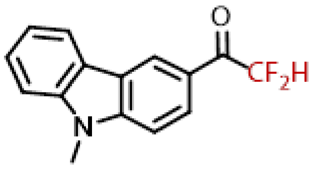

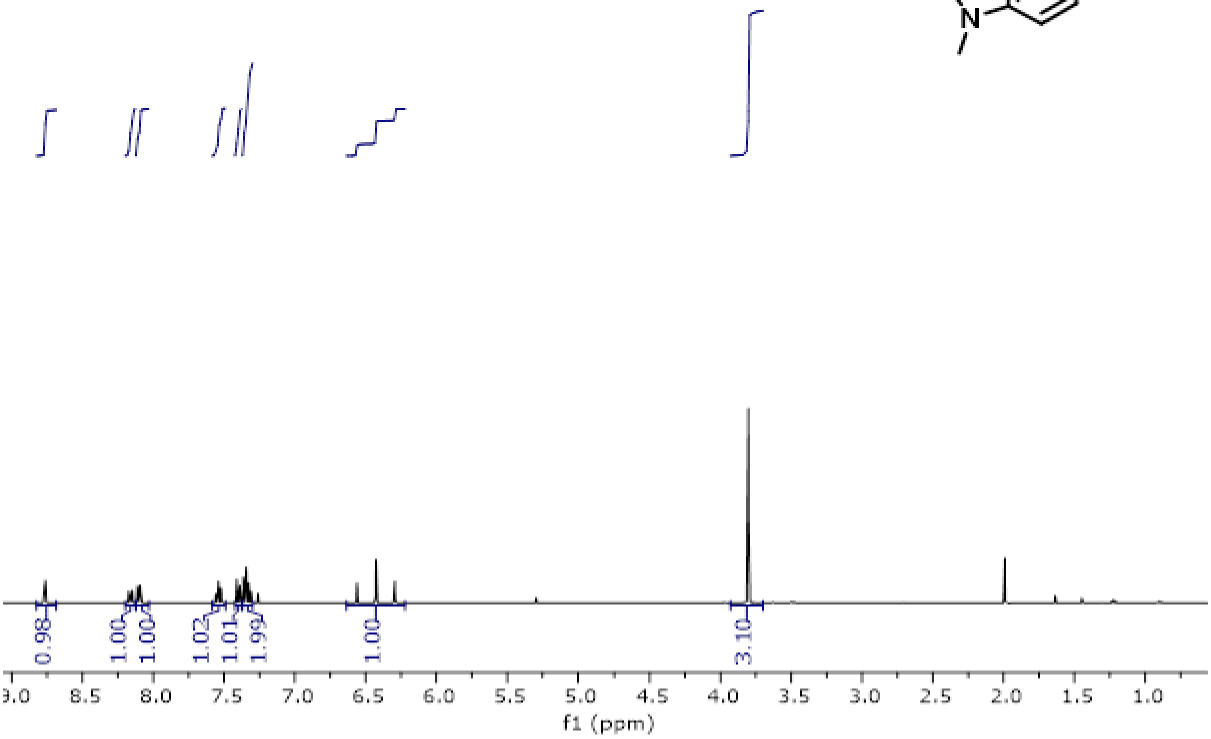

^{13}C NMR (101 MHz, $CDCl_3$):

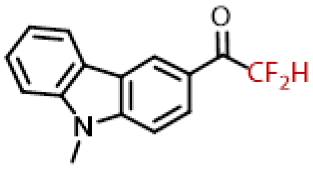

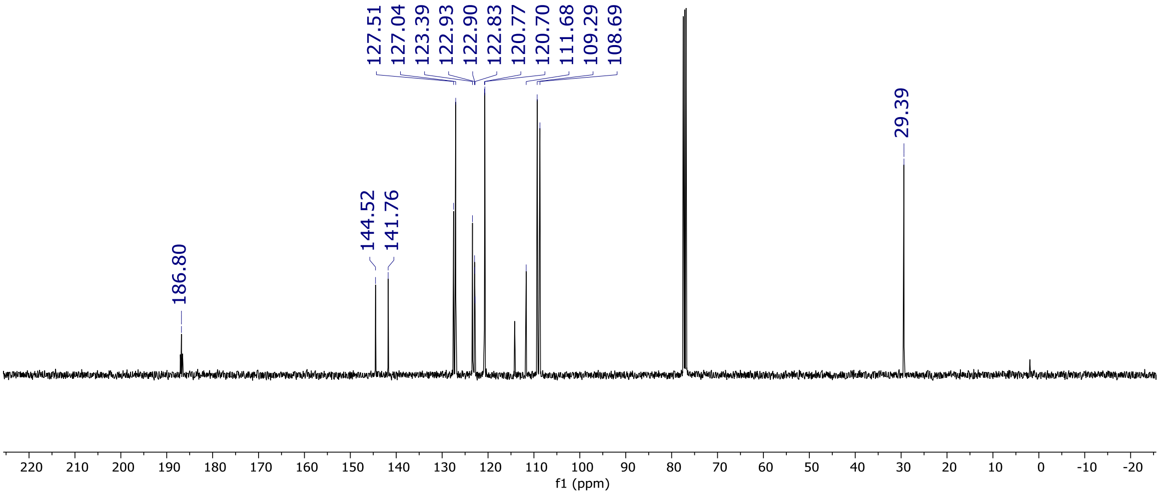

^{19}F NMR (376 MHz, $CDCl_3$):

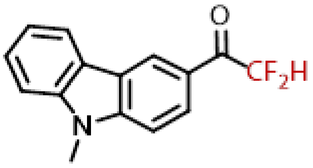

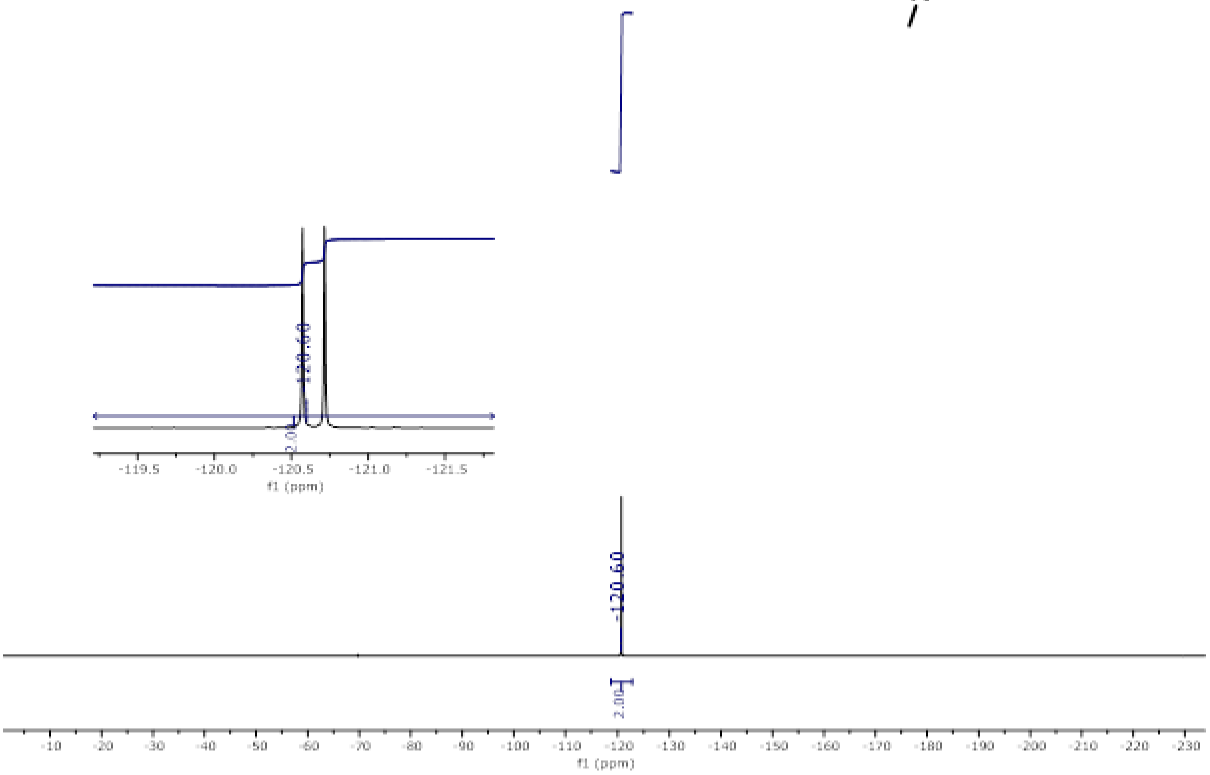

1-(4-(Dimethylamino)pyridin-3-yl)-2,2-difluoroethan-1-one, **3am**

^{1}H NMR (400 MHz, $CDCl_3$):

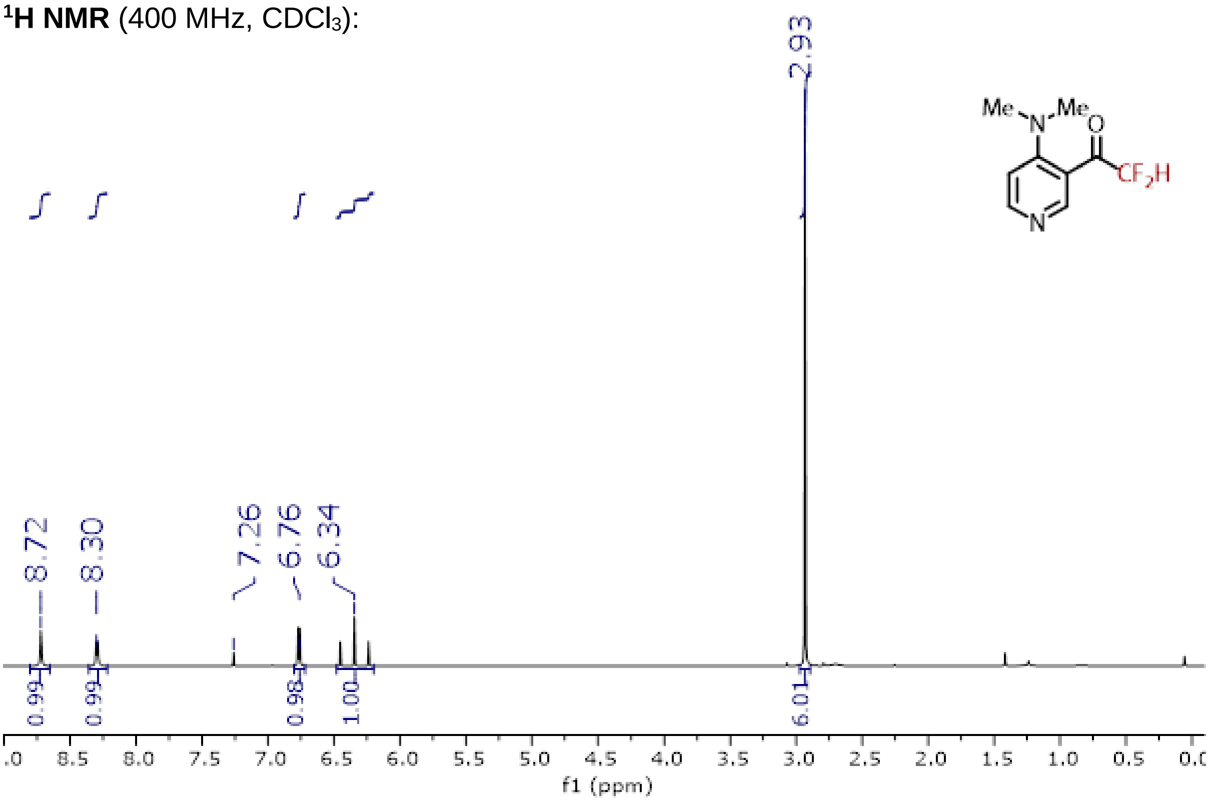

^{13}C NMR (131 MHz, $CDCl_3$):

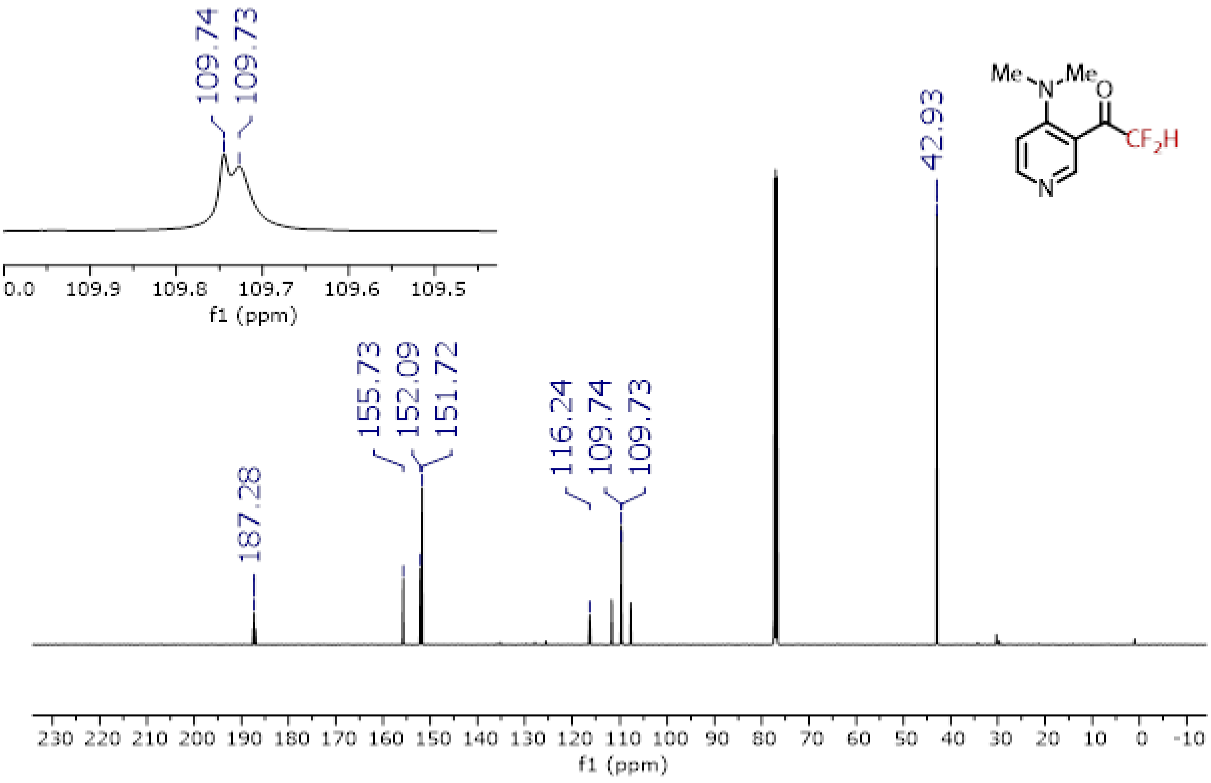

^{19}F NMR (376 MHz, $CDCl_3$):

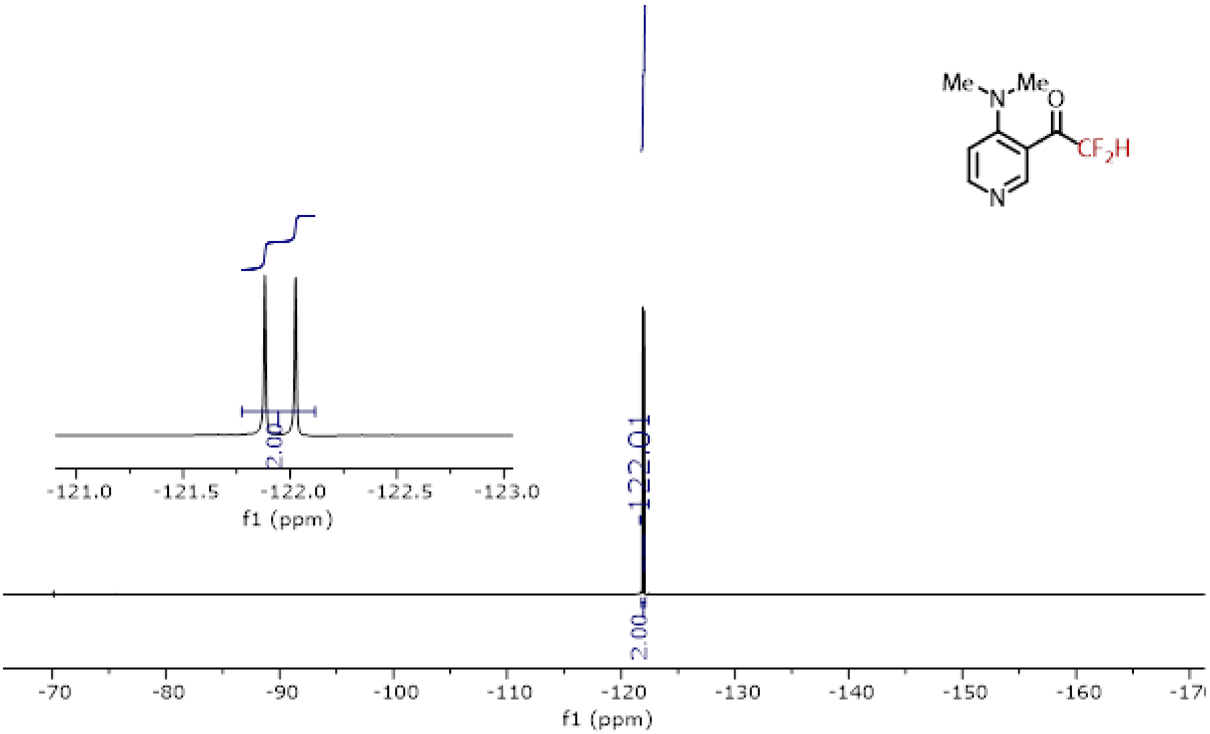

(Z)-1,1-difluorononadec-10-en-2-one, **3ap**

^{1}H NMR (400 MHz, $CDCl_3$):

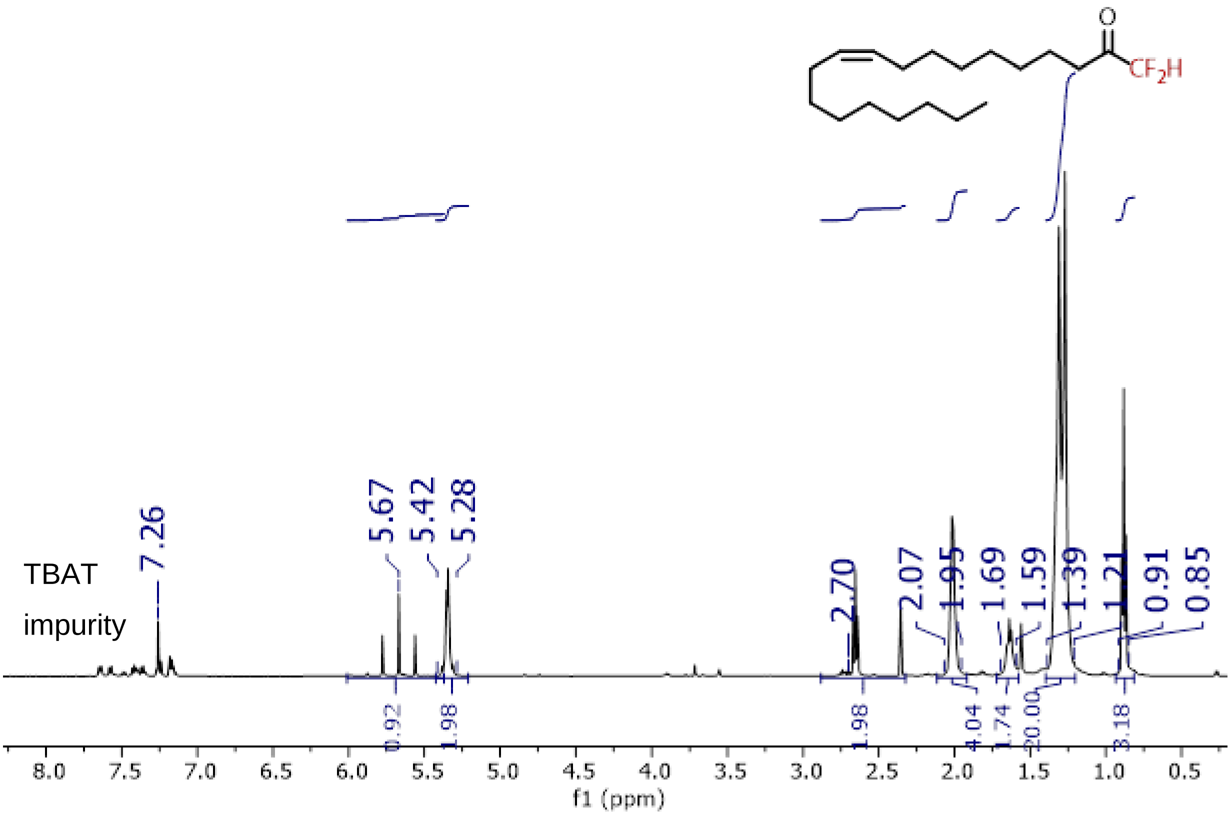

^{13}C NMR (131 MHz, $CDCl_3$):

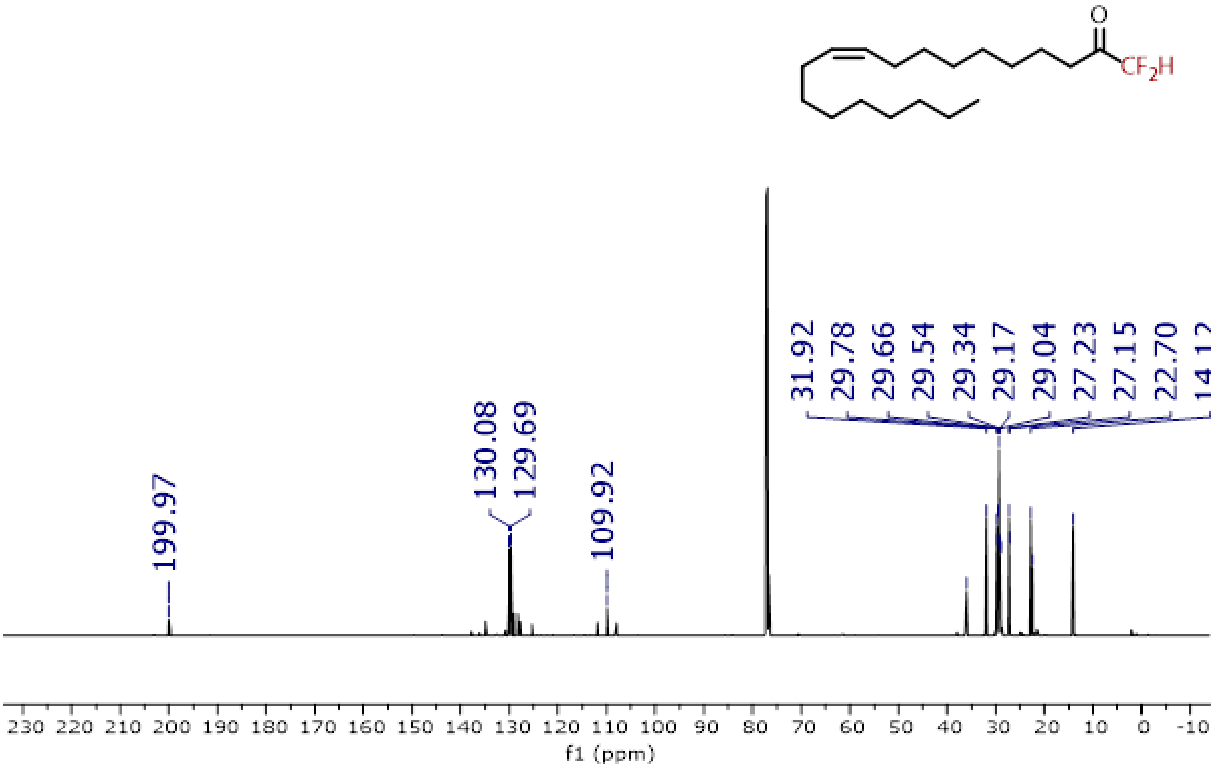

^{19}F NMR (376 MHz, $CDCl_3$):

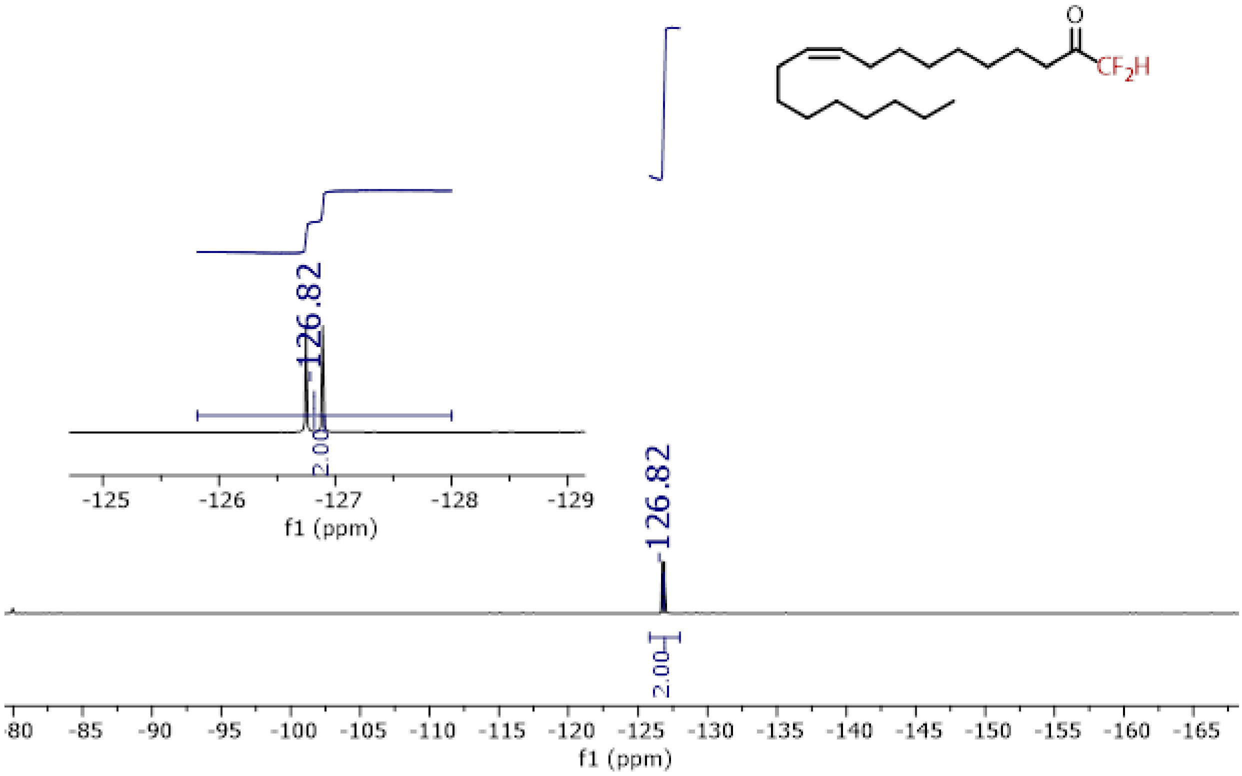

References

(1) Mitchell, L.; Williamson, P.; Ehrlichová, B.; Anderson, A. E.; Seymour, V. R.; Ashbrook, S. E.; Acerbi, N.; Daniels, L. M.; Walton, R. I.; Clarke, M. L.; Wright, P. A. Mixed-Metal MIL-100(Sc,M) (M=Al, Cr, Fe) for Lewis Acid Catalysis and Tandem C□C Bond Formation and Alcohol Oxidation. Chem. - A Eur. J. 2014, 20 (51), 17185–17197. https://doi.org/10.1002/chem.201404377.

(2) Xu, X.; Min, Q. Q.; Li, N.; Liu, F. Visible Light-Promoted Umpolung Coupling of Aryl Tri-/Difluoroethanones with 2-Alkenylpyridines. Chem. Commun. 2018, 54 (78), 11017–11020. https://doi.org/10.1039/c8cc06748a.

(3) Mohr, G. J.; Lehmann, F.; Grummt, U. W.; Spichiger-Keller, U. E. Fluorescent Ligands for Optical Sensing of Alcohols: Synthesis and Characterisation of p-N,N-Dialkylamino-Trifluoroacetylstilbenes. Anal. Chim. Acta 1997, 344 (3), 215–225. https://doi.org/10.1016/S0003-2670(97)00113-X.

(4) Wu, W.; Tian, Q.; Chen, T.; Weng, Z. Copper-Mediated Trifluoroacetylation of Arenediazonium Salts with Ethyl Trifluoropyruvate. Chem. - A Eur. J. 2016, 22 (46), 16455–16458. https://doi.org/10.1002/chem.201604300.

(5) Van Der Mei, F. W.; Qin, C.; Morrison, R. J.; Hoveyda, A. H. Practical, Broadly Applicable, α-Selective, Z-Selective, Diastereoselective, and Enantioselective Addition of Allylboron Compounds to Mono-, Di-, Tri-, and Polyfluoroalkyl Ketones. J. Am. Chem. Soc. 2017, 139 (26), 9053–9065. https://doi.org/10.1021/jacs.7b05011.

(6) Yan, G.; Cao, X.; Zheng, W.; Ke, Q.; Zhang, J.; Huang, D. Copper-Mediated Trifluoroacetylation of Indoles with Ethyl Trifluoropyruvate. Org. Biomol. Chem. 2017, 15 (28), 5904–5907. https://doi.org/10.1039/c7ob01335c.

(7) US Pat., 2003/0004157, 2003.

(8) Brüning, F.; Nagae, H.; Käch, D.; Mashima, K.; Togni, A. Asymmetric Hydrogenation of Aryl Perfluoroalkyl Ketones Catalyzed by Rhodium(III) Monohydride Complexes Bearing Josiphos Ligands. Chem. – A Eur. J. 2019, 25 (46), 10818–10822. https://doi.org/10.1002/chem.201902585.

(9) Heinz, B.; Djukanovic, D.; Ganiek, M. A.; Martin, B.; Schenkel, B.; Knochel, P. Selective Acylation of Aryl-and Heteroarylmagnesium Reagents with Esters in Continuous Flow. Org. Lett. 2020, 22 (2), 493–496. https://doi.org/10.1021/acs.orglett.9b04254.

(10) Funabiki, K.; Hayakawa, A.; Inuzuka, T. Convenient, Functional Group-Tolerant, Transition Metal-Free Synthesis of Aryl and Heteroaryl Trifluoromethyl Ketones with the Use of Methyl Trifluoroacetate. Org. Biomol. Chem. 2018, 16 (6), 913–918. https://doi.org/10.1039/c7ob02862h.

(11) Yarmoliuk, D. V.; Arkhipov, V. V.; Stambirskyi, M. V.; Dmytriv, Y. V.; Shishkin, O. V.; Tolmachev, A. A.; Mykhailiuk, P. K. Direct Noncatalytic Electrophilic Trifluoroacetylation of Electron-Rich Pyrazoles. Synth. 2014, 46 (9), 1254–1260. https://doi.org/10.1055/s-0033-1340840.

(12) Camerino, E.; Wong, D. M.; Tong, F.; Körber, F.; Gross, A. D.; Islam, R.; Viayna, E.; Mutunga, J. M.; Li, J.; Totrov, M. M.; Bloomquist, J. R.; Carlier, P. R. Difluoromethyl Ketones: Potent Inhibitors of Wild Type and Carbamate-Insensitive G119S Mutant Anopheles Gambiae Acetylcholinesterase. Bioorganic Med. Chem. Lett. 2015, 25 (20), 4405–4411. https://doi.org/10.1016/j.bmcl.2015.09.019.

(13) U.S., 6096784, 01 Aug 2000.

(14) Tian, T.; Zhong, W. H.; Meng, S.; Meng, X. B.; Li, Z. J. Hypervalent Iodine Mediated Para -Selective Fluorination of Anilides. J. Org. Chem. 2013, 78 (2), 728–732. https://doi.org/10.1021/jo302099d.

(15) Leng, D. J.; Black, C. M.; Pattison, G. One-Pot Synthesis of Difluoromethyl Ketones by a Difluorination/Fragmentation Process. Org. Biomol. Chem. 2016, 14 (5), 1531–1535. https://doi.org/10.1039/c5ob02468d.

(16) Miele, M.; D'Orsi, R.; Sridharan, V.; Holzer, W.; Pace, V. Highly Chemoselective Difluoromethylative Homologation of Iso(Thio)Cyanates: Expeditious Access to Unprecedented α,α-Difluoro(Thio)Amides. Chem. Commun. 2019, 55 (86), 12960–12963. https://doi.org/10.1039/c9cc06929a.

(17) Schmitt, E.; Rugeri, B.; Panossian, A.; Vors, J. P.; Pazenok, S.; Leroux, F. R. In Situ Generated Fluorinated Iminium Salts for Difluoromethylation and Difluoroacetylation. Org. Lett. 2015, 17 (18), 4510–4513. https://doi.org/10.1021/acs.orglett.5b02184.

(18) de Azambuja, F.; Yang, M. H.; Feoktistova, T.; Selvaraju, M.; Brueckner, A. C.; Grove, M. A.; Koley, S.; Cheong, P. H. Y.; Altman, R. A. Connecting Remote C–H Bond Functionalization and Decarboxylative Coupling Using Simple Amines. Nat. Chem. 2020, 12 (5), 489–496. https://doi.org/10.1038/s41557-020-0428-1.

(19) Zhang, Y.; Yan, W.; Wang, Y.; Weng, Z. Copper-Catalyzed Synthesis of Indol-3-Yl α-(Difluoromethyl)-α-(Trifluoromethyl)Carbinols: Construction of Difluoromethylated Sp3 Carbon Centers. Org. Lett. 2017, 19 (19), 5478–5481. https://doi.org/10.1021/acs.orglett.7b02828.

(20) Yi, X.; Cao, Y. F.; Wang, X.; Xu, H.; Ban, S. R.; Dai, H. X. Palladium-Catalyzed Fluoroacylation of (Hetero)Arylboronic Acid with Fluorothioacetates at Ambient Temperature. Tetrahedron Lett. 2020, 61 (16), 151780. https://doi.org/10.1016/j.tetlet.2020.151780.

(21) Prieto, A.; Melot, R.; Bouyssi, D.; Monteiro, N. Palladium-Catalyzed C(Sp 2)−H Alkylation of Aldehyde-Derived Hydrazones with Functionalized Difluoromethyl Bromides. Angew. Chemie Int. Ed. 2016, 55 (5), 1885–1889. https://doi.org/10.1002/anie.201510334.

(22) Mizoi, K.; Mashima, Y.; Kawashima, Y.; Takahashi, M.; Mimori, S.; Hosokawa, M.; Murakami, Y.; Hamana, H. A New Methodology for Functionalization at the 3-Position of Indoles by a Combination of Boron Lewis Acid with Nitriles. Chem. Pharm. Bull. (Tokyo). 2015, 63 (7), 538–545. https://doi.org/10.1248/cpb.c15-00157.

(23) Sessler, C. D.; Rahm, M.; Becker, S.; Goldberg, J. M.; Wang, F.; Lippard, S. J. CF2H, a Hydrogen Bond Donor. J. Am. Chem. Soc. 2017, 139 (27), 9325–9332. https://doi.org/10.1021/jacs.7b04457.

(24) Abraham, M. H.; Abraham, R. J.; Acree, W. E.; Aliev, A. E.; Leo, A. J.; Whaley, W. L. An NMR Method for the Quantitative Assessment of Intramolecular Hydrogen Bonding; Application to Physicochemical, Environmental, and Biochemical Properties. J. Org. Chem. 2014, 79 (22), 11075–11083. https://doi.org/10.1021/jo502080p.

(25) Bruker, SAINT+ v8.38A Integration Engine, Data Reduction Software, Bruker Analytical X-ray Instruments Inc., Madison, WI, USA, 2015.

(26) Bruker, SADABS 2014/5, Bruker AXS area detector scaling and absorption correction, Bruker Analytical X-ray Instruments Inc., Madison, Wisconsin, USA, 2014/5.

(27) Sheldrick, G. M. SHELXT - Integrated Space-Group and Crystal-Structure Determination. Acta Crystallogr. Sect. A Found. Crystallogr. 2015, 71 (1), 3–8. https://doi.org/10.1107/S2053273314026370.

(28) Sheldrick, G. M. A Short History of SHELX. Acta Crystallographica Section A: Foundations of Crystallography. International Union of Crystallography January 1, 2008, pp 112–122. https://doi.org/10.1107/S0108767307043930.

(29) Dolomanov, O. V.; Bourhis, L. J.; Gildea, R. J.; Howard, J. A. K.; Puschmann, H. OLEX2: A Complete Structure Solution, Refinement and Analysis Program. J. Appl. Crystallogr. 2009, 42 (2), 339–341. https://doi.org/10.1107/S0021889808042726.
